# Correlation Consistent Basis Sets for Explicitly Correlated Theory: The Transition Metals

**DOI:** 10.1021/acs.jctc.3c00506

**Published:** 2023-08-04

**Authors:** Emmanouil Semidalas, Jan M. L. Martin

**Affiliations:** Department of Molecular Chemistry and Materials Science, Weizmann Institute of Science, 7610001 Reḥovot, Israel

## Abstract

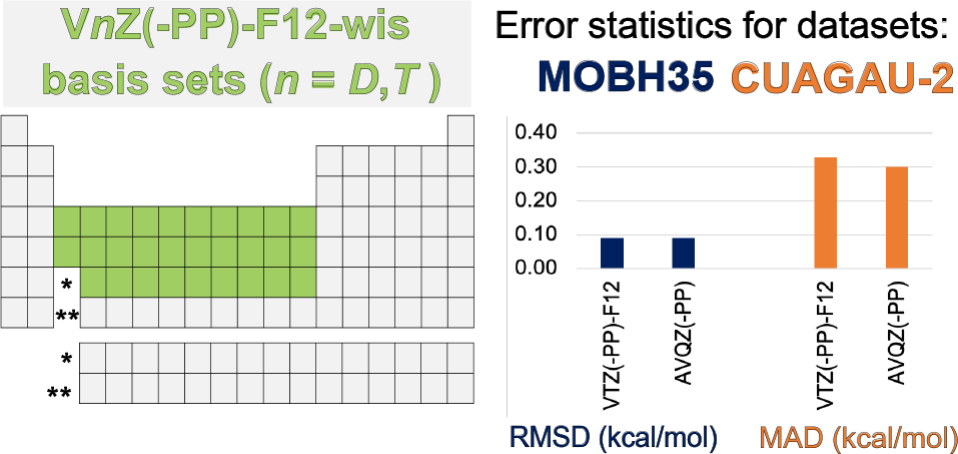

We present correlation consistent basis sets for explicitly correlated (F12) calculations, denoted V*n*Z(-PP)-F12-wis (*n* = D,T), for the *d*-block elements. The cc-pVDZ-F12-wis basis set is contracted to [8s7p5d2f] for the 3*d*-block, while its ECP counterpart for the 4*d* and 5*d*-blocks, cc-pVDZ-PP-F12-wis, is contracted to [6s6p5d2f]. The corresponding contracted sizes for cc-pVTZ(-PP)-F12-wis are [9*s*8*p*6*d*3*f*2*g*] for the 3*d*-block elements and [7*s*7*p*6*d*3*f*2*g*] for the 4*d* and 5*d*-block elements. Our V*n*Z(-PP)-F12-wis basis sets are evaluated on challenging test sets for metal–organic barrier heights (MOBH35) and group-11 metal clusters (CUAGAU-2). In F12 calculations, they are found to be about as close to the complete basis set limit as the combination of standard cc-pV*n*Z-F12 on *main-group elements* with the standard aug-cc-pV(*n*+1)Z(-PP) basis sets on the transition metal(s). While our basis sets are somewhat more compact than aug-cc-pV(*n*+1)Z(-PP), the CPU time benefit is negligible for catalytic complexes that contain only one or two transition metals among dozens of main-group elements; however, it is somewhat more significant for metal clusters.

## 1 Introduction

In explicitly correlated wave function approaches,^1−7^ the orbital basis set is enhanced with geminal terms that depend explicitly on interelectronic distances. They converge much more rapidly to the complete basis set limit (CBS) than conventional orbital approaches: asymptotically as L^–7^ compared to L^–3^, where *L* represents the largest angular momentum in the basis set.^8^ Hence, they potentially allow for greater accuracy at a much lower cost in computation time and resources. This has been demonstrated for, *inter alia*, total atomization energies,^9−11^ noncovalent interactions,^12−15^ and harmonic frequencies.^16,17^ For instance, the MP2 total atomization energies of acetylene, ethylene, and ethane, extrapolated from augmented *spdfgh* and *spdfghi* basis sets, are within ∼0.1 kcal/mol from results^18^ obtained using explicitly correlated MP2-F12 with a truncated *spdf* basis set.^19^

For the specific geminal form, Ten-no’s F12 geminals^3,5^ of the form F(r_12_) = (1 – exp(γ*r*_12_))/γ have become the *de facto* standard: for reasons of computational convenience, inspired by the pioneering work of Persson and Taylor,^20^ the Slater function is approximated as a linear combination of (usually six) Gaussians.

The accuracy of F12 methods is strongly dependent on the choice of the orbital basis set (OBS). While F12 OBSs for main group elements have been extensively studied, such as the cc-pV*n*Z-F12 (*n* = D, T, Q) basis sets by Peterson and co-workers,^21,22^ and the work of Martin and co-workers on the optimization of cc-pV5Z-F12 basis sets^11,23^ and their augmented variants,^24^ systematic investigations on *d*-block elements have only begun recently, with Shaw and Hill presenting correlation consistent basis sets for valence and core–valence correlation in the context of explicitly correlated theory for the group 11 and 12 elements.^25^

To use the F12 approaches in programs such as ORCA,^26^ MOLPRO,^27^ and TURBOMOLE,^28^ appropriate OBSs and auxiliary basis sets (ABS) must be specified. In particular, an RI-JK ABS is used to fit the Coulomb (J) and exchange integrals (K), an RI-MP2 ABS for the RI-MP2 step, and a complementary auxiliary basis set^29^ (CABS, also known as OptRI) for the computation of the F12-specific matrix elements. In practice, the ABSs utilized for density-fitted Hartree–Fock (DF-HF) and density fitted MP2 (DF-MP2) are the same ones used for F12 calculations. Additionally, explicitly correlated methods have been implemented in various contexts, including CCSD-F12b in the W4–F12 theory,^11^ and more recently, MP2-F12 as a substitute for the slower MP2 step in G4-type composite schemes^30^ (cc-G4-F12-T and cc-G4-F12-T-DLPNO) and in density functional theory (DFT) for the GLPT2 step.^31,32^

This study aims to optimize orbital basis sets for *d*-block elements within explicitly correlated theory. To that end, we optimize the *fg* exponents of the correlation consistent V*n*Z(-PP)-F12-wis (*n* = D, T) basis sets with respect to the state-averaged MP2-F12 energy of the atoms. The performance of these basis sets is evaluated for molecular calculations using the Iron and Janes’ MOBH35 dataset^33,34^ of real-life organometallic chemistry reactions and Chan’s CUAGAU-2 dataset^35^ of group 11 metal clusters. The optimized V*n*Z(-PP)-F12-wis basis sets, when employed in explicitly correlated theory, exhibit accuracy comparable to that of the standard AV(*n* + 1)Z(−PP) basis sets while being more economical and compact. Also, we perform timing comparisons, employing polyoxometalate anion Mo_6_O_19_^–2^ as a test subject to assess the efficiency of the optimized basis sets. These basis sets are expected to be useful for future explicitly correlated theory calculations.

## 2 Computational Details

All calculations in this work were performed on the ChemFarm high-performance computing cluster of the Faculty of Chemistry at the Weizmann Institute of Science, utilizing the program suites MOLPRO2022,^27^ ORCA 5.0.3,^26^ and MRCC2022.^36,37^ Density fitting approximations were employed to Hartree–Fock (DF-HF),^38^ second-order Møller–Plesset perturbation theory (DF-MP2),^39,40^ and explicitly correlated DF-MP2-F12,^41^ to efficiently compute the transformed 2-index and 3-index integrals. The 3C(Fix) ansatz^41,42^ was used for DF-MP2-F12 calculations in MOLPRO with consideration of the complementary auxiliary basis set (CABS) correction terms.^29^ For systems with unpaired electrons, we used the restricted open-shell variant of ROHF-RMP2-F12,^43^ which relied on restricted open-shell HF (ROHF) orbitals. A uniform β geminal exponent of 1.4 is commonly employed for basis set optimization in DF-MP2-F12 calculations, following prior works on basis set development^21,23,25,44^ in explicitly correlated theory. The physical meaning of setting the value of β high is effectively restricting the geminal to a short range. Stringent accuracy settings, thrcabs = 1d–9 and thrcabs_rel = 1d-9, were used to avoid numerical instabilities and include all necessary basis functions.

Spherical harmonics rather than Cartesian Gaussians were used throughout. Details on the orbital basis set construction process and the selection of appropriate auxiliary basis sets for atomic calculations can be found in section 3.A.

For comparison, we carried out conventional MP2 or density-fitted DF-MP2 molecular calculations in several orbital basis sets, such as cc-pV*n*Z (*n* = Q, 5)^45−49^ and their augmented variants,^45−50^ for both main group and 3*d*-block elements (*Z* = 21–30), cc-pV*n*Z-PP for bromine^51^ and iodine,^51,52^ and cc-pV*n*Z-PP^53,54^ and aug-cc-pV*n*Z-PP^53,54^ (*n* = Q, 5) for 4*d* (*Z* = 39–48) and 5*d* (*Z* = 72–80) block elements. These basis sets, referred to in the present paper as V*n*Z(-PP) and AV*n*Z(-PP), were retrieved from the electronic structure code libraries or the Basis Set Exchange Python library.^55^ As auxiliary basis sets in DF-MP2, we selected V*n*Z/MP2Fit for main group^56,57^ and 3*d*-block elements,^58^ and V*n*Z-PP/MP2Fit for halogens,^59^ 4*d*,^60^ and 5*d*-block^61^ elements with *n* = Q and 5.

In the explicitly correlated DF-MP2-F12 calculations, the V*n*Z-F12 (*n* = D, T)^21^ basis sets for main group elements were used as the OBS (orbital basis set) for those elements, and the corresponding V*n*Z-F12/MP2Fit by Kritikou and Hill^62^ for density fitting in PT2. For *d*-block elements, aside from our proposed orbital basis sets, we considered AV*n*Z^46^ (*n*= T, Q) for 3*d*-block elements and AV*n*Z-PP^53,54^ for 4*d*- and 5*d*-block elements. The awCVTZ(-PP)/MP2Fit^58,61,63^ and AVQZ-PP/MP2Fit^58,60,61^ (with up to *I* functions) were also used as auxiliary basis sets in MP2-F12 for *d*-block elements. The V*n*Z-PP-F12/MP2Fit reported by Hill and Peterson^44^ were chosen for halogens as the DF-MP2 ABS. The def2-QZVPP/JKFit^64^ was used as both JKFit and CABS.

With regard to effective core pseudopotentials, we utilized the ECP28MDF^53^ for elements Y through Cd, and the ECP60MDF^54^ for elements Hf through Hg.

The Hartree–Fock component in orbital-only calculations was extrapolated according to ref (65), namely, *E*_∞_ = *E*(*L*) + α[*E*(*L*) – *E*(*L* – 1)](*L*)/(*L* + 1), where α =  with β = 5.34 for both V{T,Q}Z and AV{T,Q}Z, while for V{Q,5}Z and AV{Q,5}Z, β = 8.74 was used, respectively, and *L* is the cardinal number of the basis set. This was combined with a two-point extrapolation for the MP2 correlation energy according to Halkier et al.,^66^ with *E*_∞_ = *E*(*L*) + (*E*(*L*) – *E*(*L* – 1)/((*L*/*L* – 1)^3^ – 1). For validation purposes, we considered: (a) Iron and Janes’ MOBH35 dataset of the metal–organic barrier heights of 35 reactions^33,34^ (compiled from real-life homogeneous catalysis problems); (b) Chan’s CUAGAU-2 dataset group-11 metal clusters.^35^ The geometries were obtained from the aforementioned references and we performed all DF-MP2-F12 calculations using MOLPRO. For reactions **24** and **25**, where canonical DF-MP2-F12 proved unfeasible with available computational resources, we used localized-orbital PNO-LMP2-F12^67^ calculations with tightPNO settings (see the Supporting Information for details).

In post-SCF calculations, we correlated the metal’s valence *s* and *d* orbitals and froze an argon core for the 3*d*-block elements. For the 4*d*- and 5*d*-block elements with an ECP, we froze the four subvalence (*n* – 1)*sp* orbitals. The frozen core settings in this work are further detailed in Table S1 in the Supporting Information.

## 3 Results and Discussion

### 3.A Basis Set Optimization

To optimize the basis set exponents for the *d*-block elements, we employed Powell’s BOBYQA^68^ (Bound Optimization By Quadratic Approximation) derivative-free constrained optimizer in custom Fortran, Bash, and Python scripts. As a target function for the optimization, we minimized the MP2-F12 total energy (including CABS corrections^29^ to the HF component) averaged over (with *m* the number of valence electrons) *n*s^2^(*n* – 1)d^*m*–2^, *n*s^1^(*n* – 1)d^*m*–1^, and *n*s^0^(*n* – 1)d^*m*^ states. Following Dolg and co-workers,^69,70^ as *n*p orbitals typically are unoccupied in transition-metal complexes, we did not include any *n*p states in the averaging, unlike refs (46), (53), and (54). All weights in the averaging were set to unity.

Our optimization approach, inspired by the recent work of Shaw and Hill (SH)^25^ on correlation consistent basis sets for group 11 and 12 elements in explicitly correlated wave function theory, shares similarities, in terms of energy averaging of many atomic states, but differs in other aspects. The major difference lies in the treatment of the *spd* set. In SH’s V*n*Z-PP-F12, the *spd* primitives were obtained from the corresponding V(*n* + 1)Z-PP and contraction coefficients from state-averaged MRCI-F12^71^ atomic natural orbitals (ANOs).^72−75^ Also, single uncontracted *s*, *p*, and *d* diffuse functions were included from AV(*n* + 1)Z-PP. In the V*n*Z-F12^21^ basis sets for main-group elements, the *sp* part of the AV(*n* + 1)Z basis set was used as a foundation; analogously, for the first-row transition metals (TMs), we used the *spd* primitive and contracted functions from AV(*n* + 1)Z^46^ without any modifications as a foundation, while for second- and third-row TMs, the corresponding set of exponents from pseudopotential-based AV(*n* + 1)Z-PP were used,^53,54^ and subsequently we optimized exponents of higher angular momentum than *d*. By way of illustration, our VTZ-PP-F12-wis basis set for ruthenium includes primitive and contracted *s*, *p*, and *d* functions from AVQZ-PP, in addition to three *f* and two *g* basis functions that were uncontracted and simultaneously optimized. The primitive and contracted compositions of the final basis sets are shown in Table 1.

**Table 1:** Basis Set Functions of Optimized V*n*Z(-PP)-F12-wis Orbital Basis Sets

	VDZ-F12-wis	VTZ-F12-wis
Sc–Zn	(21*s*17*p*9*d*2*f*)/[8*s*7*p*5*d*2*f*]	(23*s*19*p*12*d*3*f*2*g*)/[9*s*8*p*6*d*3*f*2*g*]

	VDZ-PP-F12-wis^a^	VTZ-PP-F12-wis^a^
Y–Cd/Hf-Hg	(11*s*10*p*9*d*2*f*)/[6*s*6*p*5*d*2*f*]	(15*s*12*p*11*d*3*f*2*g*)/[7*s*7*p*6*d*3*f*2*g*]

a The basis set includes the incorporation of the effective core potentials ECP28MDF for the Y–Cd elements and ECP60MDF for the Hf–Hg elements.

The primitives from the AV(*n* + 1)Z basis contain a substantial number of *d* exponents, which is of paramount importance for energetically closely spaced states of transition metals. (Already in the 1970s, Hay pointed out the need to add adequate *d* functions to first-row transition metal atoms.^76^) For our study, we optimized the *f* exponents of VDZ(-PP)-F12-wis basis sets for the atoms Sc–Hg on top of a fixed (21*s*17*p*9*d*) primitive set contracted to [8*s*7*p*5*d*] for Sc–Zn, and (11*s*10*p*9*d*) contracted to [6*s*6*p*5*d*] for Y–Cd and Hf–Hg. The selected electronic states are listed in Table 2, while total energies can be found in Table S2 in the Supporting Information.

**Table 2:** Selected States of *d*-Block Elements for the Optimization of Orbital Basis Sets Exponents in State-Averaged MP2-F12 Calculations for the Lowest-Lying Electronic States with *ns*^2^(*n* – 1)*d*^*m*–2^, *ns*^1^(*n* – 1)d^*m*–1^, and *ns*^0^(*n* – 1)*d*^*m*^ Valence Shells^a^

	*m* (neutral)	*ns*^2^(*n* – 1)*d*^*m*–2^	*ns*^1^(*n* – 1)d^*m*–1^	*ns*^0^(*n* – 1)*d*^*m*^
Sc/Y	3	^2^*D*	^4^*F*	^4^*F*
Ti/Zr/Hf	4	^3^*F*	^5^*F*	^5^*D*
V/Nb/Ta	5	^4^*F*	^6^*D*	^6^*S*
Cr/Mo/W	6	^5^*D*	^7^*S*	^5^*D*
Mn/Tc/Re	7	^6^*S*	^6^*D*	^4^*F*
Fe/Ru/Os	8	^5^*D*	^5^*F*	^3^*F*
Co/Rh/Ir	9	^4^*F*	^4^*F*	^2^*D*
Ni/Pd/Pt	10	^3^*F*	^3^*D*	^1^*S*
Cu/Ag/Au	11	^2^*D*	^2^*S*	...
Zn/Cd/Hg	12	^1^*S*	...	...

a The *m* represents the number of valence electrons and *n* represents the principal number. Spin–orbit coupling is neglected throughout.

The *f* and *g* basis set exponents were optimized independently (in log *a* space) without constraints, while the *spd* set was left unaltered. We verified that, regardless of the initial values, the optimized exponents consistently converged to the same values. The resulting *fg* correlating functions can be found in Table S3, along with our V*n*Z(-PP)-F12-wis basis sets, available in MOLPRO format within the Supporting Information.

In our optimization procedure, we employed several auxiliary basis sets. For Coulomb-exchange fitting (JK), we utilized the def2-QZVPP/JKFit basis set^77^ for all *d*-block elements. Moreover, for the DF-MP2 step, we employed purpose-optimized DF-MP2 auxiliary basis sets that were available to us from the literature, including awCVTZ/MP2Fit^58^ for 3*d*-block elements and awCVTZ-PP/MP2Fit^61,63^ for 4*d* and 5*d*-block elements. As complementary auxiliary basis set (CABS), we used def2-QZVPP/JKFit^77^ for all *d*-block elements, which, even though it was not originally intended for explicitly correlated methods, we deemed suitable for this work, because of its large size. This choice was also based on our previous experimentation with different CABS choices and the finding in ref (17) that VQZ-F12/JKFit as CABS yielded an RMSD of <0.01 kcal/mol, relative to a reference-OptRI^19^ CABS for the W4–08 main-group thermochemistry benchmark,^78^ comparable to purpose-optimized CABSs.^79,80^

The selected high-spin states of the *d*-block elements appear to possess single-reference character, as evidenced by the multireference character diagnostics, *T*_1_,^81^*D*_1_,^82−84^ and *D*_2_,^82^ which are listed in Table S9 in the Supporting Information. The calculated *T*_1_ and *D*_1_ values are below the suggested thresholds of 0.05 and 0.12, respectively, as per the criteria established by Wilson and co-workers in ref (85), indicating the applicability of single reference for the objectives of this study.

**Figure 1:**
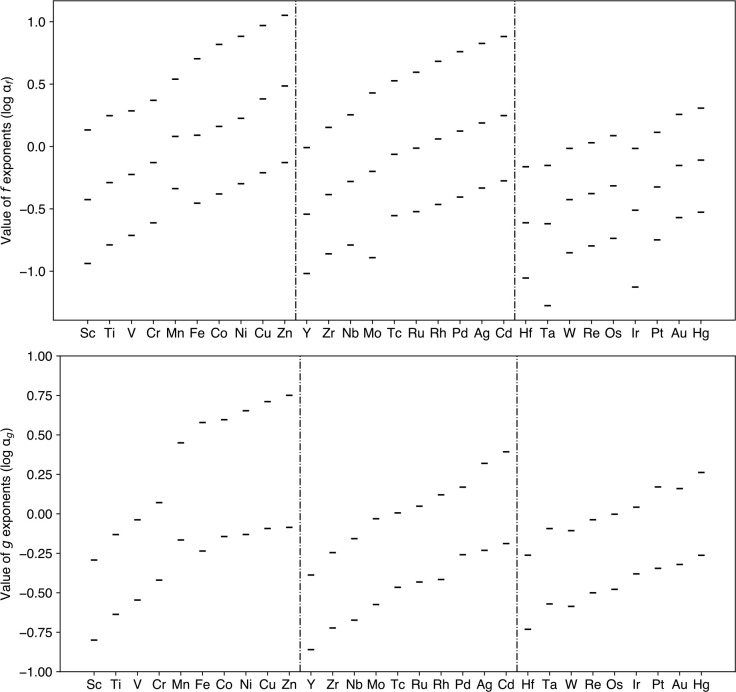
Optimized *f* and *g* exponents on a logarithmic scale for atoms Sc–Zn, Y–Cd, and Hf–Hg using the VTZ(-PP)-F12-wis basis sets.

The optimized *f* and *g* exponents for the *d*-block elements from the VTZ(-PP)-F12-wis basis set are shown in Figure 1, while the optimized *f* exponents from VDZ(-PP)-F12-wis can be found in Figure S1 in the Supporting Information. Displayed on a logarithmic scale, the *f* exponents show a consistent increase from Sc to Zn, from Y to Cd, and from Hf to Hg. As expected, the values become generally smaller upon descending the periodic table, particularly in the 5*d*-block elements. The *g* exponents exhibit a similar trend, with log *a*_g_ values lying in a narrower range between −1 and 0.25 for the 4*d* and 5*d*-block elements. However, a marked increase in these values can be seen in the 3*d*-block elements.

**Figure 2:**
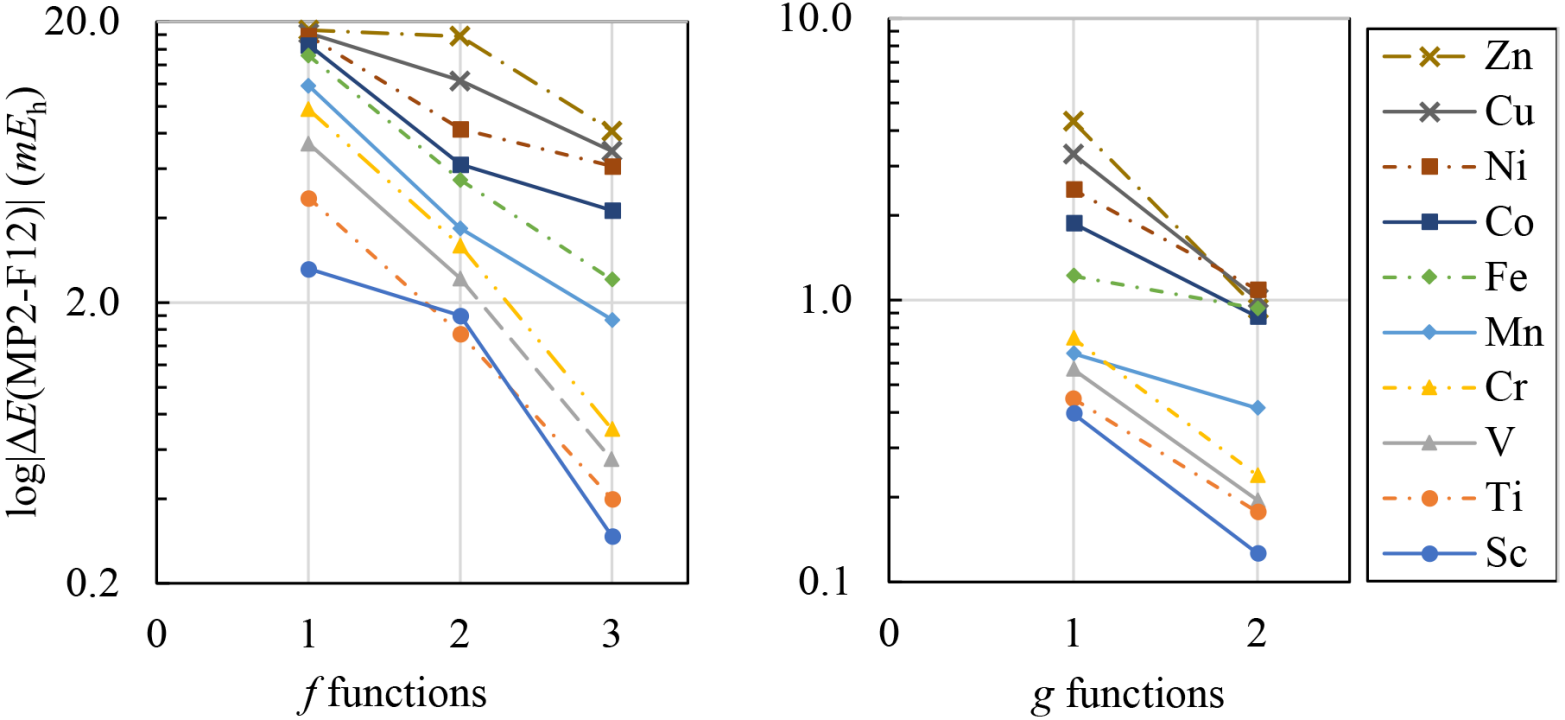
Incremental contribution of the *f* and *g* optimized exponents of cc-pVTZ-F12-wis to the state-averaged MP2-F12 correlation energy for the 3*d*-block elements.

Next, we employed a simple stepwise process to determine the incremental contribution of each additional exponent to the correlation energy. Initially, a single *f* exponent was optimized, followed by a pair of *f* exponents and finally all three *f* exponents for the VTZ(-PP)-F12-wis basis. Then, we optimized the *f* exponents in the presence of a single *g* exponent and repeated this process for the two *g* exponents, optimizing the *f* and *g* exponents simultaneously. The results, as illustrated in Figure 2 for 3*d*-block elements, reveal that the MP2-F12 correlation energy converges quite rapidly with an increasing number of exponents.

It is not surprising that, as the number of *d*-electrons increases, the contribution of each additional exponent becomes more significant. For example, the addition of a single *f* exponent for scandium lowers its correlation energy by 2.640 m*E*_h_, while the second *f* function adds another 1.794 m*E*_h_ and the third *f* just adds a measly 0.294 m*E*_h_. For the atom of copper, the corresponding values are 18.235, 12.350, and 6.920 m*E*_h_, respectively. As expected, similar conclusions can be drawn for the 4*d* and 5*d*-block elements. Table 3 shows the total energies of platinum group metals, while Table S2 in the Supporting Information lists energies for all *d*-block elements.

**Table 3:** Total Energies (E_h_) for Neutral Atom States of the Platinum Group of Metals Using VTZ-PP-F12-wis Basis Sets

			Total Energies (E_h_)	
state		M	MP2-F12	state-averaged MP2-F12
*s*^2^*d*^6^	^5^*D*	Ru	–93.994178	–94.003754
*s*^1^*d*^7^	^5^*F*		–94.023412	
*d*^8^	^3^*F*		–93.993672	
*s*^2^*d*^7^	^4^*F*	Rh	–109.614505	–109.658656
*s*^1^*d*^8^	^4^*F*		–109.682221	
*d*^9^	^2^*D*		–109.679242	
*s*^2^*d*^8^	^3^*F*	Pd	–126.884340	–126.975003
*s*^1^*d*^9^	^3^*D*		–126.993669	
*d*^10^	^1^*S*		–127.047000	
*s*^2^*d*^6^	^5^*D*	Os	–90.276508	–90.232484
*s*^1^*d*^7^	^5^*F*		–90.250099	
*d*^8^	^3^*F*		–90.170846	
*s*^2^*d*^7^	^4^*F*	Ir	–103.899644	–103.643703
*s*^1^*d*^8^	^4^*F*		–103.902200	
*d*^9^	^2^*D*		–103.129265	
*s*^2^*d*^8^	^3^*F*	Pt	–118.850224	–118.872462
*s*^1^*d*^9^	^3^*D*		–118.884844	
*d*^10^	^1^*S*		–118.882318	

Moreover, the contribution of *g* functions to the correlation energies is clearly less significant. For instance, the first two *g-* exponents on scandium add just 0.396 and 0.126 m*E*_h_, respectively. However, these contributions become more noticeable for the late transition metals.

On another note, the addition of two *f* exponents yielded contributions to the correlation energy that were comparable between VDZ(-PP)-F12-wis and VTZ(-PP)-F12-wis basis sets for most elements. For instance, the addition of a single *f* exponent to a titanium atom lowered the correlation energy by 4.73 m*E*_h_ in both VDZ-F12-wis or VTZ-F12-wis sets, and a second *f* exponent resulted in a lowering of 1.5_5_ m*E*_h_ in each set.

As noted by a reviewer, prior studies in refs^46^, (53), and (54) focused on optimizing *p*-type primitives for the lowest *s*^2^*d*^*m*–3^*p*^1^ atomic state of each transition-metal element. As the present discussion is centered around optimizing exponents of higher angular momentum than *d*, we pose a different question: Can the inclusion of an extra state into the energy averaging affect the optimized exponents and concomitant energies? To address this, we reoptimized the two *f* exponents in the VDZ-F12-wis basis set for Ni, Cu, and Zn while including this extra state in the MP2-F12 energy averaging: that amounts to ^3^*F*, ^3^*D*, ^1^*S*, and ^5^*F* for Ni; ^2^*D*, ^2^*S*, and ^4^*D* for Cu; ^1^*S* and ^3^*P* for Zn. The resulting new *f* exponents increase by 4.0% and 0.9% for Ni, by 4.1% and 0.4% for Cu, and by 6.4% and 1.3% for Zn. While these relative changes seem small, could they materially impact the atomic energies? As can be seen in Table 4, effects on individual state energies are on the order of 0.1 m*E*_h_ or less, which is negligible for a basis set as small as VDZ-F12-wis. The decision to exclude this *s*^2^*d*^*m*–3^*p*^1^ state from the optimization for the other elements would hence seem to be justified, especially as the types of transition-metal complexes most of interest to organometallic chemists are unlikely to have any significant (*n*)*p* involvement on the metal.

**Table 4:** Total Energies of Neutral Atom States Using Reoptimized and Standard VDZ-F12-wis Basis Sets

	Total Energies (E_h_)		VDZ-F12-wis (reopt.)^a^	VDZ-F12-wis	
state		M	MP2-F12 (E_h_)	MP2-F12 (E_h_)	Δ*E*_corr,MP2-F12_ (μE_h_)
*s*^2^*d*^8^	^3^*F*	Ni	–1507.231005	–1507.230924	–63.6
*s*^1^*d*^9^	^3^*D*		–1507.281095	–1507.281110	24.3
*d*^10^	^1^*S*		–1507.282402	–1507.282540	137.7
*s*^2^*d*^7^*p*^1^	^5^*F*		–1506.930006	–1506.929846	–137.0
*s*^2^*d*^9^	^2^*D*	Cu	–1639.527189	–1639.527281	92.3
*s*^1^*d*^10^	^2^*S*		–1639.424003	–1639.423992	4.6
*s*^2^*d*^8^*p*^1^	^4^*D*		–1639.044033	–1639.043906	–98.1
*s*^2^*d*^10^	^1^*S*	Zn	–1778.423522	–1778.423576	53.9
*s*^2^*d*^9^*p*^1^	^3^*P*		–1777.971401	–1777.971248	–132.2

a *f*_1_ and *f*_2_ exponents of the VDZ-F12-wis basis set were reoptimized for the total state-averaged MP2-F12 energy over all states, including the *s*^2^*d*^*m*–3^*p*^1^ state.

SH’s VnZ-PP-F12 orbital basis sets^25^ and our V*n*Z(-PP)-F12 appear to be functionally equivalent for heavier group 11 and 12 atoms. The resulting atomic energies, calculated using MP2-F12 with either SH’s or our basis sets, are given in Table 5. Evidently, these differences are on the order of a few tens of μ*E*_h_ across different electronic states for silver, cadmium, gold, and mercury atoms, all of which have an ECP for the core electrons. We remind the reader that SH’s V*n*Z-PP-F12 basis sets and our basis sets differ in their treatment of the 3*d*-block elements; we freeze an argon core for all 3*d*-block elements, while Shaw and Hill replaced the 1*s*2*s*2*p* electrons by a 10 core–electron effective core potential (ECP10MDF) for copper and zinc atoms.

**Table 5:** Total Energies of Neutral Atom States Obtained from Our V*n*Z(-PP)-F12 Basis Sets (*n* = D,T) and Shaw and Hill’s (SH’s) V*n*Z-PP-F12 Basis Sets^a^

	Basis		VDZ(-PP)-F12			VTZ(-PP)-F12		
state		M	MP2-F12 (E_h_) (this work)	MP2-F12 (E_h_) (SH’s)^b^	Δ*E*_tot,MP2-F12_ (μE_h_), this work – SH’s	MP2-F12 (E_h_) (this work)	MP2-F12 (E_h_) (SH’s)^b^	Δ*E*_tot,MP2-F12_ (μE_h_), this work – SH’s
*s*^2^*d*^9^	^2^*D*	Cu	–1639.423992	–196.662732	N/A	–1639.436376	–196.675577	N/A
*s*^1^*d*^10^	^2^*S*		–1639.527281	–196.750960	N/A	–1639.540292	–196.764317	N/A
*s*^2^*d*^10^	^1^*S*	Zn	–1778.423576	–226.553524	N/A	–1778.438409	–226.568832	N/A
*s*^2^*d*^9^	^2^*D*	Ag	–146. 349754	–146.349725	–28.1 (−28.2)	–146.366916	–146.366899	–17.1 (−16.2)
*s*^1^*d*^10^	^2^*S*		–146.511753	–146.511731	–21.6 (22.5)	–146.529112	–146.529086	–25.5 (25.2)
*s*^2^*d*^10^	^1^*S*	Cd	–167.362871	–167.362808	–62.9 (−62.5)	–167.382481	–167.382460	–20.8 (−20.6)
*s*^2^*d*^9^	^2^*D*	Au	–135.144459	–135.144435	–23.8 (−24.2)	–135.155261	–135.155230	–31.2 (−30.9)
*s*^1^*d*^10^	^2^*S*		–135.221282	–135.221283	1.1 (−0.1)	–135.231641	–135.231610	–30.9 (−30.8)
*s*^2^*d*^10^	^1^*S*	Hg	–153.054562	–153.054526	–36.4 (−36.8)	–153.065060	–153.065025	–34.7 (−34.7)

a Values in parentheses indicate differences in the MP2-F12 correlation energies.

b In SH’s V*n*Z-PP-F12 basis sets, an ECP is used for the core electrons of copper and zinc atoms.

Regarding the differences in the V*n*Z-PP-F12 basis set exponents, they are minimal overall for the *f* and *g* exponents. The mean absolute deviation (MAD) between SH’s and our VDZ(-PP)-F12 is merely 0.001 for all optimized *f* exponents of Ag, Cd, Au, and Hg. For *n* = T, we obtain a corresponding MAD value of 0.016 for all optimized *f* and *g* exponents (see Table S7 in the Supporting Information), which is still insignificant in basis set terms.

Can we consider an additional comparison of the *spd* values in the underlying F12 orbital basis sets? When we employed truncated F12 basis sets limited to *spd* functions, we found minor differences in the calculated atomic MP2-F12 energies between our VnZ-PP-F12(*d*) compared to SH’s VnZ-PP-F12(*d*) (see Table S8 in the Supporting Information). For *n* = D, the correlation energies vary from −7.0 to −50.7 μ*E*_h_, while for *n* = T, the range spans only −2.1 to −13.8 μ*E*_h_. Therefore, our current choice of the *spd* set does not compromise the accuracy of our F12 basis sets.

In response to a referee comment, we reoptimized our VDZ(-PP)-F12 exponents in the presence on an F12 geminal with β = 1.0, rather than 1.4 as used presently and in SH’s work. The results are given in Table S14 in the SI, represented as common logarithms of the exponents. Typically, exponents with β = 1.0 are somewhat smaller than those with β = 1.4. The exceptions are Cu and Zn, which are best understood by comparison with 3*f* optimized exponents. The solutions for 2*f* are then effectively *a*_1_, *a*_3_ for β = 1.4, and *a*_2_, *a*_3_ for β = 1.0.

### 3.2 Molecular Calculations with V*n*Z(-PP)-F12-wis Basis Sets

Having presented our basis sets, we will now assess their performance compared with estimated complete basis set (CBS) limits for problems of practical interest.

#### 3.2.1 The MOBH35 Metal–Organic Barrier Heights Benchmark

This benchmark from Iron and Janes^33,34^ consists of forward and reverse barrier heights of 35 catalytic reactions taken from real-life experimental organometallic literature. The original reference data were obtained by means of the domain localized pair natural orbital coupled cluster approach extrapolated to the complete basis set limit, DLPNO–CCSD(T_1_)/CBS; in ref (86), we revised reference values for part of the systems through canonical CCSD(T) and used this to assess the performance of different localized natural orbitals, including DLPNO–CCSD(T_1_),^87−91^ PNO-LCCSD(T),^92−96^ and LNO–CCSD(T).^97−101^ All of these performed well in the low-static-correlation regime, but LNO–CCSD(T) proved to be more resilient as static correlation became moderately strong. Additionally, the MOBH35 dataset serves as a benchmarking tool among researchers. Recently, Feldt and colleagues considered 13 reactions from the MOBH35 dataset to investigate “local[ized] molecular orbital: molecular orbital” (LMOMO) schemes and projection-based wave function-in-DFT embedding.^102^ Moreover, Kaupp and co-workers investigated the performance of the LH20t-D4 and ωLH22t range separated hybrids on the smaller MOBH28 set.^103^

For the purposes of the present work, we are interested primarily in basis set convergence, and the performance of MP2-F12 compared to MP2/CBS will be a good proxy for basis set sufficiency at the CCSD(T)-F12/CBS level. Hence, we have evaluated the energetics of the MOBH35 set using DF-MP2-F12. To obtain accurate reference energies, we extrapolated the HF and MP2 parts separately to reach the CBS limit for DF-MP2 by using the VQZ(-PP) and V5Z(-PP) basis sets. For the smaller reactions 15, 16, and 32–35, we obtained MP2/AV{Q,5}Z-PP quality reference energies (see the Supporting Information). Also, the def2-QZVPP/JKFit and the appropriate V*n*Z(-PP)/MP2Fit basis sets were used as MP2Fit. These calculations were carried out using MRCC, as MOLPRO is unable to accommodate the higher angular momentum functions, in this case *k* and *l*, required for the DF-MP2 ABSs for these basis sets. (As we have occupied *d* orbitals at hand, they require the highest angular momentum in the orbital basis set *plus two*.) The energy differences with and without these additional functions can be found in Table S4 in the Supporting Information; the RMS error caused by their omission (e.g., because of program limitations) is just 0.05_1_ kcal/mol. For perspective, as a rough estimate of the level of uncertainty in the reference data, we calculated the RMS difference between the V{T,Q}Z(-PP) and V{Q,5}Z(-PP) extrapolations at the DF-MP2 level and found it to be 0.17_6_ kcal/mol.

The best choice for the geminal exponent β in DF-MP2-F12 on MOBH35 is at first sight debatable. On the one hand, the “action” takes place at the metal center, which would militate in favor of β = 1.4 as used presently in the optimizations and in Shaw and Hill’s prior work.^25^ On the other hand, for each metal atom, there are dozens of main-group atoms for which the standard cc-pVDZ-F12 and cc-pVTZ-F12 basis sets come with recommended β values of 0.9 and 1.0, respectively. The only practical way to resolve this conundrum is by testing both.

Tables 6 and 7 show the error statistics for the MP2-F12 energetics of the MOBH35. Using either β = 1.0 or 1.4 leads to identical MAD values for VTZ(-PP)-F12-wis OBS for the *d*-block elements and VTZ-F12 OBS for main group elements, resulting in our most accurate RMSD of 0.09 kcal/mol for Δ*E*_all_, inclusive of all forward and reverse barrier heights, as well as reaction energies (Table 7). This accuracy can be attributed to the adequate angular and radial flexibility of AVQZ(-PP). An error breakdown analysis for VTZ(-PP)-F12-wis with β = 1.0 reveals low RMSDs for reactions of first-row transition-metal complexes (0.07 kcal/mol) and 4*d* and 5*d*-block complexes (0.11 and 0.09 kcal/mol, respectively).

**Table 6:** Root-Mean-Square-Deviations of DF-MP2-F12 with VDZ(-PP)-F12-wis or AVTZ(-PP) Basis Sets for *d*-Block Elements Relative to MP2/V{Q,5}Z(-PP) for the MOBH35 Dataset^a^

					Root-Mean-Square-Deviations, RMSDs (kcal/mol)			
β geminal exponent	Basis	3*d-*block element	4*d*/5*d-*block element	M	Δ*E*_all_	Δ*E*_fwd_^#^	Δ*E*_rev_^#^	Δ*E*_reac_
0.9	OBS	AVTZ^b^	AVTZ-PP^b^	all	0.20	0.20	0.15	0.24
	JKFit	def2-QZVPP/JKFit		3*d*	0.16	0.15	0.11	0.20
	CABS	def2-QZVPP/JKFit		4*d*	0.28	0.28	0.24	0.32
	MP2Fit	AWCVTZ/MP2Fit^c^	AWCVTZ-PP/MP2Fit^c^	5d	0.13	0.14	0.07	0.17
								
1.4	OBS	AVTZ^b^	AVTZ-PP^b^	all	0.42	0.36	0.27	0.57
	JKFit	def2-QZVPP/JKFit		3*d*	0.25	0.23	0.17	0.34
	CABS	def2-QZVPP/JKFit		4*d*	0.69	0.58	0.43	0.95
	MP2Fit	AWCVTZ/MP2Fit^c^	AWCVTZ-PP/MP2Fit^c^	5*d*	0.17	0.17	0.16	0.19
								
0.9	OBS	AVTZ(*f*)^d^	AVTZ-PP(*f*)^d^	all	0.26	0.30	0.20	0.26
	JKFit	def2-QZVPP/JKFit		3*d*	0.18	0.14	0.14	0.24
	CABS	def2-QZVPP/JKFit		4*d*	0.39	0.48	0.31	0.35
	MP2Fit	AWCVTZ/MP2Fit^c^	AWCVTZ-PP/MP2Fit^c^	5*d*	0.15	0.14	0.10	0.18
								
1.4	OBS	AVTZ(*f*)^d^	AVTZ-PP(*f*)^d^	all	0.39	0.31	0.34	0.50
	JKFit	def2-QZVPP/JKFit		3*d*	0.27	0.23	0.20	0.36
	CABS	def2-QZVPP/JKFit		4*d*	0.59	0.47	0.50	0.75
	MP2Fit	AWCVTZ/MP2Fit^c^	AWCVTZ-PP/MP2Fit^c^	5*d*	0.26	0.19	0.27	0.30
								
0.9	OBS	AVTZ(*f*-1)^e^	AVTZ-PP(*f*-1)^e^	all	0.31	0.36	0.33	0.25
	JKFit	def2-QZVPP/JKFit		3*d*	0.17	0.16	0.12	0.22
	CABS	def2-QZVPP/JKFit		4*d*	0.26	0.60	0.56	0.35
	MP2Fit	AWCVTZ/MP2Fit^c^	AWCVTZ-PP/MP2Fit^c^	5*d*	0.14	0.14	0.11	0.16
								
1.4	OBS	AVTZ(*f* – 1)^e^	AVTZ-PP(*f* – 1)^e^	all	0.55	0.34	0.62	0.63
	JKFit	def2-QZVPP/JKFit		3*d*	0.30	0.22	0.25	0.40
	CABS	def2-QZVPP/JKFit		4*d*	0.88	0.52	1.03	1.00
	MP2Fit	AWCVTZ/MP2Fit^c^	AWCVTZ-PP/MP2Fit^c^	5*d*	0.27	0.20	0.29	0.30
								
0.9	OBS	**VDZ-F12-wis**	**VDZ-PP-F12-wis**^f^	all	0.29	0.33	0.29	0.25
	JKFit	def2-QZVPP/JKFit		3*d*	0.20	0.17	0.14	0.26
	CABS	def2-QZVPP/JKFit		4*d*	0.46	0.54	0.48	0.34
	MP2Fit	AWCVTZ/MP2Fit^c^	AWCVTZ-PP/MP2Fit^c^	5*d*	0.13	0.13	0.13	0.13
								
1.4	OBS	**VDZ-F12-wis**	**VDZ-PP-F12-wis**^f^	all	0.51	0.34	0.54	0.61
	JKFit	def2-QZVPP/JKFit		3*d*	0.28	0.23	0.21	0.37
	CABS	def2-QZVPP/JKFit		4*d*	0.81	0.51	0.88	0.96
	MP2Fit	AWCVTZ/MP2Fit^c^	AWCVTZ-PP/MP2Fit^c^	5*d*	0.27	0.21	0.28	0.31

a Each block represents a set of calculations performed with the orbital and auxiliary basis sets shown below for the *d-*block elements. VDZ-F12 was used for OBS, def2-QZVPP/JKFit for JKFit and CABS, and VDZ-F12/MP2Fit for MP2Fit in main group elements in all blocks.

b AVTZ as OBS for 3*d*-block elements contracted to [8*s*7*p*5*d*3*f*2*g*], and AVTZ-PP for 4*d* and 5*d*-block elements contracted to [6*s*6*p*5*d*3*f*2*g*].

c awCVTZ/MP2Fit with contraction [15*s*14*p*12*d*11*f*9*g*6*h*4*i*] for 3*d*-block elements, awCVTZ-PP/MP2Fit contracted to [12*s*12*p*11*d*10*f*9*g*6*h*4*i*] and [13*s*13*p*12*d*10*f*8*g*6*h*4*i*] for 4*d* and 5*d*-block elements, respectively.

d Truncated AVTZ(*f*) contracted to [8*s*7*p*5*d*3*f*] for 3*d*-block elements; AVTZ-PP(*f*) with contraction to [6*s*6*p*5*d*3*f*] for 4*d* and 5*d*-block elements, and *g* exponents omitted.

e Truncated AVTZ(*f*-1), as in case (d), but a single diffuse *f* exponent is omitted, resulting in contraction to [8*s*7*p*5*d*2*f*] for 3*d-*block elements, and [6*s*6*p*5*d*2*f*] for 4*d-* and 5*d*-block elements.

f Our proposed VDZ(-PP)-F12-wis basis sets, which have the same contraction to case (e).

**Table 7:** Root-Mean-Square-Deviations of DF-MP2-F12 with VTZ(-PP)-F12-wis or AVQZ(-PP) Basis Sets for *d*-Block Elements Relative to MP2/V{Q,5}Z(-PP) for the MOBH35 Dataset^a^

					Root-Mean-Square-Deviations, RMSDs (kcal/mol)			
β geminal exponent	basis	3*d-*block element	4*d*/5*d-*block element	M	Δ*E*_all_	Δ*E*_fwd_^#^	Δ*E*_rev_^#^	Δ*E*_reac_
1.4	OBS	AVQZ^b^	AVQZ-PP^b^	all	0.09	0.06	0.07	0.10
	JKFit	def2-QZVPP/JKFit		3*d*	0.08	0.07	0.08	0.09
	CABS	def2-QZVPP/JKFit		4*d*	0.12	0.06	0.07	0.09
	MP2Fit	AVQZ(i)/MP2Fit^c^	AVQZ-PP(i)/MP2Fit^c^	5*d*	0.08	0.06	0.07	0.11
								
1.4	OBS	AVQZ(*g*)^d^	AVQZ-PP(*g*)^d^	all	0.08	0.06	0.07	0.08
	JKFit	def2-QZVPP/JKFit		3*d*	0.09	0.07	0.10	0.10
	CABS	def2-QZVPP/JKFit		4*d*	0.12	0.06	0.07	0.08
	MP2Fit	AVQZ(i)/MP2Fit^c^	AVQZ-PP(i)/MP2Fit^c^	5*d*	0.06	0.06	0.05	0.06
								
1.4	OBS	AVQZ(*g*-1)^e^	AVQZ-PP(*g*-1)^e^	all	0.09	0.07	0.07	0.09
	JKFit	def2-QZVPP/JKFit		3*d*	0.10	0.07	0.10	0.11
	CABS	def2-QZVPP/JKFit		4*d*	0.13	0.06	0.07	0.09
	MP2Fit	AVQZ(i)/MP2Fit^c^	AVQZ-PP(i)/MP2Fit^c^	5*d*	0.06	0.07	0.09	0.06
								
1.4	OBS	**VTZ-F12-wis**^f^	**VTZ-PP-F12-wis**^f^	all	0.09	0.06	0.08	0.09
	JKFit	def2-QZVPP/JKFit		3*d*	0.09	0.07	0.10	0.10
	CABS	def2-QZVPP/JKFit		4*d*	0.13	0.06	0.08	0.09
	MP2Fit	AVQZ(i)/MP2Fit^c^	AVQZ-PP(i)/MP2Fit^c^	5*d*	0.06	0.07	0.06	0.06
								
1.0	OBS	**VTZ-F12-wis**^f^	**VTZ-PP-F12-wis**^f^	all	0.09	0.06	0.07	0.10
	JKFit	def2-QZVPP/JKFit		3*d*	0.07	0.05	0.08	0.09
	CABS	def2-QZVPP/JKFit		4*d*	0.11	0.05	0.06	0.08
	MP2Fit	AVQZ(i)/MP2Fit^c^	AVQZ-PP(i)/MP2Fit^c^	5*d*	0.09	0.08	0.07	0.12

a Reactions 17–20 are omitted due to computational constraints. VTZ-F12 is used as the OBS, def2-QZVPP/JKFit for JKFit and CABS, and VTZ-F12/MP2Fit in main group elements throughout.

b AVQZ as OBS for 3*d*-block elements contracted to [9*s*8*p*6*d*4*f*3*g*2*h*], and AVQZ-PP for 4*d* and 5*d*-block elements contracted to [7*s*7*p*6*d*4*f*3*g*2*h*].

c Truncated AVQZ(i)/MP2Fit for 3*d*-block elements, and AVQZ-PP(i)/MP2Fit for 4*d* and 5*d*-block elements, with *k* functions excluded throughout.

d Truncated AVQZ(*g*) contracted to [9*s*8*p*6*d*4*f*3*g*] for 3*d*-block elements; AVQZ-PP(*g*) contracted to [7*s*7*p*6*d*4*f*3*g*] for both 4*d*- and 5*d*-block elements.

e A variation of (d) but contracted to [9*s*8*p*6*d*3*f*2*g*] for 3*d*-block elements, and with contraction to [7*s*7*p*6*d*3*f*2*g*] for 4*d* and 5*d*-block elements (one diffuse *f* and one diffuse *g* exponent are omitted).

f Our proposed VTZ(-PP)-F12-wis basis sets which have the same contraction as case (e).

The effect of the β geminal exponent is more pronounced for smaller orbital basis sets. This is evidenced by the RMSDs for Δ*E*_all_, which increase from 0.20 to 0.42 kcal/mol for β = 0.9 and 1.4, respectively, when using smaller AVTZ(-PP) OBS for transition metals and VDZ-F12 OBS for main group elements. The RMSDs for β = 1.4 become over two times as large as for β = 0.9 for 3*d*- and 4*d*-block metal complexes and ∼1.5 times as large for 5*d*-block complexes. Auxiliary basis sets are specified in Tables 6 and 7.

We shall now assess the accuracy of our V*n*Z(-PP)-F12-wis basis sets in comparison to the standard AV(*n* + 1)Z(-PP) and their truncated variants using the same auxiliary basis sets to ensure a fair evaluation. Specifically, we employ the def2-QZVPP/JKFit for JKFit and CABS, and the awCVTZ(-PP) for DF-MP2 ABS.

The advantage of AVTZ OBS over VDZ(-PP)-F12-wis is consistent at 0.09 kcal/mol for Δ*E*_all_ for both β values, 0.9 and 1.4. To investigate the effect of removing the higher angular momentum exponents, specifically the pair of *g* exponents in AVTZ(-PP), (cf. Papajak and Truhlar’s work^104^ on “calendar basis sets”), the resulting AVTZ(-PP)(*f*) set was examined. It is evident that this set performs the same as AVTZ(-PP), and therefore, AVTZ(-PP)(*f*) is better than VDZ(-PP)-F12 by only 0.03 and 0.12 kcal/mol for β = 0.9 and 1.4, respectively. However, elimination of the additional diffuse *f* exponent in the AVTZ(-PP)(*f* – 1) basis set, which now has the same number of exponents as VDZ(-PP)-F12-wis, degraded the RMSD for all reactions to 0.31 and 0.55 kcal/mol for the two respective β values.

**Figure 3:**
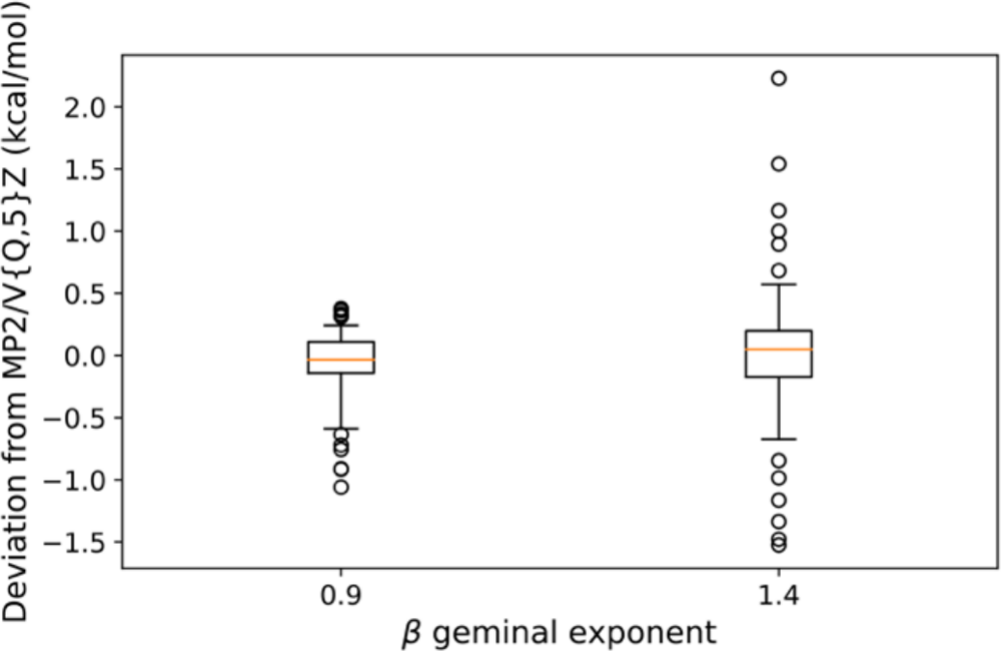
Box plot of energy deviations in DF-MP2-F12 with VDZ(-PP)-F12-wis orbital basis sets and two β geminal exponents (0.9 and 1.4), compared to DF-MP2/V{Q,5}Z reference in the MOBH35 data set.

Using β = 0.9 yields an RMSD of 0.29 kcal/mol from MP2/V{Q,5}Z for all reactions in MOBH35, slightly more than *half* the RMSD = 0.51 kcal/mol obtained for β = 1.4. This is perhaps made even clearer by the box plot in Figure 3 (see Table S5 in the Supporting Information for the deviations). Instead of following the usual practice of calculating the interquartile range (IQR) and extending the whiskers from Q_bottom_ – 1.5IQR to Q_top_ + 1.5IQR, the depicted box plot uses the 5th and 95th percentiles to determine the lengths of the whiskers. The deviations for β = 0.9 are concentrated within a narrow range of −1.0 to 0.5 kcal/mol, while those for β = 1.4 are more widely spread, with outliers ranging from −1.5 to 2.2 kcal/mol. Reactions 17–20 showed the most significant deviations in both sets of calculations.

Comparing our largest VTZ(-PP)-F12-wis orbital basis set for *d*-block elements against the standard AVQZ(-PP) basis set and its truncated variants, we find that the MP2-F12 error statistics improve with an RMSD of 0.09 kcal/mol for Δ*E*_all_, regardless of the choice of the OBS (Table 7). Even when we truncated the AVQZ(-PP) basis set to match the contraction pattern of our VTZ(-PP)-F12-wis (i.e., [9s8p6d3f2g] for 3*d*-block elements and [7s7p6d3f2g] for 4*d* and 5*d*-block elements), it did not affect the statistics. Therefore, our VTZ(-PP)-F12-wis is just as accurate as the largest standard AVQZ(-PP) statistically but with a more compact basis set structure and fewer exponents. Both β geminal exponents, 1.0 and 1.4, tested for VTZ(-PP)-F12-wis lead to identical error statistics. The RMSD for all reactions in the MOBH35 dataset, except for reactions 17–20, is 0.09 kcal/mol, implying that the β geminal exponent’s importance decreases when performing DF-MP2-F12 calculations on transition-metal complexes with large orbital basis sets that are saturated up to *g* and *h* functions.

The largest deviations of DF-MP2-F12 from MP2/V{Q,5}Z mainly occur for reactions involving 4*d*-block complexes and particularly for reactions 17–20, which are the only bimolecular reactions in the set, involving palladium complexes with two phosphines, trans-4-styrylpyride, and either bromine or iodine. Bond distances between palladium and bromine atoms in reactions 17 and 19 are up to 3.9_8_ Å, and those between palladium and iodine in reactions 18 and 20 are 4.16 Å. Using β = 0.9 for all orbital basis sets significantly improved the errors for reactions 17–20. For VDZ(-PP)-F12-wis with β = 0.9, the errors vary from −1.06 to 0.15 kcal/mol compared to errors ranging from −1.52 to 2.23 kcal/mol for β = 1.4. With the larger AVTZ-PP basis set, these errors lie in a smaller range from −0.57 to 0.36 kcal/mol with β = 0.9, and from −0.91 up to 2.26 kcal/mol with β = 1.4.

We assessed the effectiveness of ABSs in PNO-LMP2-F12^67^ calculations with tight domains approximations (DOMOPT = TIGHT). To that end, we generated ABSs from our V*n*Z(-PP)-F12-wis orbital sets for *d*-block elements using ORCA’s autoAux utility.^105^ The contractions of these V*n*Z(-PP)-F12-wis/autoAux ABSs are listed in Table 8. The RMSDs revealed a slight degradation for β = 1.4 in Δ*E*_all_ of 0.12 and 0.03 kcal/mol for *n* = D and T, respectively, compared to the errors previously obtained with def2-QZVPP/JKFit as JKFit and awCVTZ(-PP)/MP2Fit or AVQZ(-PP)(*i*)/MP2Fit ABSs as MP2Fit. Nonetheless, we conclude that these automatically generated ABSs chosen for JKFit and MP2Fit are robust and effectively serve their intended purpose.

**Table 8:** Root-Mean-Square-Deviations for PNO-LMP2-F12/V*n*Z(-PP)-F12-wis and MP2/V{Q,5}Z(-PP) for the MOBH35 Dataset, Where Reactions 17–20 Are Excluded from the Values Listed Below

						Root-Mean-Square-Deviations, RMSDs (kcal/mol)			
*β* geminal exponent	basis	main group element	3*d*-block element	4*d*/5*d*-block element	M	Δ*E*_all_	Δ*E*_fwd_^#^	Δ*E*_rev_^#^	Δ*E*_reac_
0.9	OBS	VDZ-F12	VDZ-F12-wis	VDZ-PP-F12-wis	all	0.49	0.36	0.36	0.50
	JKFit	def2-QZVPP/JKFit	VDZ(-PP)-F12-wis/AutoAux^a^		3*d*	0.61	0.35	0.60	0.80
	CABS	def2-QZVPP/JKFit			4*d*	0.52	0.38	0.26	0.30
	MP2Fit	VDZ-F12/MP2Fit	VDZ(-PP)-F12-wis/AutoAux^a^		5*d*	0.37	0.34	0.26	0.48
									
1.4	OBS	VDZ-F12	VDZ-F12-wis	VDZ-PP-F12-wis	all	0.39	0.26	0.35	0.38
	JKFit	def2-QZVPP/JKFit	VDZ(-PP)-F12-wis/AutoAux^a^		3*d*	0.24	0.18	0.21	0.32
	CABS	def2-QZVPP/JKFit			4*d*	0.53	0.26	0.36	0.34
	MP2Fit	VDZ-F12/MP2Fit	VDZ(-PP)-F12-wis/AutoAux^a^		5*d*	0.39	0.30	0.39	0.46
									
1.0	OBS	VTZ-F12	VTZ-F12-wis	VTZ-PP-F12-wis	all	0.19	0.17	0.14	0.17
	JKFit	VTZ(-PP)-F12-wis/AutoAux^b^			3*d*	0.13	0.11	0.15	0.13
	CABS	def2-QZVPP/JKFit			4*d*	0.25	0.19	0.13	0.13
	MP2Fit	VTZ-F12/MP2Fit	VTZ(-PP)-F12-wis/AutoAux^b^		5*d*	0.18	0.17	0.15	0.23
									
1.4	OBS	VTZ-F12	VTZ-F12-wis	VTZ-PP-F12-wis	all	0.12	0.10	0.10	0.11
	JKFit	VTZ(-PP)-F12-wis/AutoAux^b^			3*d*	0.08	0.05	0.09	0.08
	CABS	def2-QZVPP/JKFit			4*d*	0.17	0.10	0.10	0.12
	MP2Fit	VTZ-F12/MP2Fit	VTZ(-PP)-F12-wis/AutoAux^b^		5*d*	0.12	0.12	0.11	0.12

a Generated from VDZ(-PP)-F12-wis using ORCA’s autoAux;^105^ VDZ(-PP)-F12-wis/AutoAux is contracted as follows: [22*s*19*p*18*d*17*f*14*g*4*h*] for 3*d*-block elements, and [14*s*12*p*12*d*11*f*10*g*4*h*] and [14*s*12*p*11*d*10*f*10*g*4*h*] for 4*d* and 5*d*-block elements, respectively.

b Generated from VTZ(-PP)-F12-wis using ORCA’s autoAux; VTZ(-PP)-F12-wis/AutoAux is contracted to [22*s*19*p*18*d*18*f*17*g*7*h*4*i*] for 3*d*-block elements; VTZ-PP-F12-wis is contracted to [15*s*14*p*13*d*12*f*12*g*5*h*4*i*] and [14*s*13*p*12*d*11*f*11*g*5*h*3*i*] for 4*d* and 5*d*-block elements, respectively.

In contrast to DF-MP2-F12, adjusting the β geminal exponents in PNO-LMP2-F12 does not improve the error statistics. When β is set to 0.9 for VDZ(-PP)-F12-wis, we observe a deterioration of 0.10 kcal/mol relative to β = 1.4, mainly due to outliers in reactions 8 and 9. However, for VTZ(-PP)-F12-wis and β = 1.0, there is a slight increase of 0.07 kcal/mol in the RMSD for all reactions, compared with β = 1.4. The observed behavior could be attributed to the different MP2-F12 approximations used: DF-MP2-F12 employs the canonical 3C(FIX) ansatz,^41,42^ whereas PNO-LMP2-F12 uses a local 3*A(LOC,FIX) ansatz.^96^ For reactions 8 and 9, the deviations are much smaller than those in VDZ(-PP)-F12-wis with errors as low as −0.24 kcal/mol for the Δ*E*_reac_^#^ of reaction 8. We note that these two reactions have a high degree of static correlation, as shown in ref (86) and any discrepancies may be due to a combination of basis set incompleteness error and large multireference character, particularly for VDZ(-PP)-F12-wis.

#### 3.2.2 The CUAGAU-2 Set of Group-11 Metal Clusters

Let us now examine the effectiveness of the V*n*Z(-PP)-F12-wis basis sets for the electronic structure of the small transition metal clusters found within Chan’s CUAGAU-2 dataset.^35^ This dataset serves as an extension to the previously reported CUAGAU dataset,^106^ which included clusters of *M*_n_ (M = Cu, Ag, Au, with *n* = 2–4) and other relatively small metallic systems. For a comprehensive overview of some fundamentals for metal clusters, see ref (107), while ref (108) covers recent theoretical approaches in computational chemistry for the same.

We conducted DF-MP2-F12 calculations using various orbital basis sets for group-11 elements, including our VTZ(-PP)-F12-wis, the standard AVQZ(-PP), and two modified versions lacking *h*-type functions, namely, VQZ(-PP)(*g*) and AVQZ(-PP)(*g*-1) with a single *f* and *g* diffuse function excluded. For DF-MP2 ABS, we removed all *k*-functions from the AVQZ-PP/MP2Fit basis set for silver and gold and used the standard AVQZ/MP2Fit basis set for copper. For main group elements, we employed the standard VTZ-F12 as OBS, the VTZ-F12/MP2Fit as DF-MP2 ABS, and def2-QZVPP/JKFit as both JKFit and CABS.

Table 9 presents mean absolute deviations (in kcal/mol) for atomization energies (AE), ionization energies (IE), isomerization energies (ISO), binding energies (BE), and the total CUAGAU-2 dataset relative to conventional MP2 energies, which we calculated without density fitting and extrapolated from AV{Q,5}Z basis sets. For these MP2 and DF-MP2-F12 calculations, only the valence *d* and *s* electrons of the metal were correlated throughout. Table S6 in the Supporting Information displays the energy differences between V{Q,5}Z(-PP) and AV{Q,5}(-PP) extrapolations at the MP2 level with an RMSD of 0.46 kcal/mol between the two extrapolations.

**Table 9:** Mean Absolute Deviations for Selected Orbital Basis Sets and β Geminal Exponents in DF-MP2-F12 Relative to MP2/AV{Q,5}Z on the CUAGAU-2 Dataset

	Mean Absolute Deviations (kcal/mol)											
OBS	VTZ(-PP)-F12-wis			AVQZ(-PP)(*g* −1)			AVQZ(-PP)(*g*)			AVQZ(-PP)		
set	β = 0.9	β = 1.0	β = 1.4	β = 0.9	β = 1.0	β = 1.4	β = 0.9	β = 1.0	β = 1.4	β = 0.9	β = 1.0	β = 1.4
AE	0.35	0.42	0.26	0.36	0.39	0.68	0.26	0.27	0.14	0.28	0.29	0.22
IE	0.42	0.46	0.77	0.55	0.66	1.19	0.10	0.21	0.20	0.34	0.33	0.37
ISO	0.24	0.26	0.29	0.25	0.29	0.34	0.25	0.29	0.25	0.22	0.22	0.24
BE	0.40	0.40	0.48	0.31	0.33	0.47	0.55	0.48	0.43	0.40	0.36	0.35
CUAGAU-2	0.33	0.36	0.41	0.33	0.38	0.57	0.32	0.33	0.28	0.30	0.29	0.29

DF-MP2-F12/VTZ(-PP)-F12-wis with β = 0.9 yields excellent error statistics, with a MAD of 0.33 kcal/mol, for the entire CUAGAU-2 dataset and the lowest errors observed for atomization and isomerization energies, compared to MP2/AV{Q,5}Z. These results compare favorably to those obtained from standard AVQZ(-PP) and its truncated variants.

Moreover, we varied the β geminal exponent and performed MP2-F12 calculations for all species in the CUAGAU-2 dataset at each tested value (β = 0.9, 1.0, and 1.4). The MAD values remain relatively constant for both the standard AVQZ(-PP) and the truncated AVQZ(-PP)(*g*) for all choices of the β geminal exponents. The VTZ(-PP)-F12-wis basis set performs better than the same-sized AVQZ(-PP)(*g* – 1) for all tested β values, with a larger gap of 0.16 kcal/mol between the two basis sets for β = 1.4. Thus, we can argue that VTZ(-PP)-F12-wis represents an economical and efficient alternative to the standard AVQZ(-PP) basis set.

#### 3.2.3 Timing Comparison of Optimized Basis Sets

Can optimized V*n*Z(-PP)-F12-wis basis sets reduce the computational cost for F12 calculation relative to standard AV(*n*+1)Z-PP, for a species with a substantial percentage of metal atoms? And is either one economically worthwhile compared to non-F12 treatments of similar quality? We compared the computation time required for PNO-LCCSD(T), DLPNO–CCSD(T_1_) and DLPNO–CCSD(T_1_)-F12, where T_1_ indicates the iterative triples treatment from ref (90) for the Lindqvist anion polyoxometalate Mo_6_O_19_^–2^, at the geometry provided in ref (109) by Poblet and co-workers. (For reviews on polyoxometalates and their versatility as catalysts and redox agents, see Neumann and Weinstock^110^ and references therein.) All DLPNO–CCSD(T_1_) and DLPNO–CCSD(T_1_)-F12 calculations were carried out using the ORCA, and PNO-LCCSD(T) calculations were carried out using the MOLPRO on identical hardware: each job was run separately on an entire 52-core node with two Intel Xeon Gold 5320 CPUs (2.20 GHz) and a memory (RAM) of 768 GB. Wall clock times are listed in Table 10. We used default accuracy settings^96^ in MOLPRO and NormalPNO in ORCA as defined in Table 1 of ref (89); that is, TCutPNO, TCutMKN, TCutPairs, and TCutDO were set to 3.33 × 10^–7^*E*_h_, 1.00 × 10^–3^*E*_h_, 1.00 × 10^–4^*E*_h_, and 1.00 × 10^–2^*E*_h_, respectively. DLPNO–CCSD(T) is known to scale asymptotically linearly with the system size^90,91^ and DLPNO–CCSD(T)-F12 has an effective scaling exponent of 1.49 for *n*-alkanes up to 200 carbon atoms.^111^ Chan and Karton recently showed that DLPNO–CCSD(T_1_)-F12 scales linearly with system size for adamantane, diadamantane, pyracyclene, and chrysene.^112^

**Table 10:** Wall Clock Times (in hours) for the Computation of Single-Point Energy Calculations of Mo_6_O_19_^–2^ on a 52-Core Node, Comprising Two Intel Xeon Gold 5320 CPUs (2.20 GHz)

Basis set					Basis set					
*N*_bas_^a^	O atom	Mo atom	DLPNO–CCSD(T_1_) total wall clock time (h)^b^	PNO-LCCSD(T) total wall clock time (h)^b^	*N*_bas_^a^	O atom	Mo atom	DLPNO–CCSD(T_1_)-F12 total wall clock time (*h*)	triples correction (% of total wall clock time)	F12 correction (% of total wall clock time)
494	VDZ	VDZ-PP	2.32 (85%)	5.68 (48%)	990	VDZ-F12	AVTZ-PP(*f*)	22.19	43%	40%
948	VTZ	VTZ-PP	10.43 (73%)	17.92 (65%)	948	VDZ-F12	AVTZ-PP(*f*-1)	18.54	46%	37%
1639	VQZ	VQZ-PP	44.06 (63%)	64.12 (67%)	948	VDZ-F12	VDZ-PP-F12-wis	18.64	46%	37%
665	AVDZ	VDZ-PP	5.03 (73%)	7.67 (57%)	1589	VTZ-F12	AVQZ-PP(*g*-1)	83.98	40%	41%
1252	AVTZ	VTZ-PP	26.60 (71%)	32.95 (68%)	1589	VTZ-F12	VTZ-PP-F12-wis	84.24	40%	41%
2114	AVQZ	VQZ-PP	117.2 (60%)	113.8 (71%)						

a Total number of orbital basis set functions for Mo_6_O_19_^–2^

b Triples correction (% of total wall clock time).

We utilize def2-QZVPP/JKFit^77^ as JKFit throughout, and V*n*Z-F12/MP2Fit^62^ (*n* = D, T) and awCVTZ-PP/MP2Fit^63^ for oxygen and molybdenum as MP2Fit basis, respectively. V*n*Z-F12/OptRI^113^ is used as CABS for oxygen, while our automatically generated autoCABSs,^17^ derived from the corresponding OBS, are employed for molybdenum.

In conjunction with VDZ-F12 on oxygen, our proposed VDZ-PP-F12-wis basis set for molybdenum reduces the DLPNO–CCSD(T_1_)-F12 computational time for Mo_6_O_19_^–2^ by ∼16%, compared to AVTZ-PP(*f*) for calculations on the polyoxometalate (POM) anion. The F12 correction time makes up 37% of the total wall clock time with VDZ-PP-F12-wis, while the triples term accounts for ∼46%. An orbital-only calculation of comparable quality would be a V{T,Q}Z-PP extrapolation: the cumulative time of DLPNO–CCSD(T_1_)/VTZ and /VQZ calculations, ∼54 h, is about three times that with VDZ-F12. This, explicitly correlated localized coupled cluster calculations in conjunction with a VDZ-F12 quality basis set are a more economical alternative than localized coupled cluster for polyoxometalates and similar systems.

However, in view of the anionic character of the POM and the large number of highly electronegative oxygen atoms, it can legitimately be argued that AV*n*Z should have been used on that element. (In contrast, VnZ-F12 for oxygen already contains some anion functions.) If we use AV*n*Z and V*n*Z-PP basis sets for oxygen and molybdenum in DLPNO–CCSD(T_1_), respectively, the wall clock times are more than doubled, compared to the previous V*n*Z and V*n*Z-PP combination, tilting the balance further in favor of the F12 calculation. Our most costly DLPNO–CCSD(T_1_) calculation requires up to 117 h for AVQZ and VQZ-PP and 26 h for AVTZ and VTZ-PP, or a total of 143 h, about seven times what was required for VDZ-F12. Additionally, we were able to carry out DLPNO–CCSD(T_1_)-F12 calculations employing our VTZ-PP-F12-wis basis set and the truncated AVQZ-PP(*g*-1) for molybdenum, and both calculations took ∼84 h. The triples correction term accounted for 40% of the total wall clock time, while the F12 term accounted for 41%.

We encountered linear dependency issues in DLPNO–CCSD(T_1_)-F12 calculations with larger basis sets such as VTZ-PP-F12-wis and AVQZ-PP(*g*-1) as the OBS and our autoCABSs as the CABS. However, no such warnings occurred in MOLPRO calculations, where no functions were deleted during CABS processing. Comparing the HF CABS correction between ORCA and MOLPRO, we found large deviations in ORCA, while MOLPRO provided accurate estimates for the HF basis set limit, as established from HF/AV{Q,5}Z(-PP) canonical calculations. Further investigation revealed that for the halogen dimers X_2_ using standard cc-pV*n*Z(-PP)-F12 basis sets, the problem occurs only for Br_2_ and I_2_ (where an ECP is present) but not for Cl_2_ (where it is not). Similar conclusions were drawn when comparing the HF CABS terms of CrO_3_^–2^ and MoO_3_^–2^, with discrepancies of 0.1 *E*_h_ in the CABS correction for Mo when using VDZ-PP-F12-wis with ECP28MDF (Tables S10–S13 in the Supporting Information, ).

## 4 Conclusions

In this study, we systematically optimized V*n*Z(-PP)-F12-wis orbital basis sets, *n* = D and T, inspired by the work of Shaw and Hill.^25^ We then assessed their performance for practical applications, by means of benchmarks for representative organometallic reaction barrier heights (MOBH35) and metal clusters (CUAGAU-2). We found that, in an F12 context, the V*n*Z(-PP)-F12-wis transition-metal basis sets perform comparably with the combination of V*n*Z-F12 on the main group elements with AV(*n* + 1)Z(-PP) on the transition metals. The latter is an adequate alternative if custom basis sets are unavailable or undesired with the electronic structure system at hand.

While our transition metal basis sets are more compact than AV(*n* + 1)Z(-PP), the computational speed gain for organometallic applications is negligible, as only one or a few transition-metal atoms are present together with dozens of main-group atoms. In transition-metal clusters, however, our basis sets may lead to CPU time savings.

As a proof of concept, a DLPNO–CCSD(T_1_)-F12/VDZ-F12 calculation on a small polyoxometalate is shown to be much more economical than a DLPNO–CCSD(T_1_)/V{T,Q}Z calculation of comparable quality.

For early transition metals, it might be desirable to have core–valence basis sets; on the other hand, it may again be that AWCV(*n* + 1)Z(-PP) on the metal offers similar performance. We will address this question in a future communication.

## Data Availability

Any raw data not already provided in the Supporting Information can be obtained from the authors upon reasonable request.

## Supporting Information Available

Basis sets and ECPs in MOLPRO format; frozen core settings for MOBH35 dataset (Table S1); energies of *d*-block elements using VTZ(-PP)-F12-wis basis sets (Table S2); optimized basis set exponents (Table S3) and plots (Figure S1) for VDZ(-PP)-F12-wis; effects of *k* and *l* functions of V*n*Z(-PP)/MP2Fit basis sets in DF-MP2/CBS (Table S4); deviations of DF-MP2-F12 from DF-MP2/V{Q,5}Z in the MOBH35 dataset using various β geminal exponents and VDZ(-PP)-F12-wis basis sets (Table S5); energy differences between V{Q,5}Z(-PP) and AV{Q,5}Z(-PP) at MP2 for the CUAGAU-2 dataset (Table S6); basis set exponents comparison (Table S7); energies of neutral atom states using truncated VnZ(-PP)-F12(d) basis sets (Table S8); multireference character diagnostics for *d*-block elements (Table S9); HF basis set limit estimates of selected molecules (Tables S10–S13); basis set functions for selected values of geminal exponents β used during optimization (Table S14) (PDF)
cc-pVDZ-F12-wis basis set in MOLPRO format (TXT)
cc-pVDZ-PP-F12-wis basis set in MOLPRO format(TXT)
cc-pVTZ-F12-wis basis set in MOLPRO format (TXT)
cc-pVTZ-PP-F12-wis basis set in MOLPRO format (TXT)
Full raw energies and statistics for MOBH35 sets (XLSX)
Full raw energies and statistics for CUAGAU-2 sets (XLSX)
