# Supplementary material for: Correlation Consistent Basis Sets for Explicitly Correlated Theory: The Transition Metals
Source: J Chem Theory Comput. 2023 Aug 4;19(17):5806–20. doi: 10.1021/acs.jctc.3c00506 (PMC10500978; doi:10.1021/acs.jctc.3c00506)
Supplement: Supplementary file 1 — ct3c00506_si_001.pdf [file ct3c00506_si_001.pdf]

Supporting information for:
Correlation Consistent Basis Sets for Explicitly Correlated Theory: The Transition Metals

”

Emmanouil Semidalas and Jan M. L. Martin[1]

[1]Department of Molecular Chemistry and Materials Science, Weizmann Institute of Science, 7610001, Reḥovot, Israel. Email: gershom@weizmann.ac.il

Contents

Figure S1. Optimized f exponents on a logarithmic scale for atoms Sc-Zn, Y-Cd, and Hf-Hg using our VDZ(-PP)-F12-wis basis sets. 2

Figure S2. The T_1 and D_2 diagnostic values for all electronic states of d-block elements calculated using CCSD(T)-F12/VDZ(-PP)-wis. 2

Table S1. Frozen Core Electrons per Molecule in MOBH35 with Frozen Metal's (n–1)sp Electrons for Post-HF Calculations. 3

Table S2. Energies (in E_h) of d-Block Elements Obtained using our cc-pVTZ(-PP)-F12-wis Basis Set. 6

Table S3. Optimized Exponents for d-Block Elements. 9

Table S4. Effects of k and l Functions (kcal/mol) of VnZ(-PP)/MP2Fit Basis Sets in DF-MP2/V{Q,5}Z in the MOBH35 Dataset. 12

Table S5. Deviations Between DF-MP2-F12 and DF-MP2/V{Q,5}Z in the MOBH35 Dataset Using Our VDZ(-PP)-F12-wis Basis Sets and Different β Geminal Exponents. 15

Table S6. Energy Differences (kcal/mol) for V{Q,5}Z(-PP) and AV{Q,5}Z(-PP) Extrapolations in Conventional MP2 for the Energetics of CUAGAU-2 dataset. 18

Table S7. Basis Set Exponents (f and g) and Differences: Our VnZ(-PP)-F12 Basis Sets vs. Shaw and Hill's (SH's) VnZ-PP-F12 Basis Sets (n=D,T). 22

Table S8. Total Energies of Neutral Atom States using our VnZ(-PP)-F12(d) Basis Sets (n=D,T) and Shaw and Hill's (SH's) VnZ-PP-F12(d) Basis Sets with up to d Functions Throughout. 23

Table S9. Multireference Character Diagnostics of d-Block Elements Obtained Using our cc-pVDZ(-PP)-F12-wis Basis Set in CCSD(T)-F12. 24

Table S10. HF basis set limit estimates for the polyoxometalate $Mo_6O_{19}^{-2}$. 27

Table S11. HF basis set limit estimates for group-17 diatomic molecules 28

Table S12. HF basis set limit estimates for MoO_3^{-2} 29

Table S13. HF basis set limit estimates for CrO_3^{-2} 30
Table S14. Basis sets functions for selected geminal exponents β used during optimization. 31
cc-pVDZ-F12-wis Basis Set: Sc-Zn Atoms 32
cc-pVDZ-PP-F12-wis Basis Set: Y-Cd and Hf-Hg Atoms 42
cc-pVTZ-F12-wis Basis Set: Sc-Zn Atoms 52
cc-pVTZ-PP-F12-wis Basis Set: Y-Cd and Hf-Hg Atoms 62
ECP28MDF: Y-Cd Atoms 81
ECP60MDF: Hf-Hg Atoms 83

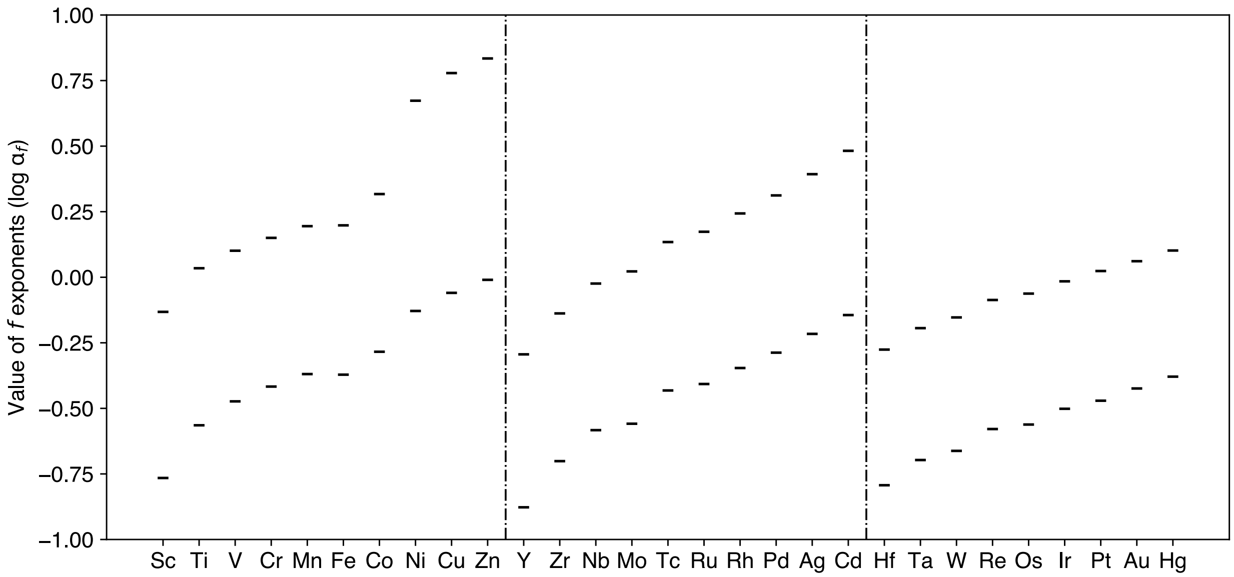

Figure S1. Optimized *f* exponents on a logarithmic scale for atoms Sc-Zn, Y-Cd, and Hf-Hg using our VDZ(-PP)-F12-wis basis sets.

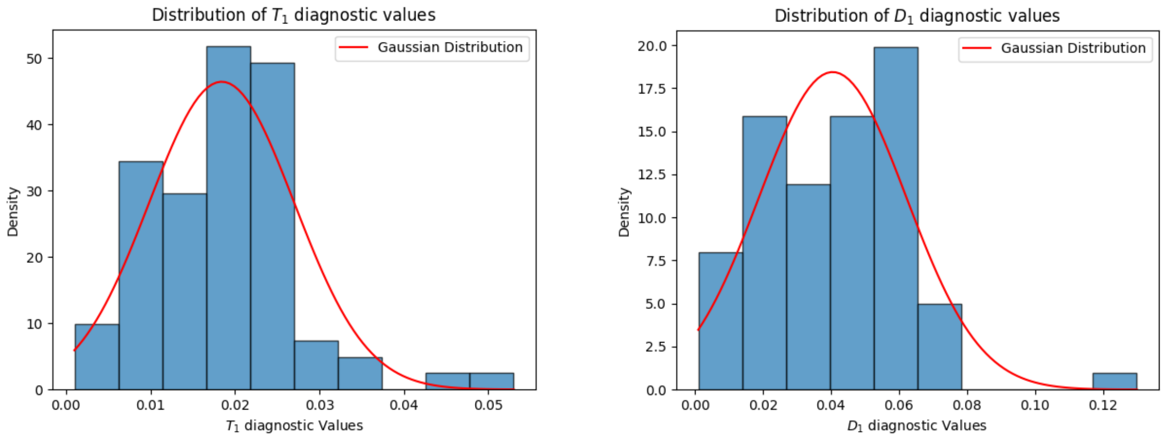

Figure S2. The T_1 and D_2 diagnostic values for all electronic states of *d*-block elements calculated using CCSD(T)-F12/VDZ(-PP)-wis.

Table S1. Frozen Core Electrons per Molecule in MOBH35 with Frozen Metal's (n–1)*sp* Electrons for Post-HF Calculations.

Molecule	Number of frozen core electrons
P_1	72
R_1	72
TS_1	72
P_2	64
R_2	64
TS_2	64
P_3	38
R_3	38
TS_3	38
P_4	36
R_4	36
TS_4	36
P_5	80
R_5	80
TS_5	80
P_6	66
R_6	66
TS_6	66
P_7	66
R_7	66
TS_7	66
P_8	84
R_8	84
TS_8	84
P_9	84
R_9	84
TS_9	84
P_{10}	56
R_{10}	60
TS_{10}	60
P_{11}	64
R_{11}	64
TS_{11}	64
P_{12}	74
R_{12}	74
TS_{12}	74
P_{13}	72
R_{13}	72

TS_{13}	72
P_{14}	50
R_{14}	50
TS_{14}	50
P_{15}	38
R_{15}	38
TS_{15}	38
P_{16}	28
R_{16}	28
TS_{16}	28
P_{17}	98
R_{17}	120
TS_{17}	120
P_{18}	98
R_{18}	120
TS_{18}	120
P_{19}	98
R_{19}	120
TS_{19}	120
P_{20}	98
R_{20}	120
TS_{20}	120
P_{21}	38
R_{21}	38
TS_{21}	38
P_{22}	50
R_{22}	50
TS_{22}	50
P_{23}	54
R_{23}	54
TS_{23}	54
P_{24}	100
R_{24}	100
TS_{24}	100
P_{25}	96
R_{25}	96
TS_{25}	96
P_{26}	44
R_{26}	44
TS_{26}	44
P_{27}	48
R_{27}	48
TS_{27}	48
P_{28}	68
R_{28}	68
TS_{28}	68

P_{29}	68
R_{29}	68
TS_{29}	68
P_{30}	48
R_{30}	48
TS_{30}	48
P_{31}	48
R_{31}	48
TS_{31}	48
P_{32}	38
R_{32}	38
TS_{32}	38
P_{33}	14
R_{33}	14
TS_{33}	14
P_{34}	42
R_{34}	42
TS_{34}	42
P_{35}	40
R_{35}	42
TS_{35}	42

R: reactant, P: product, TS: transition state

Table S2. Energies (in E_h) of *d*-Block Elements Obtained using our cc-pVTZ(-PP)-F12-wis Basis Set.

State		M	HF	HF+CABS	MP2-F12	State averaged MP2-F12
s^2d^1	^2D	Sc	-759.737507	-759.737513	-759.769296	-759.694070
s^1d^2	^4F		-759.692532	-759.692558	-759.709063	
d^3	^4F		-759.572436	-759.572460	-759.603850	
s^2d^2	^3F	Ti	-848.396944	-848.396976	-848.444090	-848.377288
s^1d^3	^5F		-848.387903	-848.387930	-848.422788	
d^4	^5D		-848.241524	-848.241572	-848.264986	
s^2d^3	^4F	V	-942.886424	-942.886457	-942.953745	-942.892463
s^1d^4	^6D		-942.881794	-942.881845	-942.947781	
d^5	^6S		-942.724611	-942.724860	-942.775864	
s^2d^4	^5D	Cr	-1043.312202	-1043.312276	-1043.168945	-1043.346654
s^1d^5	^7S		-1043.356321	-1043.356328	-1043.459123	
d^6	^5D		-1043.060352	-1043.060688	-1043.411892	
s^2d^5	^6S	Mn	-1149.748828	-1149.748889	-1149.929093	-1149.834764
s^1d^6	^6D		-1149.748713	-1149.748771	-1149.929174	
d^7	^4F		-1149.470688	-1149.471176	-1149.646024	
s^2d^6	^5D	Fe	-1262.448475	-1262.448608	-1262.659277	-1262.620333
s^1d^7	^5F		-1262.380859	-1262.380940	-1262.644054	
d^8	^3F		-1262.173265	-1262.173373	-1262.557670	
s^2d^7	^4F	Co	-1381.418141	-1381.418239	-1381.707686	-1381.696060
s^1d^8	^4F		-1381.361088	-1381.361180	-1381.718628	
d^9	^2D		-1381.159548	-1381.159703	-1381.661868	
s^2d^8	^3F	Ni	-1506.857633	-1506.857768	-1507.241087	-1507.275690
s^1d^9	^3D		-1506.827051	-1506.827172	-1507.291924	
d^{10}	^1S		-1506.669618	-1506.669663	-1507.294060	
s^2d^9	^2D	Cu	-1638.953318	-1638.953449	-1639.436376	-1639.488334
s^1d^{10}	^2S		-1638.963635	-1638.963654	-1639.540292	
s^2d^{10}	^1S	Zn	-1777.848027	-1777.848032	-1778.438409	-1778.438409

s^2d^1	^2D	Y	-37.707897	-37.707899	-37.743166	-37.679311
s^1d^2	^4F		-37.674415	-37.674425	-37.689543	
d^3	^4F		-37.585761	-37.585768	-37.605225	
s^2d^2	^3F	Zr	-46.291824	-46.291834	-46.345466	-46.279320
s^1d^3	^5F		-46.300023	-46.300034	-46.330682	
d^4	^5D		-46.143652	-46.143715	-46.161813	
s^2d^3	^4F	Nb	-56.115144	-56.115157	-56.190039	-56.135456
s^1d^4	^6D		-56.144725	-56.144742	-56.201137	
d^5	^6S		-55.975132	-55.975326	-56.015190	
s^2d^4	^5D	Mo	-67.183809	-67.257239	-67.362415	-67.367805
s^1d^5	^7S		-67.342414	-67.342416	-67.427327	
d^6	^5D		-67.257222	-67.183880	-67.313672	
s^2d^5	^6S	Tc	-79.843602	-79.817053	-79.985469	-79.882620
s^1d^6	^6D		-79.816967	-79.843603	-79.970293	
d^7	^4F		-79.548078	-79.548888	-79.692100	
s^2d^6	^5D	Ru	-93.784672	-93.784779	-93.994178	-94.003754
s^1d^7	^5F		-93.799546	-93.799697	-94.023412	
d^8	^3F		-93.725299	-93.725417	-93.993672	
s^2d^7	^4F	Rh	-109.334810	-109.334971	-109.614505	-109.658656
s^1d^8	^4F		-109.384058	-109.384199	-109.682221	
d^9	^2D		-109.327132	-109.327209	-109.679242	
s^2d^8	^3F	Pd	-126.521796	-126.521944	-126.884340	-126.975003
s^1d^9	^3D		-126.608786	-126.608887	-126.993669	
d^{10}	^1S		-126.609249	-126.609253	-127.047000	
s^2d^9	^2D	Ag	-145.917491	-145.917577	-146.366916	-146.448014
s^1d^{10}	^2S		-146.055167	-146.055169	-146.529112	
s^2d^{10}	^1S	Cd	-166.838943	-166.838944	-167.382481	-167.382481

s^2d^2	^3F	Hf	-47.936262	-47.936271	-47.994524	-47.889739
s^1d^3	^5F		-47.909205	-47.909219	-47.939759	
d^4	^5D		-47.717387	-47.717445	-47.734933	
s^2d^3	^4F	Ta	-56.737459	-56.737466	-56.818063	-56.720328
s^1d^4	^6D		-56.730668	-56.730681	-56.785229	
d^5	^6S		-56.519989	-56.520173	-56.557692	
s^2d^4	^5D	W	-66.671978	-66.671995	-66.784441	-66.742872
s^1d^5	^7S		-66.717146	-66.717147	-66.799728	
d^6	^5D		-66.518301	-66.518387	-66.644446	
s^2d^5	^6S	Re	-77.827942	-77.827943	-77.975198	-77.826914
s^1d^6	^6D		-77.764013	-77.764093	-77.914106	
d^7	^4F		-77.452174	-77.452604	-77.591438	
s^2d^6	^5D	Os	-90.061927	-90.062025	-90.276508	-90.232484
s^1d^7	^5F		-90.030396	-90.030538	-90.250099	
d^8	^3F		-89.916365	-89.916492	-90.170846	
s^2d^7	^4F	Ir	-103.617082	-103.617259	-103.899644	-103.643703
s^1d^8	^4F		-103.611860	-103.612012	-103.902200	
d^9	^2D		-102.923931	-102.924720	-103.129265	
s^2d^8	^3F	Pt	-118.487699	-118.487835	-118.850224	-118.872462
s^1d^9	^3D		-118.512190	-118.512269	-118.884844	
d^{10}	^1S		-118.476762	-118.476765	-118.882318	
s^2d^9	^2D	Au	-134.715022	-134.715112	-135.155261	-135.193451
s^1d^{10}	^2S		-134.780858	-134.780861	-135.231641	
s^2d^{10}	^1S	Hg	-152.540833	-152.540834	-153.0650601	-153.0650601

Table S3. Optimized Exponents for d-Block Elements.

	optimized *f* exponents of VDZ-F12-wis	
Element	f_1	f_2
Sc	0.172	0.738
Ti	0.273	1.082
V	0.336	1.262
Cr	0.383	1.413
Mn	0.427	1.566
Fe	0.425	1.577
Co	0.520	2.076
Ni	0.744	4.712
Cu	0.872	6.006
Zn	0.978	6.828

	optimized *f* exponents of VTZ-PP-F12-wis			optimized *g* exponents of VTZ-PP-F12-wis	
Element	f_1	f_2	f_3	g_1	g_2
Sc	0.115	0.375	1.357	0.159	0.510
Ti	0.163	0.514	1.766	0.231	0.739
V	1.930	0.596	0.194	0.284	0.917
Cr	0.244	0.743	2.344	0.380	1.178
Mn	0.460	1.204	3.465	0.683	2.817
Fe	0.351	1.231	5.053	0.581	3.788
Co	0.416	1.448	6.579	0.718	3.948
Ni	0.503	1.681	7.639	0.740	4.498
Cu	0.616	2.406	9.327	0.808	5.138
Zn	0.743	3.056	11.259	0.821	5.631

Table S3. Optimized Exponents for d-Block Elements (continued).

	optimized f exponents of VDZ-PP-F12-wis	
Element	f_1	f_2
Y	0.133	0.508
Zr	0.199	0.728
Nb	0.261	0.946
Mo	0.276	1.053
Tc	0.370	1.362
Ru	0.392	1.491
Rh	0.451	1.751
Pd	0.516	2.051
Ag	0.608	2.472
Cd	0.718	3.034
Hf	0.161	0.530
Ta	0.201	0.640
W	0.218	0.703
Re	0.264	0.819
Os	0.274	0.866
Ir	0.315	0.965
Pt	0.338	1.056
Au	0.377	1.151
Hg	0.418	1.265

Table S3. Optimized Exponents for d-Block Elements (continued).

	optimized *f* exponents of VTZ-PP-F12-wis			optimized *g* exponents of VTZ-PP-F12-wis	
Element	f_1	f_2	f_3	g_1	g_2
Y	0.096	0.287	0.980	0.138	0.410
Zr	0.138	0.412	1.423	0.189	0.568
Nb	0.162	0.524	1.795	0.212	0.696
Mo	0.128	0.631	2.687	0.266	0.931
Tc	0.279	0.865	3.362	0.342	1.014
Ru	0.300	0.970	3.937	0.370	1.117
Rh	0.343	1.149	4.817	0.384	1.320
Pd	0.393	1.330	5.756	0.551	1.476
Ag	0.464	1.544	6.701	0.587	2.087
Cd	0.530	1.769	7.615	0.648	2.471
Hf	0.088	0.245	0.688	0.186	0.547
Ta	0.053	0.240	0.705	0.269	0.806
W	0.141	0.375	0.965	0.259	0.783
Re	0.160	0.419	1.071	0.316	0.918
Os	0.183	0.484	1.221	0.332	0.995
Ir	0.075	0.308	0.964	0.417	1.102
Pt	0.178	0.474	1.298	0.452	1.480
Au	0.269	0.704	1.808	0.478	1.445
Hg	0.297	0.778	2.031	0.546	1.829

Table S4. Effects of *k* and *l* Functions (kcal/mol) of V*n*Z(-PP)/MP2Fit Basis Sets in DF-MP2/ V{Q,5}Z in the MOBH35 Dataset.

		ΔE MP2/cc-pV{Q,5}Z		
Reaction		without *k*, *l* functions in DF-MP2 ABS	wiith *k*, *l* functions in DF-MP2 ABS	δΔE
1	$\Delta E^{\#}_{fwd}$	26.42	26.41	-0.01
1	$\Delta E^{\#}_{rev}$	11.89	11.89	0.00
1	ΔE_{reac}	14.53	14.52	-0.01
2	$\Delta E^{\#}_{fwd}$	6.33	6.33	0.00
2	$\Delta E^{\#}_{rev}$	23.40	23.41	0.01
2	ΔE_{reac}	-17.08	-17.08	-0.01
3	$\Delta E^{\#}_{fwd}$	0.88	0.88	0.00
3	$\Delta E^{\#}_{rev}$	20.92	20.94	0.02
3	ΔE_{reac}	-20.04	-20.06	-0.02
4	$\Delta E^{\#}_{fwd}$	3.10	3.10	0.00
4	$\Delta E^{\#}_{rev}$	2.58	2.59	0.01
4	ΔE_{reac}	0.52	0.51	-0.02
5	$\Delta E^{\#}_{fwd}$	2.24	2.27	0.03
5	$\Delta E^{\#}_{rev}$	25.80	25.79	0.00
5	ΔE_{reac}	-23.56	-23.52	0.04
6	$\Delta E^{\#}_{fwd}$	14.40	14.40	0.00
6	$\Delta E^{\#}_{rev}$	12.09	12.09	0.00
6	ΔE_{reac}	2.32	2.31	0.00
7	$\Delta E^{\#}_{fwd}$	23.80	23.80	0.00
7	$\Delta E^{\#}_{rev}$	13.93	13.93	-0.01
7	ΔE_{reac}	9.86	9.87	0.01
8	$\Delta E^{\#}_{fwd}$	43.42	43.41	-0.01
8	$\Delta E^{\#}_{rev}$	40.65	40.62	-0.02
8	ΔE_{reac}	2.77	2.78	0.01
9	$\Delta E^{\#}_{fwd}$	30.16	30.18	0.03
9	$\Delta E^{\#}_{rev}$	0.04	0.04	0.01
9	ΔE_{reac}	30.12	30.14	0.02
10	$\Delta E^{\#}_{fwd}$	-4.54	-4.55	0.00
10	$\Delta E^{\#}_{rev}$	12.54	12.53	-0.01
10	ΔE_{reac}	-17.09	-17.08	0.01
11	$\Delta E^{\#}_{fwd}$	32.81	32.81	0.00
11	$\Delta E^{\#}_{rev}$	86.13	86.12	-0.01
11	ΔE_{reac}	-53.32	-53.31	0.00
12	$\Delta E^{\#}_{fwd}$	5.18	5.19	0.01

12	$\Delta E^{\#}_{rev}$	36.39	36.39	0.00
12	ΔE_{reac}	-31.21	-31.20	0.01
13	$\Delta E^{\#}_{fwd}$	12.46	12.44	-0.02
13	$\Delta E^{\#}_{rev}$	42.87	42.85	-0.02
13	ΔE_{reac}	-30.41	-30.41	0.00
14	$\Delta E^{\#}_{fwd}$	9.17	9.16	-0.01
14	$\Delta E^{\#}_{rev}$	13.71	13.72	0.01
14	ΔE_{reac}	-4.54	-4.56	-0.02
15	$\Delta E^{\#}_{fwd}$	15.80	15.86	0.06
15	$\Delta E^{\#}_{rev}$	78.85	78.85	0.00
15	ΔE_{reac}	-63.04	-62.99	0.06
16	$\Delta E^{\#}_{fwd}$	34.07	34.13	0.06
16	$\Delta E^{\#}_{rev}$	61.33	61.30	-0.03
16	ΔE_{reac}	-27.26	-27.17	0.09
21	$\Delta E^{\#}_{fwd}$	7.16	7.15	0.00
21	$\Delta E^{\#}_{rev}$	7.16	7.16	0.00
21	ΔE_{reac}	-0.01	-0.01	0.00
22	$\Delta E^{\#}_{fwd}$	10.47	10.47	0.00
22	$\Delta E^{\#}_{rev}$	25.02	25.02	0.00
22	ΔE_{reac}	-14.55	-14.55	0.01
23	$\Delta E^{\#}_{fwd}$	25.30	25.29	-0.01
23	$\Delta E^{\#}_{rev}$	14.52	14.49	-0.03
23	ΔE_{reac}	10.78	10.79	0.02
26	$\Delta E^{\#}_{fwd}$	32.31	32.34	0.03
26	$\Delta E^{\#}_{rev}$	0.05	0.05	0.00
26	ΔE_{reac}	32.26	32.30	0.03
27	$\Delta E^{\#}_{fwd}$	15.09	15.07	-0.01
27	$\Delta E^{\#}_{rev}$	3.73	3.73	0.00
27	ΔE_{reac}	11.36	11.35	-0.01
28	$\Delta E^{\#}_{fwd}$	32.63	32.50	-0.14
28	$\Delta E^{\#}_{rev}$	10.50	10.54	0.04
28	ΔE_{reac}	22.13	21.96	-0.18
29	$\Delta E^{\#}_{fwd}$	13.51	13.51	0.00
29	$\Delta E^{\#}_{rev}$	29.65	29.65	0.00
29	ΔE_{reac}	-16.14	-16.14	0.00
30	$\Delta E^{\#}_{fwd}$	7.72	7.71	-0.01
30	$\Delta E^{\#}_{rev}$	13.22	13.17	-0.05
30	ΔE_{reac}	-5.50	-5.46	0.04
31	$\Delta E^{\#}_{fwd}$	-0.87	-0.98	-0.11
31	$\Delta E^{\#}_{rev}$	10.31	10.43	0.12

31	ΔE_{reac}	-11.17	-11.41	-0.23
32	$\Delta E^{\#}_{fwd}$	16.02	16.03	0.01
32	$\Delta E^{\#}_{rev}$	79.64	79.63	0.00
32	ΔE_{reac}	-63.62	-63.60	0.02
33	$\Delta E^{\#}_{fwd}$	-1.04	-0.97	0.07
33	$\Delta E^{\#}_{rev}$	2.00	1.91	-0.08
33	ΔE_{reac}	-3.04	-2.88	0.15
34	$\Delta E^{\#}_{fwd}$	30.07	30.23	0.16
34	$\Delta E^{\#}_{rev}$	-0.82	-0.87	-0.05
34	ΔE_{reac}	30.89	31.10	0.21
35	$\Delta E^{\#}_{fwd}$	19.49	19.52	0.03
35	$\Delta E^{\#}_{rev}$	-1.72	-1.72	0.01
35	ΔE_{reac}	21.21	21.24	0.03

Table S5. Deviations Between DF-MP2-F12 and DF-MP2/V{Q,5}Z in the MOBH35 Dataset Using Our VDZ(-PP)-F12-wis Basis Sets and Different β Geminal Exponents.

		δΔE DF-MP2/cc-pV{Q,5}Z	
Reaction		$\beta = 0.9$	$\beta = 1.4$
1	$\Delta E^{\#}_{fwd}$	-0.14	0.04
1	$\Delta E^{\#}_{rev}$	-0.07	-0.05
1	ΔE_{reac}	-0.07	0.09
2	$\Delta E^{\#}_{fwd}$	-0.09	-0.08
2	$\Delta E^{\#}_{rev}$	-0.04	0.21
2	ΔE_{reac}	-0.06	-0.28
3	$\Delta E^{\#}_{fwd}$	-0.06	0.00
3	$\Delta E^{\#}_{rev}$	0.19	0.25
3	ΔE_{reac}	-0.24	-0.25
4	$\Delta E^{\#}_{fwd}$	-0.04	-0.04
4	$\Delta E^{\#}_{rev}$	0.18	0.37
4	ΔE_{reac}	-0.22	-0.41
5	$\Delta E^{\#}_{fwd}$	0.06	-0.02
5	$\Delta E^{\#}_{rev}$	-0.07	-0.13
5	ΔE_{reac}	0.14	0.11
6	$\Delta E^{\#}_{fwd}$	-0.33	-0.42
6	$\Delta E^{\#}_{rev}$	0.15	0.25
6	ΔE_{reac}	-0.48	-0.67
7	$\Delta E^{\#}_{fwd}$	-0.09	0.13
7	$\Delta E^{\#}_{rev}$	-0.14	-0.29
7	ΔE_{reac}	0.05	0.42
8	$\Delta E^{\#}_{fwd}$	0.14	0.51
8	$\Delta E^{\#}_{rev}$	0.11	-0.02
8	ΔE_{reac}	0.03	0.53
9	$\Delta E^{\#}_{fwd}$	-0.28	-0.09
9	$\Delta E^{\#}_{rev}$	0.22	-0.07
9	ΔE_{reac}	-0.50	-0.01
10	$\Delta E^{\#}_{fwd}$	-0.50	-0.28
10	$\Delta E^{\#}_{rev}$	-0.06	0.04
10	ΔE_{reac}	-0.44	-0.32
11	$\Delta E^{\#}_{fwd}$	-0.54	-0.27
11	$\Delta E^{\#}_{rev}$	0.37	0.57
11	ΔE_{reac}	-0.91	-0.85
12	$\Delta E^{\#}_{fwd}$	0.08	0.12
12	$\Delta E^{\#}_{rev}$	-0.08	0.10

12	ΔE_{reac}	0.16	0.02
13	$\Delta E^{\#}_{fwd}$	0.36	0.20
13	$\Delta E^{\#}_{rev}$	0.32	0.68
13	ΔE_{reac}	0.05	-0.49
14	$\Delta E^{\#}_{fwd}$	-0.19	-0.17
14	$\Delta E^{\#}_{rev}$	-0.12	0.11
14	ΔE_{reac}	-0.08	-0.28
15	$\Delta E^{\#}_{fwd}$	-0.76	-0.64
15	$\Delta E^{\#}_{rev}$	-0.50	-0.61
15	ΔE_{reac}	-0.26	-0.03
16	$\Delta E^{\#}_{fwd}$	0.19	0.20
16	$\Delta E^{\#}_{rev}$	-0.14	-0.20
16	ΔE_{reac}	0.33	0.40
17	$\Delta E^{\#}_{fwd}$	-0.37	0.89
17	$\Delta E^{\#}_{rev}$	-0.51	-1.34
17	ΔE_{reac}	0.15	2.23
18	$\Delta E^{\#}_{fwd}$	-0.54	0.18
18	$\Delta E^{\#}_{rev}$	-0.63	-0.98
18	ΔE_{reac}	0.10	1.16
19	$\Delta E^{\#}_{fwd}$	-0.59	0.06
19	$\Delta E^{\#}_{rev}$	-0.72	-1.48
19	ΔE_{reac}	0.13	1.54
20	$\Delta E^{\#}_{fwd}$	-1.06	-1.16
20	$\Delta E^{\#}_{rev}$	-0.92	-1.52
20	ΔE_{reac}	-0.14	0.36
21	$\Delta E^{\#}_{fwd}$	-0.14	0.00
21	$\Delta E^{\#}_{rev}$	-0.14	0.00
21	ΔE_{reac}	0.00	0.00
22	$\Delta E^{\#}_{fwd}$	0.03	0.01
22	$\Delta E^{\#}_{rev}$	0.06	0.15
22	ΔE_{reac}	-0.03	-0.14
23	$\Delta E^{\#}_{fwd}$	0.31	0.39
23	$\Delta E^{\#}_{rev}$	0.12	0.35
23	ΔE_{reac}	0.19	0.04
24	$\Delta E^{\#}_{fwd}$	0.13	0.17
24	$\Delta E^{\#}_{rev}$	0.24	0.02
24	ΔE_{reac}	-0.11	0.15
25	$\Delta E^{\#}_{fwd}$	0.21	0.26
25	$\Delta E^{\#}_{rev}$	0.23	0.09
25	ΔE_{reac}	-0.02	0.18

26	$\Delta E^{\#}_{fwd}$	-0.07	-0.19
26	$\Delta E^{\#}_{rev}$	0.01	0.01
26	ΔE_{reac}	-0.08	-0.20
27	$\Delta E^{\#}_{fwd}$	0.05	0.00
27	$\Delta E^{\#}_{rev}$	-0.09	-0.13
27	ΔE_{reac}	0.14	0.13
28	$\Delta E^{\#}_{fwd}$	0.02	0.17
28	$\Delta E^{\#}_{rev}$	-0.06	0.09
28	ΔE_{reac}	0.08	0.08
29	$\Delta E^{\#}_{fwd}$	-0.09	-0.07
29	$\Delta E^{\#}_{rev}$	-0.03	0.17
29	ΔE_{reac}	-0.06	-0.25
30	$\Delta E^{\#}_{fwd}$	0.01	0.14
30	$\Delta E^{\#}_{rev}$	-0.06	0.32
30	ΔE_{reac}	0.07	-0.18
31	$\Delta E^{\#}_{fwd}$	0.13	0.35
31	$\Delta E^{\#}_{rev}$	0.03	0.15
31	ΔE_{reac}	0.09	0.20
32	$\Delta E^{\#}_{fwd}$	0.18	0.33
32	$\Delta E^{\#}_{rev}$	-0.20	-0.67
32	ΔE_{reac}	0.38	1.00
33	$\Delta E^{\#}_{fwd}$	0.03	0.11
33	$\Delta E^{\#}_{rev}$	0.20	0.54
33	ΔE_{reac}	-0.17	-0.43
34	$\Delta E^{\#}_{fwd}$	0.10	0.25
34	$\Delta E^{\#}_{rev}$	0.09	0.37
34	ΔE_{reac}	0.01	-0.11
35	$\Delta E^{\#}_{fwd}$	0.06	0.12
35	$\Delta E^{\#}_{rev}$	0.09	0.06
35	ΔE_{reac}	-0.03	0.06

Table S6. Energy Differences (kcal/mol) for V{Q,5}Z(-PP) and AV{Q,5}Z(-PP) Extrapolations in Conventional MP2 for the Energetics of CUAGAU-2 dataset.

ID	ΔE MP2/ V{Q,5}Z(-PP)	ΔE MP2/ AV{Q,5}Z(-PP)	δΔE
Cu_2 (AE)	44.40	45.03	0.63
Ag_2 (AE)	42.48	42.88	0.41
Au_2 (AE)	58.41	58.59	0.18
CuH (AE)	61.58	61.67	0.09
AgH (AE)	51.83	51.99	0.16
AuH (AE)	70.19	70.36	0.17
CuF (AE)	108.35	108.42	0.07
AgF (AE)	87.61	87.74	0.13
AuF (AE)	72.48	72.64	0.16
CuO (AE)	44.73	45.08	0.35
AgO (AE)	20.98	21.17	0.20
AuO (AE)	45.24	45.50	0.25
Cu_3 (AE)	62.11	63.06	0.95
Ag_3 (AE)	61.59	62.16	0.57
Au_3 (AE)	87.64	87.72	0.08
Cu_4 (AE)	129.64	131.29	1.65
Ag_4 (AE)	121.32	121.87	0.55
Au_4 (AE)	164.65	164.40	-0.25
Cu_5 (AE)	172.08	173.93	1.85
Ag_5 (AE)	157.33	157.40	0.07
Au_5 (AE)	209.12	208.57	-0.55
Cu_6 (AE)	217.79	220.36	2.57
Ag_6 (AE)	199.72	199.91	0.19
Au_6 (AE)	267.63	266.93	-0.70
Cu_3_IE1	159.40	159.79	0.38
Ag_3_IE1	160.31	160.61	0.30
Au_3_IE1	202.60	202.86	0.27
Cu_4_2IE	403.34	404.22	0.88
Ag_4_2IE	397.99	398.76	0.76
Au_4_2IE	467.56	468.13	0.57
Cu_5_IE1	133.00	133.42	0.41
Ag_5_IE1	134.44	134.75	0.32
Au_5_IE1	163.97	164.26	0.29
Cu_5_IE2	266.32	266.72	0.41
Ag_5_IE2	255.69	255.83	0.15

Au_5_IE2	289.67	289.92	0.25
Cu_5_IE1	154.21	154.79	0.58
Ag_5_IE1	151.58	152.04	0.46
Au_5_IE1	179.77	180.14	0.38
Cu_5_IE2	262.16	262.45	0.30
Ag_5_IE2	253.98	254.12	0.14
Au_5_IE2	284.36	284.54	0.19
Cu_5_N0_I1	-7.10	-7.23	-0.14
Ag_5_N0_I1	-3.63	-3.42	0.21
Au_5_N0_I1	-4.05	-3.81	0.24
Cu_5_N0_I1	-9.84	-9.51	0.33
Ag_5_N0_I1	-5.87	-5.62	0.26
Au_5_N0_I1	-5.02	-5.00	0.02
Cu_6_N0_I1	-20.22	-19.69	0.53
Ag_6_N0_I1	-13.93	-13.40	0.53
Au_6_N0_I1	-11.07	-10.91	0.17
Cu_3_C1_I1	-41.81	-41.90	-0.09
Ag_3_C1_I1	-36.42	-36.22	0.20
Au_3_C1_I1	-46.96	-46.80	0.16
Cu_5_C1_I1	-29.25	-28.97	0.28
Ag_5_C1_I1	-24.75	-24.53	0.23
Au_5_C1_I1	-22.81	-22.80	0.01
Cu_6_C1_I1	-13.21	-12.67	0.54
Ag_6_C1_I1	-4.13	-3.55	0.59
Au_6_C1_I1	-2.65	-2.45	0.20
Cu_5_C2_I1	-19.19	-18.89	0.30
Ag_5_C2_I1	-16.36	-16.16	0.20
Au_5_C2_I1	-14.39	-14.36	0.03
Cu_6_C2_I1	11.13	11.57	0.43
Ag_6_C2_I1	-14.45	-13.81	0.64
Au_6_C2_I1	10.62	11.03	0.41
Cu_4H_I1	-13.54	-13.53	0.01
Ag_4H_I1	-4.99	-5.11	-0.12
Au_4H_I1	-0.91	-0.95	-0.03
Cu_4H_I2	-14.41	-14.07	0.34
Ag_4H_I2	-6.47	-6.52	-0.05
Au_4H_I2	10.65	10.52	-0.13
Cu_4O_I1	-163.77	-163.35	0.42
Ag_4O_I1	-66.72	-66.33	0.39
Au_4O_I1	-52.24	-52.27	-0.03

Cu_4O_I2	-139.40	-139.36	0.04
Ag_4O_I2	-99.01	-98.58	0.42
Au_4O_I2	-56.28	-56.33	-0.05
Cu_4NH_3_I1	35.64	35.55	-0.10
Ag_4NH_3_I1	27.34	27.43	0.10
Au_4NH_3_I1	47.14	47.44	0.29
Cu_4CO_I1	19.59	19.48	-0.11
Ag_4CO_I1	16.24	16.41	0.17
Au_4CO_I1	36.48	36.80	0.32
Cu_4CO_I2	29.63	29.56	-0.07
Ag_4CO_I2	20.84	20.99	0.15
Au_4CO_I2	41.98	42.38	0.40
Cu_4_H	9.73	9.72	-0.01
Ag_4_H	7.64	7.70	0.06
Au_4_H	27.24	27.49	0.25
Cu_5_H	59.22	59.18	-0.04
Ag_5_H	48.14	48.23	0.10
Au_5_H	56.47	56.77	0.30
Cu_6_H	73.75	73.41	-0.34
Ag_6_H	59.63	59.56	-0.07
Au_6_H	63.67	63.93	0.27
Cu_4_O	99.64	99.56	-0.08
Ag_4_O	44.95	44.87	-0.08
Au_4_O	35.06	35.56	0.50
Cu_5_O	74.01	73.72	-0.29
Ag_5_O	48.65	48.65	0.00
Au_5_O	25.64	25.96	0.32
Cu_6_O	124.23	124.12	-0.10
Ag_6_O	92.62	92.74	0.12
Au_6_O	52.71	53.05	0.33
Cu_4_NH3	65.07	65.19	0.13
Ag_4_NH3	52.11	52.24	0.13
Au_4_NH3	73.17	73.60	0.43
Cu_5_NH3	61.98	62.14	0.16
Ag_5_NH3	48.62	48.82	0.19
Au_5_NH3	68.70	69.14	0.44
Cu_6_NH3	57.93	58.17	0.24
Ag_6_NH3	49.52	49.76	0.24
Au_6_NH3	70.28	70.76	0.48
Cu_4_CO	40.10	40.11	0.01

Ag_4_CO	29.62	29.69	0.08
Au_4_CO	51.88	52.28	0.40
Cu_5_CO	40.66	40.67	0.01
Ag_5_CO	28.96	29.11	0.15
Au_5_CO	50.70	51.10	0.40
Cu_6_CO	43.86	43.75	-0.11
Ag_6_CO	30.93	31.10	0.17
Au_6_CO	50.70	51.12	0.43

Table S7. Basis Set Exponents (f and g) and Differences: Our VnZ(-PP)-F12 Basis Sets vs. Shaw and Hill's (SH's) VnZ-PP-F12 Basis Sets (n=D,T).

	our cc-pVDZ-F12		SH's cc-pVDZ-PP-F12		Absolute Differences	
	f_1	f_2	f_1	f_2	Δf_1	Δf_2
Cu	0.872	6.006	0.848	6.107	0.024	0.101
Zn	0.978	6.828	0.967	6.905	0.010	0.077
					MAD (all f)	0.053

	our cc-pVTZ-PP-F12		SH's cc-pVDZ-PP-F12		Absolute Differences	
	f_1	f_2	f_1	f_2	Δf_1	Δf_2
Ag	0.608	2.472	0.608	2.471	0.001	0.001
Cd	0.718	3.034	0.717	3.032	0.000	0.002
Au	0.377	1.151	0.376	1.149	0.001	0.003
Hg	0.418	1.265	0.418	1.264	0.000	0.000
					MAD (all f)	0.001

	our cc-pVTZ-F12					SH's cc-pVTZ-PP-F12					Absolute Differences				
	f_1	f_2	f_3	g_1	g_2	f_1	f_2	f_3	g_1	g_2	Δf_1	Δf_2	Δf_2	Δg_1	Δg_2
Cu	0.616	2.406	9.327	0.808	5.138	0.619	2.427	9.610	0.752	5.358	0.003	0.021	0.283	0.056	0.220
Zn	0.743	3.056	11.259	0.821	5.631	0.751	3.182	11.843	0.932	5.952	0.007	0.127	0.584	0.111	0.321
														MAD (all f and g)	0.173

	our cc-pVTZ-PP-F12					SH's cc-pVTZ-PP-F12					Absolute Differences				
	f_1	f_2	f_3	g_1	g_2	f_1	f_2	f_3	g_1	g_2	Δf_1	Δf_2	Δf_3	Δg_1	Δg_2
Ag	0.464	1.544	6.701	0.587	2.087	0.465	1.547	6.723	0.585	2.096	0.000	0.003	0.022	0.002	0.009
Cd	0.530	1.769	7.615	0.648	2.471	0.533	1.781	7.686	0.639	2.469	0.002	0.012	0.071	0.010	0.002
Au	0.269	0.704	1.808	0.478	1.445	0.265	0.694	1.780	0.487	1.557	0.005	0.010	0.028	0.009	0.112
Hg	0.297	0.778	2.031	0.546	1.829	0.297	0.776	2.026	0.544	1.820	0.001	0.001	0.004	0.002	0.008
														MAD (all f and g)	0.016

Table S8. Total Energies of Neutral Atom States using our V*n*Z(-PP)-F12(*d*) Basis Sets (*n*=D,T) and Shaw and Hill's (SH's) V*n*Z-PP-F12(*d*) Basis Sets with up to *d* Functions Throughout.

		Basis	VDZ(-PP)-F12(*d*) (this work)	VDZ-PP-F12(*d*) (SH's)[a]	(this work) – (SH's)[b]	VTZ(-PP)-F12(*d*) (this work)	VTZ-PP-F12(*d*) (SH's)[a]	(this work) – (SH's)[b]
State		M	MP2-F12 (E_h)	MP2-F12 (E_h)	$\Delta E_{tot,MP2-F12}$ (μE_h)	MP2-F12 (E_h)	MP2-F12 (E_h)	$\Delta E_{tot,MP2-F12}$ (μE_h)
s^2d^9	^2D	Cu	-1639.393006	-196.631087	N/A	-1639.394112	-196.632475	N/A
s^1d^{10}	^2S		-1639.496630	-196.719716	N/A	-1639.497593	-196.720892	N/A
s^2d^{10}	^1S	Zn	-1778.387594	-226.516824	N/A	-1778.388556	-226.518083	N/A
s^2d^9	^2D	Ag	-146.257441	-146.257431	-9.8 (-10)	-146.258075	-146.258072	-3.0 (-2.1)
s^1d^{10}	^2S		-146.418689	-146.418679	-10.3 (-11.2)	-146.419175	-146.419165	-10.0 (-9.7)
s^2d^{10}	^1S	Cd	-167.261002	-167.260950	-51.4 (-50.7)	-167.261605	-167.261599	-5.3 (-5.0)
s^2d^9	^2D	Au	-135.034828	-135.034810	-17.7 (-18.7)	-135.035267	-135.035258	-8.7 (-8.4)
s^1d^{10}	^2S		-135.111366	-135.111361	-5.8 (-7.0)	-135.111678	-135.111667	-11.5 (-11.5)
s^2d^{10}	^1S	Hg	-152.931612	-152.931579	-33.3 (-33.7)	-152.932098	-152.932085	-13.8 (-13.8)

[a] In SH's VnZ-PP-F12 basis sets, an ECP is used for the core electrons of copper and zinc atoms.

[b] Values in parentheses indicate differences in MP2-F12 correlation energies.

Table S9. Multireference Character Diagnostics of *d*-Block Elements Obtained Using our cc-pVDZ(-PP)-F12-wis Basis Set in CCSD(T)-F12.

State		M	T_1	D_1	D_2
s^2d^1	^2D	Sc	0.025	0.038	0.298
s^1d^2	^4F		0.016	0.016	0.000
d^3	^4F		0.053	0.053	0.000
s^2d^2	^3F	Ti	0.024	0.044	0.283
s^1d^3	^5F		0.020	0.021	0.000
d^4	^5D		0.010	0.014	0.000
s^2d^3	^4F	V	0.025	0.050	0.275
s^1d^4	^6D		0.020	0.026	0.000
d^5	^6S		0.010	0.013	0.000
s^2d^4	^5D	Cr	0.024	0.024	0.268
s^1d^5	^7S		0.018	0.028	0.000
d^6	^5D		0.012	0.014	0.237
s^2d^5	^6S	Mn	0.023	0.056	0.257
s^1d^6	^6D		0.025	0.041	0.168
d^7	^4F		0.015	0.029	0.198
s^2d^6	^5D	Fe	0.023	0.060	0.246
s^1d^7	^5F		0.023	0.046	0.136
d^8	^3F		0.036	0.054	0.142
s^2d^7	^4F	Co	0.023	0.062	0.238
s^1d^8	^4F		0.023	0.051	0.119
d^9	^2D		0.036	0.067	0.136
s^2d^8	^3F	Ni	0.022	0.063	0.231
s^1d^9	^3D		0.025	0.054	0.109
d^{10}	^1S		0.028	0.039	0.106
s^2d^9	^2D	Cu	0.021	0.064	0.225
s^1d^{10}	^2S		0.022	0.058	0.094
s^2d^{10}	^1S	Zn	0.020	0.065	0.220

s^2d^1	^2D	Y	0.026	0.038	0.313
s^1d^2	^4F		0.007	0.009	0.000
d^3	^4F		0.019	0.019	0.000
s^2d^2	^3F	Zr	0.025	0.045	0.298
s^1d^3	^5F		0.010	0.015	0.000
d^4	^5D		0.012	0.024	0.000
s^2d^3	^4F	Nb	0.025	0.052	0.293
s^1d^4	^6D		0.010	0.020	0.000
d^5	^6S		0.011	0.024	0.000
s^2d^4	^5D	Mo	0.030	0.058	0.285
s^1d^5	^7S		0.011	0.025	0.000
d^6	^5D		0.027	0.065	0.228
s^2d^5	^6S	Tc	0.023	0.058	0.252
s^1d^6	^6D		0.014	0.033	0.161
d^7	^4F		0.014	0.033	0.204
s^2d^6	^5D	Ru	0.023	0.062	0.237
s^1d^7	^5F		0.015	0.042	0.137
d^8	^3F		0.004	0.006	0.135
s^2d^7	^4F	Rh	0.022	0.066	0.226
s^1d^8	^4F		0.017	0.050	0.122
d^9	^2D		0.043	0.130	0.141
s^2d^8	^3F	Pd	0.022	0.069	0.218
s^1d^9	^3D		0.018	0.056	0.111
d^{10}	^1S		0.002	0.002	0.101
s^2d^9	^2D	Ag	0.021	0.071	0.210
s^1d^{10}	^2S		0.019	0.062	0.100
s^2d^{10}	^1S	Cd	0.020	0.069	0.204

s^2d^2	^3F	Hf	0.021	0.037	0.294
s^1d^3	^5F		0.009	0.009	0.000
d^4	^5D		0.015	0.030	0.000
s^2d^3	^4F	Ta	0.021	0.041	0.290
s^1d^4	^6D		0.008	0.012	0.000
d^5	^6S		0.015	0.032	0.000
s^2d^4	^5D	W	0.020	0.045	0.282
s^1d^5	^7S		0.008	0.015	0.000
d^6	^5D		0.018	0.042	0.000
s^2d^5	^6S	Re	0.018	0.045	0.246
s^1d^6	^6D		0.011	0.021	0.179
d^7	^4F		0.010	0.023	0.000
s^2d^6	^5D	Os	0.018	0.049	0.227
s^1d^7	^5F		0.011	0.028	0.152
d^8	^3F		0.003	0.004	0.222
s^2d^7	^4F	Ir	0.019	0.054	0.214
s^1d^8	^4F		0.012	0.036	0.134
d^9	^2D		0.007	0.020	0.000
s^2d^8	^3F	Pt	0.018	0.056	0.202
s^1d^9	^3D		0.013	0.041	0.122
d^{10}	^1S		0.001	0.001	0.114
s^2d^9	^2D	Au	0.015	0.049	0.109
s^1d^{10}	^2S		0.018	0.059	0.192
s^2d^{10}	^1S	Hg	0.017	0.060	0.183

Table S10. HF basis set limit estimates for the polyoxometalate $Mo_6O_{19}^{-2}$.

The CABS singles correction of $Mo_6O_{19}^{-2}$ was obtained from DLPNO-CCSD(T_1)-F12 calculations in ORCA.

Orbital Basis set			ORCA					
		CABS for Mo	autoCABS generated from OBS of Mo atom			def2-QZVPP/JKFit		
N_{bas}^{a}	O atom	Mo atom	HF (E_h)	HF CABS Singles (E_h)	HF + CABS (E_h)	HF (E_h)	HF CABS Singles (E_h)	HF + CABS (E_h)
990	VDZ-F12	AVTZ-PP(*f*)	-1827.438262	-4.408367	-1831.846629	-1827.438261	Err	Err
948	VDZ-F12	AVTZ-PP(*f*-1)	-1827.435059	-3.006465	-1830.441525	-1827.435059	Err	Err
948	VDZ-F12	VDZ-PP-F12-wis	-1827.426983	-2.569135	-1829.996118	-1827.426983	Err	Err
1589	VTZ-F12	VTZ-PP-F12-wis	-1827.600559	-0.375188	-1827.975747	-1827.600559	Err	Err

Orbital Basis set			MOLPRO					
		CABS for Mo	autoCABS generated from OBS of Mo atom			def2-QZVPP/JKFit		
N_{bas}^{a}	O atom	Mo atom	HF (E_h)	HF CABS Singles (E_h)	HF + CABS (E_h)	HF (E_h)	HF CABS Singles (E_h)	HF + CABS (E_h)
990	VDZ-F12	AVTZ-PP(*f*)	-1827.437715	-0.093990	-1827.531704	-1827.437715	-0.082160	-1827.519875
948	VDZ-F12	AVTZ-PP(*f*-1)	-1827.434513	-0.092930	-1827.527443	-1827.434513	-0.084384	-1827.518897
948	VDZ-F12	VDZ-PP-F12-wis	-1827.426435	-0.100584	-1827.527020	-1827.426435	-0.091418	-1827.517854
1589	VTZ-F12	VTZ-PP-F12-wis	-1827.599923	-0.018421	-1827.618344	-1827.599923	-0.015467	-1827.615390

Establishing the HF basis set limit from DF-HF calculations for the polyoxometalate leads to:

Orbital basis set			MOLPRO
N_{bas}^{a}	O atom	Mo atom	HF (E_h)
761	AVDZ	AVDZ-PP	-1827.107742
1402	AVTZ	AVTZ-PP	-1827.512953
2330	AVQZ	AVQZ-PP	-1827.611363
3595	AV5Z	AV5Z-PP	-1827.635537
	AV{T,Q}Z(-PP)		-1827.627062
	AV{Q,5}Z(-PP)		-1827.638189

The Karton-Martin extrapolation formulas [Theor. Chem. Acc. 115 (2006) 330-333] of exponential form are used with $\gamma = 6.57$ and 9.03 for AV{T,Q}Z and AV{Q,5}Z, respectively.

Table S11. HF basis set limit estimates for group-17 diatomic molecules

	Orbital Basis set	ORCA			MOLPRO			
	Basis Set	HF (E_h)	HF CABS Singles (E_h)	HF + CABS (E_h)	HF (E_h)	HF CABS Singles (E_h)	HF + CABS (E_h)	δE (mE_h) (HF CABS ORCA – MOLPRO)
	VDZ-F12	-918.993842	-0.009221	-919.003063	-918.993456	-0.009535	-919.002991	0.314
Cl_2	VTZ-F12	-919.008635	-0.000678	-919.009312	-919.008532	-0.000947	-919.009479	0.269
	VQZ-F12	-919.010413	-0.000018	-919.010431	-919.010359	-0.000131	-919.010490	0.113
	VDZ-PP-F12	-831.000826	-0.073938	-831.074765	-831.000761	-0.007399	-831.008160	-66.539
Br_2	VTZ-PP-F12	-831.010201	-0.209518	-831.219719	-831.010130	-0.000624	-831.010754	-208.894
	VQZ-PP-F12	-831.010906	-0.030519	-831.041425	-831.011015	-0.000101	-831.011115	-30.418
	VDZ-PP-F12	-589.338768	-0.014144	-589.352912	-589.338604	-0.008299	-589.346903	-5.845
I_2	VTZ-PP-F12	-589.347207	-0.006575	-589.353783	-589.347031	-0.001023	-589.348054	-5.552
	VQZ-PP-F12	-589.348607	-0.004600	-589.353208	-589.348424	-0.000257	-589.348681	-4.34

	HF (E_h)		
Basis set	Cl_2	Br_2	I_2
AVDZ(-PP)	-918.965702	-830.965463	-589.321806
AVTZ(-PP)	-918.999989	-831.006165	-589.344784
AVQZ(-PP)	-919.007679	-831.010328	-589.347396
AV5Z(-PP)	-919.010088	-831.010843	-589.347844
AV{T,Q}Z(-PP)	-919.008906	-831.010992	-589.347813
AV{Q,5}Z(-PP)	-919.010352	-831.010900	-589.347893

Geometries:

Cl_2

Cl	0.0000	0.0000	0.0000
Cl	0.0000	0.0000	1.9879

Br_2

Br	0.0000	0.0000	1.1405
Br	0.0000	0.0000	-1.1405

I_2

I	0.0000	0.0000	0.0000
I	0.0000	0.0000	2.6655

Table S12. HF basis set limit estimates for MoO_3^{-2}

Orbital Basis set			ORCA					
		CABS for Mo	autoCABS generated from OBS of Mo atom			def2-QZVPP/JKFit		
N_{bas}^{a}	O atom	Mo atom	HF (E_h)	HF CABS Singles (E_h)	HF + CABS (E_h)	HF (E_h)	HF CABS Singles (E_h)	HF + CABS (E_h)
160	VDZ-F12	AVTZ-PP(*f*)	-291.909263	-0.387546	-292.296809	-291.909263	-0.114272	-292.023535
153	VDZ-F12	AVTZ-PP(*f*-1)	-291.909107	-0.305687	-292.214794	-291.909107	-0.118153	-292.027260
153	VDZ-F12	VDZ-PP-F12-wis	-291.908036	-0.309711	-292.217747	-291.908036	-0.116840	-292.024876
256	VTZ-F12	VTZ-PP-F12-wis	-291.935753	-0.045919	-291.981672	-291.935753	-0.092078	-292.027831
Orbital Basis set			MOLPRO					
		CABS for Mo	autoCABS generated from OBS of Mo atom			def2-QZVPP/JKFit		
N_{bas}^{a}	O atom	Mo atom	HF (E_h)	HF CABS Singles (E_h)	HF + CABS (E_h)	HF (E_h)	HF CABS Singles (E_h)	HF + CABS (E_h)
160	VDZ-F12	AVTZ-PP(*f*)	-291.910066	-0.019950	-291.930016	-291.910066	-0.013244	-291.923311
153	VDZ-F12	AVTZ-PP(*f*-1)	-291.909912	-0.019629	-291.929541	-291.909912	-0.013318	-291.923230
153	VDZ-F12	VDZ-PP-F12-wis	-291.908837	-0.020731	-291.929569	-291.908837	-0.014422	-291.923259
256	VTZ-F12	VTZ-PP-F12-wis	-291.936929	-0.009432	-291.946361	-291.936929	-0.003005	-291.939934

N_{bas}^{a}	O atom	Mo atom	HF (E_h)
123	AVDZ	AVDZ-PP	-291.854654
226	AVTZ	AVTZ-PP	-291.921210
375	AVQZ	AVQZ-PP	-291.938702
578	AV5Z	AV5Z-PP	-291.946069
		AV{T,Q}Z(-PP)	-291.941492
		AV{Q,5}Z(-PP)	-291.946877

Geometry:

Mo	-0.000024	-0.000000	-0.196525
O	0.759300	-1.429074	0.343905
O	0.858078	1.372047	0.343903
O	-1.617250	0.057028	0.343946

Table S13. HF basis set limit estimates for CrO_3^{-2}

Orbital Basis set			ORCA					
		CABS for Cr	autoCABS generated from OBS of Cr atom			def2-QZVPP/JKFit		
N_{bas}^{a}	O atom	Cr atom	HF (E_h)	HF CABS Singles (E_h)	HF + CABS (E_h)	HF (E_h)	HF CABS Singles (E_h)	HF + CABS (E_h)
165	VDZ-F12	AVTZ(*f*)	-1267.699786	-0.002114	-1267.701332	-1267.699786	-0.005237	-1267.705023
158	VDZ-F12	AVTZ(*f*-1)	-1267.699218	-0.002941	-1267.702727	-1267.699218	-0.005723	-1267.704942
158	VDZ-F12	VDZ-F12-wis	-1267.698940	-0.002907	-1267.701847	-1267.698940	-0.006091	-1267.705030
261	VTZ-F12	VTZ-F12-wis	-1267.718154	-0.000184	-1267.718338	-1267.718154	-0.000890	-1267.719044

Orbital Basis set			MOLPRO					
		CABS for Cr	autoCABS generated from OBS of Cr atom			def2-QZVPP/JKFit		
N_{bas}^{a}	O atom	Cr atom	HF (E_h)	HF CABS Singles (E_h)	HF + CABS (E_h)	HF (E_h)	HF CABS Singles (E_h)	HF + CABS (E_h)
156	VDZ-F12	AVTZ(*f*)	-1267.699671	-0.006852	-1267.706483	-1267.699671	-0.007347	-1267.706942
158	VDZ-F12	AVTZ(*f*-1)	-1267.699102	-0.006812	-1267.705953	-1267.699102	-0.007841	-1267.707018
158	VDZ-F12	VDZ-F12-wis	-1267.698829	-0.006966	-1267.705795	-1267.698829	-0.007801	-1267.706630
261	VTZ-F12	VTZ-F12-wis	-1267.718014	-0.001611	-1267.719625	-1267.718014	-0.001668	-1267.719682

Orbital basis set			MOLPRO
N_{bas}^{a}	O atom	Cr atom	HF (E_h)
128	AVDZ	AVDZ	-1267.649085
231	AVTZ	AVTZ	-1267.704720
380	AVQZ	AVQZ	-1267.718980
583	AV5Z	AV5Z	-1267.722600
AV{T,Q}Z			-1267.721255
AV{Q,5}Z			-1267.722997

Geometry:

Cr	-0.000024	-0.000000	-0.196525
O	0.759300	-1.429074	0.343905
O	0.858078	1.372047	0.343903
O	-1.617250	0.057028	0.343946

Table S14. Basis sets functions for selected geminal exponents β used during optimization.

	Optimized exponents of our VDZ(-PP)-F12-wis basis sets for group 11 and 12 elements						Shaw and Hill's optimized exponents of VDZ-PP-F12	
	$\beta = 1.0$			$\beta = 1.4$			$\beta = 1.4$	
	f_1	f_2	f_3	f_1	f_2	f_3	f_1	f_2
Cu	2.514	9.540	-	0.872	6.006	-	0.848	6.107
	0.634	3.017	10.987	0.619	2.340	9.331		
Ag	0.492	2.180	-	0.608	2.472	-	0.608	2.471
Au	0.376	1.224	-	0.377	1.151	-	0.376	1.149
Zn	2.751	10.469	-	0.978	6.828	-	0.967	6.905
	0.798	3.443	12.435	0.746	3.075	11.352		
Cd	0.552	2.460	-	0.718	3.035	-	0.717	3.032
Hg	0.422	1.387	-	0.418	1.265	-	0.418	1.264

An argon core is kept frozen for 3d-block elements in MP2-F12 calculations

	Common logarithms of optimized exponents of our VDZ(-PP)-F12-wis basis sets for group 11 and 12 elements						Shaw and Hill's optimized exponents ($\log_{10} a$) of VDZ-PP-F12	
	$\beta = 1.0$			$\beta = 1.4$			$\beta = 1.4$	
	f_1	f_2	f_3	f_1	f_2	f_3	f_1	f_2
Cu	0.400	0.980	-	-0.060	0.779	-	-0.072	0.786
	-0.198	0.480	1.041	-0.208	0.369	0.970		
Ag	-0.308	0.338	-	-0.216	0.393	-	-0.216	0.393
Au	-0.425	0.088	-	-0.424	0.061	-	-0.425	0.060
Zn	0.439	1.020	-	-0.010	0.834	-	-0.015	0.839
	-0.098	0.537	1.095	-0.128	0.488	1.055		
Cd	-0.258	0.391	-	-0.144	0.482	-	-0.144	0.482
Hg	-0.375	0.142	-	-0.379	0.102	-	-0.379	0.102

The cc-pVnZ-F12-wis and cc-pVnZ-PP-F12-wis orbital basis sets (where n=D and T) are provided in MOLPRO format. The *s*, *p*, and *d* exponents are obtained from aug-cc-pV(n+1)Z and aug-cc-pV(n+1)Z-PP basis sets. When using the cc-pVnZ-PP-F12-wis basis sets, the Stuttgart-Köln pseudopotentials ECP28MDF and ECP60MDF must be utilized for atoms Y-Cd and Hf-Hg, respectively. (see below)

cc-pVDZ-F12-wis Basis Set: Sc-Zn Atoms

```
! scandium            (21s,17p,9d,2f) -> [8s,7p,5d,2f]
s, SC , 2.715278E+06, 4.065984E+05, 9.253004E+04, 2.620792E+04, 8.549429E+03, 3.085975E+03, 1.203172E+03, 4.984869E+02, 2.167360E+02, 9.787476E+01, 4.520433E+01,
        2.021187E+01, 9.574751E+00, 4.540346E+00, 1.995687E+00, 9.422150E-01, 4.178450E-01, 9.576100E-02, 5.135100E-02, 2.387800E-02, 1.110000E-02
c, 1.20, 8.147221E-06, 6.334788E-05, 3.330384E-04, 1.404055E-03, 5.081725E-03, 1.626926E-02, 4.624577E-02, 1.137223E-01, 2.257636E-01, 3.106700E-01, 2.191906E-01,
        7.215879E-02, 1.187030E-01, 1.220532E-01, 2.136795E-02, -5.357246E-04, 2.435774E-04, -8.796617E-05, 7.878246E-05, -1.637155E-05
c, 1.20, -4.722109E-06, -3.671829E-05, -1.930883E-04, -8.146870E-04, -2.955526E-03, -9.520035E-03, -2.746858E-02, -6.991528E-02, -1.499251E-01, -2.459153E-01,
        -2.401293E-01, 3.567987E-02, 4.915023E-01, 4.911381E-01, 9.120633E-02, -5.356723E-03, 8.812836E-04, -7.605536E-04, 6.340116E-04, -1.556163E-04
c, 1.20, 9.139905E-07, 7.108513E-06, 3.738126E-05, 1.578828E-04, 5.737686E-04, 1.859244E-03, 5.433182E-03, 1.425387E-02, 3.246144E-02, 6.003454E-02, 6.916105E-02,
        -2.113084E-02, -2.666832E-01, -4.367591E-01, 6.498243E-02, 7.009599E-01, 4.515562E-01, 3.011910E-02, -1.329480E-02, 4.633679E-03
c, 1.20, -2.201951E-07, -1.711419E-06, -9.008469E-06, -3.799997E-05, -1.383227E-04, -4.473692E-04, -1.310691E-03, -3.429860E-03, -7.847579E-03, -1.447189E-02,
        -1.690669E-02, 5.396115E-03, 6.671062E-02, 1.178356E-01, -2.738134E-02, -2.260149E-01, -3.073539E-01, 2.544054E-01, 5.981590E-01, 3.115202E-01
c, 1.20, -3.757238E-07, -2.981907E-06, -1.522586E-05, -6.684686E-05, -2.313129E-04, -7.959729E-04, -2.161961E-03, -6.206459E-03, -1.261905E-02, -2.739459E-02,
        -2.336516E-02, -5.734627E-03, 1.536025E-01, 1.447100E-01, 9.359699E-02, -8.687730E-01, 2.114597E-02, 2.275498E+00, -1.190770E+00, -7.674257E-01
c, 20.20, 1.000000E+00
c, 1.20, -5.962768E-07, -4.822853E-06, -2.395005E-05, -1.090335E-04, -3.601944E-04, -1.311233E-03, -3.322435E-03, -1.037520E-02, -1.892174E-02, -4.755287E-02,
        -2.931692E-02, -3.090088E-02, 3.074597E-01, 1.803100E-01, 2.358178E-01, -2.388544E+00, 2.329407E+00, 9.918252E-01, -3.507240E+00, 2.262804E+00
c, 21.21, 1.000000E+00
p, SC , 1.059219E+04, 2.507533E+03, 8.144571E+02, 3.115195E+02, 1.319617E+02, 5.998718E+01, 2.866250E+01, 1.410851E+01, 7.103706E+00, 3.609200E+00,
        1.776070E+00, 8.547600E-01, 4.022390E-01, 1.546650E-01, 6.494500E-02, 2.635900E-02, 1.066000E-02
c, 1.16, 4.500000E-05, 4.010000E-04, 2.302000E-03, 1.003700E-02, 3.495400E-02, 9.790900E-02, 2.106800E-01, 3.300930E-01, 3.310270E-01, 1.579600E-01, 2.209900E-02,
        -1.605000E-03, -1.326000E-03, -2.800000E-04, 3.400000E-05, -1.300000E-05
c, 1.16, -1.500000E-05, -1.310000E-04, -7.570000E-04, -3.318000E-03, -1.170600E-02, -3.360400E-02, -7.487900E-02, -1.225480E-01, -1.302760E-01, 1.459600E-02,
        3.091840E-01, 4.629980E-01, 3.049570E-01, 5.087800E-02, -4.493000E-03, 1.832000E-03
c, 1.16, 4.000000E-06, 3.900000E-05, 2.210000E-04, 9.840000E-04, 3.423000E-03, 9.993000E-03, 2.191600E-02, 3.700800E-02, 3.779400E-02, -4.379000E-03, -1.101640E-01,
        -1.610170E-01, -1.824820E-01, 3.886110E-01, 6.911000E-01, 7.960400E-02
c, 1.16, 9.000000E-06, 7.400000E-05, 4.790000E-04, 1.869000E-03, 7.424000E-03, 1.876300E-02, 4.871700E-02, 6.544000E-02, 1.079480E-01, -5.871300E-02, -1.703060E-01,
        -7.549770E-01, 6.201170E-01, 1.167548E+00, -1.009531E+00, -1.889910E-01
c, 1.16, -4.000000E-06, -3.200000E-05, -1.850000E-04, -8.080000E-04, -2.870000E-03, -8.207000E-03, -1.847300E-02, -3.010100E-02, -3.294300E-02, 7.958000E-03,
        8.799300E-02, 1.523770E-01, 9.717000E-02, -2.569380E-01, -5.878150E-01, -3.054210E-01
c, 16.16, 1.000000E+00
c, 17.17, 1.000000E+00
d, SC , 5.051380E+01, 1.474050E+01, 5.195000E+00, 2.028460E+00, 8.040860E-01, 3.076890E-01, 1.113920E-01, 3.735200E-02, 1.244000E-02
c, 1.8, 4.266000E-03, 2.770800E-02, 1.000010E-01, 2.315810E-01, 3.460330E-01, 3.733740E-01, 2.642880E-01, 6.366700E-02
c, 1.8, -4.389000E-03, -2.836300E-02, -1.051370E-01, -2.348540E-01, -3.246090E-01, -6.428900E-02, 6.017490E-01, 3.903000E-01
c, 1.8, 5.859000E-03, 3.732300E-02, 1.419240E-01, 3.068960E-01, 3.081890E-01, -7.607170E-01, -2.047750E-01, 7.892710E-01
c, 8.8, 1.000000E+00
c, 9.9, 1.000000E+00
f, SC , 7.378872394519E-01, 1.716208307926E-01
c, 1.1, 1.000000E+00
c, 2.2, 1.000000E+00
```

! titanium (21s,17p,9d,2f) -> [8s,7p,5d,2f]
s, TI , 3.014643E+06, 4.514329E+05, 1.027338E+05, 2.909817E+04, 9.492330E+03, 3.426346E+03, 1.335896E+03, 5.535026E+02, 2.406925E+02, 1.087293E+02, 5.026457E+01,
2.258004E+01, 1.071432E+01, 5.093546E+00, 2.244183E+00, 1.059570E+00, 4.688490E-01, 1.061430E-01, 5.526200E-02, 2.546500E-02, 0.0117300
c, 1.20, 8.060782E-06, 6.267518E-05, 3.295006E-04, 1.389203E-03, 5.028469E-03, 1.610419E-02, 4.581232E-02, 1.128613E-01, 2.248193E-01, 3.114571E-01, 2.224995E-01,
7.293128E-02, 1.160683E-01, 1.194774E-01, 2.097868E-02, -5.091715E-04, 2.217859E-04, -7.636896E-05, 7.719539E-05, -1.149056E-05
c, 1.20, -4.630486E-06, -3.600451E-05, -1.893420E-04, -7.988781E-04, -2.898698E-03, -9.339701E-03, -2.697464E-02, -6.878913E-02, -1.481037E-01, -2.445253E-01,
-2.419916E-01, 3.183790E-02, 4.932686E-01, 4.939655E-01, 9.196313E-02, -5.316992E-03, 8.085624E-04, -6.918459E-04, 6.086512E-04, -1.313842E-04
c, 1.20, 9.230559E-07, 7.178974E-06, 3.775134E-05, 1.594532E-04, 5.795150E-04, 1.878414E-03, 5.492747E-03, 1.443297E-02, 3.296408E-02, 6.125493E-02, 7.134113E-02,
-1.973150E-02, -2.741869E-01, -4.440977E-01, 7.776084E-02, 7.068444E-01, 4.413892E-01, 2.799769E-02, -1.210790E-02, 4.324762E-03
c, 1.20, -2.180323E-07, -1.694860E-06, -8.919208E-06, -3.763633E-05, -1.369575E-04, -4.432894E-04, -1.298868E-03, -3.406752E-03, -7.810829E-03, -1.449245E-02,
-1.708136E-02, 4.897666E-03, 6.753108E-02, 1.173318E-01, -2.985025E-02, -2.277634E-01, -2.928115E-01, 2.665300E-01, 5.912406E-01, 3.037229E-01
c, 1.20, -3.975126E-07, -3.161080E-06, -1.609375E-05, -7.092947E-05, -2.442710E-04, -8.457892E-04, -2.282208E-03, -6.619873E-03, -1.335024E-02, -2.955830E-02,
-2.477039E-02, -8.414624E-03, 1.693855E-01, 1.500787E-01, 9.787777E-02, -9.653608E-01, 1.489721E-01, 2.191179E+00, -1.243325E+00, -6.711916E-01
c, 20.20, 1.000000E+00
c, 1.20, -6.302483E-07, -5.084585E-06, -2.534482E-05, -1.148260E-04, -3.817497E-04, -1.379645E-03, -3.531666E-03, -1.092225E-02, -2.029554E-02, -5.024404E-02,
-3.316309E-02, -3.098063E-02, 3.244673E-01, 2.155689E-01, 1.499616E-01, -2.420741E+00, 2.530874E+00, 3.423600E-01, -2.733722E+00, 2.006268E+00
c, 21.21, 1.0000000
p, TI , 1.191203E+04, 2.819947E+03, 9.159479E+02, 3.503842E+02, 1.484825E+02, 6.753944E+01, 3.230332E+01, 1.592786E+01, 8.038035E+00, 4.093916E+00, 2.022390E+00,
9.761020E-01, 4.595950E-01, 1.771520E-01, 7.351700E-02, 2.940100E-02, 0.0117600
c, 1.16, 4.400000E-05, 3.910000E-04, 2.248000E-03, 9.823000E-03, 3.433800E-02, 9.666600E-02, 2.094170E-01, 3.301890E-01, 3.319360E-01, 1.584880E-01, 2.231000E-02,
-1.566000E-03, -1.324000E-03, -2.710000E-04, 3.200000E-05, -1.200000E-05
c, 1.16, -1.500000E-05, -1.310000E-04, -7.550000E-04, -3.319000E-03, -1.175000E-02, -3.392200E-02, -7.616400E-02, -1.257020E-01, -1.330980E-01, 1.740600E-02,
3.151650E-01, 4.618140E-01, 2.998560E-01, 5.000000E-02, -4.230000E-03, 1.725000E-03
c, 1.16, 4.000000E-06, 3.900000E-05, 2.230000E-04, 9.920000E-04, 3.476000E-03, 1.017200E-02, 2.257600E-02, 3.823800E-02, 3.933700E-02, -6.106000E-03, -1.129620E-01,
-1.681140E-01, -1.659320E-01, 3.914030E-01, 6.818400E-01, 8.403100E-02
c, 1.16, 9.000000E-06, 7.300000E-05, 4.680000E-04, 1.856000E-03, 7.293000E-03, 1.885600E-02, 4.840500E-02, 6.737800E-02, 1.045410E-01, -5.376000E-02, -1.917490E-01,
-6.947200E-01, 5.601620E-01, 1.181336E+00, -9.932720E-01, -2.096810E-01
c, 1.16, 4.000000E-06, 3.100000E-05, 1.820000E-04, 7.950000E-04, 2.833000E-03, 8.154000E-03, 1.847200E-02, 3.040000E-02, 3.304700E-02, -8.251000E-03, -8.855400E-02,
-1.496120E-01, -9.422700E-02, 2.508460E-01, 5.866430E-01, 3.135350E-01
c, 16.16, 1.000000E+00
c, 17.17, 1.0000000
d, TI , 6.401300E+01, 1.881790E+01, 6.728700E+00, 2.664130E+00, 1.078680E+00, 4.232090E-01, 1.559990E-01, 5.188400E-02, 0.0172600
c, 1.8, 3.887000E-03, 2.639900E-02, 9.751100E-02, 2.328480E-01, 3.531520E-01, 3.721860E-01, 2.476720E-01, 5.823600E-02
c, 1.8, -3.970000E-03, -2.687300E-02, -1.022750E-01, -2.377280E-01, -3.121140E-01, -4.237800E-02, 5.886580E-01, 4.103020E-01
c, 1.8, 6.418000E-03, 4.380900E-02, 1.723250E-01, 3.753320E-01, 2.249470E-01, -7.919070E-01, -1.044280E-01, 7.135420E-01
c, 8.8, 1.000000E+00
c, 9.9, 1.0000000
f, TI , 1.082455371778E+00, 2.726026039632E-01
c, 1.1, 1.000000E+00
c, 2.2, 1.000000E+00

```
! vanadium           (21s,17p,9d,2f) -> [8s,7p,5d,2f]
s, V , 3.321857E+06, 4.974356E+05, 1.132027E+05, 3.206333E+04, 1.045962E+04, 3.775506E+03, 1.472040E+03, 6.099331E+02, 2.652634E+02, 1.198607E+02, 5.544891E+01,
       2.498372E+01, 1.188056E+01, 5.660311E+00, 2.495703E+00, 1.177866E+00, 5.200440E-01, 1.159650E-01, 5.893800E-02, 2.694600E-02, 0.0123200
c, 1.20, 8.039999E-06, 6.251402E-05, 3.286553E-04, 1.385697E-03, 5.016217E-03, 1.606931E-02, 4.574242E-02, 1.128544E-01, 2.254344E-01, 3.140461E-01, 2.267819E-01,
       7.334069E-02, 1.102474E-01, 1.131358E-01, 1.971295E-02, -4.719088E-04, 1.861606E-04, -6.208598E-05, 7.295314E-05, -6.362062E-06
c, 1.20, -4.503003E-06, -3.501295E-05, -1.841339E-04, -7.769216E-04, -2.819505E-03, -9.087486E-03, -2.627134E-02, -6.712726E-02, -1.451130E-01, -2.412483E-01,
       -2.416314E-01, 3.067362E-02, 4.970415E-01, 4.958875E-01, 9.181868E-02, -5.392514E-03, 7.102380E-04, -6.363128E-04, 5.979932E-04, -1.100879E-04
c, 1.20, 9.320648E-07, 7.249306E-06, 3.811967E-05, 1.610238E-04, 5.852210E-04, 1.897502E-03, 5.550909E-03, 1.460584E-02, 3.342974E-02, 6.235722E-02, 7.312435E-02,
       -1.911472E-02, -2.817249E-01, -4.488151E-01, 9.202696E-02, 7.110117E-01, 4.309274E-01, 2.604589E-02, -1.101049E-02, 4.106300E-03
c, 1.20, -2.158944E-07, -1.678519E-06, -8.831213E-06, -3.727769E-05, -1.356099E-04, -4.392351E-04, -1.286948E-03, -3.382149E-03, -7.765646E-03, -1.447985E-02,
       -1.715502E-02, 4.610101E-03, 6.827831E-02, 1.161368E-01, -3.277049E-02, -2.280000E-01, -2.793991E-01, 2.771165E-01, 5.852999E-01, 2.963946E-01
c, 1.20, -4.093416E-07, -3.258956E-06, -1.656390E-05, -7.316689E-05, -2.512784E-04, -8.732657E-04, -2.347654E-03, -6.853150E-03, -1.376420E-02, -3.084679E-02,
       -2.562208E-02, -1.005123E-02, 1.795330E-01, 1.522400E-01, 9.483887E-02, -1.014876E+00, 2.308810E-01, 2.113321E+00, -1.253048E+00, -6.139502E-01
c, 1.20, -6.539963E-07, -5.267399E-06, -2.632092E-05, -1.188719E-04, -3.968552E-04, -1.427460E-03, -3.679181E-03, -1.130774E-02, -2.128221E-02, -5.218271E-02,
       -3.599014E-02, -3.088759E-02, 3.380657E-01, 2.389559E-01, 7.609068E-02, -2.408113E+00, 2.633592E+00, -5.358510E-02, -2.230135E+00, 1.820787E+00
c, 20.20, 1.000000E+00
c, 21.21, 1.0000000
p, V , 1.327320E+04, 3.142126E+03, 1.020588E+03, 3.904407E+02, 1.655043E+02, 7.532006E+01, 3.605503E+01, 1.780436E+01, 9.002929E+00, 4.594544E+00, 2.276760E+00,
       1.101178E+00, 5.186380E-01, 2.005650E-01, 8.129100E-02, 3.179500E-02, 0.0124400
c, 1.16, 4.300000E-05, 3.840000E-04, 2.210000E-03, 9.678000E-03, 3.393600E-02, 9.591700E-02, 2.088530E-01, 3.306600E-01, 3.323120E-01, 1.581880E-01, 2.225200E-02,
       -1.565000E-03, -1.353000E-03, -2.650000E-04, 2.900000E-05, -1.100000E-05
c, 1.16, -1.500000E-05, -1.310000E-04, -7.550000E-04, -3.325000E-03, -1.181100E-02, -3.425600E-02, -7.736300E-02, -1.284560E-01, -1.350780E-01, 2.083800E-02,
       3.204990E-01, 4.602600E-01, 2.953460E-01, 4.904600E-02, -3.824000E-03, 1.585000E-03
c, 1.16, 4.000000E-06, 3.900000E-05, 2.230000E-04, 9.960000E-04, 3.498000E-03, 1.029600E-02, 2.296200E-02, 3.920800E-02, 3.994300E-02, -7.121000E-03, -1.162250E-01,
       -1.694960E-01, -1.553740E-01, 3.950220E-01, 6.789080E-01, 8.312200E-02
c, 1.16, 9.000000E-06, 7.500000E-05, 4.790000E-04, 1.906000E-03, 7.512000E-03, 1.953000E-02, 5.040200E-02, 7.068500E-02, 1.087750E-01, -5.853600E-02, -2.154480E-01,
       -6.773420E-01, 5.859790E-01, 1.129108E+00, -9.740510E-01, -1.952640E-01
c, 1.16, 4.000000E-06, 3.200000E-05, 1.830000E-04, 8.020000E-04, 2.862000E-03, 8.287000E-03, 1.887000E-02, 3.130700E-02, 3.366000E-02, -9.479000E-03, -9.231300E-02,
       -1.489890E-01, -8.364400E-02, 2.493390E-01, 5.805150E-01, 3.223800E-01
c, 16.16, 1.000000E+00
c, 17.17, 1.0000000
d, V , 7.761150E+01, 2.291590E+01, 8.279540E+00, 3.309930E+00, 1.358630E+00, 5.413500E-01, 2.023560E-01, 6.756800E-02, 0.0225600
c, 1.8, 3.595000E-03, 2.521000E-02, 9.478600E-02, 2.303630E-01, 3.528940E-01, 3.704140E-01, 2.457180E-01, 6.099300E-02
c, 1.8, -3.818000E-03, -2.671700E-02, -1.036900E-01, -2.476890E-01, -3.115230E-01, -2.282700E-02, 5.697260E-01, 4.194930E-01
c, 1.8, 6.001000E-03, 4.220600E-02, 1.707510E-01, 3.855180E-01, 2.062040E-01, -7.786690E-01, -1.147210E-01, 7.270620E-01
c, 8.8, 1.000000E+00
c, 9.9, 1.0000000
f, V , 1.262379693766E+00, 3.362791956868E-01
c, 1.1, 1.000000E+00
c, 2.2, 1.000000E+00
```

! chromium (21s,17p,9d,2f) -> [8s,7p,5d,2f]
s, CR , 6.177194E+06, 9.249295E+05, 2.104865E+05, 5.962005E+04, 1.945076E+04, 7.022056E+03, 2.738763E+03, 1.135814E+03, 4.950923E+02, 2.247487E+02,
1.053836E+02, 5.019359E+01, 2.224957E+01, 1.098265E+01, 5.383665E+00, 2.343685E+00, 1.105202E+00, 4.878480E-01, 8.959900E-02, 3.342300E-02, 0.0124700
c, 1.20, 4.128667E-06, 3.210767E-05, 1.688416E-04, 7.128520E-04, 2.589325E-03, 8.377350E-03, 2.441725E-02, 6.365135E-02, 1.427618E-01, 2.541275E-01,
3.009512E-01, 1.766513E-01, 6.936709E-02, 1.179579E-01, 8.916187E-02, 1.103630E-02, -3.546048E-04, 1.057311E-04, 1.114640E-05, 2.661387E-05
c, 1.20, -2.301772E-06, -1.789536E-05, -9.416174E-05, -3.975074E-04, -1.447025E-03, -4.694622E-03, -1.382387E-02, -3.674643E-02, -8.647185E-02, -1.696735E-01,
-2.507089E-01, -1.961156E-01, 1.457244E-01, 5.466706E-01, 3.979434E-01, 5.277007E-02, -4.374537E-03, 3.204035E-04, -5.142077E-05, 1.584134E-04
c, 1.20, 4.862957E-07, 3.776645E-06, 1.990664E-05, 8.389164E-05, 3.065706E-04, 9.944107E-04, 2.961959E-03, 7.969473E-03, 1.955017E-02, 4.085035E-02,
6.929003E-02, 6.146984E-02, -6.981302E-02, -3.517597E-01, -3.828629E-01, 2.676401E-01, 7.175950E-01, 3.020814E-01, 7.749514E-03, 2.696096E-04
c, 1.20, -1.102451E-07, -8.530233E-07, -4.520358E-06, -1.891612E-05, -6.974344E-05, -2.237867E-04, -6.754503E-04, -1.789346E-03, -4.477858E-03, -9.140144E-03,
-1.610562E-02, -1.334870E-02, 1.426027E-02, 8.931690E-02, 8.885279E-02, -6.368776E-02, -2.783262E-01, -1.830071E-01, 6.790937E-01, 4.672953E-01
c, 1.20, -3.669010E-07, -2.768353E-06, -1.521014E-05, -6.066288E-05, -2.374959E-04, -7.077071E-04, -2.334389E-03, -5.555062E-03, -1.585142E-02, -2.737888E-02,
-6.106975E-02, -2.887227E-02, 6.813714E-03, 4.327670E-01, 1.968410E-01, -3.764657E-01, -2.052473E+00, 2.975741E+00, -2.211705E+00, 1.370188E+00
c, 20.20, 1.000000E+00
c, 1.20, 2.179893E-07, 1.612940E-06, 9.111842E-06, 3.500645E-05, 1.435315E-04, 4.035896E-04, 1.425177E-03, 3.114009E-03, 9.814449E-03, 1.474698E-02,
3.911512E-02, 9.170888E-03, 1.559878E-02, -2.816844E-01, -6.895261E-03, -1.769781E-01, 1.443061E+00, -1.029318E+00, -1.307667E+00, 1.503842E+00
c, 21.21, 1.0000000
p, CR , 1.445420E+04, 3.421676E+03, 1.111387E+03, 4.251918E+02, 1.802623E+02, 8.206117E+01, 3.929726E+01, 1.941959E+01, 9.828899E+00, 5.016810E+00,
2.487091E+00, 1.198780E+00, 5.586950E-01, 2.089240E-01, 8.460800E-02, 3.325800E-02, 0.0130700
c, 1.16, 4.400000E-05, 3.890000E-04, 2.241000E-03, 9.821000E-03, 3.447100E-02, 9.746000E-02, 2.119850E-01, 3.339900E-01, 3.301370E-01, 1.522270E-01,
2.042500E-02, -1.360000E-03, -1.195000E-03, -1.970000E-04, 2.300000E-05, -9.000000E-06
c, 1.16, -1.500000E-05, -1.350000E-04, -7.770000E-04, -3.427000E-03, -1.218900E-02, -3.538800E-02, -7.991500E-02, -1.323350E-01, -1.354010E-01, 3.200800E-02,
3.338490E-01, 4.617730E-01, 2.812900E-01, 4.184300E-02, -4.002000E-03, 1.521000E-03
c, 1.16, 4.000000E-06, 4.000000E-05, 2.290000E-04, 1.019000E-03, 3.602000E-03, 1.055000E-02, 2.370200E-02, 3.998800E-02, 4.043700E-02, -1.207400E-02,
-1.189390E-01, -1.781000E-01, -1.238650E-01, 4.297220E-01, 6.507860E-01, 6.417100E-02
c, 1.16, 1.000000E-05, 7.900000E-05, 5.120000E-04, 2.023000E-03, 8.055000E-03, 2.077200E-02, 5.422400E-02, 7.468500E-02, 1.159890E-01, -7.658400E-02,
-2.439310E-01, -6.801810E-01, 7.336640E-01, 9.991200E-01, -1.017081E+00, -1.200910E-01
c, 1.16, 4.000000E-06, 3.200000E-05, 1.850000E-04, 8.100000E-04, 2.906000E-03, 8.391000E-03, 1.919300E-02, 3.156400E-02, 3.341700E-02, -1.290700E-02,
-9.365900E-02, -1.499770E-01, -6.723400E-02, 2.707590E-01, 5.758070E-01, 3.011210E-01
c, 16.16, 1.000000E+00
c, 17.17, 1.0000000
d, CR , 8.857680E+01, 2.620450E+01, 9.517470E+00, 3.822480E+00, 1.575120E+00, 6.289280E-01, 2.344240E-01, 7.681500E-02, 0.0251700
c, 1.8, 3.621000E-03, 2.576600E-02, 9.755600E-02, 2.363120E-01, 3.582860E-01, 3.685430E-01, 2.354940E-01, 5.315600E-02
c, 1.8, -4.122000E-03, -2.930700E-02, -1.150620E-01, -2.730680E-01, -3.144230E-01, 4.209700E-02, 5.914030E-01, 3.582150E-01
c, 1.8, 5.954000E-03, 4.253200E-02, 1.745110E-01, 3.939000E-01, 1.492790E-01, -8.102450E-01, 1.091100E-02, 7.014040E-01
c, 8.8, 1.000000E+00
c, 9.9, 1.0000000
f, CR , 1.412992966329E+00, 3.829217191177E-01
c, 1.1, 1.000000E+00
c, 2.2, 1.000000E+00

```
! manganese            (21s,17p,9d,2f) -> [8s,7p,5d,2f]
s, MN , 3.960805E+06, 5.931155E+05, 1.349768E+05, 3.823067E+04, 1.247154E+04, 4.501743E+03, 1.755212E+03, 7.273039E+02, 3.163678E+02, 1.430098E+02,
          6.621805E+01, 2.991896E+01, 1.430318E+01, 6.839451E+00, 3.012374E+00, 1.418808E+00, 6.236240E-01, 1.340980E-01, 6.554800E-02, 2.958400E-02, 0.0133500
c, 1.20, 8.242127E-06, 6.408587E-05, 3.369253E-04, 1.420648E-03, 5.143683E-03, 1.648569E-02, 4.698560E-02, 1.162437E-01, 2.335277E-01, 3.292837E-01,
          2.440304E-01, 7.219806E-02, 7.687806E-02, 7.852235E-02, 1.294109E-02, -3.784873E-04, -2.503203E-05, -2.421517E-05, 3.462071E-05, 4.261482E-07
c, 1.20, -3.936095E-06, -3.060481E-05, -1.609626E-04, -6.792348E-04, -2.466182E-03, -7.957629E-03, -2.307248E-02, -5.932956E-02, -1.299451E-01, -2.212352E-01,
          -2.292550E-01, 3.580733E-02, 5.107602E-01, 5.008307E-01, 9.011830E-02, -6.909909E-03, -1.912925E-04, -6.032312E-04, 5.621608E-04, -1.021109E-04
c, 1.20, 9.462709E-07, 7.360584E-06, 3.869935E-05, 1.635110E-04, 5.941775E-04, 1.927737E-03, 5.641731E-03, 1.487848E-02, 3.414783E-02, 6.405794E-02,
          7.557659E-02, -1.946070E-02, -2.957874E-01, -4.521170E-01, 1.224531E-01, 7.169756E-01, 4.092712E-01, 2.221969E-02, -9.011202E-03, 3.691727E-03
c, 1.20, -2.095391E-07, -1.629439E-06, -8.570592E-06, -3.619272E-05, -1.316146E-04, -4.266810E-04, -1.250270E-03, -3.294665E-03, -7.581860E-03, -1.422864E-02,
          -1.693796E-02, 4.454298E-03, 6.867042E-02, 1.113335E-01, -3.900820E-02, -2.215755E-01, -2.544359E-01, 2.865866E-01, 5.755741E-01, 2.898778E-01
c, 1.20, -4.121231E-07, -3.282099E-06, -1.667433E-05, -7.369999E-05, -2.529495E-04, -8.801425E-04, -2.365482E-03, -6.926354E-03, -1.393851E-02, -3.143840E-02,
          -2.625749E-02, -1.048313E-02, 1.856472E-01, 1.524839E-01, 7.411368E-02, -1.018097E+00, 2.980372E-01, 1.971989E+00, -1.179253E+00, -5.837703E-01
c, 1.20, -6.805493E-07, -5.460516E-06, -2.743893E-05, -1.230291E-04, -4.146269E-04, -1.475151E-03, -3.859140E-03, -1.168405E-02, -2.257185E-02, -5.399759E-02,
          -4.032351E-02, -2.742526E-02, 3.485593E-01, 2.757818E-01, -5.499812E-02, -2.292545E+00, 2.635644E+00, -3.379113E-01, -1.789607E+00, 1.628936E+00
c, 20.20, 1.000000E+00
c, 21.21, 1.0000000
p, MN , 1.620586E+04, 3.836274E+03, 1.246048E+03, 4.767535E+02, 2.021895E+02, 9.209487E+01, 4.414720E+01, 2.185468E+01, 1.108596E+01, 5.674108E+00,
          2.823170E+00, 1.368621E+00, 6.444310E-01, 2.483820E-01, 9.725500E-02, 3.663300E-02, 0.0138000
c, 1.16, 4.200000E-05, 3.730000E-04, 2.149000E-03, 9.445000E-03, 3.329700E-02, 9.475900E-02, 2.081440E-01, 3.318050E-01, 3.331750E-01, 1.576010E-01, 2.144500E-02,
          -2.558000E-03, -2.027000E-03, -3.600000E-04, 3.400000E-05, -1.300000E-05
c, 1.16, -1.500000E-05, -1.290000E-04, -7.480000E-04, -3.308000E-03, -1.181100E-02, -3.453300E-02, -7.878500E-02, -1.321830E-01, -1.371950E-01, 2.707500E-02,
          3.288910E-01, 4.572800E-01, 2.889080E-01, 4.743300E-02, -3.522000E-03, 1.456000E-03
c, 1.16, 4.000000E-06, 4.000000E-05, 2.260000E-04, 1.013000E-03, 3.575000E-03, 1.061200E-02, 2.390200E-02, 4.127900E-02, 4.147500E-02, -9.458000E-03, -1.236950E-01,
          -1.743920E-01, -1.291700E-01, 4.003480E-01, 6.696460E-01, 8.273200E-02
c, 1.16, 1.000000E-05, 8.100000E-05, 5.120000E-04, 2.071000E-03, 8.111000E-03, 2.155700E-02, 5.531600E-02, 8.026300E-02, 1.177300E-01, -6.506900E-02, -2.829140E-01,
          -6.491660E-01, 6.925670E-01, 9.789900E-01, -9.213570E-01, -1.699150E-01
c, 1.16, 3.000000E-06, 3.000000E-05, 1.720000E-04, 7.620000E-04, 2.726000E-03, 7.976000E-03, 1.828700E-02, 3.077600E-02, 3.237300E-02, -9.978000E-03, -9.052900E-02,
            -1.380040E-01, -7.796500E-02, 2.295600E-01, 5.761220E-01, 3.485380E-01
c, 16.16, 1.000000E+00
c, 17.17, 1.0000000
d, MN , 1.006630E+02, 2.983360E+01, 1.088940E+01, 4.393580E+00, 1.817820E+00, 7.278270E-01, 2.712950E-01, 8.830900E-02, 0.0287500
c, 1.8, 3.579000E-03, 2.582700E-02, 9.855900E-02, 2.383270E-01, 3.587070E-01, 3.650920E-01, 2.337380E-01, 5.661800E-02
c, 1.8, -3.454000E-03, -2.492500E-02, -9.763500E-02, -2.366920E-01, -2.923500E-01, -4.973000E-03, 5.065880E-01, 4.979510E-01
c, 1.8, 5.685000E-03, 4.117100E-02, 1.693220E-01, 3.859120E-01, 1.869090E-01, -6.781130E-01, -2.582600E-01, 7.679850E-01
c, 8.8, 1.000000E+00
c, 9.9, 1.0000000
f, MN , 1.566029720686E+00, 4.273660314969E-01
c, 1.1, 1.000000E+00
c, 2.2, 1.000000E+00
```

```
! iron               (21s,17p,9d,2f) -> [8s,7p,5d,2f]
s, FE , 4.316265E+06, 6.463424E+05, 1.470897E+05, 4.166152E+04, 1.359077E+04, 4.905750E+03, 1.912746E+03, 7.926043E+02, 3.448065E+02, 1.558999E+02,
        7.223091E+01, 3.272506E+01, 1.566762E+01, 7.503483E+00, 3.312223E+00, 1.558471E+00, 6.839140E-01, 1.467570E-01, 7.058300E-02, 3.144900E-02, 0.0140100
c, 1.20, 8.048803E-06, 6.258306E-05, 3.290239E-04, 1.387355E-03, 5.023256E-03, 1.610140E-02, 4.590034E-02, 1.136154E-01, 2.283869E-01, 3.221159E-01,
        2.383661E-01, 7.404667E-02, 9.214197E-02, 9.339790E-02, 1.573965E-02, -4.186682E-04, 5.376318E-05, -3.816654E-05, 4.319603E-05, -3.401019E-06
c, 1.20, -4.155954E-06, -3.231401E-05, -1.699525E-04, -7.171369E-04, -2.603625E-03, -8.399109E-03, -2.434109E-02, -6.251948E-02, -1.365929E-01, -2.312707E-01,
        -2.383734E-01, 3.123837E-02, 5.086818E-01, 4.987695E-01, 9.033552E-02, -6.005337E-03, 2.312454E-04, -5.643680E-04, 4.992260E-04, -1.015293E-04
c, 1.20, 9.532178E-07, 7.414605E-06, 3.898393E-05, 1.647152E-04, 5.985980E-04, 1.942390E-03, 5.687237E-03, 1.501329E-02, 3.452455E-02, 6.495820E-02,
        7.716194E-02, -1.873411E-02, -3.009185E-01, -4.554661E-01, 1.286463E-01, 7.183316E-01, 4.051743E-01, 2.168227E-02, -8.343566E-03, 3.658979E-03
c, 1.20, -2.063008E-07, -1.604169E-06, -8.438437E-06, -3.563151E-05, -1.295998E-04, -4.201534E-04, -1.231954E-03, -3.248922E-03, -7.493717E-03, -1.410102E-02,
        -1.691600E-02, 4.218996E-03, 6.833810E-02, 1.098201E-01, -4.009005E-02, -2.174739E-01, -2.465135E-01, 2.731435E-01, 5.748321E-01, 3.012713E-01
c, 1.20, -4.009367E-07, -3.189255E-06, -1.623079E-05, -7.157920E-05, -2.463958E-04, -8.544907E-04, -2.307593E-03, -6.728292E-03, -1.366165E-02, -3.062240E-02,
        -2.631137E-02, -9.760183E-03, 1.801906E-01, 1.529634E-01, 5.505413E-02, -9.551364E-01, 2.586813E-01, 1.834049E+00, -9.333240E-01, -6.981605E-01
c, 1.20, -6.966042E-07, -5.568036E-06, -2.813684E-05, -1.252418E-04, -4.260787E-04, -1.499060E-03, -3.979103E-03, -1.185686E-02, -2.346734E-02, -5.467736E-02,
        -4.393820E-02, -2.376103E-02, 3.435928E-01, 3.192960E-01, -1.343207E-01, -2.221020E+00, 2.571142E+00, -2.292404E-01, -1.832452E+00, 1.591333E+00
c, 20.20, 1.000000E+00
c, 21.21, 1.0000000
p, FE , 1.774569E+04, 4.200721E+03, 1.364429E+03, 5.220806E+02, 2.214595E+02, 1.009096E+02, 4.840115E+01, 2.398536E+01, 1.218250E+01, 6.242298E+00,
        3.110944E+00, 1.509958E+00, 7.108450E-01, 2.731900E-01, 1.042330E-01, 3.829100E-02, 0.0140700
c, 1.16, 4.100000E-05, 3.690000E-04, 2.129000E-03, 9.369000E-03, 3.309700E-02, 9.443100E-02, 2.080770E-01, 3.323330E-01, 3.329870E-01, 1.568430E-01,
        2.154900E-02, -2.095000E-03, -1.739000E-03, -3.000000E-04, 2.900000E-05, -1.100000E-05
c, 1.16, -1.500000E-05, -1.300000E-04, -7.510000E-04, -3.329000E-03, -1.191200E-02, -3.493300E-02, -7.998900E-02, -1.346360E-01, -1.385980E-01, 3.027800E-02,
        3.332160E-01, 4.561530E-01, 2.850510E-01, 4.614400E-02, -3.249000E-03, 1.357000E-03
c, 1.16, 1.100000E-05, 8.700000E-05, 5.410000E-04, 2.226000E-03, 8.593000E-03, 2.333900E-02, 5.884400E-02, 8.828900E-02, 1.231920E-01, -6.318600E-02,
        -3.549020E-01, -6.197080E-01, 8.129860E-01, 8.191180E-01, -9.017050E-01, -1.359130E-01
c, 1.16, 5.000000E-06, 4.200000E-05, 2.410000E-04, 1.085000E-03, 3.831000E-03, 1.142300E-02, 2.579200E-02, 4.481800E-02, 4.459800E-02, -1.117700E-02,
        -1.381340E-01, -1.882850E-01, -1.073990E-01, 4.448630E-01, 6.402390E-01, 6.445700E-02
c, 1.16, 3.000000E-06, 2.900000E-05, 1.650000E-04, 7.340000E-04, 2.626000E-03, 7.725000E-03, 1.773300E-02, 3.005500E-02, 3.109400E-02, -1.004800E-02,
        -8.830600E-02, -1.298240E-01, -7.693700E-02, 2.126610E-01, 5.730610E-01, 3.696510E-01
c, 16.16, 1.000000E+00
c, 17.17, 1.0000000
d, FE , 1.133440E+02, 3.364140E+01, 1.233100E+01, 4.994780E+00, 2.072800E+00, 8.307530E-01, 3.091780E-01, 1.001300E-01, 0.0324300
c, 1.8, 3.530000E-03, 2.578400E-02, 9.911900E-02, 2.390730E-01, 3.571990E-01, 3.621880E-01, 2.364610E-01, 6.011800E-02
c, 1.8, -3.890000E-03, -2.844200E-02, -1.124290E-01, -2.742570E-01, -3.155460E-01, 5.710900E-02, 5.636040E-01, 3.846370E-01
c, 1.8, 5.695000E-03, 4.200100E-02, 1.735400E-01, 4.101570E-01, 1.132520E-01, -7.696800E-01, -3.164300E-02, 7.137970E-01
c, 8.8, 1.000000E+00
c, 9.9, 1.0000000
f, FE , 1.577248053099E+00, 4.251380732880E-01
c, 1.1, 1.000000E+00
c, 2.2, 1.000000E+00
```

! cobalt (21s,17p,9d,2f) -> [8s,7p,5d,2f]
s, CO , 4.675675E+06, 7.001615E+05, 1.593373E+05, 4.513046E+04, 1.472238E+04, 5.314222E+03, 2.072018E+03, 8.586188E+02, 3.735497E+02, 1.689229E+02, 7.829639E+01, 3.552123E+01, 1.704144E+01, 8.173000E+00, 3.610318E+00, 1.697047E+00, 7.435320E-01, 1.583440E-01, 7.503600E-02, 3.309100E-02, 0.0145900
c, 1.20, 7.979026E-06, 6.204071E-05, 3.261735E-04, 1.375360E-03, 4.979997E-03, 1.596434E-02, 4.552086E-02, 1.127385E-01, 2.268262E-01, 3.203074E-01, 2.374021E-01, 7.477686E-02, 9.581872E-02, 9.649911E-02, 1.623362E-02, -4.535497E-04, 5.113519E-05, -4.174508E-05, 4.027577E-05, -5.789067E-06
c, 1.20, -4.200240E-06, -3.265831E-05, -1.717644E-04, -7.247853E-04, -2.631462E-03, -8.489272E-03, -2.460619E-02, -6.322059E-02, -1.381957E-01, -2.340680E-01, -2.415002E-01, 3.035312E-02, 5.101341E-01, 4.974939E-01, 8.970746E-02, -5.941034E-03, 2.175362E-04, -5.480155E-04, 4.525804E-04, -1.066748E-04
c, 1.20, 9.592692E-07, 7.461851E-06, 3.923137E-05, 1.657706E-04, 6.024335E-04, 1.955217E-03, 5.726326E-03, 1.512984E-02, 3.483973E-02, 6.570351E-02, 7.831503E-02, -1.877037E-02, -3.062663E-01, -4.566429E-01, 1.378169E-01, 7.193676E-01, 3.992579E-01, 2.079933E-02, -7.820663E-03, 3.533911E-03
c, 1.20, -2.028840E-07, -1.577580E-06, -8.298813E-06, -3.504154E-05, -1.274655E-04, -4.132695E-04, -1.212261E-03, -3.199318E-03, -7.390972E-03, -1.393649E-02, -1.678575E-02, 4.149856E-03, 6.797646E-02, 1.075807E-01, -4.166022E-02, -2.128044E-01, -2.381360E-01, 2.650788E-01, 5.722774E-01, 3.091556E-01
c, 1.20, -7.174687E-07, -5.722157E-06, -2.900970E-05, -1.285845E-04, -4.398434E-04, -1.537586E-03, -4.116022E-03, -1.215514E-02, -2.440441E-02, -5.601976E-02, -4.689449E-02, -2.168828E-02, 3.477789E-01, 3.500597E-01, -2.048551E-01, -2.163211E+00, 2.524091E+00, -1.590011E-01, -1.825267E+00, 1.534207E+00
c, 1.20, -3.863053E-07, -3.068788E-06, -1.564826E-05, -6.883588E-05, -2.377367E-04, -8.213173E-04, -2.229630E-03, -6.467841E-03, -1.325463E-02, -2.946686E-02, -2.599066E-02, -8.499807E-03, 1.727316E-01, 1.512189E-01, 3.554509E-02, -8.829353E-01, 2.143530E-01, 1.711865E+00, -7.140037E-01, -8.027727E-01
c, 20.20, 1.000000E+00
c, 21.21, 1.0000000
p, CO , 1.926778E+04, 4.560986E+03, 1.481436E+03, 5.668671E+02, 2.404910E+02, 1.096105E+02, 5.259491E+01, 2.608361E+01, 1.326143E+01, 6.799778E+00, 3.393414E+00, 1.648766E+00, 7.762820E-01, 2.980030E-01, 1.136180E-01, 4.162400E-02, 0.0152500
c, 1.16, 4.100000E-05, 3.690000E-04, 2.128000E-03, 9.372000E-03, 3.315500E-02, 9.475200E-02, 2.090930E-01, 3.337220E-01, 3.322080E-01, 1.546130E-01, 2.090200E-02, -2.024000E-03, -1.697000E-03, -2.800000E-04, 2.600000E-05, -1.000000E-05
c, 1.16, -1.500000E-05, -1.310000E-04, -7.580000E-04, -3.363000E-03, -1.205400E-02, -3.542400E-02, -8.128700E-02, -1.369080E-01, -1.390190E-01, 3.546800E-02, 3.384980E-01, 4.544330E-01, 2.797930E-01, 4.477600E-02, -3.151000E-03, 1.317000E-03
c, 1.16, 1.100000E-05, 9.200000E-05, 5.630000E-04, 2.354000E-03, 8.976000E-03, 2.481000E-02, 6.158000E-02, 9.477700E-02, 1.266930E-01, -6.375400E-02, -4.185660E-01, -5.678650E-01, 8.754060E-01, 7.182630E-01, -8.747220E-01, -1.319270E-01
c, 1.16, 5.000000E-06, 4.500000E-05, 2.550000E-04, 1.144000E-03, 4.061000E-03, 1.209500E-02, 2.747600E-02, 4.755700E-02, 4.730200E-02, -1.441800E-02, -1.500620E-01, -1.990920E-01, -7.978300E-02, 4.590350E-01, 6.174950E-01, 6.469000E-02
c, 1.16, -3.000000E-06, -2.900000E-05, -1.670000E-04, -7.420000E-04, -2.662000E-03, -7.841000E-03, -1.805100E-02, -3.058000E-02, -3.131200E-02, 1.131100E-02, 8.999000E-02, 1.307330E-01, 7.180800E-02, -2.216580E-01, -5.710250E-01, -3.637890E-01
c, 16.16, 1.000000E+00
c, 17.17, 1.0000000
d, CO , 1.262640E+02, 3.752260E+01, 1.380210E+01, 5.609270E+00, 2.333690E+00, 9.364150E-01, 3.482370E-01, 1.123530E-01, 0.0362500
c, 1.8, 3.510000E-03, 2.588400E-02, 1.000580E-01, 2.405470E-01, 3.568430E-01, 3.595790E-01, 2.366290E-01, 6.212900E-02
c, 1.8, -4.067000E-03, -3.005300E-02, -1.196200E-01, -2.915130E-01, -3.180480E-01, 9.169800E-02, 5.608230E-01, 3.586780E-01
c, 1.8, 5.470000E-03, 4.081300E-02, 1.689780E-01, 4.093590E-01, 8.631600E-02, -7.690080E-01, 3.355000E-03, 7.184230E-01
c, 8.8, 1.000000E+00
c, 9.9, 1.0000000
f, CO , 2.075964870009E+00, 5.198762768143E-01
c, 1.1, 1.000000E+00
c, 2.2, 1.000000E+00

```
! nickel              (21s,17p,9d,2f) -> [8s,7p,5d,2f]
s, NI , 5.045991E+06, 7.556142E+05, 1.719568E+05, 4.870479E+04, 1.588841E+04, 5.735123E+03, 2.236137E+03, 9.266468E+02, 4.031743E+02, 1.823476E+02,
        8.454885E+01, 3.839634E+01, 1.845859E+01, 8.863548E+00, 3.916227E+00, 1.838870E+00, 8.043620E-01, 1.697970E-01, 7.930600E-02, 3.467700E-02, 0.0151600
c, 1.20, 8.208996E-06, 6.382884E-05, 3.355800E-04, 1.415075E-03, 5.124444E-03, 1.643256E-02, 4.689398E-02, 1.163534E-01, 2.350511E-01, 3.350184E-01,
        2.534779E-01, 7.300901E-02, 6.184244E-02, 6.302956E-02, 1.008063E-02, -2.244528E-04, -5.932767E-05, -1.158562E-05, 8.115109E-06, -1.681699E-06
c, 1.20, -3.657849E-06, -2.844094E-05, -1.495928E-04, -6.313009E-04, -2.293052E-03, -7.405123E-03, -2.152032E-02, -5.560974E-02, -1.230176E-01, -2.130104E-01,
        -2.265837E-01, 3.546796E-02, 5.181697E-01, 5.025630E-01, 8.955674E-02, -7.031311E-03, -4.339167E-04, -5.831711E-04, 4.228788E-04, -1.266714E-04
c, 1.20, 9.594149E-07, 7.462614E-06, 3.923843E-05, 1.657868E-04, 6.025905E-04, 1.955662E-03, 5.730391E-03, 1.514756E-02, 3.493499E-02, 6.598072E-02,
        7.893083E-02, -1.906249E-02, -3.095921E-01, -4.558610E-01, 1.482931E-01, 7.134039E-01, 3.976063E-01, 2.295523E-02, -9.151758E-03, 3.875414E-03
c, 1.20, -2.013753E-07, -1.565832E-06, -8.237182E-06, -3.478105E-05, -1.265265E-04, -4.102589E-04, -1.203834E-03, -3.179062E-03, -7.353828E-03, -1.389022E-02,
        -1.677875E-02, 4.163378E-03, 6.814703E-02, 1.061029E-01, -4.339980E-02, -2.094950E-01, -2.310271E-01, 2.590532E-01, 5.691426E-01, 3.158125E-01
c, 1.20, -7.303633E-07, -5.802013E-06, -2.958547E-05, -1.301499E-04, -4.495332E-04, -1.553314E-03, -4.219884E-03, -1.225419E-02, -2.519707E-02, -5.621717E-02,
        -5.022229E-02, -1.677412E-02, 3.388021E-01, 3.984975E-01, -3.032053E-01, -2.079619E+00, 2.500542E+00, -2.169002E-01, -1.709178E+00, 1.469166E+00
c, 1.20, -3.924245E-07, -3.113909E-06, -1.590447E-05, -6.981394E-05, -2.417848E-04, -8.326195E-04, -2.270294E-03, -6.557427E-03, -1.354288E-02, -2.989768E-02,
        -2.693106E-02, -7.827693E-03, 1.741667E-01, 1.595468E-01, 1.995550E-02, -8.897000E-01, 2.486892E-01, 1.613012E+00, -5.990277E-01, -8.369078E-01
c, 20.20, 1.000000E+00
c, 21.21, 1.0000000
p, NI , 2.102792E+04, 4.977560E+03, 1.616740E+03, 6.186718E+02, 2.625183E+02, 1.196907E+02, 5.746585E+01, 2.852829E+01, 1.452148E+01, 7.453850E+00,
        3.723553E+00, 1.809813E+00, 8.513360E-01, 3.248140E-01, 1.195220E-01, 4.236600E-02, 0.0150200
c, 1.16, 4.100000E-05, 3.630000E-04, 2.097000E-03, 9.250000E-03, 3.279600E-02, 9.400400E-02, 2.082800E-01, 3.336540E-01, 3.329040E-01, 1.553720E-01,
        2.085900E-02, -2.440000E-03, -1.998000E-03, -3.380000E-04, 3.500000E-05, -1.200000E-05
c, 1.16, -1.500000E-05, -1.290000E-04, -7.490000E-04, -3.328000E-03, -1.194700E-02, -3.524200E-02, -8.120400E-02, -1.374930E-01, -1.392260E-01, 3.601600E-02,
        3.391280E-01, 4.504720E-01, 2.817830E-01, 4.789800E-02, -2.987000E-03, 1.309000E-03
c, 1.16, 6.000000E-06, 5.300000E-05, 3.050000E-04, 1.364000E-03, 4.876000E-03, 1.450300E-02, 3.329600E-02, 5.748200E-02, 5.870200E-02, -1.990400E-02,
        -1.946950E-01, -2.396130E-01, -2.232000E-03, 5.214350E-01, 5.455400E-01, 4.362200E-02
c, 1.16, 1.100000E-05, 9.500000E-05, 5.800000E-04, 2.451000E-03, 9.282000E-03, 2.600900E-02, 6.409600E-02, 1.007100E-01, 1.325390E-01, -6.508900E-02,
        -4.897560E-01, -4.984550E-01, 9.663570E-01, 5.283790E-01, -8.676760E-01, -1.044560E-01
c, 1.16, 3.000000E-06, 2.600000E-05, 1.520000E-04, 6.780000E-04, 2.427000E-03, 7.201000E-03, 1.657800E-02, 2.839200E-02, 2.859900E-02, -1.013200E-02,
        -8.291200E-02, -1.159980E-01, -7.279500E-02, 1.956400E-01, 5.670990E-01, 3.952700E-01
c, 16.16, 1.000000E+00
c, 17.17, 1.0000000
d, NI , 1.402527E+02, 4.172610E+01, 1.539810E+01, 6.277100E+00, 2.618500E+00, 1.052600E+00, 3.916000E-01, 1.262000E-01, 0.0406700
c, 1.8, 3.376000E-03, 2.514100E-02, 9.774600E-02, 2.347090E-01, 3.469450E-01, 3.510680E-01, 2.502550E-01, 1.000820E-01
c, 1.8, -3.495000E-03, -2.601500E-02, -1.038760E-01, -2.520700E-01, -2.945800E-01, 1.152000E-03, 4.385890E-01, 5.436260E-01
c, 1.8, 5.052000E-03, 3.808700E-02, 1.561130E-01, 3.861560E-01, 1.756050E-01, -6.268070E-01, -3.427750E-01, 7.918190E-01
c, 8.8, 1.000000E+00
c, 9.9, 1.0000000
f, NI , 4.711942694203E+00, 7.437380486770E-01
c, 1.1, 1.000000E+00
c, 2.2, 1.000000E+00
```

```
! copper              (21s,17p,9d,2f) -> [8s,7p,5d,2f]
s, CU , 5.430321E+06, 8.131665E+05, 1.850544E+05, 5.241466E+04, 1.709868E+04, 6.171994E+03, 2.406481E+03, 9.972584E+02, 4.339289E+02, 1.962869E+02,
        9.104280E+01, 4.138425E+01, 1.993278E+01, 9.581891E+00, 4.234516E+00, 1.985814E+00, 8.670830E-01, 1.813390E-01, 8.365700E-02, 3.626700E-02, 0.0157200
c, 1.20, 7.801026E-06, 6.065666E-05, 3.188964E-04, 1.344687E-03, 4.869050E-03, 1.561013E-02, 4.452077E-02, 1.103111E-01, 2.220342E-01, 3.133739E-01,
        2.315121E-01, 7.640920E-02, 1.103818E-01, 1.094372E-01, 1.836311E-02, -6.043084E-04, 5.092245E-05, -5.540730E-05, 3.969482E-05, -1.269538E-05
c, 1.20, -4.404706E-06, -3.424801E-05, -1.801238E-04, -7.600455E-04, -2.759348E-03, -8.900970E-03, -2.579378E-02, -6.623861E-02, -1.445927E-01, -2.440110E-01,
        -2.504837E-01, 2.852577E-02, 5.115874E-01, 4.928061E-01, 8.788437E-02, -5.820281E-03, 2.013508E-04, -5.182553E-04, 3.731503E-04, -1.193171E-04
c, 1.20, 9.704682E-07, 7.549245E-06, 3.968892E-05, 1.677200E-04, 6.095101E-04, 1.978846E-03, 5.798049E-03, 1.534158E-02, 3.540484E-02, 6.702098E-02,
        8.026945E-02, -1.927231E-02, -3.160129E-01, -4.573162E-01, 1.550841E-01, 7.202872E-01, 3.885122E-01, 1.924326E-02, -7.103807E-03, 3.272906E-03
c, 1.20, -1.959354E-07, -1.523472E-06, -8.014808E-06, -3.383992E-05, -1.231191E-04, -3.992085E-04, -1.171900E-03, -3.096141E-03, -7.171993E-03, -1.356621E-02,
        -1.643989E-02, 4.107628E-03, 6.693964E-02, 1.028221E-01, -4.422945E-02, -2.031191E-01, -2.230022E-01, 2.517975E-01, 5.650091E-01, 3.247243E-01
c, 1.20, -7.508267E-07, -5.972018E-06, -3.039682E-05, -1.340405E-04, -4.615778E-04, -1.601064E-03, -4.330942E-03, -1.265434E-02, -2.586864E-02, -5.835428E-02,
        -5.132322E-02, -1.908953E-02, 3.586116E-01, 3.885818E-01, -3.057106E-01, -2.069896E+00, 2.431774E+00, -2.121974E-02, -1.820251E+00, 1.434585E+00
c, 1.20, -3.532229E-07, -2.798812E-06, -1.432517E-05, -6.270946E-05, -2.179490E-04, -7.474316E-04, -2.049271E-03, -5.885203E-03, -1.226885E-02, -2.683147E-02,
        -2.479261E-02, -5.984746E-03, 1.557124E-01, 1.436683E-01, 8.374103E-03, -7.460711E-01, 1.244367E-01, 1.510110E+00, -3.477122E-01, -9.774169E-01
c, 20.20, 1.000000E+00
c, 21.21, 1.0000000
p, CU , 2.276057E+04, 5.387679E+03, 1.749945E+03, 6.696653E+02, 2.841948E+02, 1.296077E+02, 6.225415E+01, 3.092964E+01, 1.575827E+01, 8.094211E+00,
        4.046921E+00, 1.967869E+00, 9.252950E-01, 3.529920E-01, 1.273070E-01, 4.435600E-02, 0.0154500
c, 1.16, 4.000000E-05, 3.610000E-04, 2.083000E-03, 9.197000E-03, 3.266000E-02, 9.379500E-02, 2.082740E-01, 3.339930E-01, 3.324930E-01, 1.547280E-01,
        2.127100E-02, -1.690000E-03, -1.516000E-03, -2.420000E-04, 2.300000E-05, -9.000000E-06
c, 1.16, -1.500000E-05, -1.310000E-04, -7.550000E-04, -3.359000E-03, -1.208100E-02, -3.570300E-02, -8.250200E-02, -1.398900E-01, -1.407290E-01, 3.876600E-02,
        3.426950E-01, 4.523100E-01, 2.770540E-01, 4.388500E-02, -2.802000E-03, 1.152000E-03
c, 1.16, 5.000000E-06, 4.900000E-05, 2.780000E-04, 1.253000E-03, 4.447000E-03, 1.337000E-02, 3.046900E-02, 5.344700E-02, 5.263900E-02, -1.688100E-02,
        -1.794480E-01, -2.095880E-01, -3.963300E-02, 5.021300E-01, 5.811110E-01, 4.566600E-02
c, 1.16, 1.100000E-05, 9.600000E-05, 5.900000E-04, 2.484000E-03, 9.463000E-03, 2.645300E-02, 6.568900E-02, 1.027320E-01, 1.370410E-01, -7.096100E-02,
        -5.047080E-01, -4.780560E-01, 9.428920E-01, 5.446990E-01, -8.327660E-01, -1.084160E-01
c, 1.16, 3.000000E-06, 2.500000E-05, 1.470000E-04, 6.560000E-04, 2.351000E-03, 7.004000E-03, 1.613100E-02, 2.777000E-02, 2.756700E-02, -1.011500E-02,
        -8.100900E-02, -1.104090E-01, -7.173200E-02, 1.879300E-01, 5.646290E-01, 4.070000E-01
c, 16.16, 1.000000E+00
c, 17.17, 1.0000000
d, CU , 1.738970E+02, 5.188690E+01, 1.934190E+01, 7.975720E+00, 3.398230E+00, 1.409320E+00, 5.488580E-01, 1.901990E-01, 0.0659100
c, 1.8, 2.700000E-03, 2.090900E-02, 8.440800E-02, 2.139990E-01, 3.359800E-01, 3.573010E-01, 2.645780E-01, 1.039720E-01
c, 1.8, -3.363000E-03, -2.607900E-02, -1.082310E-01, -2.822170E-01, -3.471900E-01, 2.671100E-02, 4.920470E-01, 4.384220E-01
c, 1.8, 4.133000E-03, 3.308500E-02, 1.383360E-01, 3.901660E-01, 1.698420E-01, -6.830180E-01, -2.657970E-01, 8.380630E-01
c, 8.8, 1.000000E+00
c, 9.9, 1.0000000
f, CU , 6.006064612854E+00, 8.719066700278E-01
c, 1.1, 1.000000E+00
c, 2.2, 1.000000E+00
```

```
! zinc                (21s,17p,9d,2f) -> [8s,7p,5d,2f]
s, ZN , 5.820021E+06, 8.715234E+05, 1.983350E+05, 5.617631E+04, 1.832582E+04, 6.614955E+03, 2.579199E+03, 1.068849E+03, 4.651045E+02, 2.104130E+02,
        9.761629E+01, 4.438020E+01, 2.142308E+01, 1.030891E+01, 4.553645E+00, 2.132821E+00, 9.296970E-01, 1.921470E-01, 8.759500E-02, 3.770200E-02, 0.0162300
c, 1.20, 8.549241E-06, 6.647410E-05, 3.494962E-04, 1.473832E-03, 5.338330E-03, 1.712708E-02, 4.894085E-02, 1.217934E-01, 2.476589E-01, 3.582431E-01,
        2.798174E-01, 6.857491E-02, -1.311092E-03, 1.914001E-03, -8.759220E-04, 3.740096E-04, -1.401399E-04, 4.757132E-05, -3.642711E-05, 1.153248E-05
c, 1.20, -2.640069E-06, -2.052720E-05, -1.079859E-04, -4.558577E-04, -1.657758E-03, -5.368492E-03, -1.571249E-02, -4.122558E-02, -9.406459E-02, -1.719954E-01,
        -1.958523E-01, 4.532907E-02, 5.244442E-01, 5.006142E-01, 8.945527E-02, -2.146262E-03, 2.112113E-03, -4.133980E-04, 3.209752E-04, -1.016140E-04
c, 1.20, 9.967103E-07, 7.754163E-06, 4.076019E-05, 1.722811E-04, 6.259370E-04, 2.032855E-03, 5.954646E-03, 1.576640E-02, 3.637638E-02, 6.892343E-02,
        8.238093E-02, -2.011360E-02, -3.252526E-01, -4.602899E-01, 1.635546E-01, 7.297118E-01, 3.769751E-01, 1.433224E-02, -6.671210E-03, 1.766214E-03
c, 1.20, 1.995818E-07, 1.552973E-06, 8.161259E-06, 3.450747E-05, 1.253275E-04, 4.072990E-04, 1.192734E-03, 3.163140E-03, 7.303942E-03, 1.391279E-02,
        1.670620E-02, -4.035586E-03, -6.968861E-02, -1.030105E-01, 4.471442E-02, 2.150027E-01, 2.220163E-01, -3.114776E-01, -5.693429E-01, -2.678440E-01
c, 1.20, 8.040791E-07, 6.282936E-06, 3.281868E-05, 1.398858E-04, 5.029836E-04, 1.655419E-03, 4.778677E-03, 1.292479E-02, 2.925831E-02, 5.791816E-02,
        6.640681E-02, -7.388966E-03, -3.329989E-01, -5.917865E-01, 9.011406E-01, 1.585951E+00, -2.788008E+00, 2.071884E+00, -6.012025E-01, -5.937184E-01
c, 1.20, -5.435910E-07, -4.336894E-06, -2.197572E-05, -9.747392E-05, -3.331615E-04, -1.166192E-03, -3.119308E-03, -9.239504E-03, -1.855471E-02, -4.281189E-02,
        -3.571095E-02, -1.638350E-02, 2.644664E-01, 2.086588E-01, -1.774382E-02, -1.353873E+00, 8.182926E-01, 1.695036E+00, -1.388656E+00, -2.188900E-01
c, 20.20, 1.000000E+00
c, 21.21, 1.0000000
p, ZN , 2.441198E+04, 5.778518E+03, 1.876862E+03, 7.182361E+02, 3.048327E+02, 1.390453E+02, 6.680417E+01, 3.320699E+01, 1.692816E+01, 8.696229E+00,
        4.350510E+00, 2.116523E+00, 9.953870E-01, 3.781120E-01, 1.345790E-01, 4.628200E-02, 0.0159200
c, 1.15, 4.100000E-05, 3.610000E-04, 2.088000E-03, 9.221000E-03, 3.277300E-02, 9.417900E-02, 2.091320E-01, 3.345690E-01, 3.303590E-01, 1.523470E-01,
        2.298400E-02, 1.607000E-03, 4.680000E-04, 6.600000E-05, -2.000000E-06
c, 1.16, -1.500000E-05, -1.350000E-04, -7.820000E-04, -3.478000E-03, -1.252000E-02, -3.701600E-02, -8.555900E-02, -1.447180E-01, -1.434420E-01, 4.359500E-02,
        3.488880E-01, 4.538650E-01, 2.685940E-01, 3.886800E-02, -2.492000E-03, 1.014000E-03
c, 1.16, 1.200000E-05, 9.600000E-05, 5.940000E-04, 2.484000E-03, 9.537000E-03, 2.647900E-02, 6.636600E-02, 1.024580E-01, 1.386830E-01, -8.014000E-02,
        -4.960690E-01, -4.635100E-01, 8.745310E-01, 6.297900E-01, -8.116860E-01, -1.089480E-01
c, 1.16, 5.000000E-06, 4.200000E-05, 2.380000E-04, 1.088000E-03, 3.821000E-03, 1.164400E-02, 2.616700E-02, 4.675000E-02, 4.330900E-02, -1.342900E-02,
        -1.538970E-01, -1.674130E-01, -8.499500E-02, 4.508130E-01, 6.408690E-01, 5.417200E-02
c, 1.16, 3.000000E-06, 2.500000E-05, 1.440000E-04, 6.450000E-04, 2.311000E-03, 6.898000E-03, 1.588200E-02, 2.735000E-02, 2.662100E-02, -1.085800E-02,
        -7.985300E-02, -1.061270E-01, -6.888300E-02, 1.843850E-01, 5.617880E-01, 4.144160E-01
c, 16.16, 1.000000E+00
c, 17.17, 1.0000000
d, ZN , 2.056177E+02, 6.144981E+01, 2.305689E+01, 9.577739E+00, 4.133734E+00, 1.747518E+00, 6.995600E-01, 2.516080E-01, 0.0904900
c, 1.8, 2.342000E-03, 1.860600E-02, 7.710200E-02, 2.020260E-01, 3.294540E-01, 3.609760E-01, 2.716570E-01, 1.049810E-01
c, 1.8, 3.279000E-03, 2.617600E-02, 1.113670E-01, 3.045810E-01, 3.862990E-01, -5.837500E-02, -5.388760E-01, -3.454730E-01
c, 1.8, 3.740000E-03, 3.182500E-02, 1.322290E-01, 4.245500E-01, 1.203700E-01, -7.626610E-01, -1.128230E-01, 8.096230E-01
c, 8.8, 1.000000E+00
c, 9.9, 1.0000000
f, ZN , 6.827944777420E+00, 9.775748050298E-01
c, 1.1, 1.000000E+00
c, 2.2, 1.000000E+00
```

cc-pVDZ-PP-F12-wis Basis Set: Y-Cd and Hf-Hg Atoms

```
! yttrium          (11s,10p,9d,2f) -> [6s,6p,5d,2f]
s, Y , 121.5550000, 13.3508000, 8.3430400, 5.2126200, 2.8482200, 0.7360920, 0.3395250, 0.0850000, 0.0457190, 0.0217750, 0.0104000
c, 1.9, 0.0003990, -0.0373740, 0.2104080, -0.1742590, -0.4490590, 0.7515460, 0.5039130, 0.0381900, -0.0168190
c, 1.9, -0.0000250, 0.0025120, -0.0493070, -0.0036710, 0.3721140, -1.0040470, -0.0462620, 2.7996160, -1.8967450
c, 1.9, -0.0001380, 0.0126550, -0.0725770, 0.0689460, 0.1410760, -0.3097690, -0.3640910, 0.3089760, 0.6183900
c, 1.9, 0.0003360, -0.0302260, 0.0575900, -0.2202050, 0.8124860, -2.9159010, 2.9601310, 0.3098150, -3.0151070
c, 10.10, 1.0000000
c, 11.11, 1.0000000
p, Y , 15.7057000, 9.8143400, 3.9515200, 1.0287300, 0.5203410, 0.2628060, 0.1191270, 0.0532630, 0.0233840, 0.0103000
c, 1.8, -0.0010080, 0.0184510, -0.1533130, 0.3882870, 0.4715490, 0.2442250, 0.0374550, 0.0009170
c, 1.8, -0.0006490, 0.0126010, -0.1098480, 0.3192010, 0.8044380, -0.8756500, -1.0192500, 0.9413270
c, 1.8, 0.0008090, -0.0150420, 0.1388090, -0.6034190, -0.8228890, 2.6576020, -1.8092190, -0.4174460
c, 1.8, -0.0001460, -0.0045380, 0.0466130, -0.1376680, -0.1888270, -0.1161280, 0.3231850, 0.6065240
c, 9.9, 1.0000000
c, 10.10, 1.0000000
d, Y , 15.8535000, 5.0818300, 1.4509500, 0.6892770, 0.3133420, 0.1383830, 0.0595550, 0.0245030, 0.0101000
c, 1.7, 0.0009920, -0.0052340, 0.0867890, 0.2297090, 0.3256070, 0.3432860, 0.2503880
c, 1.7, 0.0013370, -0.0069910, 0.1521840, 0.4617290, 0.1555670, -0.9016730, -0.0617510
c, 1.7, -0.0010910, 0.0056570, -0.1099010, -0.2902090, -0.3566240, 0.0229490, 0.5623060
c, 8.8, 1.0000000
c, 9.9, 1.0000000
f, Y , 5.081360414922E-01, 1.326508386379E-01
c, 1.1, 1.000000E+00
c, 2.2, 1.000000E+00
! zirconium          (11s,10p,9d,2f) -> [6s,6p,5d,2f]
s, ZR , 136.8260000, 14.5128000, 9.0698100, 5.6664000, 3.1131900, 0.8234820, 0.3811930, 0.0977920, 0.0508390, 0.0236930, 0.0110000
c, 1.9, 0.0004090, -0.0385170, 0.2158150, -0.1733400, -0.4700670, 0.7634610, 0.5037400, 0.0378590, -0.0153900
c, 1.9, -0.0000310, 0.0019190, -0.0509770, -0.0153840, 0.4351660, -1.1911120, 0.1292570, 2.6494430, -1.8700320
c, 1.9, -0.0001420, 0.0131450, -0.0753410, 0.0699780, 0.1504360, -0.3253820, -0.3567120, 0.3070140, 0.6239180
c, 1.9, 0.0000940, -0.0109880, -0.0305380, -0.0961350, 0.8023080, -3.2348630, 3.4978270, -0.7468840, -1.7815950
c, 10.10, 1.0000000
c, 11.11, 1.0000000
p, ZR , 17.4119000, 10.8799000, 4.3280400, 1.1386400, 0.5758550, 0.2886700, 0.1280140, 0.0552900, 0.0236130, 0.0101000
c, 1.8, -0.0011830, 0.0191740, -0.1600700, 0.4057620, 0.4736200, 0.2319610, 0.0326620, 0.0006340
c, 1.8, -0.0001070, -0.0067180, 0.0683650, -0.2045500, -0.2797650, -0.0980140, 0.6499590, 0.4986110
c, 1.8, 0.0003190, -0.0136660, 0.1316590, -0.4538160, -0.8019150, 1.2831030, 0.5992920, -1.0604800
c, 1.8, -0.0001340, -0.0046090, 0.0481890, -0.1454840, -0.1898740, -0.0778140, 0.3319510, 0.5837920
c, 9.9, 1.0000000
c, 10.10, 1.0000000
d, ZR , 18.0732000, 5.2195900, 1.6998500, 0.8363230, 0.3933540, 0.1788240, 0.0782750, 0.0322480, 0.0133000
c, 1.7, 0.0011410, -0.0082810, 0.0933770, 0.2509730, 0.3439070, 0.3298380, 0.2121770
c, 1.7, 0.0016120, -0.0118120, 0.1997150, 0.5039140, 0.0093700, -0.9116610, 0.0859150
c, 1.7, -0.0012140, 0.0087890, -0.1164370, -0.3126190, -0.3388120, 0.1097870, 0.5744830
c, 8.8, 1.0000000
c, 9.9, 1.0000000
f, ZR , 7.279138788795E-01, 1.989665181513E-01
c, 1.1, 1.000000E+00
c, 2.2, 1.000000E+00
```

```
! niobium              (11s,10p,9d,2f) -> [6s,6p,5d,2f]
s, NB , 153.0710000, 15.6363000, 9.7721800, 6.1054400, 3.3909000, 0.9116760, 0.4221390, 0.1090790, 0.0556240, 0.0255240, 0.0117000
c, 1.9, 0.0004270, -0.0398340, 0.2232090, -0.1760000, -0.4860610, 0.7744000, 0.5027280, 0.0374030, -0.0146450
c, 1.9, -0.0000530, 0.0020220, -0.0540960, -0.0218900, 0.4738280, -1.2925960, 0.2337550, 2.5185120, -1.7822210
c, 1.9, -0.0001480, 0.0135970, -0.0781900, 0.0718380, 0.1563340, -0.3360430, -0.3472660, 0.3058060, 0.6211110
c, 1.9, -0.0001160, 0.0059360, -0.1098620, 0.0225290, 0.7625580, -3.3668890, 3.7305380, -1.2109090, -1.2127230
c, 10.10, 1.0000000
c, 11.11, 1.0000000
p, NB , 18.8713000, 11.7929000, 4.7152900, 1.2553000, 0.6350230, 0.3175950, 0.1410540, 0.0604940, 0.0256300, 0.0109000
c, 1.8, -0.0013830, 0.0207100, -0.1677060, 0.4181250, 0.4741640, 0.2237340, 0.0306890, 0.0006280
c, 1.8, -0.0000540, -0.0077120, 0.0758660, -0.2266570, -0.3034970, -0.0467380, 0.6780350, 0.4528850
c, 1.8, 0.0002690, -0.0147500, 0.1419370, -0.5173210, -0.7179400, 1.3148940, 0.4894290, -1.0389510
c, 1.8, -0.0001020, -0.0050790, 0.0513210, -0.1541440, -0.1927810, -0.0631470, 0.3412080, 0.5757850
c, 9.9, 1.0000000
c, 10.10, 1.0000000
d, NB , 20.8985000, 5.6013400, 1.9380500, 0.9775120, 0.4697960, 0.2179050, 0.0969170, 0.0403560, 0.0168000
c, 1.7, 0.0012390, -0.0091370, 0.0974180, 0.2640250, 0.3520390, 0.3189190, 0.1905900
c, 1.7, 0.0018290, -0.0134510, 0.2322370, 0.5415760, -0.1098090, -0.8826960, 0.1429900
c, 1.7, -0.0013190, 0.0096450, -0.1237060, -0.3240320, -0.3217030, 0.1565040, 0.5624300
c, 8.8, 1.0000000
c, 9.9, 1.0000000
f, NB , 9.462153737008E-01, 2.611860635521E-01
c, 1.1, 1.000000E+00
c, 2.2, 1.000000E+00

! molybdenum           (11s,10p,9d,2f) -> [6s,6p,5d,2f]
s, MO , 170.0690000, 16.7338000, 10.4587000, 6.5343400, 3.6793500, 1.0019200, 0.4631400, 0.1187270, 0.0595140, 0.0270390, 0.0123000
c, 1.9, 0.0004530, -0.0413800, 0.2326990, -0.1825190, -0.4983180, 0.7853790, 0.5013700, 0.0358290, -0.0140370
c, 1.9, -0.0000750, 0.0015070, -0.0536740, -0.0331090, 0.5047010, -1.3515930, 0.3070100, 2.3973260, -1.6896850
c, 1.9, -0.0002980, 0.0209690, -0.1814220, 0.1285330, 0.7288700, -3.4647110, 3.8855920, -1.5475660, -0.7618020
c, 1.9, -0.0001560, 0.0139920, -0.0809630, 0.0744540, 0.1588830, -0.3414150, -0.3366740, 0.3095560, 0.6149970
c, 10.10, 1.0000000
c, 11.11, 1.0000000
p, MO , 20.3080000, 12.6907000, 5.1195000, 1.3778300, 0.6968240, 0.3473670, 0.1528190, 0.0650490, 0.0273390, 0.0115000
c, 1.8, -0.0015140, 0.0221680, -0.1743690, 0.4279430, 0.4753060, 0.2174030, 0.0285240, 0.0004610
c, 1.8, -0.0000340, -0.0086930, 0.0831140, -0.2494680, -0.3235300, 0.0156020, 0.6864390, 0.4139530
c, 1.8, 0.0001020, -0.0158590, 0.1536300, -0.5873860, -0.6442110, 1.3803170, 0.3384870, -0.9969510
c, 1.8, -0.0000830, -0.0054470, 0.0532030, -0.1581500, -0.1918290, -0.0524490, 0.3447620, 0.5699340
c, 9.9, 1.0000000
c, 10.10, 1.0000000
d, MO , 22.6032000, 6.3391400, 2.1776300, 1.1070000, 0.5353720, 0.2489800, 0.1104090, 0.0453770, 0.0186000
c, 1.7, 0.0013060, -0.0124380, 0.1035120, 0.2776610, 0.3588570, 0.3092260, 0.1740360
c, 1.7, 0.0020230, -0.0206910, 0.2714220, 0.5579390, -0.2102870, -0.8724070, 0.2634070
c, 1.7, -0.0013410, 0.0130470, -0.1256280, -0.3434410, -0.2978970, 0.2001940, 0.5671490
c, 8.8, 1.0000000
c, 9.9, 1.0000000
f, MO , 1.052664555606E+00, 2.763439748097E-01
c, 1.1, 1.000000E+00
c, 2.2, 1.000000E+00
```

! technetium (11s,10p,9d,2f) -> [6s,6p,5d,2f]
s, TC , 188.5090000, 17.8080000, 11.1310000, 6.9548300, 3.9764000, 1.0938100, 0.5040080, 0.1272060, 0.0630470, 0.0284080, 0.0128000
c, 1.9, 0.0004850, -0.0430660, 0.2441460, -0.1935770, -0.5059760, 0.7962750, 0.4985920, 0.0341460, -0.0129910
c, 1.9, -0.0001130, 0.0021590, -0.0586310, -0.0346400, 0.5239370, -1.3880530, 0.3582630, 2.2951440, -1.5928100
c, 1.9, -0.0004540, 0.0339630, -0.2396960, 0.2133340, 0.6897390, -3.4513310, 3.8753210, -1.6683870, -0.5242970
c, 1.9, -0.0001640, 0.0143460, -0.0837410, 0.0784280, 0.1569650, -0.3397950, -0.3239530, 0.3005360, 0.6094500
c, 10.10, 1.0000000
c, 11.11, 1.0000000
p, TC , 21.6444000, 13.5268000, 5.5426600, 1.5061100, 0.7616800, 0.3783660, 0.1635880, 0.0689410, 0.0286740, 0.0119000
c, 1.8, -0.0015590, 0.0237090, -0.1803710, 0.4355170, 0.4764540, 0.2128830, 0.0266430, 0.0001880
c, 1.8, -0.0000390, -0.0097340, 0.0897940, -0.2706050, -0.3378530, 0.0755710, 0.6858690, 0.3823930
c, 1.8, 0.0003340, -0.0181240, 0.1656770, -0.6480380, -0.5825740, 1.4399830, 0.1921730, -0.9459650
c, 1.8, -0.0000960, -0.0057220, 0.0539440, -0.1582580, -0.1873640, -0.0448460, 0.3427490, 0.5657040
c, 9.9, 1.0000000
c, 10.10, 1.0000000
d, TC , 25.3828000, 6.9853800, 2.4121700, 1.2417700, 0.6052420, 0.2828290, 0.1255600, 0.0514850, 0.0211000
c, 1.7, 0.0013390, -0.0132810, 0.1080380, 0.2860310, 0.3608340, 0.3011930, 0.1650010
c, 1.7, 0.0022390, -0.0238620, 0.3053790, 0.5904270, -0.3180930, -0.8455750, 0.3421110
c, 1.7, -0.0013560, 0.0137140, -0.1234670, -0.3501630, -0.2873210, 0.2197000, 0.5668830
c, 8.8, 1.0000000
c, 9.9, 1.0000000
f, TC , 1.362197252525E+00, 3.700496518902E-01
c, 1.1, 1.000000E+00
c, 2.2, 1.000000E+00

! ruthenium (11s,10p,9d,2f) -> [6s,6p,5d,2f]
s, RU , 208.2500000, 18.8471000, 11.7818000, 7.3619500, 4.2777100, 1.1910700, 0.5485680, 0.1386940, 0.0676510, 0.0301410, 0.0134000
c, 1.9, 0.0005230, -0.0449130, 0.2576260, -0.2091300, -0.5106620, 0.8032470, 0.4984140, 0.0337660, -0.0120940
c, 1.9, -0.0001820, 0.0052730, -0.0785570, -0.0138970, 0.5479870, -1.4980850, 0.4795000, 2.1402540, -1.4545610
c, 1.9, -0.0006350, 0.0517470, -0.3171890, 0.3298480, 0.6270530, -3.4222090, 3.8479480, -1.6239260, -0.6032230
c, 1.9, -0.0001750, 0.0148030, -0.0875270, 0.0842820, 0.1549110, -0.3381410, -0.3160050, 0.2835260, 0.6092730
c, 10.10, 1.0000000
c, 11.11, 1.0000000
p, RU , 22.7753000, 14.2340000, 5.9875500, 1.6392400, 0.8278800, 0.4090100, 0.1718770, 0.0712710, 0.0292260, 0.0120000
c, 1.8, -0.0015440, 0.0257710, -0.1865980, 0.4427740, 0.4781510, 0.2083690, 0.0246350, -0.0002190
c, 1.8, -0.0000630, -0.0114610, 0.0999930, -0.3036410, -0.3638860, 0.1659350, 0.7005450, 0.3189430
c, 1.8, -0.0001480, -0.0091740, 0.0813740, -0.2361250, -0.2943330, 0.0150980, 0.6560890, 0.4389440
c, 1.8, 0.0011030, -0.0225190, 0.1821610, -0.7313300, -0.4901170, 1.5235390, -0.0391820, -0.8661830
c, 9.9, 1.0000000
c, 10.10, 1.0000000
d, RU , 25.8838000, 7.5925500, 2.7129300, 1.3970800, 0.6825650, 0.3193780, 0.1417810, 0.0580170, 0.0237000
c, 1.7, 0.0015230, -0.0181620, 0.1096910, 0.2915970, 0.3620440, 0.2975140, 0.1610370
c, 1.7, 0.0026560, -0.0337600, 0.2929230, 0.6173710, -0.3625300, -0.8031580, 0.3154610
c, 1.7, -0.0015510, 0.0190350, -0.1257740, -0.3632850, -0.2793510, 0.2457120, 0.5472610
c, 8.8, 1.0000000
c, 9.9, 1.0000000
f, RU , 1.490939428533E+00, 3.916607038084E-01
c, 1.1, 1.000000E+00
c, 2.2, 1.000000E+00

```
! rhodium              (11s,10p,9d,2f) -> [6s,6p,5d,2f]
s, RH , 228.9320000, 19.9087000, 12.4468000, 7.7782100, 4.5800600, 1.2906800, 0.5934140, 0.1491580, 0.0719530, 0.0317520, 0.0140000
c, 1.9, 0.0005680, -0.0468030, 0.2723670, -0.2301680, -0.5095540, 0.8098500, 0.4978760, 0.0331540, -0.0115130
c, 1.9, -0.0002440, 0.0079290, -0.0938930, 0.0079360, 0.5418830, -1.4974880, 0.4948550, 1.9905270, -1.2260370
c, 1.9, -0.0001860, 0.0151800, -0.0911300, 0.0911990, 0.1503790, -0.3344630, -0.3074430, 0.2695540, 0.6048640
c, 1.9, -0.0007250, 0.0599300, -0.3549510, 0.3902970, 0.5857430, -3.3028560, 3.6257170, -1.2367490, -1.0070470
c, 10.10, 1.0000000
c, 11.11, 1.0000000
p, RH , 24.2117000, 15.1331000, 6.4472000, 1.7777600, 0.8990200, 0.4445220, 0.1886740, 0.0785140, 0.0321950, 0.0132000
c, 1.8, -0.0015790, 0.0271980, -0.1914630, 0.4476580, 0.4777240, 0.2058190, 0.0243520, -0.0000580
c, 1.8, 0.0001470, -0.0133530, 0.1094800, -0.3362590, -0.3686360, 0.2138540, 0.6803790, 0.3077610
c, 1.8, 0.0023040, -0.0272970, 0.1976470, -0.8063470, -0.3874520, 1.5168270, -0.1135820, -0.8286130
c, 1.8, -0.0001320, -0.0065320, 0.0564450, -0.1610630, -0.1842580, -0.0336100, 0.3445440, 0.5595730
c, 9.9, 1.0000000
c, 10.10, 1.0000000
d, RH , 29.4998000, 7.4043700, 3.0595900, 1.5820500, 0.7748340, 0.3630560, 0.1610470, 0.0656200, 0.0267000
c, 1.7, 0.0016770, -0.0184390, 0.1050760, 0.2937700, 0.3641780, 0.2972650, 0.1599580
c, 1.7, 0.0030290, -0.0362820, 0.2815510, 0.6384210, -0.3871850, -0.7962390, 0.3375180
c, 1.7, -0.0017860, 0.0201300, -0.1279690, -0.3807800, -0.2708410, 0.2651630, 0.5378200
c, 8.8, 1.0000000
c, 9.9, 1.0000000
f, RH , 1.751015538392E+00, 4.506921564904E-01
c, 1.1, 1.000000E+00
c, 2.2, 1.000000E+00

! palladium            (11s,10p,9d,2f) -> [6s,6p,5d,2f]
s, PD , 247.2020000, 20.7937000, 13.0019000, 8.1256200, 4.8884200, 1.3920600, 0.6383820, 0.1588280, 0.0757930, 0.0331840, 0.0145000
c, 1.9, 0.0006240, -0.0501980, 0.2962420, -0.2640150, -0.5037150, 0.8177430, 0.4964090, 0.0323180, -0.0110230
c, 1.9, -0.0002890, 0.0100490, -0.1055420, 0.0255180, 0.5196830, -1.4174220, 0.4318030, 1.8563460, -0.9626340
c, 1.9, -0.0002000, 0.0158670, -0.0968700, 0.1017160, 0.1434910, -0.3291740, -0.2996310, 0.2591500, 0.5993290
c, 1.9, -0.0008060, 0.0673350, -0.3972070, 0.4583510, 0.5570950, -3.2352070, 3.4344500, -0.8478430, -1.3772410
c, 10.10, 1.0000000
c, 11.11, 1.0000000
p, PD , 24.8824000, 15.5517000, 6.9234300, 1.9228600, 0.9711910, 0.4769190, 0.1938660, 0.0787920, 0.0316530, 0.0127000
c, 1.8, -0.0017220, 0.0315340, -0.2010620, 0.4525720, 0.4799470, 0.2040450, 0.0225170, -0.0006140
c, 1.8, 0.0002580, -0.0165210, 0.1208050, -0.3642750, -0.3807400, 0.2890590, 0.6824540, 0.2585140
c, 1.8, 0.0034320, -0.0345360, 0.2162500, -0.8692850, -0.2962990, 1.5226960, -0.2638220, -0.7598920
c, 1.8, -0.0002120, -0.0068010, 0.0539880, -0.1486710, -0.1667440, -0.0347260, 0.3263210, 0.5599760
c, 9.9, 1.0000000
c, 10.10, 1.0000000
d, PD , 29.0204000, 6.4114900, 3.9973600, 1.8874200, 0.9132080, 0.4256750, 0.1877270, 0.0757900, 0.0306000
c, 1.7, 0.0021720, -0.0479580, 0.1033430, 0.2932100, 0.3716870, 0.3064560, 0.1666290
c, 1.7, 0.0037610, -0.0967630, 0.2519190, 0.7039300, -0.3283750, -0.8381000, 0.3242530
c, 1.7, -0.0023520, 0.0544140, -0.1263090, -0.3977950, -0.2964330, 0.2617170, 0.5376890
c, 8.8, 1.0000000
c, 9.9, 1.0000000
f, PD , 2.051351106544E+00, 5.157984111307E-01
c, 1.1, 1.000000E+00
c, 2.2, 1.000000E+00
```

```
! silver              (11s,10p,9d,2f) -> [6s,6p,5d,2f]
s, AG , 222.6450000, 20.1725000, 12.7197000, 8.0021100, 5.0205300, 1.5208500, 0.6949200, 0.1729330, 0.0818970, 0.0351900, 0.0151000
c, 1.10, 0.0007000, -0.0798090, 0.4589790, -0.5218260, -0.4075440, 0.8414580, 0.4956480, 0.0276600, -0.0086430, 0.0035010
c, 1.10, -0.0002080, 0.0228050, -0.1373250, 0.1730850, 0.1057550, -0.3140630, -0.2985340, 0.2313240, 0.5969110, 0.3448480
c, 1.10, -0.0007140, 0.0726550, -0.4696600, 0.5845510, 0.5663560, -3.2222430, 3.1502630, -0.1517250, -1.9921310, 1.5740570
c, 1.10, -0.0002500, 0.0210250, -0.1682510, 0.1442000, 0.4139110, -1.1739400, 0.1871810, 1.7116850, -0.5350550, -0.8921690
c, 10.10, 1.0000000
c, 11.11, 1.0000000
p, AG , 22.0490000, 13.8356000, 7.6299400, 2.1037200, 1.0626200, 0.5193830, 0.2051990, 0.0829790, 0.0331440, 0.0132000
c, 1.9, -0.0058860, 0.0763570, -0.2661720, 0.4622010, 0.4894950, 0.2000890, 0.0193450, -0.0007170, 0.0003960
c, 1.9, 0.0022350, -0.0383630, 0.1454190, -0.3278470, -0.3817440, 0.2524550, 0.6915130, 0.2763110, 0.0092800
c, 1.9, 0.0087290, -0.0828090, 0.2865900, -0.8760860, -0.3443670, 1.5334530, -0.2330540, -0.7471730, 0.0010450
c, 1.9, 0.0005940, -0.0170800, 0.0679410, -0.1427200, -0.1683930, -0.0329600, 0.3267400, 0.5580540, 0.2652810
c, 9.9, 1.0000000
c, 10.10, 1.0000000
d, AG , 73.2665000, 18.7729000, 10.9237000, 3.0019700, 1.4717700, 0.6847640, 0.3003970, 0.1201550, 0.0481000
c, 1.8, 0.0004510, 0.0059840, -0.0189680, 0.1923860, 0.3721720, 0.3721880, 0.2300660, 0.0642420
c, 1.8, -0.0006060, -0.0075860, 0.0246380, -0.3135690, -0.5000300, 0.1211390, 0.5946850, 0.3049170
c, 1.8, 0.0004890, 0.0155170, -0.0470560, 0.6569190, 0.2238260, -1.1068170, 0.1657200, 0.7113670
c, 8.8, 1.0000000
c, 9.9, 1.0000000
f, AG , 2.472008729156E+00, 6.081910151618E-01
c, 1.1, 1.000000E+00
c, 2.2, 1.000000E+00

! cadmium             (11s,10p,9d,2f) -> [6s,6p,5d,2f]
s, CD , 257.4970000, 21.3546000, 13.4806000, 8.4865100, 5.3255100, 1.6268600, 0.7404690, 0.1809330, 0.0850960, 0.0363840, 0.0156000
c, 1.10, 0.0007680, -0.0781940, 0.4609280, -0.5362100, -0.4008230, 0.8553620, 0.4898680, 0.0220290, -0.0083600, 0.0019660
c, 1.10, -0.0002310, 0.0222790, -0.1382050, 0.1777840, 0.1056950, -0.3259540, -0.2993250, 0.2821000, 0.6020440, 0.2966350
c, 1.10, 0.0004270, -0.0241760, 0.2190290, -0.1984150, -0.5647670, 1.8215160, -0.8098690, -1.9098510, 1.4360880, 0.2772270
c, 1.10, 0.0014390, -0.1719290, 0.9499110, -1.5172470, 0.1055120, 3.3939830, -4.0474090, 2.4869710, -0.5824690, -0.6802540
c, 10.10, 1.0000000
c, 11.11, 1.0000000
p, CD , 243.1650000, 13.9588000, 8.3100100, 2.2020000, 1.1007200, 0.5359750, 0.2136540, 0.0854690, 0.0338230, 0.0134000
c, 1.9, 0.0001130, 0.0696720, -0.2536750, 0.4824920, 0.4845500, 0.1796400, 0.0152290, -0.0001880, 0.0002340
c, 1.9, -0.0000610, -0.0330520, 0.1255300, -0.3052730, -0.3223690, 0.1819080, 0.6863790, 0.3216860, 0.0073220
c, 1.9, -0.0000860, -0.0719040, 0.2712270, -0.9039590, -0.2760210, 1.4946140, -0.2070140, -0.7304040, 0.0051470
c, 1.9, -0.0000370, -0.0165070, 0.0636620, -0.1465210, -0.1561870, -0.0273550, 0.3226010, 0.5566980, 0.2700640
c, 9.9, 1.0000000
c, 10.10, 1.0000000
d, CD , 86.0666000, 22.6386000, 12.1384000, 3.4408100, 1.7176100, 0.8148490, 0.3660070, 0.1502820, 0.0617000
c, 1.8, 0.0004430, 0.0050060, -0.0160770, 0.1771520, 0.3666560, 0.3772260, 0.2339200, 0.0645830
c, 1.8, 0.0006330, 0.0073050, -0.0238720, 0.3340440, 0.5448850, -0.1745820, -0.6047310, -0.2492790
c, 1.8, -0.0003260, -0.0146770, 0.0459900, -0.6525580, -0.2130730, 1.1468110, -0.2158710, -0.7322260
c, 8.8, 1.0000000
c, 9.9, 1.0000000
f, CD , 3.034519970680E+00, 7.175298947884E-01
c, 1.1, 1.000000E+00
c, 2.2, 1.000000E+00
```

```
! hafnium              (11s,10p,9d,2f) -> [6s,6p,5d,2f]
s, HF , 26.5042000, 16.5840000, 10.3831000, 6.4955000, 3.4643100, 0.9862000, 0.4578280, 0.1321180, 0.0669670, 0.0290100, 0.0126000
c, 1.9, 0.0320170, -0.2583380, 0.7419580, -0.5972460, -0.4674330, 0.7814380, 0.5337560, 0.0363800, -0.0136480
c, 1.9, -0.0051870, 0.0780690, -0.2633410, 0.1631730, 0.4121450, -0.9279190, -0.3552850, 2.5901110, -1.2877130
c, 1.9, -0.0092200, 0.0766750, -0.2256410, 0.1855680, 0.1565490, -0.3114170, -0.3945230, 0.2034830, 0.6862960
c, 1.9, 0.0266200, -0.0510920, -0.0133860, -0.2493960, 1.1377230, -3.2919740, 3.0952960, 0.5233810, -2.9817690
c, 10.10, 1.0000000
c, 11.11, 1.0000000
p, HF , 15.2724000, 9.5703500, 5.0361800, 1.1521500, 0.5624010, 0.2742040, 0.1205050, 0.0528120, 0.0228910, 0.0099000
c, 1.8, -0.0249160, 0.1349670, -0.3145280, 0.4766040, 0.4841910, 0.1844940, 0.0186390, 0.0015040
c, 1.8, 0.0094170, -0.0530870, 0.1282870, -0.2170080, -0.3251220, -0.0046510, 0.7343430, 0.3849980
c, 1.8, 0.0190460, -0.1039530, 0.2510270, -0.5289630, -0.8006570, 1.6510980, 0.1195080, -0.9590230
c, 1.8, 0.0066140, -0.0369260, 0.0887770, -0.1514170, -0.2041520, -0.0401350, 0.3663260, 0.5626960
c, 9.9, 1.0000000
c, 10.10, 1.0000000
d, HF , 11.9756000, 7.4799800, 4.6738300, 1.1750300, 0.5283500, 0.2277570, 0.0941690, 0.0366310, 0.0142000
c, 1.7, -0.0014780, 0.0187080, -0.0580870, 0.1823340, 0.3486640, 0.3933500, 0.2772230
c, 1.7, -0.0031330, 0.0334770, -0.1038090, 0.4716820, 0.5191890, -1.0917460, 0.0084120
c, 1.7, 0.0012390, -0.0202160, 0.0669450, -0.2392560, -0.4736770, -0.0843030, 0.6333300
c, 8.8, 1.0000000
c, 9.9, 1.0000000
f, HF , 5.298464147603E-01, 1.610386050695E-01
c, 1.1, 1.000000E+00
c, 2.2, 1.000000E+00

! tantalum             (11s,10p,9d,2f) -> [6s,6p,5d,2f]
s, TA , 27.1236000, 16.9695000, 10.6228000, 6.6449300, 3.5948000, 1.0504400, 0.4928350, 0.1487360, 0.0722280, 0.0309970, 0.0133000
c, 1.9, 0.0310530, -0.2562460, 0.7537040, -0.6221810, -0.4709360, 0.7957440, 0.5320760, 0.0359520, -0.0130440
c, 1.9, -0.0036940, 0.0741990, -0.2667320, 0.1638740, 0.4542910, -1.0387690, -0.2840230, 2.4631730, -1.1724650
c, 1.9, -0.0090380, 0.0776200, -0.2351160, 0.1985680, 0.1643530, -0.3367730, -0.3948460, 0.2230040, 0.6886390
c, 1.9, 0.0199390, -0.0119830, -0.1263190, -0.1072520, 1.0858150, -3.3698120, 3.2164070, 0.3284360, -2.7861580
c, 10.10, 1.0000000
c, 11.11, 1.0000000
p, TA , 15.5273000, 9.7259300, 5.2380300, 1.2270200, 0.6053810, 0.2963140, 0.1305110, 0.0571160, 0.0246790, 0.0107000
c, 1.8, -0.0247840, 0.1412710, -0.3273280, 0.4834390, 0.4839520, 0.1812620, 0.0183560, 0.0014010
c, 1.8, 0.0100470, -0.0601510, 0.1452770, -0.2448290, -0.3637170, 0.0650640, 0.7639420, 0.3237320
c, 1.8, 0.0206680, -0.1177830, 0.2832870, -0.6451770, -0.6698480, 1.6952860, -0.0513130, -0.8985720
c, 1.8, 0.0068180, -0.0402940, 0.0966590, -0.1633710, -0.2142180, -0.0232540, 0.3814550, 0.5524390
c, 9.9, 1.0000000
c, 10.10, 1.0000000
d, TA , 12.1917000, 7.6161300, 4.7595400, 1.2759000, 0.5929460, 0.2642870, 0.1124950, 0.0447800, 0.0178000
c, 1.7, -0.0017560, 0.0229090, -0.0715900, 0.1997750, 0.3628730, 0.3846300, 0.2504110
c, 1.7, -0.0034800, 0.0408190, -0.1314870, 0.5878110, 0.4024980, -1.1685840, 0.1607410
c, 1.7, 0.0014680, -0.0248100, 0.0829610, -0.2725710, -0.4890720, -0.0016350, 0.6453820
c, 8.8, 1.0000000
c, 9.9, 1.0000000
f, TA , 6.396759164402E-01, 2.009185336882E-01
c, 1.1, 1.000000E+00
c, 2.2, 1.000000E+00
```

```
! tungsten              (11s,10p,9d,2f) -> [6s,6p,5d,2f]
s, W , 28.3881000, 17.7568000, 11.1120000, 6.9493400, 3.8150600, 1.1124900, 0.5254010, 0.1615290, 0.0764090, 0.0326700, 0.0140000
c, 1.9, 0.0286980, -0.2410370, 0.7152180, -0.5713300, -0.5072560, 0.8128760, 0.5252410, 0.0346380, -0.0129350
c, 1.9, -0.0018080, 0.0639220, -0.2421830, 0.1239880, 0.5003090, -1.1225650, -0.1993080, 2.3607790, -1.1023500
c, 1.9, -0.0084840, 0.0745440, -0.2285180, 0.1874400, 0.1806120, -0.3615620, -0.3873630, 0.2480770, 0.6828620
c, 1.9, 0.0138380, 0.0202260, -0.2093720, -0.0130360, 1.0461320, -3.4415170, 3.3512290, 0.1364630, -2.6080750
c, 10.10, 1.0000000
c, 11.11, 1.0000000
p, W , 16.5554000, 10.3744000, 5.5219000, 1.2890200, 0.6355960, 0.3096450, 0.1370120, 0.0599380, 0.0258750, 0.0112000
c, 1.8, -0.0252630, 0.1409610, -0.3299160, 0.5017200, 0.4805660, 0.1689050, 0.0157270, 0.0014690
c, 1.8, 0.0109680, -0.0640750, 0.1564780, -0.2790200, -0.3897030, 0.1566280, 0.7687580, 0.2655370
c, 1.8, 0.0221780, -0.1230550, 0.2995730, -0.7580510, -0.4980530, 1.7176990, -0.2467290, -0.8204790
c, 1.8, 0.0071290, -0.0411130, 0.0996190, -0.1760570, -0.2167410, -0.0011430, 0.3928970, 0.5406250
c, 9.9, 1.0000000
c, 10.10, 1.0000000
d, W , 13.6383000, 8.5173400, 5.3232800, 1.3931900, 0.6543070, 0.2943900, 0.1257220, 0.0497170, 0.0197000
c, 1.7, -0.0026060, 0.0290880, -0.0831780, 0.2105050, 0.3794490, 0.3825380, 0.2279060
c, 1.7, 0.0068120, -0.0594810, 0.1659640, -0.7073810, -0.2548900, 1.2651180, -0.4506990
c, 1.7, 0.0025740, -0.0333260, 0.1006620, -0.3117890, -0.5101020, 0.1043210, 0.6647930
c, 8.8, 1.0000000
c, 9.9, 1.0000000
f, W , 7.027000751780E-01, 2.177208393873E-01
c, 1.1, 1.000000E+00
c, 2.2, 1.000000E+00

! rhenium               (11s,10p,9d,2f) -> [6s,6p,5d,2f]
s, RE , 30.4120000, 19.0142000, 11.8921000, 7.4336100, 4.1811300, 1.1685700, 0.5539770, 0.1707810, 0.0792870, 0.0339090, 0.0145000
c, 1.9, 0.0229870, -0.1976360, 0.5813760, -0.3840040, -0.6002800, 0.8264270, 0.5102290, 0.0365510, -0.0087660
c, 1.9, 0.0008190, 0.0437380, -0.1772190, 0.0197240, 0.5658650, -1.1960450, -0.0926010, 2.2639340, -1.0605870
c, 1.9, -0.0072090, 0.0645740, -0.1963520, 0.1354270, 0.2175540, -0.3902690, -0.3723360, 0.2672200, 0.6716710
c, 1.9, -0.0059360, -0.0062770, 0.0754730, 0.0512310, -0.5040200, 1.6252170, -1.6511890, 0.1479060, 0.9643860
c, 10.10, 1.0000000
c, 11.11, 1.0000000
p, RE , 18.3488000, 11.4877000, 5.4325600, 1.3785900, 0.6887660, 0.3380830, 0.1491600, 0.0643210, 0.0273440, 0.0116000
c, 1.8, -0.0168770, 0.0929150, -0.2914010, 0.4982270, 0.4796070, 0.1752920, 0.0173410, 0.0013480
c, 1.8, 0.0073690, -0.0437140, 0.1463200, -0.3009630, -0.4000020, 0.1900900, 0.7604950, 0.2562560
c, 1.8, 0.0150340, -0.0837840, 0.2804860, -0.8342650, -0.4202230, 1.7104440, -0.3011560, -0.7920800
c, 1.8, 0.0046080, -0.0268720, 0.0889710, -0.1794810, -0.2111050, -0.0043780, 0.3822050, 0.5438540
c, 9.9, 1.0000000
c, 10.10, 1.0000000
d, RE , 13.8248000, 8.6358600, 5.3969500, 1.4943200, 0.7134790, 0.3249100, 0.1398360, 0.0555530, 0.0221000
c, 1.7, -0.0010140, 0.0200690, -0.0709260, 0.2136340, 0.3740960, 0.3691180, 0.2324990
c, 1.7, -0.0008920, 0.0318260, -0.1289560, 0.6891180, 0.2530110, -1.1068680, 0.1625610
c, 1.7, 0.0014410, -0.0233020, 0.0813920, -0.2826220, -0.4844350, 0.0772300, 0.6339580
c, 8.8, 1.0000000
c, 9.9, 1.0000000
f, RE , 8.190492179877E-01, 2.637606473324E-01
c, 1.1, 1.000000E+00
c, 2.2, 1.000000E+00
```

```
! osmium                (11s,10p,9d,2f) -> [6s,6p,5d,2f]
s, OS , 32.3055000, 20.1934000, 12.6258000, 7.8902100, 4.4618300, 1.2361300, 0.5888300, 0.1832610, 0.0839720, 0.0358740, 0.0153000
c, 1.9, 0.0197370, -0.1722810, 0.5020590, -0.2729310, -0.6628560, 0.8403110, 0.5041200, 0.0360460, -0.0077430
c, 1.9, 0.0032420, 0.0321220, -0.1472230, -0.0521880, 0.6858550, -1.4787110, 0.1833530, 2.2357940, -1.2321000
c, 1.9, -0.0065050, 0.0585150, -0.1762530, 0.1041850, 0.2405050, -0.4093240, -0.3607630, 0.2734870, 0.6639500
c, 1.9, 0.0069550, 0.0417920, -0.2181670, -0.1049580, 1.2105800, -4.1259100, 4.5902800, -1.6062050, -0.9747310
c, 10.10, 1.0000000
c, 11.11, 1.0000000
p, OS , 19.5631000, 12.2451000, 5.5534200, 1.4628400, 0.7345370, 0.3605750, 0.1566910, 0.0663330, 0.0277460, 0.0116000
c, 1.8, -0.0147080, 0.0813650, -0.2865920, 0.5046710, 0.4803480, 0.1720070, 0.0167790, 0.0010290
c, 1.8, 0.0068020, -0.0412110, 0.1569900, -0.3433910, -0.4303710, 0.2960230, 0.7536120, 0.1966590
c, 1.8, 0.0137510, -0.0773820, 0.2953390, -0.9653980, -0.2465530, 1.7281840, -0.5259660, -0.6793560
c, 1.8, 0.0038110, -0.0227510, 0.0856230, -0.1797460, -0.2015160, -0.0014850, 0.3717870, 0.5436820
c, 9.9, 1.0000000
c, 10.10, 1.0000000
d, OS , 14.6857000, 9.1712400, 5.7299200, 1.5875600, 0.7672690, 0.3528390, 0.1530360, 0.0612280, 0.0245000
c, 1.7, -0.0002690, 0.0164940, -0.0666740, 0.2245470, 0.3796600, 0.3618810, 0.2216830
c, 1.7, 0.0026700, -0.0374650, 0.1404650, -0.8231180, -0.0536040, 1.1529670, -0.4177890
c, 1.7, 0.0012700, -0.0225880, 0.0838610, -0.3263310, -0.5066390, 0.1706070, 0.6360850
c, 8.8, 1.0000000
c, 9.9, 1.0000000
f, OS , 8.663233099262E-01, 2.743721332960E-01
c, 1.1, 1.000000E+00
c, 2.2, 1.000000E+00

! iridium               (11s,10p,9d,2f) -> [6s,6p,5d,2f]
s, IR , 34.2048000, 21.3768000, 13.3624000, 8.3483500, 4.7316300, 1.3078500, 0.6243340, 0.1938710, 0.0879490, 0.0375390, 0.0160000
c, 1.9, 0.0173300, -0.1532030, 0.4419330, -0.1886920, -0.7122250, 0.8529050, 0.4997460, 0.0352490, -0.0070780
c, 1.9, 0.0057000, 0.0186860, -0.1098820, -0.1262300, 0.7876390, -1.7027670, 0.4383340, 2.1004080, -1.2318660
c, 1.9, -0.0060010, 0.0538850, -0.1606020, 0.0803950, 0.2575940, -0.4237510, -0.3483540, 0.2800310, 0.6545570
c, 1.9, -0.0001450, 0.0001550, 0.0004580, 0.0027310, -0.0111600, 0.0322630, -0.0309800, -0.0019260, 0.0244470
c, 10.10, 1.0000000
c, 11.11, 1.0000000
p, IR , 20.7204000, 12.9677000, 5.7222400, 1.5503800, 0.7826600, 0.3855150, 0.1706090, 0.0728290, 0.0305710, 0.0128000
c, 1.8, -0.0132020, 0.0739520, -0.2843120, 0.5102230, 0.4795020, 0.1697720, 0.0166680, 0.0012420
c, 1.8, 0.0068620, -0.0422290, 0.1771470, -0.4201130, -0.4381970, 0.4034530, 0.7048150, 0.1692730
c, 1.8, 0.0121460, -0.0723020, 0.3121050, -1.1249840, 0.0092840, 1.6533010, -0.6527860, -0.6166420
c, 1.8, 0.0035200, -0.0215200, 0.0890920, -0.1924180, -0.2089670, 0.0046730, 0.3828460, 0.5399630
c, 9.9, 1.0000000
c, 10.10, 1.0000000
d, IR , 91.4125000, 9.7301300, 6.0771200, 1.6882000, 0.8237860, 0.3820680, 0.1669650, 0.0672310, 0.0271000
c, 1.7, 0.0000240, 0.0152170, -0.0657240, 0.2340050, 0.3845350, 0.3558490, 0.2119760
c, 1.7, -0.0000080, 0.0306140, -0.1301790, 0.7766970, 0.1046450, -1.0602650, 0.1697160
c, 1.7, -0.0000130, -0.0183130, 0.0780220, -0.3254460, -0.4793950, 0.1391480, 0.5831340
c, 8.8, 1.0000000
c, 9.9, 1.0000000
f, IR , 9.648060097215E-01, 3.151011585706E-01
c, 1.1, 1.000000E+00
c, 2.2, 1.000000E+00
```

```
! platinum              (11s,10p,9d,2f) -> [6s,6p,5d,2f]
s, PT , 36.0064000, 22.4991000, 14.0609000, 8.7816100, 4.9923800, 1.3838500, 0.6616170, 0.2048820, 0.0922300, 0.0392640, 0.0167000
c, 1.9, 0.0156030, -0.1396000, 0.3994690, -0.1277440, -0.7514510, 0.8633270, 0.4975940, 0.0347580, -0.0065890
c, 1.9, 0.0070370, 0.0105250, -0.0854980, -0.1765590, 0.8567070, -1.8442100, 0.6011560, 1.9822930, -1.1764800
c, 1.9, -0.0114450, 0.1220440, -0.3759060, 0.0553880, 1.1335870, -4.2149190, 4.8759410, -2.1659360, -0.4268040
c, 1.9, -0.0056780, 0.0507550, -0.1500300, 0.0639820, 0.2704110, -0.4346570, -0.3372900, 0.2814460, 0.6455790
c, 10.10, 1.0000000
c, 11.11, 1.0000000
p, PT , 21.5986000, 13.5160000, 5.9203700, 1.6412600, 0.8311310, 0.4084300, 0.1754270, 0.0726850, 0.0297610, 0.0122000
c, 1.8, -0.0125150, 0.0715700, -0.2872760, 0.5154190, 0.4804610, 0.1679960, 0.0158600, 0.0007950
c, 1.8, 0.0067690, -0.0429400, 0.1894550, -0.4606470, -0.4463350, 0.4896840, 0.6825880, 0.1279470
c, 1.8, 0.0116510, -0.0715220, 0.3259640, -1.2148310, 0.1587270, 1.5953330, -0.7779330, -0.5304420
c, 1.8, 0.0029600, -0.0191100, 0.0838980, -0.1830090, -0.1878840, -0.0017870, 0.3606710, 0.5449210
c, 9.9, 1.0000000
c, 10.10, 1.0000000
d, PT , 72.7562000, 10.4742000, 6.2509000, 1.8018500, 0.8884540, 0.4156650, 0.1830800, 0.0742330, 0.0301000
c, 1.7, 0.0000520, 0.0126260, -0.0632410, 0.2396470, 0.3876720, 0.3517440, 0.2052900
c, 1.7, -0.0000260, 0.0271890, -0.1307380, 0.8378570, 0.0073940, -1.0612590, 0.2490050
c, 1.7, -0.0000370, -0.0157890, 0.0782970, -0.3582960, -0.4793250, 0.1884070, 0.5781450
c, 8.8, 1.0000000
c, 9.9, 1.0000000
f, PT , 1.056233651616E+00, 3.382517093687E-01
c, 1.1, 1.000000E+00
c, 2.2, 1.000000E+00

! gold              (11s,10p,9d,2f) -> [6s,6p,5d,2f]
s, AU , 36.0126000, 22.5645000, 14.1620000, 8.8548700, 5.0607200, 1.4740500, 0.7049130, 0.2177870, 0.0974080, 0.0412140, 0.0174000
c, 1.10, 0.0190290, -0.1678000, 0.4966770, -0.2662560, -0.6998980, 0.8731080, 0.5025820, 0.0321960, -0.0055030, 0.0025190
c, 1.10, -0.0067770, 0.0600080, -0.1833280, 0.1147190, 0.2488220, -0.4376710, -0.3311080, 0.2741810, 0.6377380, 0.3092360
c, 1.10, 0.0039090, 0.0337600, -0.1660600, -0.0517890, 0.7950230, -1.8059270, 0.5612390, 1.8784270, -0.9692290, -0.5756350
c, 1.10, -0.0119140, 0.1330690, -0.4342870, 0.1701570, 1.0647560, -4.0055360, 4.4028320, -1.4321630, -1.1432550, 1.3908690
c, 10.10, 1.0000000
c, 11.11, 1.0000000
p, AU , 21.5954000, 13.5145000, 6.2551400, 1.7181400, 0.8694390, 0.4267590, 0.1842970, 0.0763790, 0.0312200, 0.0128000
c, 1.9, -0.0132180, 0.0801960, -0.2966860, 0.5289590, 0.4788890, 0.1576200, 0.0138970, 0.0008350, -0.0000290
c, 1.9, 0.0070860, -0.0474930, 0.1918930, -0.4636370, -0.4351380, 0.4944030, 0.6751140, 0.1315360, 0.0024710
c, 1.9, 0.0129200, -0.0820290, 0.3386190, -1.2549920, 0.2258130, 1.5758790, -0.8043540, -0.5073130, 0.0102040
c, 1.9, 0.0030800, -0.0214060, 0.0867760, -0.1897740, -0.1858350, 0.0047800, 0.3638360, 0.5417640, 0.2345610
c, 9.9, 1.0000000
c, 10.10, 1.0000000
d, AU , 87.0208000, 11.0668000, 6.6604700, 1.9469600, 0.9847060, 0.4746550, 0.2165580, 0.0914720, 0.0386000
c, 1.8, 0.0000440, 0.0142880, -0.0688760, 0.2395590, 0.3873230, 0.3487560, 0.1984920, 0.0498790
c, 1.8, -0.0000310, -0.0199590, 0.0953200, -0.4213430, -0.5069330, 0.2854660, 0.5825650, 0.2194620
c, 1.8, -0.0000440, 0.0350830, -0.1619420, 1.0421550, -0.2648650, -1.1163050, 0.5457620, 0.4996220
c, 8.8, 1.0000000
c, 9.9, 1.0000000
f, AU , 1.151303964097E+00, 3.765823835975E-01
c, 1.1, 1.000000E+00
c, 2.2, 1.000000E+00
```

```
! mercury              (11s,10p,9d,2f) -> [6s,6p,5d,2f]
s, HG , 98.3058000, 19.6406000, 12.3397000, 7.7478200, 4.8629100, 1.5771900, 0.7529260, 0.2351120, 0.1039680, 0.0434000, 0.0181000
c, 1.10, 0.0010330, -0.1063820, 0.5320730, -0.5223240, -0.5373210, 0.8847880, 0.5138340, 0.0294070, -0.0061590, 0.0013840
c, 1.10, -0.0003560, 0.0379090, -0.2000130, 0.2240930, 0.1793740, -0.4508330, -0.3390550, 0.2934140, 0.6446280, 0.2916260
c, 1.10, -0.0002170, -0.0440670, 0.2876950, -0.1894760, -0.7807040, 2.1756040, -0.8865840, -1.8749170, 1.2470530, 0.3523510
c, 1.10, -0.0020930, 0.1557830, -0.7951410, 0.9970540, 0.5928610, -4.4071970, 5.2898370, -2.8866330, 0.3158740, 0.8226100
c, 10.10, 1.0000000
c, 11.11, 1.0000000
p, HG , 21.4065000, 13.3929000, 6.5102100, 1.8222200, 0.9265070, 0.4550820, 0.1927600, 0.0786890, 0.0317290, 0.0128000
c, 1.9, -0.0128370, 0.0865310, -0.3080410, 0.5327940, 0.4822070, 0.1546950, 0.0132010, 0.0005850, 0.0000200
c, 1.9, 0.0061400, -0.0480560, 0.1891060, -0.4381190, -0.4321930, 0.4570570, 0.6953570, 0.1420160, 0.0020300
c, 1.9, 0.0130140, -0.0890970, 0.3480200, -1.2184470, 0.1232280, 1.6284380, -0.7824510, -0.5112540, 0.0165110
c, 1.9, 0.0026690, -0.0218770, 0.0865390, -0.1847680, -0.1773950, 0.0009770, 0.3536030, 0.5435820, 0.2455390
c, 9.9, 1.0000000
c, 10.10, 1.0000000
d, HG , 86.9881000, 11.9913000, 6.9364800, 2.0864100, 1.0766400, 0.5311530, 0.2488080, 0.1081700, 0.0470000
c, 1.8, 0.0000580, 0.0127790, -0.0689630, 0.2415630, 0.3878580, 0.3454410, 0.1915090, 0.0465020
c, 1.8, 0.0000500, 0.0192460, -0.1034870, 0.4847690, 0.5069510, -0.3625720, -0.5729800, -0.1666370
c, 1.8, -0.0000430, 0.0337600, -0.1746100, 1.2017020, -0.5030930, -1.1299990, 0.7749310, 0.3655340
c, 8.8, 1.0000000
c, 9.9, 1.0000000
f, HG , 1.264532512485E+00, 4.178111252902E-01
c, 1.1, 1.000000E+00
c, 2.2, 1.000000E+0
```

cc-pVTZ-F12-wis Basis Set: Sc-Zn Atoms

```
! scandium           (23s,19p,12d,3f,2g) -> [9s,8p,6d,3f,2g]
s, SC , 8.417909E+06, 1.260312E+06, 2.867984E+05, 8.123469E+04, 2.650311E+04, 9.568701E+03, 3.732497E+03, 1.548422E+03, 6.755420E+02, 3.072522E+02, 1.446672E+02,
        7.004387E+01, 3.432250E+01, 1.561224E+01, 7.743722E+00, 3.844260E+00, 1.719766E+00, 8.325230E-01, 3.855640E-01, 9.670000E-02, 4.777800E-02, 2.236300E-02, 0.0104700
c, 1.22, 1.981292E-06, 1.540697E-05, 8.106065E-05, 3.423943E-04, 1.246990E-03, 4.056425E-03, 1.200596E-02, 3.235944E-02, 7.819972E-02, 1.617396E-01, 2.618480E-01,
        2.748284E-01, 1.419647E-01, 7.046176E-02, 1.288392E-01, 9.269352E-02, 1.229949E-02, -4.588239E-04, 1.722703E-04, -5.410745E-05, 4.892875E-05, -9.555437E-06
c, 1.22, -1.147926E-06, -8.929259E-06, -4.696382E-05, -1.985315E-04, -7.230932E-04, -2.358822E-03, -7.008148E-03, -1.912407E-02, -4.728922E-02, -1.032480E-01, -1.861960E-01,
        -2.480547E-01, -1.614781E-01, 1.779183E-01, 5.324076E-01, 3.752830E-01, 5.198123E-02, -4.029053E-03, 3.794092E-04, -4.006692E-04, 3.219248E-04, -7.690632E-05
c, 1.22, 2.221960E-07, 1.729799E-06, 9.088001E-06, 3.848622E-05, 1.399997E-04, 4.585975E-04, 1.364588E-03, 3.773739E-03, 9.493887E-03, 2.174611E-02, 4.228528E-02,
        6.540610E-02, 4.759732E-02, -8.160330E-02, -3.283980E-01, -3.654561E-01, 2.102218E-01, 6.856793E-01, 3.684308E-01, 2.050798E-02, -6.410776E-03, 3.021766E-03
c, 1.22, -5.350285E-08, -4.164419E-07, -2.188499E-06, -9.264628E-06, -3.371711E-05, -1.103886E-04, -3.287051E-04, -9.084250E-04, -2.288204E-03, -5.238462E-03, -1.021547E-02,
        -1.581707E-02, -1.162945E-02, 2.013432E-02, 8.405086E-02, 9.848250E-02, -6.838523E-02, -2.353627E-01, -2.776984E-01, 2.987423E-01, 6.193723E-01, 2.563270E-01
c, 1.22, -9.140993E-08, -7.270775E-07, -3.702449E-06, -1.633468E-05, -5.642360E-05, -1.968190E-04, -5.428811E-04, -1.642692E-03, -3.709752E-03, -9.699229E-03, -1.589350E-02,
        -3.149937E-02, -1.153920E-02, 1.426789E-02, 1.977719E-01, 9.493216E-02, 2.678950E-02, -9.351082E-01, 1.820725E-01, 2.077423E+00, -1.113944E+00, -6.910434E-01
c, 22.22, 1.000000E+00
c, 1.22, -1.445075E-07, -1.167681E-06, -5.810201E-06, -2.641620E-05, -8.781407E-05, -3.207912E-04, -8.363750E-04, -2.703640E-03, -5.633960E-03, -1.622651E-02, -2.336426E-02,
        -5.523292E-02, -8.807771E-03, -2.060869E-04, 3.882857E-01, 9.743430E-02, 6.858569E-03, -2.321570E+00, 2.666580E+00, 1.898281E-01, -2.684065E+00, 2.043701E+00
c, 1.22, -1.607992E-07, -1.216461E-06, -6.659894E-06, -2.670499E-05, -1.039947E-04, -3.133995E-04, -1.029884E-03, -2.530633E-03, -7.335917E-03, -1.414587E-02, -3.451496E-02,
        -3.859251E-02, -5.560506E-02, 1.025062E-01, 2.265527E-01, 6.017289E-01, -1.402974E+00, -8.478364E-01, 3.094789E+00, -5.792733E+00, 6.944158E+00, -3.006484E+00
        c, 23.23, 1.0000000
p, SC , 2.264097E+04, 5.357545E+03, 1.740373E+03, 6.666163E+02, 2.836384E+02, 1.297976E+02, 6.267956E+01, 3.150441E+01, 1.623525E+01, 8.518324E+00, 4.519005E+00,
        2.366591E+00, 1.215235E+00, 6.143480E-01, 3.007450E-01, 1.260040E-01, 5.484000E-02, 2.355700E-02, 0.0101200
c, 1.18, 1.200000E-05, 1.070000E-04, 6.250000E-04, 2.830000E-03, 1.053000E-02, 3.288100E-02, 8.588400E-02, 1.800300E-01, 2.892240E-01, 3.279100E-01, 2.101980E-01,
        5.468000E-02, 1.853000E-03, -1.417000E-03, -9.530000E-04, -5.100000E-05, -2.100000E-05, 4.000000E-06
c, 1.18, -4.000000E-06, -3.500000E-05, -2.050000E-04, -9.320000E-04, -3.484000E-03, -1.101900E-02, -2.941500E-02, -6.371300E-02, -1.060850E-01, -1.286380E-01, -5.301200E-02,
        1.740260E-01, 3.925500E-01, 3.965140E-01, 1.765440E-01, 1.911100E-02, 7.500000E-05, 3.040000E-04
c, 1.18, 1.000000E-06, 1.000000E-05, 6.000000E-05, 2.740000E-04, 1.025000E-03, 3.250000E-03, 8.674000E-03, 1.886600E-02, 3.146600E-02, 3.866300E-02, 1.492300E-02,
        -5.967000E-02, -1.347060E-01, -1.728090E-01, -8.069800E-02, 5.542640E-01, 5.500070E-01, 3.853300E-02
c, 1.18, 2.000000E-06, 2.000000E-05, 1.280000E-04, 5.340000E-04, 2.164000E-03, 6.307000E-03, 1.837700E-02, 3.601600E-02, 6.943000E-02, 7.194900E-02, 5.778900E-02,
        -1.752840E-01, -3.373290E-01, -5.644270E-01, 1.163380E+00, 5.658350E-01, -9.715060E-01, -8.536100E-02
c, 1.18, -2.000000E-06, -2.900000E-05, -1.300000E-04, -7.520000E-04, -2.212000E-03, -8.893000E-03, -1.826200E-02, -5.304100E-02, -5.993900E-02, -1.556420E-01, 9.572000E-03,
        9.189800E-02, 1.256710E+00, -1.011115E+00, -1.368836E+00, 2.377782E+00, -1.153984E+00, -2.094090E-01
c, 1.18, 1.000000E-06, 9.000000E-06, 5.000000E-05, 2.270000E-04, 8.530000E-04, 2.686000E-03, 7.225000E-03, 1.559000E-02, 2.630300E-02, 3.177700E-02, 1.248300E-02,
        -5.262700E-02, -1.140250E-01, -1.463710E-01, -1.386300E-02, 3.524440E-01, 5.504540E-01, 2.261050E-01
c, 18.18, 1.000000E+00
c, 19.19, 1.0000000
d, SC , 1.454680E+02, 4.341880E+01, 1.657680E+01, 6.971110E+00, 3.133470E+00, 1.457680E+00, 6.745380E-01, 3.051620E-01, 1.341130E-01, 5.727000E-02, 2.335400E-02, 0.0095200
c, 1.11, 4.940000E-04, 4.198000E-03, 1.896700E-02, 5.840800E-02, 1.376050E-01, 2.314700E-01, 2.965300E-01, 3.052000E-01, 2.380750E-01, 1.073860E-01, 1.173300E-02
c, 1.11, -5.050000E-04, -4.293000E-03, -1.947500E-02, -6.063100E-02, -1.436610E-01, -2.292940E-01, -2.619730E-01, -5.300600E-02, 4.119990E-01, 4.831900E-01, 1.071880E-01
c, 1.11, 8.360000E-04, 8.495000E-03, 3.348100E-02, 1.270110E-01, 2.482810E-01, 4.455630E-01, -5.406450E-01, -8.783940E-01, 1.443118E+00, -4.690810E-01, -3.989850E-01
c, 1.11, -6.670000E-04, -5.593000E-03, -2.599000E-02, -7.914000E-02, -1.936220E-01, -2.899810E-01, -1.702510E-01, 6.126870E-01, 4.817940E-01, -6.794480E-01, -2.771730E-01
c, 11.11, 1.000000E+00
c, 12.12, 1.0000000
f, SC , 1.357094217513E+00, 3.750507169498E-01, 1.153692315775E-01
c, 1.1, 1.000000E+00
c, 2.2, 1.000000E+00
c, 3.3, 1.000000E+00
g, SC , 5.099175975749E-01, 1.585988376112E-01
c, 1.1, 1.000000E+00
c, 2.2, 1.000000E+00
```

```
! titanium             (23s,19p,12d,3f,2g) -> [9s,8p,6d,3f,2g]
s, TI , 9.317034E+06, 1.394971E+06, 3.174531E+05, 8.992100E+04, 2.933816E+04, 1.059266E+04, 4.132056E+03, 1.714235E+03, 7.479151E+02, 3.401991E+02, 1.602060E+02,
        7.758851E+01, 3.804489E+01, 1.735362E+01, 8.642062E+00, 4.304404E+00, 1.937718E+00, 9.365940E-01, 4.326240E-01, 1.073520E-01, 5.203400E-02, 2.403800E-02, 0.0111000
c, 1.22, 1.967884E-06, 1.530198E-05, 8.050403E-05, 3.400302E-04, 1.238349E-03, 4.028475E-03, 1.192508E-02, 3.215601E-02, 7.778620E-02, 1.612030E-01, 2.618826E-01,
        2.766541E-01, 1.442968E-01, 6.993131E-02, 1.259449E-01, 9.025830E-02, 1.200401E-02, -3.976168E-04, 1.425444E-04, -4.196664E-05, 4.824213E-05, -4.676898E-06
c, 1.22, -1.130007E-06, -8.789416E-06, -4.622623E-05, -1.954047E-04, -7.117000E-04, -2.321755E-03, -6.899511E-03, -1.883765E-02, -4.663976E-02, -1.020764E-01, -1.848693E-01,
        -2.479601E-01, -1.634863E-01, 1.779147E-01, 5.351738E-01, 3.752822E-01, 5.217038E-02, -3.863192E-03, 2.845374E-04, -3.500414E-04, 3.192906E-04, -5.613970E-05
c, 1.22, 2.252735E-07, 1.753631E-06, 9.213052E-06, 3.901242E-05, 1.419184E-04, 4.648635E-04, 1.383544E-03, 3.827354E-03, 9.639191E-03, 2.211719E-02, 4.314954E-02,
        6.703481E-02, 4.946424E-02, -8.333456E-02, -3.384435E-01, -3.674069E-01, 2.247056E-01, 6.878815E-01, 3.594435E-01, 1.933695E-02, -5.997819E-03, 2.828064E-03
c, 1.22, -5.318433E-08, -4.139855E-07, -2.175153E-06, -9.209567E-06, -3.350761E-05, -1.097393E-04, -3.267006E-04, -9.036567E-04, -2.277257E-03, -5.226756E-03, -1.021707E-02,
        -1.591468E-02, -1.182814E-02, 2.011999E-02, 8.526584E-02, 9.693489E-02, -7.082672E-02, -2.359740E-01, -2.629613E-01, 3.011214E-01, 6.125074E-01, 2.571368E-01
c, 1.22, -9.711872E-08, -7.733970E-07, -3.931052E-06, -1.738323E-05, -5.986536E-05, -2.095853E-04, -5.756482E-04, -1.751595E-03, -3.935120E-03, -1.038490E-02, -1.691100E-02,
        -3.410989E-02, -1.220437E-02, 1.397535E-02, 2.176212E-01, 9.532708E-02, 1.782524E-02, -1.016342E+00, 3.107026E-01, 1.991786E+00, -1.142955E+00, -6.206923E-01
c, 22.22, 1.000000E+00
c, 1.22, -1.533644E-07, -1.234295E-06, -6.177188E-06, -2.787253E-05, -9.355199E-05, -3.378352E-04, -8.935475E-04, -2.842353E-03, -6.052332E-03, -1.704690E-02, -2.549806E-02,
        -5.791149E-02, -1.271791E-02, 6.260762E-03, 4.080306E-01, 1.284258E-01, -1.231286E-01, -2.267331E+00, 2.807507E+00, -3.028009E-01, -2.071227E+00, 1.803625E+00
c, 1.22, -1.764526E-07, -1.314401E-06, -7.355464E-06, -2.863762E-05, -1.156551E-04, -3.330732E-04, -1.155156E-03, -2.659832E-03, -8.332036E-03, -1.462611E-02, -4.030890E-02,
        -3.745842E-02, -7.375959E-02, 1.443268E-01, 2.119031E-01, 7.474187E-01, -2.031080E+00, 1.922693E-01, 2.287768E+00, -5.168135E+00, 6.460323E+00, -2.915551E+00
c, 23.23, 1.0000000
p, TI , 2.553733E+04, 6.042243E+03, 1.962653E+03, 7.517338E+02, 3.198833E+02, 1.464300E+02, 7.074861E+01, 3.559088E+01, 1.836893E+01, 9.658655E+00, 5.136730E+00,
        2.698872E+00, 1.389703E+00, 7.031630E-01, 3.441230E-01, 1.413610E-01, 6.086300E-02, 2.590700E-02, 0.0110300
c, 1.18, 1.200000E-05, 1.040000E-04, 6.070000E-04, 2.751000E-03, 1.026600E-02, 3.218000E-02, 8.448600E-02, 1.783320E-01, 2.885740E-01, 3.287580E-01, 2.115410E-01,
        5.551900E-02, 2.020000E-03, -1.429000E-03, -9.410000E-04, -5.300000E-05, -1.700000E-05, 3.000000E-06
c, 1.18, -4.000000E-06, -3.500000E-05, -2.030000E-04, -9.250000E-04, -3.470000E-03, -1.102000E-02, -2.957700E-02, -6.457400E-02, -1.084580E-01, -1.319100E-01, -5.308900E-02,
        1.781250E-01, 3.957010E-01, 3.927280E-01, 1.744770E-01, 1.894600E-02, -3.280000E-04, 3.760000E-04
c, 1.18, 1.000000E-06, 1.000000E-05, 6.100000E-05, 2.750000E-04, 1.034000E-03, 3.283000E-03, 8.829000E-03, 1.930800E-02, 3.261200E-02, 3.997700E-02, 1.529900E-02,
        -6.262000E-02, -1.382880E-01, -1.763480E-01, -5.540400E-02, 5.592910E-01, 5.331150E-01, 3.817200E-02
c, 1.18, 2.000000E-06, 2.000000E-05, 1.260000E-04, 5.260000E-04, 2.151000E-03, 6.257000E-03, 1.845500E-02, 3.624700E-02, 7.092000E-02, 7.182700E-02, 5.920100E-02,
        -1.842720E-01, -3.296530E-01, -5.457640E-01, 1.145431E+00, 5.526250E-01, -9.642600E-01, -8.824000E-02
c, 1.18, -3.000000E-06, -2.800000E-05, -1.340000E-04, -7.360000E-04, -2.293000E-03, -8.752000E-03, -1.922600E-02, -5.258900E-02, -6.589400E-02, -1.481120E-01, -2.412400E-02,
        1.769790E-01, 1.158428E+00, -9.634580E-01, -1.324508E+00, 2.291373E+00, -1.112932E+00, -2.156310E-01
c, 1.18, 1.000000E-06, 8.000000E-06, 4.900000E-05, 2.210000E-04, 8.360000E-04, 2.641000E-03, 7.151000E-03, 1.553000E-02, 2.647900E-02, 3.198000E-02, 1.256100E-02,
        -5.299400E-02, -1.130390E-01, -1.436980E-01, -1.033600E-02, 3.547360E-01, 5.481010E-01, 2.252960E-01
c, 18.18, 1.000000E+00
c, 19.19, 1.0000000
d, TI , 1.969490E+02, 5.883720E+01, 2.256540E+01, 9.575660E+00, 4.318590E+00, 2.023270E+00, 9.467780E-01, 4.338760E-01, 1.922740E-01, 8.150200E-02, 3.228500E-02, 0.0127900
c, 1.11, 3.820000E-04, 3.368000E-03, 1.624200E-02, 5.237700E-02, 1.299280E-01, 2.312330E-01, 3.035610E-01, 3.092680E-01, 2.316440E-01, 1.015400E-01, 1.168400E-02
c, 1.11, -3.880000E-04, -3.428000E-03, -1.659800E-02, -5.417200E-02, -1.366600E-01, -2.294490E-01, -2.549950E-01, -4.766100E-02, 3.948800E-01, 4.964530E-01, 1.257560E-01
c, 1.11, -6.220000E-04, -5.493000E-03, -2.696800E-02, -8.953300E-02, -2.283740E-01, -3.312580E-01, -1.063490E-01, 6.310360E-01, 3.966210E-01, -6.180090E-01, -2.695920E-01
c, 1.11, -6.960000E-04, -7.089000E-03, -3.101300E-02, -1.200550E-01, -2.789100E-01, -3.789610E-01, 5.737430E-01, 7.279500E-01, -1.297656E+00, 3.203760E-01, 5.230100E-01
c, 11.11, 1.000000E+00
c, 12.12, 1.0000000
f, TI , 1.766281824683E+00, 5.136768574398E-01, 1.626072855227E-01
c, 1.1, 1.000000E+00
c, 2.2, 1.000000E+00
c, 3.3, 1.000000E+00
g, TI , 7.389754318858E-01, 2.308075441408E-01
c, 1.1, 1.000000E+00
c, 2.2, 1.000000E+00
```

```
! vanadium (23s,19p,12d,3f,2g) -> [9s,8p,6d,3f,2g]
s, V , 1.025178E+07, 1.534920E+06, 3.493009E+05, 9.894205E+04, 3.228136E+04, 1.165527E+04, 4.546548E+03, 1.886185E+03, 8.229417E+02, 3.743439E+02, 1.763069E+02,
    8.540661E+01, 4.190435E+01, 1.915525E+01, 9.571617E+00, 4.780086E+00, 2.161860E+00, 1.042711E+00, 4.802820E-01, 1.176380E-01, 5.617400E-02, 2.566100E-02, 0.0117200
c, 1.22, 1.966291E-06, 1.528967E-05, 8.043932E-05, 3.397617E-04, 1.237405E-03, 4.025740E-03, 1.191912E-02, 3.215431E-02, 7.785452E-02, 1.616367E-01, 2.634194E-01,
    2.799986E-01, 1.473474E-01, 6.826405E-02, 1.194436E-01, 8.507582E-02, 1.125814E-02, -3.353521E-04, 1.070571E-04, -2.979622E-05, 4.672816E-05, -3.321273E-08
c, 1.22, -1.100923E-06, -8.563250E-06, -4.503678E-05, -1.903808E-04, -6.934284E-04, -2.262429E-03, -6.724991E-03, -1.837325E-02, -4.555260E-02, -9.996969E-02, -1.819111E-01,
    -2.458744E-01, -1.638089E-01, 1.795843E-01, 5.389547E-01, 3.749098E-01, 5.200269E-02, -3.775873E-03, 1.686888E-04, -3.063802E-04, 3.238096E-04, -3.672244E-05
c, 1.22, 2.278854E-07, 1.773942E-06, 9.319992E-06, 3.946449E-05, 1.435720E-04, 4.702911E-04, 1.399998E-03, 3.874111E-03, 9.765942E-03, 2.244179E-02, 4.390091E-02,
    6.843462E-02, 5.096500E-02, -8.535940E-02, -3.474804E-01, -3.672368E-01, 2.385408E-01, 6.888178E-01, 3.509919E-01, 1.826144E-02, -5.541134E-03, 2.713002E-03
c, 1.22, -5.275948E-08, -4.107129E-07, -2.157711E-06, -9.137220E-06, -3.323885E-05, -1.088925E-04, -3.241407E-04, -8.972319E-04, -2.262105E-03, -5.203068E-03, -1.019123E-02,
    -1.594820E-02, -1.193588E-02, 2.018344E-02, 8.610071E-02, 9.487917E-02, -7.309753E-02, -2.349632E-01, -2.500297E-01, 3.026096E-01, 6.061635E-01, 2.583574E-01
c, 1.22, -1.003479E-07, -7.997061E-07, -4.060397E-06, -1.798070E-05, -6.181370E-05, -2.168915E-04, -5.942417E-04, -1.814506E-03, -4.064807E-03, -1.079223E-02, -1.752264E-02,
    -3.573873E-02, -1.263079E-02, 1.393494E-02, 2.305046E-01, 9.365740E-02, 7.828960E-03, -1.053854E+00, 3.865591E-01, 1.925783E+00, -1.146429E+00, -5.799093E-01
c, 1.22, -1.592148E-07, -1.278305E-06, -6.420097E-06, -2.883633E-05, -9.735890E-05, -3.491402E-04, -9.316194E-04, -2.934959E-03, -6.333703E-03, -1.760698E-02, -2.696848E-02,
    -5.982079E-02, -1.545613E-02, 1.103001E-02, 4.234006E-01, 1.454088E-01, -2.197140E-01, -2.194329E+00, 2.857441E+00, -5.832475E-01, -1.692845E+00, 1.642726E+00
c, 22.22, 1.000000E+00
c, 1.22, -1.885199E-07, -1.394550E-06, -7.881519E-06, -3.027981E-05, -1.243160E-04, -3.507231E-04, -1.246440E-03, -2.787420E-03, -9.045618E-03, -1.522967E-02, -4.437496E-02,
    -3.793507E-02, -8.564216E-02, 1.715635E-01, 2.192374E-01, 8.190393E-01, -2.439698E+00, 9.048685E-01, 1.706923E+00, -4.675814E+00, 6.048734E+00, -2.815697E+00
    c, 23.23, 1.0000000
p, V , 2.856332E+04, 6.757191E+03, 2.194652E+03, 8.405257E+02, 3.576666E+02, 1.637534E+02, 7.914466E+01, 3.983858E+01, 2.058536E+01, 1.084307E+01, 5.777969E+00,
    3.043170E+00, 1.570239E+00, 7.949750E-01, 3.889800E-01, 1.573270E-01, 6.656600E-02, 2.786100E-02, 0.0116600
c, 1.18, 1.100000E-05, 1.010000E-04, 5.920000E-04, 2.690000E-03, 1.006300E-02, 3.165400E-02, 8.347800E-02, 1.772260E-01, 2.883980E-01, 3.294320E-01, 2.121300E-01,
    5.588200E-02, 2.097000E-03, -1.471000E-03, -9.480000E-04, -5.500000E-05, -1.300000E-05, 2.000000E-06
c, 1.18, -4.000000E-06, -3.400000E-05, -2.020000E-04, -9.200000E-04, -3.459000E-03, -1.102500E-02, -2.973200E-02, -6.534900E-02, -1.105270E-01, -1.345370E-01, -5.243700E-02,
    1.822160E-01, 3.980180E-01, 3.891530E-01, 1.727170E-01, 1.874600E-02, -5.940000E-04, 4.220000E-04
c, 1.18, 1.000000E-06, 1.000000E-05, 6.000000E-05, 2.750000E-04, 1.033000E-03, 3.296000E-03, 8.896000E-03, 1.961300E-02, 3.331800E-02, 4.100900E-02, 1.495900E-02,
    -6.425600E-02, -1.418170E-01, -1.739660E-01, -4.113200E-02, 5.639950E-01, 5.238960E-01, 3.568900E-02
c, 1.18, 2.000000E-06, 2.000000E-05, 1.290000E-04, 5.360000E-04, 2.203000E-03, 6.428000E-03, 1.907100E-02, 3.771200E-02, 7.426700E-02, 7.526000E-02, 6.099800E-02,
    -2.009760E-01, -3.453430E-01, -5.018350E-01, 1.143631E+00, 5.044870E-01, -9.441030E-01, -7.577800E-02
c, 1.18, -3.000000E-06, -2.900000E-05, -1.410000E-04, -7.730000E-04, -2.422000E-03, -9.267000E-03, -2.055500E-02, -5.648500E-02, -7.149300E-02, -1.577910E-01, -2.155200E-02,
    2.379500E-01, 1.131837E+00, -1.089180E+00, -1.125773E+00, 2.168189E+00, -1.110263E+00, -1.840100E-01
c, 1.18, 1.000000E-06, 8.000000E-06, 4.900000E-05, 2.210000E-04, 8.380000E-04, 2.656000E-03, 7.233000E-03, 1.580800E-02, 2.717200E-02, 3.280100E-02, 1.233800E-02,
    -5.541800E-02, -1.156040E-01, -1.390920E-01, 6.820000E-04, 3.520090E-01, 5.422050E-01, 2.313580E-01
c, 18.18, 1.000000E+00
c, 19.19, 1.0000000
d, V , 2.380090E+02, 7.118960E+01, 2.739460E+01, 1.171730E+01, 5.324800E+00, 2.516980E+00, 1.190750E+00, 5.518090E-01, 2.469260E-01, 1.051000E-01, 4.139300E-02, 0.0163000
c, 1.11, 3.500000E-04, 3.130000E-03, 1.547800E-02, 5.082900E-02, 1.275480E-01, 2.304470E-01, 3.040580E-01, 3.083660E-01, 2.290220E-01, 1.013600E-01, 1.296100E-02
c, 1.11, -3.700000E-04, -3.317000E-03, -1.647400E-02, -5.483900E-02, -1.408390E-01, -2.395530E-01, -2.519100E-01, -2.610600E-02, 3.877700E-01, 4.867240E-01, 1.342240E-01
c, 1.11, -5.750000E-04, -5.157000E-03, -2.592900E-02, -8.805900E-02, -2.311790E-01, -3.383520E-01, -8.503000E-02, 6.267990E-01, 3.834730E-01, -6.102620E-01, -2.841040E-01
c, 1.11, 7.340000E-04, 7.258000E-03, 3.395800E-02, 1.298550E-01, 3.316480E-01, 3.569830E-01, -6.934390E-01, -5.768500E-01, 1.228029E+00, -3.167410E-01, -4.942840E-01
c, 11.11, 1.000000E+00
c, 12.12, 1.0000000
f, V , 1.930056399457E+00, 5.964719154878E-01, 1.937358533397E-01
c, 1.1, 1.000000E+00
c, 2.2, 1.000000E+00
c, 3.3, 1.000000E+00
g, V , 9.165158923226E-01, 2.844002671432E-01
c, 1.1, 1.000000E+00
c, 2.2, 1.000000E+00
```

```
! chromium            (23s,19p,12d,3f,2g) -> [9s,8p,6d,3f,2g]
s, CR , 1.101664E+07, 1.649423E+06, 3.753589E+05, 1.063236E+05, 3.468976E+04, 1.252483E+04, 4.885752E+03, 2.026918E+03, 8.843645E+02, 4.023170E+02, 1.895271E+02,
        9.186095E+01, 4.512476E+01, 2.062512E+01, 1.031973E+01, 5.157723E+00, 2.312628E+00, 1.103079E+00, 4.980420E-01, 1.182470E-01, 5.735600E-02, 2.576000E-02, 0.0115700
c, 1.22, 2.002522E-06, 1.557145E-05, 8.192146E-05, 3.460132E-04, 1.260141E-03, 4.099273E-03, 1.213424E-02, 3.271636E-02, 7.912296E-02, 1.638397E-01, 2.655107E-01,
        2.789671E-01, 1.444466E-01, 6.941606E-02, 1.195153E-01, 8.084582E-02, 9.975024E-03, -2.865770E-04, 1.176051E-04, -2.164013E-05, 5.032702E-05, 2.325982E-06
c, 1.22, -1.118844E-06, -8.702790E-06, -4.576974E-05, -1.934803E-04, -7.046922E-04, -2.299139E-03, -6.833193E-03, -1.866493E-02, -4.624835E-02, -1.013820E-01, -1.839390E-01,
        -2.469356E-01, -1.597390E-01, 1.940936E-01, 5.461479E-01, 3.610571E-01, 4.658647E-02, -3.493969E-03, 2.600074E-04, -2.440100E-04, 3.266435E-04, -2.070386E-05
c, 1.22, 2.358227E-07, 1.836140E-06, 9.643686E-06, 4.085166E-05, 1.485404E-04, 4.868639E-04, 1.448156E-03, 4.010503E-03, 1.009579E-02, 2.321493E-02, 4.524056E-02,
        7.025001E-02, 5.032135E-02, -9.350992E-02, -3.650249E-01, -3.530889E-01, 2.829720E-01, 7.001955E-01, 3.067473E-01, 1.083139E-02, -1.458427E-03, 1.552323E-03
c, 1.22, -5.343793E-08, -4.166295E-07, -2.183977E-06, -9.275174E-06, -3.361788E-05, -1.106225E-04, -3.275214E-04, -9.122671E-04, -2.281936E-03, -5.294506E-03, -1.022548E-02,
        -1.618464E-02, -1.124211E-02, 2.096236E-02, 9.050667E-02, 8.642079E-02, -7.853630E-02, -2.526007E-01, -2.133579E-01, 3.577908E-01, 5.691145E-01, 2.371138E-01
c, 1.22, -1.038573E-07, -8.291687E-07, -4.198902E-06, -1.865777E-05, -6.386131E-05, -2.252576E-04, -6.131653E-04, -1.886356E-03, -4.185316E-03, -1.123172E-02, -1.791544E-02,
        -3.714198E-02, -1.126485E-02, 1.522975E-02, 2.458640E-01, 7.882802E-02, -1.862956E-02, -1.096262E+00, 5.510966E-01, 1.894727E+00, -1.315773E+00, -4.715708E-01
c, 1.22, -1.671645E-07, -1.331951E-06, -6.764614E-06, -2.994507E-05, -1.029930E-04, -3.611865E-04, -9.903414E-04, -3.022002E-03, -6.778242E-03, -1.798719E-02, -2.926094E-02,
        -5.950832E-02, -2.030054E-02, 2.995049E-02, 4.202560E-01, 1.844826E-01, -4.526654E-01, -1.892157E+00, 2.848594E+00, -1.124775E+00, -9.810669E-01, 1.359031E+00
c, 22.22, 1.000000E+00
c, 1.22, -2.018526E-07, -1.476099E-06, -8.479016E-06, -3.186673E-05, -1.344019E-04, -3.665342E-04, -1.355222E-03, -2.884635E-03, -9.908511E-03, -1.547488E-02, -4.925232E-02,
        -3.533906E-02, -9.963499E-02, 2.140183E-01, 2.137561E-01, 8.609798E-01, -2.925843E+00, 1.966558E+00, 7.515651E-01, -4.107397E+00, 5.701781E+00, -2.735618E+00
c, 23.23, 1.0000000
p, CR , 3.153989E+04, 7.460234E+03, 2.422622E+03, 9.277547E+02, 3.948041E+02, 1.808027E+02, 8.742393E+01, 4.403427E+01, 2.277704E+01, 1.201547E+01, 6.411751E+00,
        3.381136E+00, 1.747054E+00, 8.850040E-01, 4.330280E-01, 1.734150E-01, 7.260800E-02, 2.991500E-02, 0.0123300
c, 1.18, 1.100000E-05, 1.000000E-04, 5.870000E-04, 2.667000E-03, 9.991000E-03, 3.149300E-02, 8.326600E-02, 1.773280E-01, 2.892300E-01, 3.296730E-01, 2.108870E-01,
        5.517400E-02, 2.230000E-03, -1.284000E-03, -8.540000E-04, -4.600000E-05, -1.200000E-05, 2.000000E-06
c, 1.18, -4.000000E-06, -3.500000E-05, -2.030000E-04, -9.260000E-04, -3.487000E-03, -1.114100E-02, -3.012800E-02, -6.648400E-02, -1.128540E-01, -1.367850E-01, -5.005700E-02,
        1.884690E-01, 4.004550E-01, 3.843320E-01, 1.695200E-01, 1.825700E-02, -7.430000E-04, 4.390000E-04
c, 1.18, 1.000000E-06, 1.000000E-05, 6.100000E-05, 2.770000E-04, 1.041000E-03, 3.338000E-03, 9.013000E-03, 2.001300E-02, 3.400000E-02, 4.199300E-02, 1.380400E-02,
        -6.627500E-02, -1.461070E-01, -1.694750E-01, -2.858700E-02, 5.663070E-01, 5.156880E-01, 3.536200E-02
c, 1.18, 3.000000E-06, 2.100000E-05, 1.340000E-04, 5.590000E-04, 2.297000E-03, 6.727000E-03, 1.999000E-02, 3.978900E-02, 7.842600E-02, 7.988200E-02, 6.097200E-02,
        -2.226880E-01, -3.668130E-01, -4.471090E-01, 1.151801E+00, 4.413050E-01, -9.140660E-01, -6.694600E-02
c, 1.18, -3.000000E-06, -3.100000E-05, -1.500000E-04, -8.150000E-04, -2.583000E-03, -9.813000E-03, -2.211600E-02, -6.029600E-02, -7.818700E-02, -1.674430E-01, -1.352300E-02,
        3.066960E-01, 1.090783E+00, -1.234545E+00, -9.015550E-01, 2.048266E+00, -1.103984E+00, -1.641220E-01
c, 1.18, 1.000000E-06, 8.000000E-06, 4.800000E-05, 2.180000E-04, 8.270000E-04, 2.626000E-03, 7.172000E-03, 1.573800E-02, 2.715400E-02, 3.263100E-02, 1.149300E-02,
        -5.607700E-02, -1.142520E-01, -1.337370E-01, 3.473000E-03, 3.451660E-01, 5.418440E-01, 2.389580E-01
c, 18.18, 1.000000E+00
c, 19.19, 1.0000000
d, CR , 2.680400E+02, 8.022230E+01, 3.089500E+01, 1.324810E+01, 6.040770E+00, 2.861590E+00, 1.355450E+00, 6.282480E-01, 2.806230E-01, 1.186680E-01, 4.593100E-02, 0.0177800
c, 1.11, 3.600000E-04, 3.236000E-03, 1.615300E-02, 5.341800E-02, 1.335240E-01, 2.386370E-01, 3.094550E-01, 3.056910E-01, 2.182890E-01, 8.969300E-02, 9.672000E-03
c, 1.11, -4.070000E-04, -3.677000E-03, -1.842200E-02, -6.205000E-02, -1.589460E-01, -2.629000E-01, -2.438830E-01, 3.774000E-02, 4.270640E-01, 4.428120E-01, 9.654900E-02
c, 1.11, 8.280000E-04, 8.072000E-03, 3.896000E-02, 1.497060E-01, 3.879330E-01, 2.970340E-01, -8.758060E-01, -2.958520E-01, 1.204424E+00, -5.298930E-01, -3.455720E-01
c, 1.11, -5.820000E-04, -5.276000E-03, -2.672300E-02, -9.201200E-02, -2.423410E-01, -3.383560E-01, -1.388600E-02, 6.731700E-01, 2.610890E-01, -6.613960E-01, -2.245580E-01
c, 11.11, 1.000000E+00
c, 12.12, 1.0000000
f, CR , 2.344012913810E+00, 7.429335995812E-01, 2.443093003606E-01
c, 1.1, 1.000000E+00
c, 2.2, 1.000000E+00
c, 3.3, 1.000000E+00
g, CR , 1.177822916538E+00, 3.803907958863E-01
c, 1.1, 1.000000E+00
c, 2.2, 1.000000E+00
```

```
! manganese            (23s,19p,12d,3f,2g) -> [9s,8p,6d,3f,2g]
s, MN , 1.221050E+07, 1.828157E+06, 4.160303E+05, 1.178436E+05, 3.844814E+04, 1.388171E+04, 5.415002E+03, 2.246460E+03, 9.801463E+02, 4.458952E+02, 2.100558E+02,
        1.018046E+02, 5.001126E+01, 2.293939E+01, 1.152264E+01, 5.776338E+00, 2.625959E+00, 1.260322E+00, 5.771300E-01, 1.365820E-01, 6.353500E-02, 2.852100E-02, 0.0128000
c, 1.22, 2.018537E-06, 1.569638E-05, 8.257853E-05, 3.488107E-04, 1.270414E-03, 4.133905E-03, 1.224366E-02, 3.306040E-02, 8.019877E-02, 1.671550E-01, 2.743977E-01,
        2.963690E-01, 1.592852E-01, 5.681818E-02, 8.278990E-02, 5.840628E-02, 7.343047E-03, -2.934793E-04, -4.562245E-05, -9.934988E-06, 2.391143E-05, 3.058560E-06
c, 1.22, -9.633574E-07, -7.493672E-06, -3.941004E-05, -1.666155E-04, -6.069041E-04, -1.981108E-03, -5.893486E-03, -1.613849E-02, -4.018929E-02, -8.903423E-02, -1.646993E-01,
        -2.288840E-01, -1.560684E-01, 1.882585E-01, 5.512725E-01, 3.755111E-01, 5.061607E-02, -4.926193E-03, -6.126838E-04, -2.821975E-04, 3.009192E-04, -2.702120E-05
c, 1.22, 2.316977E-07, 1.803633E-06, 9.476176E-06, 4.012553E-05, 1.459870E-04, 4.782368E-04, 1.424063E-03, 3.942807E-03, 9.952411E-03, 2.292420E-02, 4.501638E-02,
        7.048742E-02, 5.291064E-02, -8.983951E-02, -3.626155E-01, -3.621266E-01, 2.656979E-01, 6.892091E-01, 3.337072E-01, 1.594677E-02, -4.614546E-03, 2.502379E-03
c, 1.22, -5.127786E-08, -3.991995E-07, -2.097137E-06, -8.881375E-06, -3.230705E-05, -1.058611E-04, -3.151614E-04, -8.730066E-04, -2.203528E-03, -5.081761E-03, -9.988834E-03,
        -1.570961E-02, -1.183955E-02, 2.031039E-02, 8.593055E-02, 8.897427E-02, -7.678891E-02, -2.261750E-01, -2.271869E-01, 3.003127E-01, 5.953419E-01, 2.632967E-01
c, 1.22, -1.017421E-07, -8.110718E-07, -4.116344E-06, -1.823899E-05, -6.265924E-05, -2.200741E-04, -6.024678E-04, -1.843000E-03, -4.127938E-03, -1.100471E-02, -1.790120E-02,
        -3.676227E-02, -1.301427E-02, 1.478153E-02, 2.391713E-01, 8.964957E-02, -1.299627E-02, -1.051330E+00, 4.478536E-01, 1.804806E+00, -1.071026E+00, -5.693171E-01
c, 1.22, -1.642320E-07, -1.314241E-06, -6.632840E-06, -2.960420E-05, -1.007673E-04, -3.579039E-04, -9.666897E-04, -3.005179E-03, -6.605816E-03, -1.803820E-02, -2.853084E-02,
        -6.124413E-02, -1.893791E-02, 1.888653E-02, 4.329357E-01, 1.661622E-01, -3.401427E-01, -2.039320E+00, 2.794661E+00, -7.303582E-01, -1.408003E+00, 1.496335E+00
c, 22.22, 1.000000E+00
c, 1.22, -2.001522E-07, -1.478765E-06, -8.372432E-06, -3.208892E-05, -1.321413E-04, -3.714493E-04, -1.326456E-03, -2.951265E-03, -9.655762E-03, -1.616919E-02, -4.776647E-02,
        -4.049212E-02, -9.389925E-02, 1.920182E-01, 2.440487E-01, 8.589060E-01, -2.793145E+00, 1.564069E+00, 1.118462E+00, -4.054664E+00, 5.462054E+00, -2.638636E+00
        c, 23.23, 1.0000000
p, MN , 3.500512E+04, 8.279462E+03, 2.688465E+03, 1.029490E+03, 4.380708E+02, 2.006153E+02, 9.700769E+01, 4.887052E+01, 2.529281E+01, 1.335382E+01, 7.131786E+00,
        3.764365E+00, 1.946265E+00, 9.854550E-01, 4.818650E-01, 1.892120E-01, 7.798000E-02, 3.168500E-02, 0.0128700
c, 1.17, 1.100000E-05, 9.800000E-05, 5.720000E-04, 2.604000E-03, 9.779000E-03, 3.093800E-02, 8.219400E-02, 1.761490E-01, 2.891330E-01, 3.309560E-01, 2.122180E-01,
        5.522700E-02, 1.207000E-03, -2.355000E-03, -1.320000E-03, -9.400000E-05, -7.000000E-06
c, 1.18, -4.000000E-06, -3.400000E-05, -1.990000E-04, -9.070000E-04, -3.425000E-03, -1.098500E-02, -2.985600E-02, -6.636600E-02, -1.135040E-01, -1.379910E-01, -4.935500E-02,
        1.912420E-01, 4.023550E-01, 3.821160E-01, 1.681990E-01, 1.790500E-02, -9.580000E-04, 4.860000E-04
c, 1.18, 1.000000E-06, 1.000000E-05, 6.100000E-05, 2.780000E-04, 1.052000E-03, 3.366000E-03, 9.193000E-03, 2.040400E-02, 3.527700E-02, 4.298600E-02, 1.478300E-02,
        -7.085100E-02, -1.488630E-01, -1.726610E-01, -4.857000E-03, 5.718810E-01, 5.013280E-01, 3.227100E-02
c, 1.18, 3.000000E-06, 2.100000E-05, 1.380000E-04, 5.680000E-04, 2.368000E-03, 6.887000E-03, 2.080500E-02, 4.120700E-02, 8.294900E-02, 8.233000E-02, 6.764900E-02,
        -2.505870E-01, -3.787840E-01, -3.965170E-01, 1.150112E+00, 3.830850E-01, -8.923280E-01, -5.308700E-02
c, 1.18, -3.000000E-06, -3.200000E-05, -1.650000E-04, -8.520000E-04, -2.864000E-03, -1.033800E-02, -2.492000E-02, -6.390800E-02, -9.170400E-02, -1.688330E-01, -4.571700E-02,
        4.475430E-01, 1.044544E+00, -1.485721E+00, -5.306040E-01, 1.821718E+00, -1.068450E+00, -1.281010E-01
c, 1.18, -1.000000E-06, -8.000000E-06, -4.600000E-05, -2.080000E-04, -7.920000E-04, -2.522000E-03, -6.928000E-03, -1.529200E-02, -2.661100E-02, -3.197800E-02, -1.122200E-02,
        5.498800E-02, 1.104650E-01, 1.281620E-01, -2.980000E-03, -3.368240E-01, -5.424410E-01, -2.486330E-01
c, 18.18, 1.000000E+00
c, 19.19, 1.0000000
d, MN , 3.014270E+02, 9.032880E+01, 3.483370E+01, 1.497700E+01, 6.849830E+00, 3.252170E+00, 1.543090E+00, 7.156410E-01, 3.193230E-01, 1.344280E-01, 5.157000E-02, 0.0197800
c, 1.11, 3.610000E-04, 3.259000E-03, 1.641900E-02, 5.476000E-02, 1.367270E-01, 2.424190E-01, 3.103760E-01, 3.021700E-01, 2.135730E-01, 8.909400E-02, 1.085700E-02
c, 1.11, -3.670000E-04, -3.331000E-03, -1.681300E-02, -5.692900E-02, -1.457700E-01, -2.466730E-01, -2.342270E-01, 2.332000E-02, 3.875770E-01, 4.676950E-01, 1.492920E-01
c, 1.11, -5.810000E-04, -5.304000E-03, -2.701900E-02, -9.406700E-02, -2.469380E-01, -3.420510E-01, -1.462100E-02, 6.120700E-01, 3.201120E-01, -5.609790E-01, -3.146700E-01
c, 1.11, -7.460000E-04, -6.858000E-03, -3.503600E-02, -1.265920E-01, -3.598640E-01, -3.065310E-01, 7.526320E-01, 4.176590E-01, -1.072832E+00, 2.000430E-01, 5.541570E-01
c, 11.11, 1.000000E+00
c, 12.12, 1.0000000
f, MN , 3.464575261360E+00, 1.203677105162E+00, 4.596740635520E-01
c, 1.1, 1.000000E+00
c, 2.2, 1.000000E+00
c, 3.3, 1.000000E+00
g, MN , 2.816890726131E+00, 6.830618027650E-01
c, 1.1, 1.000000E+00
c, 2.2, 1.000000E+00
```

! iron (23s,19p,12d,3f,2g) -> [9s,8p,6d,3f,2g]
s, FE , 1.327714E+07, 1.987888E+06, 4.523871E+05, 1.281437E+05, 4.180917E+04, 1.509533E+04, 5.888438E+03, 2.442876E+03, 1.065857E+03, 4.849096E+02, 2.284585E+02,
1.107453E+02, 5.443116E+01, 2.501096E+01, 1.259262E+01, 6.323202E+00, 2.883384E+00, 1.382868E+00, 6.322010E-01, 1.492380E-01, 6.849400E-02, 3.038100E-02, 0.0134800
c, 1.22, 1.976511E-06, 1.536912E-05, 8.085571E-05, 3.415253E-04, 1.243883E-03, 4.047547E-03, 1.198848E-02, 3.237358E-02, 7.854828E-02, 1.637396E-01, 2.687335E-01,
2.896317E-01, 1.554235E-01, 6.284331E-02, 9.927972E-02, 6.931444E-02, 8.885666E-03, -2.776200E-04, 1.271590E-06, -1.537759E-05, 2.552339E-05, 1.483208E-06
c, 1.22, -1.020185E-06, -7.935347E-06, -4.173309E-05, -1.764249E-04, -6.426379E-04, -2.097440E-03, -6.238750E-03, -1.707492E-02, -4.248570E-02, -9.392294E-02, -1.730554E-01,
-2.386626E-01, -1.623971E-01, 1.869345E-01, 5.495686E-01, 3.723118E-01, 5.057065E-02, -4.072645E-03, -2.732199E-04, -2.612296E-04, 2.547393E-04, -2.990448E-05
c, 1.22, 2.340322E-07, 1.821727E-06, 9.571315E-06, 4.052652E-05, 1.474531E-04, 4.830333E-04, 1.438609E-03, 3.983915E-03, 1.006355E-02, 2.320610E-02, 4.566780E-02,
7.169322E-02, 5.421018E-02, -9.163776E-02, -3.693219E-01, -3.616033E-01, 2.734191E-01, 6.889090E-01, 3.294179E-01, 1.558687E-02, -4.161049E-03, 2.512001E-03
c, 1.22, -5.062297E-08, -3.940586E-07, -2.070340E-06, -8.766406E-06, -3.189537E-05, -1.044911E-04, -3.112113E-04, -8.620181E-04, -2.178129E-03, -5.026970E-03, -9.908113E-03,
-1.561357E-02, -1.187731E-02, 2.031246E-02, 8.560291E-02, 8.702717E-02, -7.758197E-02, -2.214919E-01, -2.196263E-01, 2.869893E-01, 5.927434E-01, 2.762253E-01
c, 1.22, -9.957952E-08, -7.925506E-07, -4.031635E-06, -1.780938E-05, -6.142010E-05, -2.147300E-04, -5.912544E-04, -1.797193E-03, -4.061089E-03, -1.073419E-02, -1.773464E-02,
-3.586338E-02, -1.374002E-02, 1.645324E-02, 2.322749E-01, 9.256802E-02, -3.322957E-02, -9.860290E-01, 4.046445E-01, 1.688889E+00, -8.479093E-01, -6.816407E-01
c, 1.22, -1.678013E-07, -1.337004E-06, -6.790249E-06, -3.005871E-05, -1.033899E-04, -3.626365E-04, -9.947359E-04, -3.038134E-03, -6.831095E-03, -1.819172E-02, -2.987575E-02,
-6.129076E-02, -2.302594E-02, 2.701671E-02, 4.229967E-01, 2.153088E-01, -4.343164E-01, -1.950348E+00, 2.720233E+00, -6.123241E-01, -1.468329E+00, 1.469516E+00
c, 22.22, 1.000000E+00
c, 1.22, -1.979190E-07, -1.454458E-06, -8.296892E-06, -3.147678E-05, -1.312505E-04, -3.631851E-04, -1.321103E-03, -2.874345E-03, -9.655667E-03, -1.565078E-02, -4.818210E-02,
-3.801945E-02, -9.789664E-02, 1.990898E-01, 2.020013E-01, 9.776064E-01, -3.003579E+00, 1.801662E+00, 9.747971E-01, -3.897557E+00, 5.227909E+00, -2.518672E+00
c, 23.23, 1.0000000
p, FE , 3.828205E+04, 9.061556E+03, 2.944450E+03, 1.128158E+03, 4.803380E+02, 2.201368E+02, 1.065482E+02, 5.374262E+01, 2.786318E+01, 1.474382E+01, 7.893351E+00,
4.179497E+00, 2.165669E+00, 1.097240E+00, 5.360850E-01, 2.023740E-01, 8.174200E-02, 3.266200E-02, 0.0130500
c, 1.18, 1.100000E-05, 9.700000E-05, 5.660000E-04, 2.575000E-03, 9.676000E-03, 3.064400E-02, 8.155300E-02, 1.752450E-01, 2.884650E-01, 3.307340E-01, 2.127700E-01,
5.620300E-02, 1.807000E-03, -1.999000E-03, -1.155000E-03, -7.900000E-05, -3.000000E-06, -1.000000E-06
c, 1.18, -4.000000E-06, -3.400000E-05, -1.990000E-04, -9.110000E-04, -3.437000E-03, -1.104200E-02, -3.006100E-02, -6.704900E-02, -1.150700E-01, -1.399170E-01, -4.948000E-02,
1.917090E-01, 4.030120E-01, 3.807170E-01, 1.693050E-01, 1.787200E-02, -1.433000E-03, 5.990000E-04
c, 1.18, 1.000000E-06, 1.100000E-05, 6.600000E-05, 2.950000E-04, 1.134000E-03, 3.585000E-03, 9.950000E-03, 2.181000E-02, 3.866100E-02, 4.562800E-02, 1.795800E-02,
-8.159300E-02, -1.581940E-01, -1.890710E-01, 4.462100E-02, 6.116920E-01, 4.461700E-01, 2.030900E-02
c, 1.18, 3.000000E-06, 2.200000E-05, 1.470000E-04, 5.980000E-04, 2.527000E-03, 7.260000E-03, 2.230000E-02, 4.359200E-02, 8.982400E-02, 8.632800E-02, 7.970000E-02,
-2.943090E-01, -4.046430E-01, -3.242020E-01, 1.189837E+00, 2.280980E-01, -8.474340E-01, -2.274000E-02
c, 1.18, -3.000000E-06, -3.300000E-05, -1.780000E-04, -8.800000E-04, -3.075000E-03, -1.071400E-02, -2.695300E-02, -6.627900E-02, -1.011440E-01, -1.704670E-01, -8.377500E-02,
6.056490E-01, 9.572390E-01, -1.734836E+00, -9.786500E-02, 1.574813E+00, -1.081817E+00, -6.820800E-02
c, 1.18, 1.000000E-06, 7.000000E-06, 4.400000E-05, 1.970000E-04, 7.510000E-04, 2.391000E-03, 6.595000E-03, 1.457000E-02, 2.552900E-02, 3.048500E-02, 1.085300E-02,
-5.238500E-02, -1.032710E-01, -1.212410E-01, 8.400000E-04, 3.272880E-01, 5.442260E-01, 2.589440E-01
c, 18.18, 1.000000E+00
c, 19.19, 1.0000000
d, FE , 3.385060E+02, 1.014920E+02, 3.918670E+01, 1.690340E+01, 7.762960E+00, 3.698870E+00, 1.759630E+00, 8.166510E-01, 3.638210E-01, 1.525410E-01, 5.799100E-02, 0.0220500
c, 1.11, 3.560000E-04, 3.229000E-03, 1.641400E-02, 5.513300E-02, 1.375020E-01, 2.425950E-01, 3.085230E-01, 2.998140E-01, 2.150480E-01, 9.257700E-02, 1.190800E-02
c, 1.11, -3.910000E-04, -3.576000E-03, -1.819100E-02, -6.209000E-02, -1.592870E-01, -2.697760E-01, -2.389970E-01, 6.198500E-02, 4.136570E-01, 4.348780E-01, 1.142190E-01
c, 1.11, -5.650000E-04, -5.185000E-03, -2.661100E-02, -9.307900E-02, -2.482970E-01, -3.498230E-01, 3.692200E-02, 6.494720E-01, 2.342380E-01, -6.097450E-01, -2.622020E-01
c, 1.11, -8.160000E-04, -7.124000E-03, -3.856000E-02, -1.335670E-01, -4.152110E-01, -2.793690E-01, 9.276650E-01, 1.855890E-01, -1.079616E+00, 4.197090E-01, 4.038090E-01
c, 11.11, 1.000000E+00
c, 12.12, 1.0000000
f, FE , 5.052426449402E+00, 1.231289001076E+00, 3.511720962166E-01
c, 1.1, 1.000000E+00
c, 2.2, 1.000000E+00
c, 3.3, 1.000000E+00
g, FE , 3.787913037852E+00, 5.812995677254E-01
c, 1.1, 1.000000E+00
c, 2.2, 1.000000E+00

! cobalt (23s,19p,12d,3f,2g) -> [9s,8p,6d,3f,2g]
s, CO , 1.437022E+07, 2.151540E+06, 4.896257E+05, 1.386903E+05, 4.524989E+04, 1.633762E+04, 6.373073E+03, 2.643958E+03, 1.153614E+03, 5.248606E+02, 2.473069E+02,
1.199063E+02, 5.896429E+01, 2.713616E+01, 1.368912E+01, 6.882771E+00, 3.144647E+00, 1.506266E+00, 6.872680E-01, 1.609720E-01, 7.301700E-02, 3.207100E-02, 0.0140900
c, 1.22, 1.961579E-06, 1.525308E-05, 8.024671E-05, 3.389582E-04, 1.234551E-03, 4.017237E-03, 1.189936E-02, 3.213721E-02, 7.799970E-02, 1.626825E-01, 2.671849E-01,
2.881411E-01, 1.548301E-01, 6.451896E-02, 1.030728E-01, 7.137991E-02, 9.131332E-03, -2.919679E-04, -5.581488E-06, -1.732351E-05, 2.132347E-05, -3.016488E-07
c, 1.22, -1.032046E-06, -8.027614E-06, -4.221930E-05, -1.784817E-04, -6.501411E-04, -2.121909E-03, -6.311755E-03, -1.727558E-02, -4.299353E-02, -9.507125E-02, -1.752212E-01,
-2.416127E-01, -1.645933E-01, 1.880060E-01, 5.505157E-01, 3.701682E-01, 5.007243E-02, -3.975686E-03, -2.896409E-04, -2.552515E-04, 2.182807E-04, -3.660248E-05
c, 1.22, 2.357772E-07, 1.835300E-06, 9.642951E-06, 4.082978E-05, 1.485630E-04, 4.866688E-04, 1.449618E-03, 4.015116E-03, 1.014820E-02, 2.342321E-02, 4.617220E-02,
7.262205E-02, 5.512470E-02, -9.356803E-02, -3.752066E-01, -3.595010E-01, 2.821187E-01, 6.882851E-01, 3.243680E-01, 1.501498E-02, -3.906005E-03, 2.450562E-03
c, 1.22, -4.983880E-08, -3.879304E-07, -2.038379E-06, -8.630129E-06, -3.140519E-05, -1.028682E-04, -3.064776E-04, -8.488581E-04, -2.146732E-03, -4.957499E-03, -9.792183E-03,
-1.545065E-02, -1.181794E-02, 2.031574E-02, 8.494754E-02, 8.455870E-02, -7.824438E-02, -2.161403E-01, -2.120657E-01, 2.777048E-01, 5.886342E-01, 2.865914E-01
c, 1.22, -1.733620E-07, -1.376434E-06, -7.026909E-06, -3.089757E-05, -1.071929E-04, -3.721161E-04, -1.033758E-03, -3.111602E-03, -7.127428E-03, -1.859372E-02, -3.148302E-02,
-6.222078E-02, -2.684359E-02, 3.499360E-02, 4.220093E-01, 2.573147E-01, -5.363089E-01, -1.856112E+00, 2.651475E+00, -5.228775E-01, -1.483086E+00, 1.420599E+00
c, 1.22, -9.633772E-08, -7.654083E-07, -3.903640E-06, -1.718665E-05, -5.952640E-05, -2.070482E-04, -5.737293E-04, -1.731567E-03, -3.950029E-03, -1.033969E-02, -1.736027E-02,
-3.449074E-02, -1.422040E-02, 1.816713E-02, 2.219044E-01, 9.496945E-02, -5.356770E-02, -9.071045E-01, 3.491874E-01, 1.586689E+00, -6.425986E-01, -7.884823E-01
c, 22.22, 1.000000E+00
c, 1.22, -1.968109E-07, -1.439789E-06, -8.265947E-06, -3.108989E-05, -1.310184E-04, -3.577366E-04, -1.321726E-03, -2.821630E-03, -9.692646E-03, -1.528143E-02, -4.871094E-02,
-3.611465E-02, -1.014559E-01, 2.059185E-01, 1.740209E-01, 1.061896E+00, -3.187512E+00, 2.052730E+00, 7.962451E-01, -3.730949E+00, 5.018110E+00, -2.419108E+00
c, 23.23, 1.0000000
p, CO , 4.196140E+04, 9.928306E+03, 3.224971E+03, 1.235293E+03, 5.258469E+02, 2.409762E+02, 1.166418E+02, 5.884834E+01, 3.052910E+01, 1.616943E+01, 8.664236E+00,
4.591943E+00, 2.381048E+00, 1.206246E+00, 5.888220E-01, 2.246580E-01, 9.043600E-02, 3.584800E-02, 0.0142100
c, 1.17, 1.100000E-05, 9.500000E-05, 5.570000E-04, 2.538000E-03, 9.554000E-03, 3.033500E-02, 8.098400E-02, 1.746920E-01, 2.885440E-01, 3.311360E-01, 2.128970E-01,
5.629500E-02, 1.895000E-03, -1.955000E-03, -1.126000E-03, -7.400000E-05, -3.000000E-06
c, 1.18, -4.000000E-06, -3.400000E-05, -1.980000E-04, -9.060000E-04, -3.428000E-03, -1.103900E-02, -3.015700E-02, -6.754900E-02, -1.164660E-01, -1.414410E-01, -4.871700E-02,
1.946550E-01, 4.041480E-01, 3.789040E-01, 1.673060E-01, 1.771900E-02, -1.252000E-03, 5.510000E-04
c, 1.18, 1.000000E-06, 1.100000E-05, 6.900000E-05, 3.080000E-04, 1.187000E-03, 3.760000E-03, 1.047800E-02, 2.305800E-02, 4.109500E-02, 4.852900E-02, 1.882100E-02,
-8.898300E-02, -1.710610E-01, -1.897640E-01, 7.187400E-02, 6.049170E-01, 4.357830E-01, 2.341000E-02
c, 1.18, 3.000000E-06, 2.300000E-05, 1.500000E-04, 6.200000E-04, 2.592000E-03, 7.571000E-03, 2.300100E-02, 4.594300E-02, 9.293700E-02, 9.340700E-02, 8.015200E-02,
-3.177930E-01, -4.378130E-01, -2.329460E-01, 1.164897E+00, 1.731560E-01, -8.160710E-01, -3.118700E-02
c, 1.18, -4.000000E-06, -3.300000E-05, -1.850000E-04, -8.870000E-04, -3.204000E-03, -1.084900E-02, -2.833100E-02, -6.736900E-02, -1.083230E-01, -1.719860E-01, -1.084480E-01,
7.416070E-01, 8.082640E-01, -1.780272E+00, 6.365300E-02, 1.474242E+00, -1.038549E+00, -8.033000E-02
c, 1.18, 1.000000E-06, 7.000000E-06, 4.400000E-05, 1.990000E-04, 7.600000E-04, 2.425000E-03, 6.706000E-03, 1.489000E-02, 2.618100E-02, 3.131000E-02, 1.084800E-02,
-5.386200E-02, -1.061120E-01, -1.217750E-01, 3.863000E-03, 3.271980E-01, 5.419610E-01, 2.617780E-01
c, 18.18, 1.000000E+00
c, 19.19, 1.0000000
d, CO , 3.753200E+02, 1.126040E+02, 4.351900E+01, 1.881470E+01, 8.664910E+00, 4.137110E+00, 1.970800E+00, 9.150070E-01, 4.073700E-01, 1.704440E-01, 6.437900E-02, 0.0243200
c, 1.11, 3.560000E-04, 3.239000E-03, 1.656900E-02, 5.598300E-02, 1.394030E-01, 2.443780E-01, 3.081070E-01, 2.972660E-01, 2.136850E-01, 9.341400E-02, 1.248300E-02
c, 1.11, -4.110000E-04, -3.774000E-03, -1.931600E-02, -6.643700E-02, -1.705420E-01, -2.854770E-01, -2.328840E-01, 9.447100E-02, 4.201780E-01, 4.094740E-01, 1.040510E-01
c, 1.11, -8.270000E-04, -7.041000E-03, -3.926800E-02, -1.333410E-01, -4.342300E-01, -2.565540E-01, 9.662400E-01, 1.097980E-01, -1.047035E+00, 4.375990E-01, 3.852040E-01
c, 1.11, -5.450000E-04, -5.028000E-03, -2.594500E-02, -9.110400E-02, -2.453950E-01, -3.481600E-01, 7.008300E-02, 6.494450E-01, 1.939420E-01, -6.184680E-01, -2.610280E-01
c, 11.11, 1.000000E+00
c, 12.12, 1.0000000
f, CO , 6.578395800389E+00, 1.448104823051E+00, 4.162171776866E-01
c, 1.1, 1.000000E+00
c, 2.2, 1.000000E+00
c, 3.3, 1.000000E+00
g, CO , 3.947480560610E+00, 7.181414595113E-01
c, 1.1, 1.000000E+00
c, 2.2, 1.000000E+00

```
! nickel          (23s,19p,12d,3f,2g) -> [9s,8p,6d,3f,2g]
s, NI , 1.550302E+07, 2.321071E+06, 5.281802E+05, 1.496040E+05, 4.880907E+04, 1.762239E+04, 6.874201E+03, 2.851857E+03, 1.244334E+03, 5.661547E+02, 2.667873E+02,
    1.293752E+02, 6.365161E+01, 2.933302E+01, 1.482212E+01, 7.460513E+00, 3.413576E+00, 1.632884E+00, 7.435790E-01, 1.725920E-01, 7.742000E-02, 3.370100E-02, 0.0146700
c, 1.22, 2.085023E-06, 1.621425E-05, 8.530773E-05, 3.603793E-04, 1.312632E-03, 4.272395E-03, 1.265994E-02, 3.423065E-02, 8.326735E-02, 1.745758E-01, 2.898023E-01,
    3.207323E-01, 1.778265E-01, 3.633007E-02, 2.089458E-02, 1.596954E-02, 1.730245E-03, 4.817365E-04, 1.246800E-04, 2.340378E-05, -1.348962E-05, 4.799539E-06
c, 1.22, -7.298155E-07, -5.677768E-06, -2.985922E-05, -1.262784E-04, -4.600252E-04, -1.503230E-03, -4.479171E-03, -1.232512E-02, -3.097639E-02, -6.998651E-02, -1.339256E-01,
    -1.966881E-01, -1.402258E-01, 1.960730E-01, 5.564517E-01, 3.702167E-01, 5.396034E-02, 4.706492E-03, 3.736115E-03, -7.468050E-05, 9.575336E-05, -3.437115E-05
c, 1.22, 2.453237E-07, 1.909637E-06, 1.003489E-05, 4.248835E-05, 1.546187E-04, 5.064550E-04, 1.508840E-03, 4.178816E-03, 1.056668E-02, 2.439300E-02, 4.812101E-02,
    7.565170E-02, 5.748534E-02, -9.783186E-02, -3.864536E-01, -3.606598E-01, 2.888650E-01, 6.830917E-01, 3.206778E-01, 1.651471E-02, 3.124693E-04, 5.347675E-03
c, 1.22, -5.114261E-08, -3.980779E-07, -2.092033E-06, -8.856836E-06, -3.223602E-05, -1.055757E-04, -3.146356E-04, -8.714113E-04, -2.205415E-03, -5.095517E-03, -1.008237E-02,
    -1.592070E-02, -1.222037E-02, 2.115869E-02, 8.747304E-02, 8.511605E-02, -8.115683E-02, -2.166359E-01, -2.076958E-01, 2.699296E-01, 5.842711E-01, 2.957903E-01
c, 1.22, -1.771733E-07, -1.397615E-06, -7.203859E-06, -3.128552E-05, -1.102699E-04, -3.755735E-04, -1.067900E-03, -3.128427E-03, -7.410272E-03, -1.859501E-02, -3.322798E-02,
    -6.118440E-02, -3.286334E-02, 4.844202E-02, 3.989880E-01, 3.379290E-01, -7.016224E-01, -1.694434E+00, 2.582680E+00, -5.498758E-01, -1.394551E+00, 1.361618E+00
c, 1.22, -9.762069E-08, -7.741142E-07, -3.959746E-06, -1.736914E-05, -6.044953E-05, -2.090555E-04, -5.834312E-04, -1.746713E-03, -4.026784E-03, -1.042362E-02, -1.781172E-02,
    -3.466996E-02, -1.540945E-02, 2.086455E-02, 2.206820E-01, 1.073966E-01, -8.310668E-02, -8.942999E-01, 3.750141E-01, 1.496753E+00, -5.320390E-01, -8.263939E-01
c, 22.22, 1.000000E+00
c, 1.22, -1.988311E-07, -1.449716E-06, -8.363444E-06, -3.125532E-05, -1.327624E-04, -3.589662E-04, -1.341488E-03, -2.824018E-03, -9.863607E-03, -1.523481E-02, -4.984489E-02,
    -3.522354E-02, -1.056424E-01, 2.138034E-01, 1.517675E-01, 1.180428E+00, -3.526490E+00, 2.550815E+00, 4.306461E-01, -3.450041E+00, 4.740342E+00, -2.312156E+00
c, 23.23, 1.0000000
p, NI , 4.506662E+04, 1.066264E+04, 3.463431E+03, 1.326624E+03, 5.647414E+02, 2.588288E+02, 1.253115E+02, 6.324233E+01, 3.282406E+01, 1.739692E+01, 9.324394E+00,
    4.938839E+00, 2.562025E+00, 1.299294E+00, 6.346780E-01, 2.322620E-01, 9.154600E-02, 3.564300E-02, 0.0138800
c, 1.18, 1.100000E-05, 9.600000E-05, 5.640000E-04, 2.570000E-03, 9.677000E-03, 3.074200E-02, 8.206500E-02, 1.768450E-01, 2.911620E-01, 3.308160E-01, 2.088190E-01,
    5.373900E-02, 1.709000E-03, -1.798000E-03, -1.046000E-03, -6.200000E-05, 1.000000E-07, -1.000000E-06
c, 1.18, -4.000000E-06, -3.500000E-05, -2.020000E-04, -9.250000E-04, -3.499000E-03, -1.128000E-02, -3.081900E-02, -6.903600E-02, -1.187330E-01, -1.424430E-01, -4.310700E-02,
    2.034990E-01, 4.062280E-01, 3.709380E-01, 1.643120E-01, 1.726500E-02, -1.682000E-03, 6.510000E-04
c, 1.18, 2.000000E-06, 1.400000E-05, 8.400000E-05, 3.710000E-04, 1.460000E-03, 4.537000E-03, 1.292200E-02, 2.776600E-02, 5.095600E-02, 5.674500E-02, 2.411900E-02,
    -1.250870E-01, -2.065410E-01, -2.149830E-01, 2.078190E-01, 6.385420E-01, 3.262840E-01, 1.298100E-02
c, 1.18, 4.000000E-06, 3.600000E-05, 1.990000E-04, 9.590000E-04, 3.463000E-03, 1.175600E-02, 3.069000E-02, 7.308100E-02, 1.168270E-01, 1.911630E-01, 1.065430E-01,
    -9.499240E-01, -5.636050E-01, 1.934611E+00, -5.101770E-01, -1.172297E+00, 1.015898E+00, 2.379800E-02
c, 1.18, 3.000000E-06, 2.400000E-05, 1.550000E-04, 6.460000E-04, 2.689000E-03, 7.899000E-03, 2.391200E-02, 4.800600E-02, 9.621300E-02, 9.758200E-02, 7.702200E-02,
    -3.601360E-01, -4.455890E-01, -1.165380E-01, 1.170503E+00, -3.893100E-02, -7.688140E-01, -5.941000E-03
c, 1.18, 1.000000E-06, 7.000000E-06, 4.100000E-05, 1.860000E-04, 7.120000E-04, 2.269000E-03, 6.291000E-03, 1.393400E-02, 2.453100E-02, 2.877000E-02, 8.781000E-03,
    -5.173100E-02, -9.594500E-02, -1.106750E-01, 2.727000E-03, 3.150270E-01, 5.428100E-01, 2.757520E-01
c, 18.18, 1.000000E+00
c, 19.19, 1.0000000
d, NI , 4.143427E+02, 1.243926E+02, 4.811540E+01, 2.084200E+01, 9.622100E+00, 4.603400E+00, 2.196300E+00, 1.020600E+00, 4.544000E-01, 1.900000E-01, 7.150000E-02, 0.0269100
c, 1.11, 3.500000E-04, 3.200000E-03, 1.647700E-02, 5.600100E-02, 1.392340E-01, 2.427760E-01, 3.040690E-01, 2.933190E-01, 2.172590E-01, 1.046990E-01, 1.733900E-02
c, 1.11, -3.620000E-04, -3.335000E-03, -1.717600E-02, -5.924500E-02, -1.512320E-01, -2.544610E-01, -2.287180E-01, 2.061600E-02, 3.427530E-01, 4.738710E-01, 1.834350E-01
c, 1.11, -5.290000E-04, -4.928000E-03, -2.537200E-02, -8.965400E-02, -2.387440E-01, -3.597210E-01, 4.444000E-03, 5.965470E-01, 3.313020E-01, -5.090090E-01, -3.501390E-01
c, 1.11, -7.870000E-04, -6.778000E-03, -3.772600E-02, -1.291250E-01, -4.148090E-01, -2.839570E-01, 8.671940E-01, 2.723270E-01, -9.868420E-01, 1.853600E-01, 5.026100E-01
c, 11.11, 1.000000E+00
c, 12.12, 1.0000000
f, NI , 7.638533717491E+00, 1.681163684256E+00, 5.031645086469E-01
c, 1.1, 1.000000E+00
c, 2.2, 1.000000E+00
c, 3.3, 1.000000E+00
g, NI , 4.497798548933E+00, 7.398096637840E-01
c, 1.1, 1.000000E+00
c, 2.2, 1.000000E+00
```

```
! copper              (23s,19p,12d,3f,2g) -> [9s,8p,6d,3f,2g]
s, CU , 1.666549E+07, 2.495213E+06, 5.678507E+05, 1.608531E+05, 5.248186E+04, 1.894885E+04, 7.391655E+03, 3.066517E+03, 1.337994E+03, 6.087830E+02, 2.868980E+02,
        1.391530E+02, 6.849455E+01, 3.160108E+01, 1.599158E+01, 8.056510E+00, 3.690329E+00, 1.762902E+00, 8.012690E-01, 1.842430E-01, 8.179300E-02, 3.530300E-02, 0.0152400
c, 1.22, 1.922210E-06, 1.494655E-05, 7.863106E-05, 3.321189E-04, 1.209649E-03, 3.936231E-03, 1.166031E-02, 3.149512E-02, 7.646385E-02, 1.595162E-01, 2.618983E-01,
        2.816708E-01, 1.509587E-01, 7.041955E-02, 1.180039E-01, 8.015187E-02, 1.020122E-02, -3.720464E-04, -2.206886E-05, -2.401085E-05, 1.529816E-05, -5.151167E-06
c, 1.22, -1.082642E-06, -8.420869E-06, -4.428625E-05, -1.872071E-04, -6.819265E-04, -2.225485E-03, -6.619620E-03, -1.811400E-02, -4.506622E-02, -9.955443E-02, -1.830558E-01,
        -2.510577E-01, -1.703417E-01, 1.900077E-01, 5.507048E-01, 3.642532E-01, 4.868977E-02, -3.799993E-03, -3.156308E-04, -2.430411E-04, 1.553645E-04, -5.180256E-05
c, 1.22, 2.389605E-07, 1.860009E-06, 9.772580E-06, 4.137619E-05, 1.505588E-04, 4.932175E-04, 1.469467E-03, 4.071487E-03, 1.030091E-02, 2.381408E-02, 4.707008E-02,
        7.424450E-02, 5.657657E-02, -9.760862E-02, -3.856399E-01, -3.540312E-01, 2.983959E-01, 6.861809E-01, 3.150989E-01, 1.394564E-02, -3.611640E-03, 2.289115E-03
c, 1.22, -4.823436E-08, -3.753858E-07, -1.972743E-06, -8.349961E-06, -3.039535E-05, -9.953010E-05, -2.967188E-04, -8.217293E-04, -2.081377E-03, -4.811149E-03, -9.537683E-03,
        -1.507351E-02, -1.161088E-02, 2.032716E-02, 8.327403E-02, 7.953125E-02, -7.901524E-02, -2.054312E-01, -1.985134E-01, 2.627133E-01, 5.794031E-01, 3.054149E-01
c, 1.22, -1.849356E-07, -1.461905E-06, -7.510534E-06, -3.274989E-05, -1.148239E-04, -3.935903E-04, -1.110617E-03, -3.284174E-03, -7.697235E-03, -1.959013E-02, -3.444712E-02,
        -6.507176E-02, -3.269416E-02, 4.738915E-02, 4.333712E-01, 3.189521E-01, -7.281955E-01, -1.663116E+00, 2.513267E+00, -3.736861E-01, -1.479102E+00, 1.315894E+00
c, 1.22, -8.876635E-08, -7.032135E-07, -3.601366E-06, -1.576871E-05, -5.499726E-05, -1.897055E-04, -5.311656E-04, -1.584593E-03, -3.671316E-03, -9.458893E-03, -1.630134E-02,
        -3.145402E-02, -1.444712E-02, 2.037856E-02, 1.997307E-01, 9.475926E-02, -7.935398E-02, -7.591353E-01, 2.365079E-01, 1.422604E+00, -3.066203E-01, -9.619310E-01
c, 22.22, 1.000000E+00
c, 1.22, -1.980951E-07, -1.446191E-06, -8.326308E-06, -3.119380E-05, -1.320842E-04, -3.585362E-04, -1.333996E-03, -2.824728E-03, -9.806333E-03, -1.529323E-02, -4.956573E-02,
        -3.583468E-02, -1.044906E-01, 2.105854E-01, 1.646732E-01, 1.150513E+00, -3.483732E+00, 2.516636E+00, 4.429064E-01, -3.404310E+00, 4.627850E+00, -2.240120E+00
c, 23.23, 1.0000000
p, CU , 4.821841E+04, 1.142055E+04, 3.713569E+03, 1.423762E+03, 6.066041E+02, 2.782564E+02, 1.348449E+02, 6.812254E+01, 3.540088E+01, 1.879029E+01, 1.008356E+01,
        5.346120E+00, 2.775228E+00, 1.407564E+00, 6.872130E-01, 2.468220E-01, 9.622000E-02, 3.703700E-02, 0.0142600
c, 1.18, 1.100000E-05, 9.700000E-05, 5.690000E-04, 2.589000E-03, 9.742000E-03, 3.093000E-02, 8.253000E-02, 1.777580E-01, 2.922250E-01, 3.303490E-01, 2.068630E-01,
        5.279300E-02, 1.718000E-03, -1.701000E-03, -9.760000E-04, -5.400000E-05, 1.000000E-07, -1.000000E-06
c, 1.18, -4.000000E-06, -3.500000E-05, -2.060000E-04, -9.410000E-04, -3.556000E-03, -1.146100E-02, -3.130900E-02, -7.013900E-02, -1.205620E-01, -1.435520E-01, -4.032700E-02,
        2.084660E-01, 4.087240E-01, 3.682010E-01, 1.596530E-01, 1.595500E-02, -1.681000E-03, 6.310000E-04
c, 1.18, 2.000000E-06, 1.300000E-05, 7.900000E-05, 3.490000E-04, 1.373000E-03, 4.261000E-03, 1.214600E-02, 2.606400E-02, 4.790700E-02, 5.282900E-02, 2.157500E-02,
        -1.177830E-01, -1.883970E-01, -2.019160E-01, 1.737430E-01, 6.368480E-01, 3.515180E-01, 1.238300E-02
c, 1.18, 3.000000E-06, 2.500000E-05, 1.630000E-04, 6.760000E-04, 2.808000E-03, 8.255000E-03, 2.494600E-02, 5.016000E-02, 1.002890E-01, 1.023190E-01, 7.724100E-02,
        -3.844490E-01, -4.513540E-01, -9.477800E-02, 1.173118E+00, -5.413300E-02, -7.282440E-01, 4.270000E-04
c, 1.18, 4.000000E-06, 3.700000E-05, 1.900000E-04, 9.780000E-04, 3.308000E-03, 1.198000E-02, 2.920700E-02, 7.512200E-02, 1.078350E-01, 2.073790E-01, 6.040600E-02,
        -8.949770E-01, -5.584020E-01, 1.899706E+00, -5.022240E-01, -1.182413E+00, 1.044507E+00, 1.223600E-02
c, 1.18, 1.000000E-06, 7.000000E-06, 4.000000E-05, 1.820000E-04, 6.970000E-04, 2.220000E-03, 6.163000E-03, 1.363100E-02, 2.403300E-02, 2.786200E-02, 7.975000E-03,
        -5.113300E-02, -9.230500E-02, -1.064580E-01, 3.738000E-03, 3.105410E-01, 5.419030E-01, 2.825020E-01
c, 18.18, 1.000000E+00
c, 19.19, 1.0000000
d, CU , 5.278860E+02, 1.587330E+02, 6.155560E+01, 2.688340E+01, 1.251750E+01, 6.069770E+00, 2.958319E+00, 1.414122E+00, 6.533290E-01, 2.866920E-01, 1.150140E-01, 0.0461400
c, 1.11, 2.560000E-04, 2.378000E-03, 1.272200E-02, 4.501200E-02, 1.171960E-01, 2.185550E-01, 2.914480E-01, 2.971380E-01, 2.360920E-01, 1.314440E-01, 3.434500E-02
c, 1.11, -3.160000E-04, -2.958000E-03, -1.587700E-02, -5.686400E-02, -1.535840E-01, -2.811390E-01, -2.685180E-01, 2.313000E-02, 3.566200E-01, 4.184700E-01, 1.928270E-01
c, 1.11, -5.930000E-04, -5.106000E-03, -2.992200E-02, -1.032930E-01, -3.595680E-01, -4.128070E-01, 7.476890E-01, 5.425150E-01, -9.777060E-01, -8.860300E-02, 6.550700E-01
c, 1.11, 3.790000E-04, 3.573000E-03, 1.922900E-02, 6.990300E-02, 1.993800E-01, 3.488480E-01, 5.139400E-02, -5.596790E-01, -3.845810E-01, 4.389250E-01, 4.904980E-01
c, 11.11, 1.000000E+00
c, 12.12, 1.0000000
f, CU , 9.327172310327E+00, 2.405858052241E+00, 6.157862680964E-01
c, 1.1, 1.000000E+00
c, 2.2, 1.000000E+00
c, 3.3, 1.000000E+00
g, CU , 5.138424739870E+00, 8.075696705149E-01
c, 1.1, 1.000000E+00
c, 2.2, 1.000000E+00
```

```
! zinc                (23s,19p,12d,3f,2g) -> [9s,8p,6d,3f,2g]
s, ZN , 1.785293E+07, 2.673038E+06, 6.083244E+05, 1.723176E+05, 5.622269E+04, 2.029985E+04, 7.918871E+03, 3.285340E+03, 1.433524E+03, 6.522889E+02, 3.074360E+02,
        1.491466E+02, 7.345108E+01, 3.392562E+01, 1.718947E+01, 8.666595E+00, 3.972505E+00, 1.894665E+00, 8.593850E-01, 1.952560E-01, 8.585400E-02, 3.678100E-02, 0.0157600
c, 1.22, 2.106603E-06, 1.638051E-05, 8.617178E-05, 3.640028E-04, 1.325778E-03, 4.315394E-03, 1.278917E-02, 3.459541E-02, 8.423468E-02, 1.769497E-01, 2.947718E-01,
        3.286054E-01, 1.837326E-01, 2.845640E-02, -1.562471E-03, 1.057694E-03, -4.832147E-04, 1.795943E-04, -7.198366E-05, 2.200105E-05, -1.565510E-05, 4.980717E-06
c, 1.22, -6.503871E-07, -5.059606E-06, -2.660362E-05, -1.125168E-04, -4.098844E-04, -1.340085E-03, -3.996271E-03, -1.102397E-02, -2.783658E-02, -6.352200E-02, -1.235647E-01,
        -1.860330E-01, -1.345412E-01, 2.026370E-01, 5.627948E-01, 3.687315E-01, 5.055865E-02, -2.856100E-04, 1.213965E-03, -1.706895E-04, 1.266229E-04, -4.036833E-05
c, 1.22, 2.455424E-07, 1.911263E-06, 1.004137E-05, 4.251710E-05, 1.546957E-04, 5.068054E-04, 1.509742E-03, 4.183744E-03, 1.058425E-02, 2.447935E-02, 4.837945E-02,
        7.630985E-02, 5.798021E-02, -1.010517E-01, -3.950702E-01, -3.534286E-01, 3.070109E-01, 6.935144E-01, 3.040583E-01, 1.003318E-02, -4.007622E-03, 1.059799E-03
c, 1.22, -1.379282E-07, -1.098688E-06, -5.581627E-06, -2.469757E-05, -8.499700E-05, -2.979550E-04, -8.180999E-04, -2.498043E-03, -5.630866E-03, -1.501607E-02, -2.479935E-02,
        -5.092472E-02, -1.923797E-02, 2.480840E-02, 3.402582E-01, 1.317501E-01, -2.092734E-01, -1.289970E+00, 9.898685E-01, 1.477960E+00, -1.234764E+00, -2.308975E-01
c, 1.22, 4.916948E-08, 3.829348E-07, 2.010287E-06, 8.520784E-06, 3.096268E-05, 1.016040E-04, 3.021322E-04, 8.393851E-04, 2.119040E-03, 4.923291E-03, 9.713665E-03,
        1.549413E-02, 1.170570E-02, -2.068558E-02, -8.652477E-02, -7.840824E-02, 7.950591E-02, 2.182560E-01, 1.957366E-01, -3.195337E-01, -5.833695E-01, -2.511224E-01
c, 1.22, -1.951222E-07, -1.513488E-06, -7.991991E-06, -3.361493E-05, -1.233416E-04, -4.000292E-04, -1.206705E-03, -3.298144E-03, -8.503531E-03, -1.932075E-02, -3.951180E-02,
        -6.074193E-02, -5.154698E-02, 9.063520E-02, 3.930253E-01, 5.283548E-01, -1.462611E+00, -8.366769E-01, 2.615168E+00, -2.241322E+00, 7.597394E-01, 5.193961E-01
c, 1.22, -2.631275E-07, -1.954514E-06, -1.098059E-05, -4.252529E-05, -1.728905E-04, -4.940605E-04, -1.731281E-03, -3.952778E-03, -1.260194E-02, -2.205362E-02, -6.268701E-02,
        -5.878225E-02, -1.200370E-01, 2.236128E-01, 4.980742E-01, 1.191580E+00, -5.649063E+00, 6.843489E+00, -3.320100E+00, -2.249601E-01, 1.902154E+00, -1.424758E+00
c, 22.22, 1.000000E+00
c, 23.23, 1.0000000
p, ZN , 5.286793E+04, 1.251179E+04, 4.065286E+03, 1.557595E+03, 6.632892E+02, 3.041561E+02, 1.473680E+02, 7.445469E+01, 3.871493E+01, 2.057022E+01, 1.105425E+01,
        5.874661E+00, 3.052936E+00, 1.547225E+00, 7.541510E-01, 2.653490E-01, 1.020670E-01, 3.875500E-02, 0.0147200
c, 1.18, 1.100000E-05, 9.400000E-05, 5.510000E-04, 2.511000E-03, 9.470000E-03, 3.016900E-02, 8.085500E-02, 1.751390E-01, 2.897160E-01, 3.298490E-01, 2.092940E-01,
        5.625900E-02, 5.052000E-03, 9.970000E-04, 1.870000E-04, 5.100000E-05, -1.300000E-05, 4.000000E-06
c, 1.18, -4.000000E-06, -3.500000E-05, -2.060000E-04, -9.420000E-04, -3.570000E-03, -1.154000E-02, -3.165200E-02, -7.128500E-02, -1.232840E-01, -1.476380E-01, -4.447700E-02,
        2.053650E-01, 4.100860E-01, 3.721450E-01, 1.581180E-01, 1.454000E-02, -1.608000E-03, 6.010000E-04
c, 1.18, 3.000000E-06, 2.400000E-05, 1.590000E-04, 6.460000E-04, 2.747000E-03, 7.938000E-03, 2.460100E-02, 4.857600E-02, 1.006820E-01, 9.846800E-02, 8.623500E-02,
        -3.793800E-01, -4.203930E-01, -1.825620E-01, 1.177887E+00, 5.197400E-02, -7.444770E-01, 4.419000E-03
c, 1.18, 1.000000E-06, 1.100000E-05, 6.700000E-05, 2.960000E-04, 1.157000E-03, 3.634000E-03, 1.030700E-02, 2.248200E-02, 4.103300E-02, 4.646000E-02, 1.814600E-02,
        -9.436400E-02, -1.601770E-01, -1.808950E-01, 9.482600E-02, 6.291530E-01, 4.083180E-01, 1.197700E-02
c, 1.18, -3.000000E-06, -3.600000E-05, -1.740000E-04, -9.530000E-04, -3.047000E-03, -1.173600E-02, -2.701600E-02, -7.479800E-02, -9.823700E-02, -2.150840E-01, -3.221400E-02,
        7.563080E-01, 7.019320E-01, -1.814187E+00, 2.914540E-01, 1.295623E+00, -1.067089E+00, -1.193500E-02
c, 1.18, 1.000000E-06, 6.000000E-06, 3.800000E-05, 1.730000E-04, 6.640000E-04, 2.120000E-03, 5.916000E-03, 1.315300E-02, 2.337200E-02, 2.722100E-02, 8.334000E-03,
        -4.886000E-02, -8.889400E-02, -1.036180E-01, 1.051000E-03, 3.030640E-01, 5.427240E-01, 2.921230E-01
c, 18.18, 1.000000E+00
c, 19.19, 1.0000000
d, ZN , 6.397806E+02, 1.926742E+02, 7.484368E+01, 3.286048E+01, 1.539353E+01, 7.525512E+00, 3.715429E+00, 1.806459E+00, 8.529730E-01, 3.847800E-01, 1.593370E-01, 0.0659800
c, 1.11, 2.080000E-04, 1.954000E-03, 1.071100E-02, 3.905700E-02, 1.048480E-01, 2.045690E-01, 2.852080E-01, 3.014460E-01, 2.462280E-01, 1.390160E-01, 3.626900E-02
c, 1.11, -2.900000E-04, -2.738000E-03, -1.511100E-02, -5.564300E-02, -1.558640E-01, -3.034890E-01, -3.062760E-01, 3.339500E-02, 4.000750E-01, 3.899720E-01, 1.364320E-01
c, 1.11, -3.170000E-04, -3.056000E-03, -1.675300E-02, -6.271200E-02, -1.879800E-01, -3.720840E-01, -2.343900E-02, 6.054700E-01, 3.230630E-01, -4.713720E-01, -4.838860E-01
c, 1.11, 4.520000E-04, 3.879000E-03, 2.357000E-02, 8.252600E-02, 2.972800E-01, 4.368320E-01, -6.042900E-01, -6.746050E-01, 9.387020E-01, 2.779680E-01, -8.326530E-01
c, 11.11, 1.000000E+00
c, 12.12, 1.0000000
f, ZN , 1.126626597804E+01, 3.057806503476E+00, 7.437380486770E-01
c, 1.1, 1.000000E+00
c, 2.2, 1.000000E+00
c, 3.3, 1.000000E+00
g, ZN , 5.631057992154E+00, 8.207861131168E-01
c, 1.1, 1.000000E+00
c, 2.2, 1.000000E+00
```

cc-pVTZ-PP-F12-wis Basis Set: Y-Cd and Hf-Hg Atoms

```
! yttrium              (15s,12p,11d,3f,2g) -> [7s,7p,6d,3f,2g]
s, Y , 5177.1300000, 803.7880000, 176.8550000, 24.7094000, 15.4311000, 9.6370600, 3.6556900, 2.1887900, 0.9669010, 0.5203350, 0.2687380, 0.0934310, 0.0442780, 0.0205860, 0.0096000
c, 1.13, 0.0000060, 0.0000420, 0.0001850, 0.0068560, -0.0514100, 0.1537220, -0.3427500, -0.2814160, 0.4317850, 0.6443500, 0.2560410, 0.0142160, -0.0008570
c, 1.13, -0.0000030, -0.0000340, -0.0000560, -0.0065930, 0.0389400, -0.1029080, 0.2703540, 0.0618350, -0.1353620, -1.2866270, 0.5668990, 2.0738270, -1.1966140
c, 1.13, -0.0000020, -0.0000130, -0.0000630, -0.0020340, 0.0156890, -0.0487030, 0.1209540, 0.0857950, -0.1647410, -0.3133730, -0.2505130, 0.3309260, 0.6689400
c, 1.13, -0.0000040, -0.0000520, -0.0000830, -0.0098760, 0.0577990, -0.1538970, 0.4217130, 0.1309820, -0.5555320, -2.5771630, 4.1711070, -1.4383750, -1.6047110
c, 1.13, -0.0000120, 0.0000460, -0.0006750, 0.0212080, -0.0598710, 0.0151140, -0.0377770, 1.1622360, -3.2770720, 1.2701190, 3.1537770, -6.7129510, 7.3005270
c, 14.14, 1.0000000
c, 15.15, 1.0000000
p, Y , 107.4110000, 7.8253700, 4.8896400, 2.6346700, 1.2906400, 0.6865630, 0.3642540, 0.1880790, 0.0906470, 0.0433930, 0.0203400, 0.0095000
c, 1.10, 0.0000910, 0.0412730, -0.1207510, -0.0913090, 0.2467110, 0.4542820, 0.3496890, 0.1146010, 0.0109550, 0.0016130
c, 1.10, -0.0000790, -0.0378270, 0.1192150, 0.0674820, -0.2648700, -1.1887760, 1.3402300, 1.1973990, -2.3967800, 0.7039560
c, 1.10, -0.0000650, -0.0284960, 0.0852470, 0.0687010, -0.1845780, -0.6131370, -0.2279870, 1.3845570, 0.1841280, -0.8904790
c, 1.10, -0.0000300, -0.0128200, 0.0389180, 0.0262170, -0.0850530, -0.1644870, -0.1767940, 0.0390610, 0.4379790, 0.5157770
c, 1.10, -0.0001660, -0.0512630, 0.1531410, 0.2656770, -1.1525810, -1.1729520, 4.9116730, -5.0069800, 1.9625810, 0.7231000
c, 11.11, 1.0000000
c, 12.12, 1.0000000
d, Y , 44.4707000, 12.2260000, 5.0145900, 1.6657600, 0.8894000, 0.4492320, 0.2178540, 0.1023240, 0.0468400, 0.0205940, 0.0091000
c, 1.9, 0.0001300, 0.0012450, -0.0064180, 0.0515240, 0.1608100, 0.2592560, 0.3131190, 0.2970510, 0.1865630
c, 1.9, 0.0001980, 0.0019390, -0.0100050, 0.0955710, 0.2915650, 0.4281810, -0.3428950, -0.8129610, 0.3614820
c, 1.9, -0.0001640, -0.0014080, 0.0073280, -0.0672140, -0.1989690, -0.3268610, -0.2274790, 0.2344810, 0.5414920
c, 1.9, -0.0003660, -0.0021570, 0.0129660, -0.1214120, -0.6795100, 0.0093200, 1.4118300, -0.9492730, -0.5277300
c, 10.10, 1.0000000
c, 11.11, 1.0000000
f, Y , 9.796704307509E-01, 2.865695224740E-01, 9.583849827605E-02
c, 1.1, 1.000000E+00
c, 2.2, 1.000000E+00
c, 3.3, 1.000000E+00
g, Y , 4.097321098135E-01, 1.380288914329E-01
c, 1.1, 1.000000E+00
c, 2.2, 1.000000E+00
```

```
! zirconium            (15s,12p,11d,3f,2g) -> [7s,7p,6d,3f,2g]
s, ZR , 5675.2400000, 863.1340000, 190.3400000, 26.9090000, 16.8039000, 10.4957000, 3.9316800, 2.4065300, 1.0562800, 0.5710270, 0.2958580, 0.1043250, 0.0485630, 0.0223560,
        0.0103000
c, 1.13, 0.0000060, 0.0000460, 0.0001980, 0.0070570, -0.0530490, 0.1577920, -0.3577070, -0.2830480, 0.4624190, 0.6391000, 0.2418360, 0.0136140, -0.0007280
c, 1.13, -0.0000030, -0.0000400, -0.0000710, -0.0072040, 0.0429900, -0.1141280, 0.3101060, 0.0689160, -0.2137110, -1.4303860, 0.8787700, 1.8905350, -1.2450350
c, 1.13, -0.0000020, -0.0000150, -0.0000680, -0.0021350, 0.0164650, -0.0508100, 0.1293950, 0.0858250, -0.1802650, -0.3258040, -0.2273070, 0.3456950, 0.6582420
c, 1.13, -0.0000060, -0.0000510, -0.0001690, -0.0077740, 0.0527550, -0.1563380, 0.4515910, 0.2384230, -1.1251000, -2.0104270, 4.3180980, -2.3194350, -0.6481260
c, 1.13, -0.0000160, 0.0000580, -0.0008910, 0.0273980, -0.0769940, 0.0179110, -0.0205690, 1.4847690, -5.2993640, 5.0797270, -0.0139520, -4.7172460, 6.0430530
c, 14.14, 1.0000000
c, 15.15, 1.0000000
p, ZR , 122.6140000, 8.6017600, 5.3756700, 2.8732600, 1.4314800, 0.7585420, 0.3983330, 0.2005520, 0.0942630, 0.0439730, 0.0202460, 0.0093000
c, 1.10, 0.0000890, 0.0431890, -0.1256900, -0.0984530, 0.2616530, 0.4684370, 0.3431180, 0.1015390, 0.0077750, 0.0014680
c, 1.10, -0.0000420, -0.0186700, 0.0562710, 0.0412580, -0.1303570, -0.2424890, -0.2520430, 0.2222450, 0.6873330, 0.2793880
c, 1.10, -0.0000720, -0.0353270, 0.1073430, 0.0781030, -0.2483300, -0.7742400, 0.1204770, 1.5143590, -0.4188840, -0.7176520
c, 1.10, -0.0000290, -0.0132920, 0.0402610, 0.0278420, -0.0900280, -0.1740550, -0.1576400, 0.0813280, 0.4341340, 0.4929950
c, 1.10, -0.0001010, -0.0557630, 0.1714680, 0.1546890, -0.7228250, -1.2765790, 2.9768200, -1.0541120, -1.4600030, 1.3306580
c, 11.11, 1.0000000
c, 12.12, 1.0000000
d, ZR , 48.6431000, 13.5835000, 5.3945300, 1.8630000, 1.0079200, 0.5198200, 0.2594550, 0.1255900, 0.0589320, 0.0262290, 0.0117000
c, 1.9, 0.0001600, 0.0015130, -0.0091330, 0.0638690, 0.1893790, 0.2877480, 0.3152610, 0.2608760, 0.1415580
c, 1.9, 0.0003110, 0.0020970, -0.0135190, 0.1372450, 0.3797260, 0.3322720, -0.5609850, -0.6443880, 0.4851650
c, 1.9, -0.0004680, -0.0028050, 0.0199580, -0.2202750, -0.7531930, 0.4522480, 1.1905720, -1.2301770, -0.2159390
c, 1.9, -0.0001990, -0.0016270, 0.0100510, -0.0817780, -0.2318160, -0.3431630, -0.1316050, 0.3309010, 0.5074600
c, 10.10, 1.0000000
c, 11.11, 1.0000000
f, ZR , 1.423082244223E+00, 4.115759601415E-01, 1.378383291383E-01
c, 1.1, 1.000000E+00
c, 2.2, 1.000000E+00
c, 3.3, 1.000000E+00
g, ZR , 5.680414142306E-01, 1.893128092000E-01
c, 1.1, 1.000000E+00
c, 2.2, 1.000000E+00
```

```
! niobium             (15s,12p,11d,3f,2g) -> [7s,7p,6d,3f,2g]
s, NB , 5729.3600000, 898.7570000, 203.1540000, 29.0821000, 18.1596000, 11.3420000, 4.2245400, 2.6381700, 1.1509900, 0.6250000, 0.3244810, 0.1141370, 0.0524230, 0.0239040,
        0.0109000
c, 1.13, 0.0000080, 0.0000520, 0.0002160, 0.0073960, -0.0552510, 0.1632580, -0.3721490, -0.2827570, 0.4840580, 0.6333390, 0.2343950, 0.0132460, -0.0007750
c, 1.13, -0.0000040, -0.0000460, -0.0000850, -0.0076920, 0.0460280, -0.1222600, 0.3382530, 0.0701100, -0.2716550, -1.4798190, 1.0436440, 1.7512880, -1.2255670
c, 1.13, -0.0000080, -0.0000580, -0.0002260, -0.0074160, 0.0539120, -0.1667540, 0.5029010, 0.2662620, -1.5007590, -1.5285370, 4.2085080, -2.6574950, -0.1836000
c, 1.13, -0.0000030, -0.0000170, -0.0000750, -0.0022520, 0.0172420, -0.0528630, 0.1363550, 0.0845410, -0.1912600, -0.3296300, -0.2097360, 0.3537950, 0.6495660
c, 1.13, -0.0000200, 0.0000500, -0.0009920, 0.0264760, -0.0672280, -0.0180330, 0.1081030, 1.5653000, -6.7398560, 7.9760920, -2.5603720, -2.9311650, 4.7735390
c, 14.14, 1.0000000
c, 15.15, 1.0000000
p, NB , 138.4600000, 9.3123800, 5.8201500, 3.0589600, 1.5902700, 0.8361380, 0.4360320, 0.2167160, 0.1018830, 0.0473870, 0.0217480, 0.0100000
c, 1.10, 0.0000870, 0.0460850, -0.1347720, -0.1053350, 0.2747760, 0.4792360, 0.3375730, 0.0941580, 0.0062330, 0.0014690
c, 1.10, -0.0000430, -0.0210770, 0.0638960, 0.0466950, -0.1449560, -0.2742540, -0.2375990, 0.2952600, 0.6708660, 0.2356770
c, 1.10, -0.0000730, -0.0390570, 0.1198810, 0.0864220, -0.2891420, -0.8052000, 0.2953950, 1.4185360, -0.5209570, -0.6615440
c, 1.10, -0.0000290, -0.0143860, 0.0438130, 0.0302870, -0.0963090, -0.1837030, -0.1496950, 0.1031560, 0.4396420, 0.4785870
c, 1.10, -0.0001020, -0.0630370, 0.1953600, 0.1871900, -0.9029120, -1.0655850, 3.0265000, -1.4246220, -1.1257930, 1.2566670
c, 11.11, 1.0000000
c, 12.12, 1.0000000
d, NB , 55.5243000, 15.8430000, 5.7920600, 2.1776800, 1.2209000, 0.6445770, 0.3278110, 0.1609180, 0.0761310, 0.0339110, 0.0151000
c, 1.9, 0.0001800, 0.0015650, -0.0100360, 0.0585180, 0.1897930, 0.2945090, 0.3174790, 0.2524380, 0.1316200
c, 1.9, 0.0003560, 0.0023390, -0.0159450, 0.1419710, 0.4109560, 0.3210380, -0.5989470, -0.6084580, 0.4610850
c, 1.9, -0.0005480, -0.0030070, 0.0229940, -0.2487660, -0.7513270, 0.5055700, 1.1509160, -1.1909780, -0.2850970
c, 1.9, -0.0002210, -0.0016600, 0.0108870, -0.0759490, -0.2327040, -0.3415990, -0.1222670, 0.3328420, 0.5027080
c, 10.10, 1.0000000
c, 11.11, 1.0000000
f, NB , 1.794816279274E+00, 5.240346449668E-01, 1.622631863748E-01
c, 1.1, 1.000000E+00
c, 2.2, 1.000000E+00
c, 3.3, 1.000000E+00
g, NB , 6.962897467544E-01, 2.121338626948E-01
c, 1.1, 1.000000E+00
c, 2.2, 1.000000E+00
```

```
! molybdenum           (15s,12p,11d,3f,2g) -> [7s,7p,6d,3f,2g]
s, MO , 6711.2300000, 988.2620000, 216.2910000, 31.1617000, 19.4654000, 12.1603000, 4.5171000, 2.8203200, 1.2602800, 0.6800130, 0.3514060, 0.1247460, 0.0566870, 0.0255690,
        0.0115000
c, 1.13, 0.0000080, 0.0000590, 0.0002440, 0.0077510, -0.0577500, 0.1702780, -0.3983930, -0.2761780, 0.5039470, 0.6368170, 0.2246810, 0.0120130, -0.0007040
c, 1.13, -0.0000050, -0.0000490, -0.0001130, -0.0076210, 0.0466430, -0.1260310, 0.3550690, 0.0788460, -0.3292100, -1.4622420, 1.1047470, 1.6428490, -1.1446510
c, 1.13, -0.0000090, -0.0000660, -0.0002910, -0.0072570, 0.0559090, -0.1790880, 0.5626060, 0.2990240, -1.8724510, -0.9933480, 3.9567310, -2.7368330, -0.0070370
c, 1.13, -0.0000030, -0.0000190, -0.0000840, -0.0023280, 0.0178340, -0.0546970, 0.1452280, 0.0805810, -0.1986470, -0.3318030, -0.1945790, 0.3483100, 0.6441110
c, 1.13, -0.0000180, -0.0000170, -0.0008440, 0.0129570, -0.0099490, -0.1236980, 0.4468930, 1.4039530, -7.7842940, 10.2859540, -4.7884940, -1.2294860, 3.4559250
c, 14.14, 1.0000000
c, 15.15, 1.0000000
p, MO , 152.9580000, 10.0454000, 6.2786900, 3.2529000, 1.7561900, 0.9164400, 0.4752100, 0.2340410, 0.1099870, 0.0509000, 0.0231980, 0.0106000
c, 1.10, 0.0000890, 0.0486580, -0.1429160, -0.1119240, 0.2872550, 0.4882670, 0.3325040, 0.0879450, 0.0051370, 0.0014690
c, 1.10, -0.0000460, -0.0234060, 0.0713700, 0.0523310, -0.1606860, -0.3056960, -0.2096950, 0.3498440, 0.6427110, 0.2113020
c, 1.10, -0.0000820, -0.0425660, 0.1316610, 0.1041920, -0.3540870, -0.8231200, 0.4691120, 1.3060670, -0.6005210, -0.6033790
c, 1.10, -0.0001570, -0.0617300, 0.1912730, 0.3034920, -1.2601600, -0.6601690, 2.9941100, -1.8628630, -0.6548950, 1.0766410
c, 1.10, -0.0000300, -0.0151180, 0.0462370, 0.0321610, -0.1003680, -0.1878280, -0.1412290, 0.1129270, 0.4365490, 0.4735680
c, 11.11, 1.0000000
c, 12.12, 1.0000000
d, MO , 64.0194000, 17.5538000, 6.5001600, 2.4048200, 1.3421000, 0.7078230, 0.3603360, 0.1770770, 0.0836650, 0.0370260, 0.0164000
c, 1.9, 0.0001670, 0.0016530, -0.0132930, 0.0670070, 0.2091290, 0.3096950, 0.3139960, 0.2323510, 0.1119340
c, 1.9, 0.0003680, 0.0022850, -0.0210710, 0.1657440, 0.4848470, 0.2113940, -0.7066180, -0.4649570, 0.5321680
c, 1.9, -0.0003500, -0.0041070, 0.0359670, -0.2938650, -0.8126500, 0.8503400, 0.8690250, -1.3237980, 0.0049480
c, 1.9, -0.0001970, -0.0017110, 0.0142690, -0.0826730, -0.2573070, -0.3434210, -0.0548000, 0.3765310, 0.4797980
c, 10.10, 1.0000000
c, 11.11, 1.0000000
f, MO , 2.686766966146E+00, 6.314369615584E-01, 1.284665318979E-01
c, 1.1, 1.000000E+00
c, 2.2, 1.000000E+00
c, 3.3, 1.000000E+00
g, MO , 9.310221212125E-01, 2.663606095021E-01
c, 1.1, 1.000000E+00
c, 2.2, 1.000000E+00
```

! technetium (15s,12p,11d,3f,2g) -> [7s,7p,6d,3f,2g]
s, TC , 7011.0800000, 1046.1000000, 230.4930000, 33.3218000, 20.8105000, 12.9975000, 4.8305100, 3.0155200, 1.3706200, 0.7358360, 0.3788160, 0.1335490, 0.0601560, 0.0269230, 0.0120000
c, 1.13, 0.0000090, 0.0000670, 0.0002740, 0.0082370, -0.0606330, 0.1778160, -0.4220620, -0.2693360, 0.5218430, 0.6380050, 0.2160910, 0.0112120, -0.0003020
c, 1.13, -0.0000060, -0.0000540, -0.0001390, -0.0077180, 0.0477700, -0.1303120, 0.3691480, 0.0873940, -0.3819580, -1.4316690, 1.1436500, 1.5516400, -1.0701580
c, 1.13, -0.0000110, -0.0000760, -0.0003310, -0.0077450, 0.0590240, -0.1882670, 0.5902840, 0.3330380, -2.0874360, -0.6503990, 3.7087420, -2.6700620, 0.0200220
c, 1.13, -0.0000030, -0.0000220, -0.0000930, -0.0024160, 0.0183200, -0.0560010, 0.1511880, 0.0755970, -0.2027390, -0.3248550, -0.1823170, 0.3372970, 0.6378330
c, 1.13, -0.0000160, -0.0001130, -0.0005110, -0.0109830, 0.0864930, -0.2861790, 1.0012380, 0.7225520, -7.6503270, 10.7993970, -5.5917980, -0.4226910, 2.7545010
c, 14.14, 1.0000000
c, 15.15, 1.0000000
p, TC , 165.0311000, 10.7957000, 6.7484100, 3.4824900, 1.9185300, 1.0018800, 0.5221550, 0.2608820, 0.1212740, 0.0554020, 0.0248480, 0.0111000
c, 1.10, 0.0000940, 0.0509640, -0.1500490, -0.1153530, 0.2970100, 0.4901870, 0.3265400, 0.0873840, 0.0060650, 0.0011260
c, 1.10, -0.0000510, -0.0254430, 0.0778710, 0.0573030, -0.1772360, -0.3257380, -0.1883490, 0.3694040, 0.6276010, 0.2078840
c, 1.10, -0.0002000, -0.0617730, 0.1884450, 0.4209310, -1.6231020, -0.2356560, 2.8630840, -2.0324030, -0.4381220, 0.9624810
c, 1.10, -0.0000970, -0.0448590, 0.1386420, 0.1301660, -0.4343680, -0.7982230, 0.5413080, 1.2254840, -0.5919300, -0.5926290
c, 1.10, -0.0000310, -0.0155010, 0.0475240, 0.0326450, -0.1022890, -0.1840740, -0.1346980, 0.1036920, 0.4224280, 0.4828080
c, 11.11, 1.0000000
c, 12.12, 1.0000000
d, TC , 71.1813000, 19.5319000, 7.2374300, 2.6113400, 1.4773400, 0.7880050, 0.4049350, 0.2003640, 0.0949110, 0.0419450, 0.0185000
c, 1.9, 0.0001750, 0.0017220, -0.0138320, 0.0740660, 0.2185740, 0.3134600, 0.3079090, 0.2213540, 0.1051100
c, 1.9, 0.0003950, 0.0025090, -0.0230280, 0.1928110, 0.5425200, 0.1303490, -0.7811880, -0.3613460, 0.5512450
c, 1.9, -0.0002340, -0.0055190, 0.0446410, -0.3882320, -0.8042540, 1.1596620, 0.5374260, -1.3024870, 0.1537280
c, 1.9, -0.0001940, -0.0017430, 0.0144030, -0.0844590, -0.2669030, -0.3360340, -0.0315050, 0.3870680, 0.4744620
c, 10.10, 1.0000000
c, 11.11, 1.0000000
f, TC , 3.362172564747E+00, 8.652268736217E-01, 2.790101483371E-01
c, 1.1, 1.000000E+00
c, 2.2, 1.000000E+00
c, 3.3, 1.000000E+00
g, TC , 1.013654610360E+00, 3.424364609442E-01
c, 1.1, 1.000000E+00
c, 2.2, 1.000000E+00

! ruthenium (15s,12p,11d,3f,2g) -> [7s,7p,6d,3f,2g]
s, RU , 7269.8900000, 1087.8400000, 241.7870000, 35.4525000, 22.1415000, 13.8292000, 5.1321300, 3.1705900, 1.4948700, 0.8005300, 0.4116380, 0.1424170, 0.0635630, 0.0282960, 0.0126000
c, 1.13, 0.0000110, 0.0000780, 0.0003110, 0.0087340, -0.0636040, 0.1857150, -0.4544900, -0.2563420, 0.5330450, 0.6399720, 0.2152730, 0.0112610, -0.0000950
c, 1.13, -0.0000070, -0.0000650, -0.0001740, -0.0083680, 0.0517850, -0.1417710, 0.4144270, 0.0784730, -0.4446880, -1.4550350, 1.2722320, 1.4072450, -0.9996440
c, 1.13, -0.0000120, -0.0000840, -0.0003620, -0.0076580, 0.0584390, -0.1862370, 0.5749660, 0.4015520, -2.1997860, -0.4355220, 3.4276070, -2.4454790, -0.1499430
c, 1.13, -0.0000040, -0.0000250, -0.0001060, -0.0024970, 0.0188420, -0.0575810, 0.1604580, 0.0691260, -0.2039790, -0.3204980, -0.1746560, 0.3326960, 0.6303210
c, 1.13, -0.0000160, -0.0001810, -0.0003320, -0.0254290, 0.1443970, -0.3836440, 1.3902860, 0.1271540, -6.8551520, 9.6961050, -4.7399940, -0.9214660, 3.0543570
c, 14.14, 1.0000000
c, 15.15, 1.0000000
p, RU , 174.3070000, 11.5040000, 7.1917600, 3.5749800, 2.1307800, 1.0892200, 0.5586370, 0.2704770, 0.1238960, 0.0558230, 0.0248420, 0.0111000
c, 1.10, 0.0001030, 0.0539940, -0.1610120, -0.1290380, 0.3147630, 0.5041410, 0.3252710, 0.0787020, 0.0037630, 0.0012860
c, 1.10, -0.0000610, -0.0287850, 0.0893050, 0.0707170, -0.2047470, -0.3715110, -0.1436350, 0.4622210, 0.5855810, 0.1507490
c, 1.10, -0.0002500, -0.0629920, 0.1916210, 0.6528290, -2.1533740, 0.4094420, 2.4570950, -2.2868480, 0.0777690, 0.7656040
c, 1.10, 0.0001120, 0.0492620, -0.1538200, -0.1731520, 0.5309750, 0.8176260, -0.7867320, -1.0474860, 0.7549080, 0.4632510
c, 1.10, -0.0000330, -0.0157130, 0.0487720, 0.0351930, -0.1032230, -0.1811150, -0.1243880, 0.1120280, 0.4225110, 0.4772310
c, 11.11, 1.0000000
c, 12.12, 1.0000000
d, RU , 75.3751000, 19.8901000, 7.8792400, 2.9083400, 1.6388300, 0.8757300, 0.4504830, 0.2227700, 0.1053420, 0.0464550, 0.0205000
c, 1.9, 0.0001800, 0.0020840, -0.0186710, 0.0780540, 0.2263280, 0.3171000, 0.3050360, 0.2151500, 0.0997580
c, 1.9, -0.0003550, -0.0031930, 0.0319560, -0.1855990, -0.5831850, -0.0727660, 0.7987070, 0.3016360, -0.5409680
c, 1.9, -0.0001820, -0.0072830, 0.0616630, -0.4441680, -0.7998390, 1.3061620, 0.3470880, -1.2223020, 0.1744000
c, 1.9, -0.0002030, -0.0020860, 0.0194280, -0.0871060, -0.2864130, -0.3319080, -0.0021210, 0.4023550, 0.4543320
c, 10.10, 1.0000000
c, 11.11, 1.0000000
f, RU , 3.936950913730E+00, 9.697281437014E-01, 3.003723829795E-01
c, 1.1, 1.000000E+00
c, 2.2, 1.000000E+00
c, 3.3, 1.000000E+00
g, RU , 1.117429159091E+00, 3.700240906487E-01
c, 1.1, 1.000000E+00
c, 2.2, 1.000000E+00

```
! rhodium             (15s,12p,11d,3f,2g) -> [7s,7p,6d,3f,2g]
s, RH , 7374.2200000, 1114.7200000, 249.9380000, 37.5315000, 23.4385000, 14.6379000, 5.5542100, 3.4224200, 1.6492800, 0.8832260, 0.4519700, 0.1530060, 0.0678490, 0.0299320,
        0.0132000
c, 1.13, 0.0000130, 0.0000920, 0.0003570, 0.0091740, -0.0666920, 0.1956740, -0.4574820, -0.2746970, 0.5224540, 0.6524920, 0.2257080, 0.0117950, -0.0001350
c, 1.13, -0.0000090, -0.0000760, -0.0002050, -0.0086430, 0.0537070, -0.1483390, 0.4122880, 0.1007430, -0.4427320, -1.4323570, 1.2199820, 1.3428560, -0.8361540
c, 1.13, -0.0000140, -0.0000950, -0.0003820, -0.0080940, 0.0598120, -0.1876680, 0.5255080, 0.4667230, -2.0232050, -0.6914780, 3.3320100, -1.9922090, -0.6067250
c, 1.13, -0.0000040, -0.0000290, -0.0001190, -0.0025760, 0.0194110, -0.0596780, 0.1589680, 0.0738260, -0.1968910, -0.3141660, -0.1768790, 0.3157110, 0.6247220
c, 1.13, -0.0000200, -0.0002080, -0.0003950, -0.0264950, 0.1507880, -0.4024970, 1.3689580, 0.2373890, -6.6015610, 8.6166140, -3.5310110, -1.7537080, 3.5259850
c, 14.14, 1.0000000
c, 15.15, 1.0000000
p, RH , 186.8620000, 12.3059000, 7.6935800, 3.7795600, 2.3232600, 1.1847300, 0.6092370, 0.2968270, 0.1372710, 0.0621260, 0.0276060, 0.0123000
c, 1.10, 0.0001090, 0.0560310, -0.1675940, -0.1363730, 0.3251810, 0.5055390, 0.3232040, 0.0788210, 0.0040650, 0.0012500
c, 1.10, -0.0000680, -0.0314700, 0.0979240, 0.0812320, -0.2287880, -0.4007930, -0.1097740, 0.4791740, 0.5581190, 0.1517420
c, 1.10, 0.0002540, 0.0713490, -0.2177380, -0.7639570, 2.4866360, -0.8314040, -2.1695430, 2.2490950, -0.1275420, -0.7284670
c, 1.10, 0.0001220, 0.0521280, -0.1624920, -0.2125080, 0.6239090, 0.7831600, -0.8575390, -0.9666410, 0.7421150, 0.4634940
c, 1.10, -0.0000350, -0.0167420, 0.0520850, 0.0385720, -0.1095340, -0.1882860, -0.1245600, 0.1171100, 0.4230670, 0.4736850
c, 11.11, 1.0000000
c, 12.12, 1.0000000
d, RH , 80.6204000, 22.1259000, 7.9583400, 3.1956900, 1.8113700, 0.9713830, 0.5017970, 0.2490680, 0.1179700, 0.0519500, 0.0229000
c, 1.9, 0.0002370, 0.0022370, -0.0176500, 0.0788370, 0.2317500, 0.3192230, 0.3020620, 0.2104350, 0.0966100
c, 1.9, -0.0002410, -0.0084470, 0.0698380, -0.5173290, -0.7709280, 1.4511960, 0.1698730, -1.1898870, 0.2468430
c, 1.9, -0.0004280, -0.0036360, 0.0308490, -0.1829010, -0.6144640, -0.0158290, 0.8110440, 0.2541130, -0.5519210
c, 1.9, -0.0002760, -0.0023470, 0.0190780, -0.0936260, -0.3085340, -0.3262390, 0.0310980, 0.4099490, 0.4368930
c, 10.10, 1.0000000
c, 11.11, 1.0000000
f, RH , 4.816482647744E+00, 1.149132215220E+00, 3.428704046638E-01
c, 1.1, 1.000000E+00
c, 2.2, 1.000000E+00
c, 3.3, 1.000000E+00
g, RH , 1.319805701007E+00, 3.838132823712E-01
c, 1.1, 1.000000E+00
c, 2.2, 1.000000E+00
```

```
! palladium           (15s,12p,11d,3f,2g) -> [7s,7p,6d,3f,2g]
s, PD , 7844.8900000, 1169.5300000, 259.6440000, 39.2211000, 24.4934000, 15.2969000, 6.0389600, 3.7622800, 1.8071000, 0.9743050, 0.4972030, 0.1620200, 0.0714600, 0.0313100,
         0.0137000
c, 1.13, 0.0000150, 0.0001070, 0.0004120, 0.0098640, -0.0719220, 0.2128330, -0.4542200, -0.2996210, 0.5046120, 0.6622770, 0.2406810, 0.0126460, -0.0004720
c, 1.13, -0.0000100, -0.0000840, -0.0002300, -0.0090740, 0.0563390, -0.1563130, 0.3943670, 0.1184910, -0.3911140, -1.3967980, 1.1011240, 1.3128540, -0.6576300
c, 1.13, -0.0000050, -0.0000330, -0.0001350, -0.0027160, 0.0205170, -0.0636890, 0.1551180, 0.0800520, -0.1865700, -0.3054990, -0.1813090, 0.3039740, 0.6177640
c, 1.13, 0.0000020, 0.0000150, 0.0000580, 0.0012900, -0.0092020, 0.0283700, -0.0690030, -0.0717590, 0.2549580, 0.1451930, -0.4647570, 0.2244370, 0.1291750
c, 1.13, -0.0000150, -0.0001090, -0.0004080, -0.0090780, 0.0648030, -0.1999130, 0.4866890, 0.5061480, -1.8058520, -1.0058890, 3.2670160, -1.5764190, -0.9382990
c, 14.14, 1.0000000
c, 15.15, 1.0000000
p, PD , 98.8243000, 27.9859000, 17.4873000, 6.7863800, 1.9878800, 1.0657300, 0.5838290, 0.3146360, 0.1357370, 0.0602770, 0.0265400, 0.0117000
c, 1.10, 0.0003180, -0.0046710, 0.0292410, -0.1992820, 0.4101390, 0.4471440, 0.2362210, 0.0624690, 0.0060750, -0.0000850
c, 1.10, -0.0001660, 0.0022660, -0.0161180, 0.1219530, -0.3403790, -0.3281810, -0.0330520, 0.5487950, 0.5332220, 0.0994340
c, 1.10, 0.0000090, -0.0013240, 0.0372040, -0.3882460, 2.6384130, -2.7457290, -0.6384020, 1.9045610, -0.1954810, -0.6798620
c, 1.10, 0.0004190, -0.0058840, 0.0317000, -0.2240400, 0.8656960, 0.3452800, -0.9931720, -0.6775520, 0.7731840, 0.3765310
c, 1.10, -0.0000510, 0.0006780, -0.0066790, 0.0544150, -0.1382020, -0.1410000, -0.1010710, 0.1265930, 0.4211200, 0.4722370
c, 11.11, 1.0000000
c, 12.12, 1.0000000
d, PD , 78.3406000, 21.0235000, 7.5187300, 3.6893300, 2.0193400, 1.0717600, 0.5516620, 0.2739330, 0.1301110, 0.0574730, 0.0254000
c, 1.9, 0.0003190, 0.0031690, -0.0301280, 0.0820580, 0.2405330, 0.3254140, 0.3009480, 0.2050090, 0.0921770
c, 1.9, -0.0004130, -0.0116470, 0.1255530, -0.5684920, -0.7972550, 1.5702270, 0.0164980, -1.1631360, 0.3227120
c, 1.9, -0.0005710, -0.0049650, 0.0523140, -0.1757260, -0.6558300, 0.0297190, 0.8321610, 0.1960240, -0.5651970
c, 1.9, -0.0003900, -0.0033600, 0.0334560, -0.0998880, -0.3344540, -0.3267360, 0.0663700, 0.4205140, 0.4170070
c, 10.10, 1.0000000
c, 11.11, 1.0000000
f, PD , 5.756254671585E+00, 1.329841862133E+00, 3.930610409805E-01
c, 1.1, 1.000000E+00
c, 2.2, 1.000000E+00
c, 3.3, 1.000000E+00
g, PD , 1.476318289219E+00, 5.510106588447E-01
c, 1.1, 1.000000E+00
c, 2.2, 1.000000E+00
```

```
! silver              (15s,12p,11d,3f,2g) -> [7s,7p,6d,3f,2g]
s, AG , 8123.1400000, 1213.3500000, 268.3390000, 37.7949000, 23.5569000, 14.6887000, 6.8550100, 4.1501000, 1.9670800, 1.0555800, 0.5356910, 0.1714340, 0.0751230, 0.0327140,
        0.0142000
c, 1.14, 0.0000150, 0.0001100, 0.0004200, 0.0131810, -0.1045750, 0.3282440, -0.5360540, -0.3312650, 0.5220930, 0.6769540, 0.2298530, 0.0099980, 0.0000040, 0.0012490
c, 1.14, -0.0000050, -0.0000320, -0.0001350, -0.0033140, 0.0279050, -0.0929070, 0.1697180, 0.0874880, -0.1802730, -0.2987180, -0.1766820, 0.2949860, 0.6098960, 0.2873860
c, 1.14, -0.0000090, -0.0000750, -0.0002150, -0.0098780, 0.0689300, -0.2095320, 0.4003210, 0.1121400, -0.3203310, -1.2009440, 0.7920340, 1.3257180, -0.3968510, -0.8604900
c, 1.14, -0.0000160, -0.0001110, -0.0004430, -0.0111380, 0.0911750, -0.3069920, 0.5628120, 0.5281440, -1.6646220, -1.2981790, 3.2811190, -1.1821200, -1.2688480, 1.3981890
c, 1.14, -0.0000260, -0.0001440, -0.0008240, -0.0083010, 0.1039930, -0.4235890, 0.8799960, 1.1771790, -6.5454940, 6.9751850, -1.6693400, -2.9472570, 4.1809470, -2.0578000
c, 14.14, 1.0000000
c, 15.15, 1.0000000
p, AG , 99.5339000, 24.3926000, 15.2691000, 7.3895000, 2.1891400, 1.1641300, 0.6136600, 0.3066080, 0.1363460, 0.0609370, 0.0269680, 0.0119000
c, 1.11, 0.0002930, -0.0086430, 0.0626310, -0.2546790, 0.4153370, 0.4712950, 0.2399810, 0.0459130, 0.0026410, 0.0004260, -0.0000360
c, 1.11, -0.0001390, 0.0039750, -0.0321780, 0.1419020, -0.3013970, -0.3496070, 0.0078870, 0.5448030, 0.5057240, 0.0982270, 0.0051900
c, 1.11, -0.0000650, -0.0044780, 0.0834410, -0.4753380, 2.5592720, -2.4904940, -0.6965260, 1.9699180, -0.4364670, -0.5794010, 0.0184220
c, 1.11, -0.0003560, 0.0094120, -0.0644850, 0.2789050, -0.8354180, -0.4395730, 1.1479170, 0.5663380, -0.8183880, -0.3034240, -0.0039640
c, 1.11, -0.0000460, 0.0014650, -0.0143630, 0.0661260, -0.1305340, -0.1506930, -0.0888000, 0.1440160, 0.4270630, 0.4565880, 0.1478750
c, 11.11, 1.0000000
c, 12.12, 1.0000000
d, AG , 94.2866000, 26.0504000, 8.5332100, 4.1393000, 2.3676000, 1.2934300, 0.6854690, 0.3511500, 0.1722770, 0.0788950, 0.0361000
c, 1.10, 0.0002750, 0.0024600, -0.0240320, 0.0638430, 0.2247680, 0.3195440, 0.3060530, 0.2131570, 0.0957390, 0.0173080
c, 1.10, -0.0003860, -0.0030490, 0.0312650, -0.0929530, -0.3825950, -0.3670700, 0.1100820, 0.4598890, 0.3715490, 0.0993590
c, 1.10, -0.0004640, -0.0043970, 0.0475130, -0.1523550, -0.7265610, 0.0858510, 0.9312950, 0.0992390, -0.6326340, -0.2791080
c, 1.10, -0.0002070, -0.0112610, 0.1297190, -0.6288980, -0.8758070, 1.8842660, -0.2016960, -1.2470160, 0.5101280, 0.4504590
c, 10.10, 1.0000000
c, 11.11, 1.0000000
f, AG , 6.701468800184E+00, 1.544080719176E+00, 4.643976356672E-01
c, 1.1, 1.000000E+00
c, 2.2, 1.000000E+00
c, 3.3, 1.000000E+00
g, AG , 2.087468833189E+00, 5.871107062209E-01
c, 1.1, 1.000000E+00
c, 2.2, 1.000000E+00
```

```
! cadmium              (15s,12p,11d,3f,2g) -> [7s,7p,6d,3f,2g]
s, CD , 8793.0000000, 1304.3500000, 286.9300000, 40.9458000, 25.4342000, 15.8264000, 7.0915300, 4.4135500, 1.9778500, 1.0595900, 0.5428770, 0.1790010, 0.0775800, 0.0336320,
        0.0146000
c, 1.14, 0.0000180, 0.0001320, 0.0005050, 0.0135020, -0.1022910, 0.3144790, -0.5633230, -0.2766280, 0.5888700, 0.6409640, 0.1862320, 0.0058780, -0.0011090, 0.0002590
c, 1.14, -0.0000250, -0.0005180, 0.0001440, -0.1004100, 0.5137630, -1.2429870, 3.4112760, -2.1883730, -6.1974000, 11.4249990, -7.5722620, 2.0448040, 0.4634670, -0.8954360
c, 1.14, 0.0000140, 0.0001180, 0.0003700, 0.0129780, -0.0880930, 0.2653300, -0.5610540, -0.1382420, 0.7673490, 1.3726410, -1.6006730, -1.0887970, 1.0119630, 0.2952320
c, 1.14, -0.0000060, -0.0000390, -0.0001620, -0.0035000, 0.0277310, -0.0900690, 0.1821160, 0.0677900, -0.2051570, -0.3064560, -0.1475430, 0.3470450, 0.6136400, 0.2377100
c, 1.14, 0.0000220, 0.0001240, 0.0007350, 0.0049080, -0.0671910, 0.2753480, -0.5469830, -0.7971070, 3.3339810, -1.1322300, -2.6764780, 3.0276260, -0.9754520, -0.5270070
c, 14.14, 1.0000000
c, 15.15, 1.0000000
p, CD , 120.1895000, 27.9530000, 17.5012000, 7.8216000, 2.3231500, 1.2310900, 0.6486230, 0.3251940, 0.1425240, 0.0630880, 0.0276800, 0.0121000
c, 1.11, 0.0002880, -0.0060500, 0.0453130, -0.2309620, 0.4229230, 0.4720020, 0.2302990, 0.0414230, 0.0024440, 0.0003630, -0.0000220
c, 1.11, -0.0001300, 0.0025440, -0.0212710, 0.1174690, -0.2756440, -0.3062620, -0.0323190, 0.5011630, 0.5490480, 0.1145660, 0.0050730
c, 1.11, -0.0003210, 0.0066800, -0.0465340, 0.2512720, -0.8207010, -0.4365830, 1.0883990, 0.6322200, -0.8039930, -0.3005600, -0.0016920
c, 1.11, -0.0003640, 0.0052480, 0.0417290, -0.4067060, 2.4451880, -2.3362140, -0.8066910, 1.9782120, -0.3944950, -0.5958600, 0.0276980
c, 1.11, -0.0000460, 0.0008440, -0.0098170, 0.0582890, -0.1301770, -0.1415740, -0.0857170, 0.1398650, 0.4210830, 0.4603340, 0.1545780
c, 11.11, 1.0000000
c, 12.12, 1.0000000
d, CD , 105.3300000, 29.3090000, 9.5323900, 4.7796100, 2.6613300, 1.4457400, 0.7683300, 0.3985390, 0.1999000, 0.0942680, 0.0445000
c, 1.10, 0.0002970, 0.0025980, -0.0221920, 0.0592660, 0.2292930, 0.3280280, 0.3078190, 0.2047850, 0.0855270, 0.0141120
c, 1.10, -0.0004580, -0.0035730, 0.0319050, -0.0955770, -0.4517700, -0.3712080, 0.2107970, 0.4842170, 0.3019970, 0.0589150
c, 1.10, -0.0004410, -0.0049140, 0.0469010, -0.1432230, -0.7734170, 0.2242430, 0.9456240, -0.0343390, -0.6751870, -0.2304630
c, 1.10, 0.0001290, 0.0120660, -0.1289060, 0.5781290, 0.9086400, -1.9362210, 0.2848470, 1.3005940, -0.6077290, -0.4564420
c, 10.10, 1.0000000
c, 11.11, 1.0000000
f, CD , 7.615352310755E+00, 1.768927363181E+00, 5.303102238965E-01
c, 1.1, 1.000000E+00
c, 2.2, 1.000000E+00
c, 3.3, 1.000000E+00
g, CD , 2.470984379569E+00, 6.483059388428E-01
c, 1.1, 1.000000E+00
c, 2.2, 1.000000E+00
```

```
! hafnium              (15s,12p,11d,3f,2g) -> [7s,7p,6d,3f,2g]
s, HF , 72.4389000, 45.2162000, 28.2220000, 17.6385000, 11.0226000, 6.8849000, 3.5401300, 1.9114300, 1.1108400, 0.6210720, 0.3317360, 0.1185800, 0.0548520, 0.0251920, 0.0116000
c, 1.13, 0.0011630, -0.0114520, 0.0739620, -0.3243560, 0.7373860, -0.4920420, -0.5503310, 0.0068040, 0.5487620, 0.5890330, 0.2060050, 0.0088010, -0.0030800
c, 1.13, 0.0012180, -0.0046350, -0.0055870, 0.0992940, -0.2810020, 0.1448310, 0.4847140, -0.2888910, 0.0737130, -1.6459660, 0.9783500, 1.9238700, -1.2965760
c, 1.13, 0.0016570, -0.0059790, -0.0118030, 0.1606530, -0.4541850, 0.2557780, 0.7460350, -0.2399350, -0.8580200, -2.5354000, 4.9533640, -2.7189210, -0.3507780
c, 1.13, -0.0002440, 0.0027980, -0.0202250, 0.0943500, -0.2219970, 0.1514670, 0.1831980, -0.0037340, -0.2019920, -0.3343960, -0.1997340, 0.3916460, 0.6450710
c, 1.13, -0.0189520, 0.1126140, -0.3791580, 0.9695960, -1.8053410, 1.7993640, -1.2136050, 4.3487530, -10.5038250, 9.8551680, -2.5265540, -3.6117060, 5.3189020
c, 14.14, 1.0000000
c, 15.15, 1.0000000
p, HF , 27.5515000, 17.2296000, 10.7808000, 4.8379500, 1.1914000, 0.6007080, 0.3074110, 0.1562270, 0.0760330, 0.0373420, 0.0181300, 0.0088000
c, 1.10, 0.0043130, -0.0366020, 0.1160700, -0.2926280, 0.4420380, 0.4780660, 0.2122140, 0.0327530, 0.0048070, -0.0002130
c, 1.10, -0.0014480, 0.0136000, -0.0454180, 0.1200980, -0.2032580, -0.2941450, -0.1276190, 0.5058440, 0.5783360, 0.1178420
c, 1.10, -0.0032460, 0.0272840, -0.0875310, 0.2316860, -0.4719300, -0.8206520, 1.1440980, 0.9145160, -0.9777530, -0.3066250
c, 1.10, 0.0093880, -0.0585180, 0.1606140, -0.4095390, 1.5183920, -0.4586730, -2.6576990, 3.0602870, -0.6219400, -0.6289150
c, 1.10, -0.0010340, 0.0094990, -0.0314490, 0.0827700, -0.1400300, -0.1925740, -0.0865620, 0.2069060, 0.4684290, 0.3988560
c, 11.11, 1.0000000
c, 12.12, 1.0000000
d, HF , 47.2322000, 13.1047000, 7.5050100, 4.5180600, 1.2669600, 0.6271160, 0.3015600, 0.1411600, 0.0642070, 0.0277910, 0.0120000
c, 1.9, 0.0000170, -0.0008770, 0.0155100, -0.0573800, 0.1453560, 0.2843090, 0.3476220, 0.3054930, 0.1632340
c, 1.9, -0.0000330, -0.0003780, 0.0220450, -0.0947120, 0.3497240, 0.6024320, -0.5248690, -0.7984990, 0.6042640
c, 1.9, 0.0006970, -0.0110350, 0.0668960, -0.1839770, 0.8013200, 0.2430220, -1.8839990, 1.6425780, -0.0995440
c, 1.9, 0.0000070, 0.0005750, -0.0165460, 0.0660740, -0.1879000, -0.3975450, -0.2773590, 0.3362230, 0.5471920
c, 10.10, 1.0000000
c, 11.11, 1.0000000
f, HF , 6.884937465475E-01, 2.446245276051E-01, 8.822669282177E-02
c, 1.1, 1.000000E+00
c, 2.2, 1.000000E+00
c, 3.3, 1.000000E+00
g, HF , 5.473939586746E-01, 1.857376729053E-01
c, 1.1, 1.000000E+00
c, 2.2, 1.000000E+00
```

```
! tantalum            (15s,12p,11d,3f,2g) -> [7s,7p,6d,3f,2g]
s, TA , 73.7813000, 46.0585000, 28.7729000, 17.9697000, 11.2263000, 7.0033200, 3.6334100, 1.9104700, 1.1325100, 0.6371150, 0.3430120, 0.1304260, 0.0597240, 0.0272230, 0.0124000
c, 1.13, 0.0010840, -0.0106880, 0.0702380, -0.3188000, 0.7504170, -0.5237200, -0.5605690, 0.0417880, 0.5891840, 0.5664000, 0.1788110, 0.0069420, -0.0034680
c, 1.13, 0.0009880, -0.0033780, -0.0096380, 0.1133850, -0.3230420, 0.1973730, 0.4721130, -0.2411030, -0.1267900, -1.5906280, 1.1589900, 1.7377590, -1.1917780
c, 1.13, -0.0002130, 0.0025590, -0.0193980, 0.0949430, -0.2327050, 0.1669980, 0.1927810, -0.0150810, -0.2349860, -0.3487000, -0.1647020, 0.4033670, 0.6386680
c, 1.13, 0.0009140, -0.0018790, -0.0242770, 0.1948080, -0.5400090, 0.3650020, 0.7127130, -0.1537290, -1.5167170, -1.5267620, 4.5523910, -2.7308240, -0.4295940
c, 1.13, -0.0137090, 0.0819450, -0.2819230, 0.7594780, -1.4786470, 1.4265190, -0.5641730, 3.4165950, -10.1513490, 10.4775230, -3.1726800, -3.3629210, 5.1320540
c, 14.14, 1.0000000
c, 15.15, 1.0000000
p, TA , 27.9136000, 17.4530000, 10.9180000, 5.0300800, 1.2717500, 0.6519410, 0.3393560, 0.1775360, 0.0863880, 0.0416460, 0.0197310, 0.0093000
c, 1.10, 0.0040610, -0.0358630, 0.1199340, -0.3036450, 0.4445670, 0.4750100, 0.2119180, 0.0361900, 0.0054550, -0.0001940
c, 1.10, -0.0014500, 0.0143690, -0.0509460, 0.1359460, -0.2282980, -0.3233980, -0.1091860, 0.5313070, 0.5690130, 0.1056730
c, 1.10, 0.0077180, -0.0556400, 0.1747500, -0.4767370, 1.9284790, -1.1687440, -2.2422620, 3.0810600, -0.7866630, -0.5436600
c, 1.10, -0.0034200, 0.0290570, -0.0975450, 0.2603850, -0.5643600, -0.7742520, 1.2323600, 0.7955210, -0.9257990, -0.3360500
c, 1.10, -0.0009880, 0.0096120, -0.0338360, 0.0898790, -0.1496740, -0.2007630, -0.0819650, 0.2001060, 0.4661240, 0.4100630
c, 11.11, 1.0000000
c, 12.12, 1.0000000
d, TA , 47.2399000, 13.1212000, 7.9410300, 4.5789100, 1.3728600, 0.7006000, 0.3472210, 0.1669860, 0.0775680, 0.0340580, 0.0150000
c, 1.9, 0.0000260, -0.0014810, 0.0178090, -0.0690840, 0.1596480, 0.2981540, 0.3480990, 0.2867490, 0.1438900
c, 1.9, -0.0000610, -0.0004510, 0.0240590, -0.1166800, 0.4375110, 0.5807630, -0.6903200, -0.6825310, 0.6453860
c, 1.9, 0.0006040, -0.0113380, 0.0659210, -0.2133010, 1.0202930, -0.1151460, -1.7382210, 1.7498200, -0.2073400
c, 1.9, 0.0000050, 0.0010590, -0.0188100, 0.0800010, -0.2138050, -0.4252560, -0.2277030, 0.3960240, 0.5188780
c, 10.10, 1.0000000
c, 11.11, 1.0000000
f, TA , 7.053424142581E-01, 2.403809238746E-01, 5.299074190948E-02
c, 1.1, 1.000000E+00
c, 2.2, 1.000000E+00
c, 3.3, 1.000000E+00
g, TA , 8.064918815951E-01, 2.688437843065E-01
c, 1.1, 1.000000E+00
c, 2.2, 1.000000E+00
```

```
! tungsten             (15s,12p,11d,3f,2g) -> [7s,7p,6d,3f,2g]
s, W , 77.1195000, 48.1958000, 30.1187000, 18.7768000, 11.7358000, 7.2788700, 3.8670800, 1.9127300, 1.1804400, 0.6475100, 0.3432030, 0.1412450, 0.0640210, 0.0289530, 0.0131000
c, 1.13, 0.0009710, -0.0094870, 0.0630910, -0.2947690, 0.7008950, -0.4703580, -0.5853380, 0.0318840, 0.6455090, 0.5593650, 0.1432700, 0.0040440, -0.0035460
c, 1.13, -0.0006030, 0.0013840, 0.0144160, -0.1211050, 0.3338390, -0.2077230, -0.4467040, 0.1281090, 0.4202140, 1.3598420, -1.2237640, -1.5559960, 1.0824180
c, 1.13, -0.0001850, 0.0022560, -0.0176440, 0.0896450, -0.2227610, 0.1544930, 0.2057680, -0.0119820, -0.2742450, -0.3627550, -0.1249040, 0.4138630, 0.6326800
c, 1.13, -0.0000710, 0.0034200, -0.0385710, 0.2232260, -0.5873970, 0.4160230, 0.6715970, -0.0045440, -2.1351460, -0.4472160, 4.0371680, -2.7972560, -0.4402180
c, 1.13, -0.0090760, 0.0545770, -0.1942180, 0.5635980, -1.1452700, 1.0220890, 0.0759150, 2.4385620, -9.0879040, 10.2451000, -3.7254950, -2.9267180, 4.8592420
c, 14.14, 1.0000000
c, 15.15, 1.0000000
p, W , 29.7849000, 18.6260000, 11.6544000, 5.3065700, 1.3322000, 0.6793550, 0.3475220, 0.1736150, 0.0847170, 0.0414500, 0.0200010, 0.0097000
c, 1.10, 0.0042390, -0.0368190, 0.1206080, -0.3068540, 0.4655600, 0.4774170, 0.1982390, 0.0280040, 0.0038430, -0.0002560
c, 1.10, -0.0016220, 0.0157240, -0.0545450, 0.1466050, -0.2607220, -0.3620910, -0.0073890, 0.6264040, 0.4630080, 0.0634300
c, 1.10, 0.0053940, -0.0480700, 0.1663880, -0.4878230, 2.1901880, -1.9515560, -1.2594270, 2.8022120, -1.1616660, -0.3249580
c, 1.10, -0.0039160, 0.0317850, -0.1032130, 0.2769090, -0.6727660, -0.6716600, 1.4911520, 0.4168600, -0.9480850, -0.2160280
c, 1.10, -0.0010730, 0.0101370, -0.0347900, 0.0928310, -0.1624000, -0.2088740, -0.0538850, 0.2474960, 0.4681480, 0.3682370
c, 11.11, 1.0000000
c, 12.12, 1.0000000
d, W , 47.6003000, 14.5420000, 9.0813200, 5.0997400, 1.4962900, 0.7689810, 0.3842550, 0.1856380, 0.0862530, 0.0375810, 0.0164000
c, 1.9, 0.0000330, -0.0025530, 0.0218860, -0.0784400, 0.1687640, 0.3145610, 0.3535110, 0.2727040, 0.1226270
c, 1.9, 0.0005810, -0.0123990, 0.0712450, -0.2416100, 1.2819070, -0.6743030, -1.3615990, 1.9042820, -0.5902650
c, 2.9, -0.0032460, 0.0353940, -0.1435400, 0.5278820, 0.5600390, -0.9431490, -0.4525680, 0.7364580
c, 1.9, 0.0000080, 0.0022640, -0.0243420, 0.0948420, -0.2450010, -0.4648380, -0.1570150, 0.4777850, 0.4723200
c, 10.10, 1.0000000
c, 11.11, 1.0000000
f, W , 9.653837851165E-01, 3.750161750929E-01, 1.405659072511E-01
c, 1.1, 1.000000E+00
c, 2.2, 1.000000E+00
c, 3.3, 1.000000E+00
g, W , 7.825282050666E-01, 2.593044679785E-01
c, 1.1, 1.000000E+00
c, 2.2, 1.000000E+00
```

! rhenium (15s,12p,11d,3f,2g) -> [7s,7p,6d,3f,2g]
s, RE , 82.3602000, 51.4686000, 32.1603000, 20.0660000, 12.4622000, 7.5180000, 4.1506800, 1.9643400, 1.2270000, 0.6571530, 0.3387020, 0.1441200, 0.0649710, 0.0295140, 0.0134000
c, 1.13, 0.0006360, -0.0064370, 0.0462420, -0.2274960, 0.5498740, -0.3203770, -0.6378710, 0.0269990, 0.6930860, 0.5435260, 0.1155350, 0.0046460, 0.0007070
c, 1.13, -0.0004390, 0.0009780, 0.0120630, -0.1013780, 0.2812560, -0.1516170, -0.4639690, 0.0623710, 0.6503050, 1.1453480, -1.3314350, -1.3425100, 1.0609040
c, 1.13, -0.0009080, 0.0074070, -0.0459360, 0.2203600, -0.5556240, 0.3751760, 0.7129580, 0.1590340, -2.9451850, 0.8920090, 3.3695610, -3.1755850, 0.1060080
c, 1.13, -0.0001250, 0.0016030, -0.0136440, 0.0728800, -0.1841680, 0.1126340, 0.2319940, -0.0147960, -0.3107090, -0.3716290, -0.0795990, 0.4391580, 0.6122060
c, 1.13, -0.0021520, 0.0143330, -0.0679810, 0.2773630, -0.6550320, 0.3786980, 1.0665680, 0.6140170, -7.8614560, 11.7297390, -6.9458570, 0.0308980, 3.0805360
c, 14.14, 1.0000000
c, 15.15, 1.0000000
p, RE , 32.4690000, 20.2970000, 12.6941000, 5.2869700, 1.4202400, 0.7343010, 0.3810460, 0.1971460, 0.0952250, 0.0459080, 0.0217670, 0.0103000
c, 1.10, 0.0029450, -0.0256880, 0.0857520, -0.2824710, 0.4641420, 0.4730530, 0.2020920, 0.0319370, 0.0048080, -0.0002900
c, 1.10, -0.0010490, 0.0110530, -0.0402980, 0.1433430, -0.2853640, -0.3650760, -0.0032000, 0.6099320, 0.4790080, 0.0693610
c, 1.10, -0.0030230, 0.0239340, -0.0783600, 0.2752990, -0.7659500, -0.5943630, 1.4982640, 0.3730740, -0.8983170, -0.2479210
c, 1.10, 0.0013180, -0.0252900, 0.1097100, -0.4629510, 2.3397460, -2.2266480, -1.1081910, 2.6957210, -0.9828420, -0.4244410
c, 1.10, -0.0006380, 0.0067070, -0.0244150, 0.0864310, -0.1669190, -0.2014050, -0.0604410, 0.2224410, 0.4576740, 0.3927610
c, 11.11, 1.0000000
c, 12.12, 1.0000000
d, RE , 47.7382000, 15.0360000, 9.3875900, 5.1609800, 1.6027500, 0.8343150, 0.4207260, 0.2047550, 0.0957060, 0.0419030, 0.0183000
c, 1.9, 0.0000420, -0.0012520, 0.0144400, -0.0676120, 0.1717950, 0.3138640, 0.3428700, 0.2655630, 0.1355580
c, 1.9, 0.0009540, -0.0145770, 0.0668650, -0.2302910, 1.2509670, -0.5515880, -1.4396770, 1.7125070, -0.2654990
c, 1.9, -0.0000670, 0.0003250, 0.0181980, -0.1178440, 0.5306130, 0.5052540, -0.7784010, -0.5695280, 0.6333480
c, 1.9, -0.0000470, 0.0013030, -0.0157960, 0.0766030, -0.2203650, -0.4468310, -0.1480700, 0.4277470, 0.4973000
c, 10.10, 1.0000000
c, 11.11, 1.0000000
f, RE , 1.071173943893E+00, 4.189768236878E-01, 1.595732168104E-01
c, 1.1, 1.000000E+00
c, 2.2, 1.000000E+00
c, 3.3, 1.000000E+00
g, RE , 9.176984538498E-01, 3.161549602652E-01
c, 1.1, 1.000000E+00
c, 2.2, 1.000000E+00

! osmium (15s,12p,11d,3f,2g) -> [7s,7p,6d,3f,2g]
s, OS , 88.2006000, 55.1180000, 34.4482000, 21.5217000, 13.4044000, 7.0802200, 4.3353500, 2.6646000, 1.3201000, 0.7030860, 0.3585070, 0.1567570, 0.0704870, 0.0318370, 0.0144000
c, 1.13, 0.0006090, -0.0057630, 0.0399770, -0.1928220, 0.4375150, -0.2402670, -0.6446130, 0.0080600, 0.7119800, 0.5543000, 0.1138550, 0.0044490, 0.0013710
c, 1.13, -0.0001790, -0.0003850, 0.0154440, -0.1044350, 0.2649760, -0.1403670, -0.5192940, 0.0234310, 0.8927930, 1.0766050, -1.5497940, -1.1321140, 1.0563670
c, 1.13, 0.0013940, -0.0098010, 0.0506940, -0.2173830, 0.4993420, -0.3676680, -0.6773930, -0.3262780, 3.7857020, -2.2376600, -2.6737740, 3.4798650, -0.6550940
c, 1.13, -0.0001370, 0.0015220, -0.0121600, 0.0632540, -0.1499420, 0.0892650, 0.2391400, -0.0095340, -0.3288700, -0.3731540, -0.0749070, 0.4235470, 0.6120600
c, 1.13, 0.0119650, -0.0648350, 0.1667400, -0.2028200, 0.0740870, -0.9957190, 3.8484290, -2.7108190, -5.9258730, 12.4114900, -9.7779040, 3.1563620, 0.9797310
c, 14.14, 1.0000000
c, 15.15, 1.0000000
p, OS , 34.4538000, 21.5352000, 13.4667000, 5.4212400, 1.5072000, 0.7852120, 0.4101120, 0.2131350, 0.1007730, 0.0475860, 0.0221800, 0.0103000
c, 1.10, 0.0026020, -0.0226770, 0.0765000, -0.2805070, 0.4691690, 0.4726960, 0.1998810, 0.0328220, 0.0045210, -0.0002090
c, 1.10, -0.0009960, 0.0105380, -0.0390140, 0.1558430, -0.3274530, -0.3897860, 0.0552590, 0.6453450, 0.4392330, 0.0458690
c, 1.10, -0.0034350, 0.0245630, -0.0772030, 0.2984360, -0.9059580, -0.4693980, 1.6277740, 0.1275790, -0.8670940, -0.1952430
c, 1.10, -0.0014480, -0.0135590, 0.0892570, -0.4749820, 2.6052390, -2.8769040, -0.4307110, 2.4141580, -1.0494770, -0.3535840
c, 1.10, -0.0004860, 0.0055470, -0.0210020, 0.0840370, -0.1672940, -0.1910410, -0.0601410, 0.2088230, 0.4505520, 0.4058320
c, 11.11, 1.0000000
c, 12.12, 1.0000000
d, OS , 46.1455000, 17.4458000, 10.8908000, 5.4010400, 1.7049400, 0.8973250, 0.4561300, 0.2232570, 0.1048330, 0.0461230, 0.0203000
c, 1.9, 0.0000690, -0.0010520, 0.0100340, -0.0624020, 0.1801220, 0.3211200, 0.3405410, 0.2566720, 0.1277410
c, 1.9, 0.0009660, -0.0105910, 0.0462480, -0.2268280, 1.6025830, -1.3450410, -0.7878900, 1.6682930, -0.5570430
c, 1.9, 0.0002460, -0.0027010, 0.0198360, -0.1263280, 0.6377010, 0.4486600, -1.0127690, -0.3275190, 0.6835000
c, 1.9, -0.0001310, 0.0016440, -0.0126990, 0.0767760, -0.2526490, -0.4906830, -0.0789280, 0.4814540, 0.4524130
c, 10.10, 1.0000000
c, 11.11, 1.0000000
f, OS , 1.221349615442E+00, 4.835261872827E-01, 1.833918366733E-01
c, 1.1, 1.000000E+00
c, 2.2, 1.000000E+00
c, 3.3, 1.000000E+00
g, OS , 9.945806371983E-01, 3.324834252428E-01
c, 1.1, 1.000000E+00
c, 2.2, 1.000000E+00

```
! iridium             (15s,12p,11d,3f,2g) -> [7s,7p,6d,3f,2g]
s, IR , 99.3982000, 59.7348000, 37.3064000, 23.3112000, 13.4959000, 7.6049800, 4.5313300, 2.3087100, 1.3601100, 0.7322980, 0.3813520, 0.1640300, 0.0737290, 0.0331980, 0.0149000
c, 1.13, 0.0004580, -0.0046060, 0.0316100, -0.1428720, 0.3942810, -0.2127580, -0.6912870, 0.0445860, 0.7267130, 0.5268050, 0.1082530, 0.0055800, 0.0013060
c, 1.13, 0.0002210, -0.0001990, -0.0113270, 0.0794760, -0.2534940, 0.1289840, 0.5909610, -0.0593750, -1.0626430, -0.9731780, 1.7188600, 0.9367500, -1.0082480
c, 1.13, -0.0005110, 0.0015380, 0.0130200, -0.1133550, 0.3795890, -0.1479200, -1.1631820, 0.1491150, 4.0963770, -3.3812610, -1.6774500, 3.1755010, -0.7454630
c, 1.13, -0.0001200, 0.0013160, -0.0099630, 0.0478750, -0.1385630, 0.0830830, 0.2569740, -0.0292970, -0.3396010, -0.3604030, -0.0562520, 0.4218680, 0.6036200
c, 1.13, -0.0132150, 0.0783910, -0.2111880, 0.2858610, -0.2379870, 1.2800340, -4.3148610, 5.6632220, 1.5419410, -10.3480140, 9.5660340, -3.5876350, -0.5221100
c, 14.14, 1.0000000
c, 15.15, 1.0000000
p, IR , 36.4097000, 22.7558000, 14.2284000, 5.5960900, 1.5973000, 0.8378880, 0.4411290, 0.2344660, 0.1115150, 0.0528360, 0.0246160, 0.0115000
c, 1.10, 0.0023870, -0.0206380, 0.0702780, -0.2798050, 0.4739250, 0.4710650, 0.1978030, 0.0338300, 0.0051130, -0.0002720
c, 1.10, -0.0010530, 0.0108300, -0.0403920, 0.1766660, -0.3992470, -0.4077810, 0.1392320, 0.6411880, 0.4014080, 0.0408130
c, 1.10, -0.0039200, 0.0258350, -0.0785180, 0.3276760, -1.0985930, -0.2325000, 1.6725640, -0.0845670, -0.8015440, -0.1976930
c, 1.10, 0.0080660, -0.0114990, -0.0537570, 0.4715090, -2.8764400, 3.6250360, -0.3925860, -2.0037910, 0.9419720, 0.3970350
c, 1.10, -0.0004360, 0.0051630, -0.0200500, 0.0879310, -0.1789800, -0.1980510, -0.0603120, 0.2143880, 0.4546260, 0.4018720
c, 11.11, 1.0000000
c, 12.12, 1.0000000
d, IR , 52.9731000, 19.0521000, 11.8598000, 5.7186000, 1.8057500, 0.9571620, 0.4901660, 0.2416410, 0.1142040, 0.0505400, 0.0224000
c, 1.9, 0.0000690, -0.0008690, 0.0088410, -0.0614440, 0.1897440, 0.3281660, 0.3375200, 0.2474600, 0.1199030
c, 1.9, 0.0004330, -0.0044960, 0.0215990, -0.1214710, 0.6027800, 0.4503180, -0.8956850, -0.4187670, 0.5525300
c, 1.9, -0.0005010, 0.0057870, -0.0318090, 0.1983400, -1.5056470, 1.1365150, 0.9103540, -1.4285030, 0.1201680
c, 1.9, -0.0001380, 0.0015270, -0.0111590, 0.0729160, -0.2570590, -0.4663920, -0.0753040, 0.4160710, 0.4628000
c, 10.10, 1.0000000
c, 11.11, 1.0000000
f, IR , 9.644950431351E-01, 3.084608137757E-01, 7.463456398264E-02
c, 1.1, 1.000000E+00
c, 2.2, 1.000000E+00
c, 3.3, 1.000000E+00
g, IR , 1.102046703963E+00, 4.165911119924E-01
c, 1.1, 1.000000E+00
c, 2.2, 1.000000E+00
```

```
! platinum             (15s,12p,11d,3f,2g) -> [7s,7p,6d,3f,2g]
s, PT , 103.6120000, 64.6859000, 39.8400000, 24.9011000, 14.0033000, 7.8909000, 4.8456000, 2.3501200, 1.4671700, 0.7806710, 0.3946960, 0.1753230, 0.0788420, 0.0353890, 0.0159000
c, 1.13, 0.0005410, -0.0042820, 0.0279230, -0.1215420, 0.3554330, -0.1534930, -0.7258970, 0.0097520, 0.7419290, 0.5463830, 0.1046140, 0.0041520, 0.0020360
c, 1.13, -0.0000140, 0.0009880, -0.0135500, 0.0756570, -0.2479560, 0.1113150, 0.6080690, 0.0444240, -1.2579130, -0.8214190, 1.7271730, 0.7946170, -0.8962850
c, 1.13, -0.0005030, 0.0014690, 0.0108430, -0.0940950, 0.3378940, -0.0650820, -1.2245280, 0.1173930, 4.0554430, -3.3801850, -1.5351860, 2.9459960, -0.4434390
c, 1.13, -0.0001630, 0.0013240, -0.0091150, 0.0414900, -0.1272130, 0.0650280, 0.2695050, -0.0189050, -0.3462670, -0.3651790, -0.0511390, 0.4075980, 0.5982990
c, 1.13, -0.0140470, 0.0725050, -0.1892580, 0.2492270, -0.2112040, 1.3748360, -4.3141010, 6.2274430, 0.2464040, -8.7140170, 8.0961770, -2.6016490, -1.3052060
c, 14.14, 1.0000000
c, 15.15, 1.0000000
p, PT , 37.9380000, 23.7097000, 14.8236000, 5.7904600, 1.6955600, 0.8984940, 0.4791880, 0.2565920, 0.1170930, 0.0536550, 0.0243160, 0.0110000
c, 1.10, 0.0022880, -0.0197140, 0.0680900, -0.2834570, 0.4743470, 0.4685560, 0.1999420, 0.0374850, 0.0049230, -0.0002350
c, 1.10, -0.0011220, 0.0110810, -0.0414980, 0.1899730, -0.4366320, -0.4092230, 0.1550110, 0.6645690, 0.3881160, 0.0254450
c, 1.10, -0.0041670, 0.0265230, -0.0799420, 0.3477530, -1.1962950, -0.1291690, 1.6717400, -0.1548040, -0.8066650, -0.1591940
c, 1.10, 0.0103860, -0.0201920, -0.0425740, 0.4853910, -3.0474380, 3.9852690, -0.7191050, -1.8265930, 0.9270640, 0.3635220
c, 1.10, -0.0003110, 0.0043080, -0.0177850, 0.0831780, -0.1697580, -0.1738130, -0.0704300, 0.1836310, 0.4407300, 0.4297890
c, 11.11, 1.0000000
c, 12.12, 1.0000000
d, PT , 64.4513000, 19.3509000, 12.0833000, 5.9549800, 1.9173700, 1.0249700, 0.5295130, 0.2633060, 0.1253540, 0.0558110, 0.0248000
c, 1.9, 0.0000680, -0.0005040, 0.0083040, -0.0611390, 0.1964910, 0.3325240, 0.3345920, 0.2408270, 0.1146710
c, 1.9, 0.0000130, -0.0007420, -0.0189880, 0.1919930, -1.6656810, 1.4998920, 0.6224520, -1.3952750, 0.2067550
c, 1.9, 0.0004210, -0.0050290, 0.0243060, -0.1285460, 0.6635180, 0.3912420, -0.9712290, -0.3239450, 0.5670810
c, 1.9, -0.0001260, 0.0011530, -0.0110570, 0.0760890, -0.2885010, -0.4787580, -0.0290100, 0.4365010, 0.4392350
c, 10.10, 1.0000000
c, 11.11, 1.0000000
f, PT , 1.298434361350E+00, 4.736308691316E-01, 1.784103314294E-01
c, 1.1, 1.000000E+00
c, 2.2, 1.000000E+00
c, 3.3, 1.000000E+00
g, PT , 1.480403148243E+00, 4.516999058091E-01
c, 1.1, 1.000000E+00
c, 2.2, 1.000000E+00
```

```
! gold              (15s,12p,11d,3f,2g) -> [7s,7p,6d,3f,2g]
s, AU , 1403.8400000, 98.8655000, 61.6103000, 38.4670000, 24.0230000, 14.9251000, 7.3979500, 4.6219200, 1.7196100, 0.9556040, 0.4955840, 0.2037700, 0.0883320, 0.0383800,
        0.0167000
c, 1.14, 0.0000090, 0.0007400, -0.0059430, 0.0384440, -0.1847220, 0.4326540, -0.3385500, -0.6038720, 0.6213980, 0.6413890, 0.1742400, 0.0084930, 0.0015920, 0.0007130
c, 1.14, -0.0000030, -0.0002870, 0.0021480, -0.0132580, 0.0639000, -0.1539000, 0.1370550, 0.2150480, -0.2974980, -0.3719440, -0.1326630, 0.3601880, 0.6234710, 0.2540440
c, 1.14, -0.0000060, -0.0006540, 0.0045960, -0.0269110, 0.1284200, -0.3115220, 0.2723770, 0.5006430, -0.8481520, -1.3047410, 1.4622140, 1.1438780, -0.7584210, -0.5634080
c, 1.14, -0.0000160, 0.0014070, -0.0063250, -0.0005930, 0.1074110, -0.3455100, 0.2038070, 1.1112970, -3.1991300, 0.8134510, 3.1814680, -2.4541020, -0.4960330, 1.2260410
c, 1.14, 0.0000380, -0.0158500, 0.0864280, -0.2655710, 0.6411340, -1.0951470, 1.1586130, 1.0457890, -8.3301350, 11.8469770, -5.9776210, -0.7003390, 3.1033340, -1.8293480
c, 14.14, 1.0000000
c, 15.15, 1.0000000
p, AU , 38.2384000, 23.8975000, 14.9411000, 6.0974600, 1.7764500, 0.9433330, 0.5075860, 0.2788190, 0.1265610, 0.0576300, 0.0258940, 0.0116000
c, 1.11, 0.0025440, -0.0212200, 0.0745370, -0.2904710, 0.4859980, 0.4668230, 0.1905620, 0.0368290, 0.0050260, -0.0002420, 0.0001340
c, 1.11, -0.0013070, 0.0119300, -0.0448560, 0.1909790, -0.4386610, -0.3960480, 0.1302710, 0.6604720, 0.4090810, 0.0306010, 0.0046490
c, 1.11, -0.0045570, 0.0286310, -0.0879770, 0.3577880, -1.2377500, -0.0700640, 1.6256530, -0.1061240, -0.8142740, -0.1729260, 0.0018690
c, 1.11, 0.0106050, -0.0205480, -0.0480130, 0.4932400, -3.1135120, 4.1463100, -0.8180840, -1.7883020, 0.8552750, 0.4014420, -0.0217970
c, 1.11, -0.0003580, 0.0046250, -0.0194900, 0.0854730, -0.1760270, -0.1709450, -0.0705780, 0.1781830, 0.4375940, 0.4357120, 0.1344980
c, 11.11, 1.0000000
c, 12.12, 1.0000000
d, AU , 63.8112000, 20.7582000, 12.9143000, 6.3557100, 2.0708700, 1.1376900, 0.6067780, 0.3124420, 0.1540440, 0.0711190, 0.0328000
c, 1.10, 0.0000800, -0.0007480, 0.0095170, -0.0660540, 0.1946510, 0.3284690, 0.3311780, 0.2382780, 0.1126730, 0.0216860
c, 1.10, -0.0001870, 0.0018800, -0.0144830, 0.0924210, -0.3364040, -0.5298960, 0.0326050, 0.4869650, 0.3971970, 0.1017900
c, 1.10, 0.0006710, -0.0078160, 0.0333940, -0.1592270, 0.8199200, 0.2962380, -1.2094240, -0.0975860, 0.6326270, 0.2788380
c, 1.10, 0.0009760, -0.0111920, -0.0000080, 0.2043670, -2.0945970, 2.4496430, -0.1199040, -1.3458080, 0.4747170, 0.4927670
c, 10.10, 1.0000000
c, 11.11, 1.0000000
f, AU , 1.807548670317E+00, 7.043037487688E-01, 2.691348885711E-01
c, 1.1, 1.000000E+00
c, 2.2, 1.000000E+00
c, 3.3, 1.000000E+00
g, AU , 1.444973891111E+00, 4.777602353684E-01
c, 1.1, 1.000000E+00
c, 2.2, 1.000000E+00
```

```
! mercury             (15s,12p,11d,3f,2g) -> [7s,7p,6d,3f,2g]
s, HG , 8630.8100000, 1283.2600000, 283.3070000, 34.6635000, 21.5638000, 13.4091000, 7.8202700, 4.8853400, 1.7671400, 0.9649210, 0.4918620, 0.2114770, 0.0913810, 0.0396610,
        0.0172000
c, 1.14, 0.0000090, 0.0000660, 0.0002510, 0.0149190, -0.1362110, 0.4613660, -0.3696460, -0.6293820, 0.6764100, 0.6278350, 0.1403450, 0.0043010, 0.0002370, -0.0000350
c, 1.14, 0.0000040, 0.0000250, 0.0000970, 0.0051520, -0.0484270, 0.1726080, -0.1646160, -0.2168790, 0.3333150, 0.3795260, 0.0969840, -0.3986080, -0.6167610, -0.2234630
c, 1.14, 0.0000080, 0.0000550, 0.0002390, 0.0101900, -0.1020190, 0.3774670, -0.3523890, -0.5798870, 1.2286510, 1.1010790, -1.8511230, -0.8243020, 0.9526400, 0.3295130
c, 1.14, 0.0000090, -0.0005590, 0.0017000, -0.2081090, 1.1201160, -2.9639790, 4.5463850, -1.7965670, -7.5297170, 14.3323400, -11.4744850, 4.7479960, -0.3556550, -0.7354960
c, 1.14, 0.0000130, 0.0000650, 0.0004170, 0.0070780, -0.1058240, 0.4418890, -0.2909040, -1.2906930, 4.5311250, -3.1426130, -2.1864450, 3.5030800, -0.9786360, -0.5691660
c, 14.14, 1.0000000
c, 15.15, 1.0000000
p, HG , 38.2938000, 23.9271000, 14.9555000, 6.3153600, 1.8977500, 1.0214900, 0.5518790, 0.2926970, 0.1304920, 0.0586130, 0.0260970, 0.0116000
c, 1.11, 0.0026410, -0.0212850, 0.0778820, -0.2996290, 0.4796160, 0.4704650, 0.1985100, 0.0370840, 0.0038680, -0.0000090, 0.0000620
c, 1.11, -0.0011620, 0.0108590, -0.0440970, 0.1881240, -0.4085970, -0.3911550, 0.0907750, 0.6711120, 0.4206810, 0.0315370, 0.0045270
c, 1.11, -0.0045930, 0.0282990, -0.0904990, 0.3639660, -1.1777460, -0.2063620, 1.6160990, 0.0022430, -0.8518630, -0.1487560, -0.0010820
c, 1.11, -0.0075320, 0.0104010, 0.0657390, -0.5283100, 3.1374050, -3.9142240, 0.4368260, 1.9816000, -0.9444140, -0.3385740, 0.0138650
c, 1.11, -0.0002970, 0.0041670, -0.0192670, 0.0849230, -0.1685650, -0.1618750, -0.0744770, 0.1721280, 0.4338950, 0.4398990, 0.1393980
c, 11.11, 1.0000000
c, 12.12, 1.0000000
d, HG , 70.1490000, 21.6955000, 13.4986000, 6.7047800, 2.1920000, 1.2171500, 0.6609710, 0.3488820, 0.1770630, 0.0842580, 0.0401000
c, 1.10, 0.0000870, -0.0006170, 0.0095880, -0.0672710, 0.2029970, 0.3331230, 0.3268120, 0.2283160, 0.1044170, 0.0195310
c, 1.10, -0.0002170, 0.0020040, -0.0161650, 0.1027230, -0.4032390, -0.5523160, 0.1242760, 0.5170710, 0.3493250, 0.0660530
c, 1.10, -0.0023960, 0.0314370, -0.0451680, -0.1681110, 2.4674650, -3.5155560, 1.2021150, 1.0535060, -0.7529190, -0.2875250
c, 1.10, -0.0007190, 0.0088660, -0.0384430, 0.1785180, -0.9959520, -0.0997170, 1.3725970, -0.1544020, -0.6751060, -0.1668330
c, 10.10, 1.0000000
c, 11.11, 1.0000000
f, HG , 2.030532756923E+00, 7.776783344557E-01, 2.972966393808E-01
c, 1.1, 1.000000E+00
c, 2.2, 1.000000E+00
c, 3.3, 1.000000E+00
g, HG , 1.828521200239E+00, 5.461852907135E-01
c, 1.1, 1.000000E+00
c, 2.2, 1.000000E+00
```

The following pseudopotentials are used in MOLPRO format:

ECP28MDF: Y-Cd Atoms

ECP,Y,28,4,3;
1; 2,1.000000,0.000000;
2; 2,7.858275,135.134974; 2,3.382128,15.411632;
4; 2,6.849791,29.251437; 2,6.710092,58.508363; 2,3.042159,3.780243; 2,2.937330,7.676547;
4; 2,5.416315,11.849911; 2,5.333416,17.778103; 2,1.976212,2.062383; 2,1.961111,3.075654;
2; 2,5.028590,-6.928078; 2,5.005582,-9.155099;
4; 2,6.849791,-58.502875; 2,6.710092,58.508363; 2,3.042159,-7.560487; 2,2.937330,7.676547;
4; 2,5.416315,-11.849911; 2,5.333416,11.852069; 2,1.976212,-2.062383; 2,1.961111,2.050436;
2; 2,5.028590,4.618718; 2,5.005582,-4.577550;

ECP,Zr,28,4,3;
1; 2,1.000000,0.000000;
2; 2,8.636528,150.242994; 2,3.717639,18.780036;
4; 2,7.626728,33.192791; 2,7.453207,66.389039; 2,3.358389,4.620726; 2,3.229738,9.260270;
4; 2,5.938086,13.993383; 2,5.825544,20.995882; 2,2.205019,2.285166; 2,2.206292,3.441260;
2; 2,4.800215,-5.239320; 2,4.798992,-6.987424;
4; 2,7.626728,-66.385582; 2,7.453207,66.389039; 2,3.358389,-9.241452; 2,3.229738,9.260270;
4; 2,5.938086,-13.993383; 2,5.825544,13.997255; 2,2.205019,-2.285166; 2,2.206292,2.294174;
2; 2,4.800215,3.492880; 2,4.798992,-3.493712;

ECP,Nb,28,4,3;
1; 2,1.000000,0.000000;
2; 2,9.376578,165.156736; 2,4.043572,21.823951;
4; 2,8.363609,37.249284; 2,8.166898,74.507389; 2,3.693075,5.439486; 2,3.551047,10.913252;
4; 2,6.689108,15.214549; 2,6.537193,22.833508; 2,2.551118,3.000052; 2,2.567896,4.553734;
2; 2,5.568285,-6.281354; 2,5.671372,-8.755644;
4; 2,8.363609,-74.498569; 2,8.166898,74.507389; 2,3.693075,-10.878972; 2,3.551047,10.913252;
4; 2,6.689108,-15.214549; 2,6.537193,15.222338; 2,2.551118,-3.000052; 2,2.567896,3.035823;
2; 2,5.568285,4.187569; 2,5.671372,-4.377822;

ECP,Mo,28,4,3;
1; 2,1.000000,0.000000;
2; 2,10.097000,180.076853; 2,4.375670,24.715920;
4; 2,9.126564,41.227678; 2,8.863223,82.452670; 2,4.044948,6.345092; 2,3.866657,12.458423;
4; 2,7.535754,19.308744; 2,7.278976,28.977674; 2,2.763205,3.189516; 2,2.772085,4.700169;
2; 2,6.306633,-7.178888; 2,6.356448,-9.745978;
4; 2,9.126564,-82.455357; 2,8.863223,82.452670; 2,4.044948,-12.690183; 2,3.866657,12.458423;
4; 2,7.535754,-19.308744; 2,7.278976,19.318449; 2,2.763205,-3.189516; 2,2.772085,3.133446;
2; 2,6.306633,4.785926; 2,6.356448,-4.872989;

ECP,Tc,28,4,3;
1; 2,1.000000,0.000000;
2; 2,10.799369,195.091303; 2,4.707257,27.340413;
4; 2,9.826699,45.060812; 2,9.561115,90.117295; 2,4.378578,7.070517; 2,4.190907,14.048019;
4; 2,8.336045,21.657755; 2,8.244533,32.471959; 2,3.093707,3.789525; 2,3.168722,6.053430;
2; 2,7.040940,-7.995623; 2,7.069473,-10.745696;
4; 2,9.826699,-90.121625; 2,9.561115,90.117295; 2,4.378578,-14.141034; 2,4.190907,14.048019;
4; 2,8.336045,-21.657755; 2,8.244533,21.647973; 2,3.093707,-3.789525; 2,3.168722,4.035620;
2; 2,7.040940,5.330415; 2,7.069473,-5.372848;

ECP,Ru,28,4,3;
1; 2,1.000000,0.000000;
2; 2,11.500590,209.786493; 2,5.068575,30.214307;
4; 2,10.532634,48.751244; 2,10.192010,97.496529; 2,4.734892,7.860188; 2,4.509065,15.329751;
4; 2,8.877977,26.967506; 2,8.766122,40.432303; 2,3.170196,3.340758; 2,3.228851,5.256352;
2; 2,7.820249,-8.847525; 2,7.839647,-11.835518;
4; 2,10.532634,-97.502488; 2,10.192010,97.496529; 2,4.734892,-15.720375; 2,4.509065,15.329751;
4; 2,8.877977,-26.967506; 2,8.766122,26.954869; 2,3.170196,-3.340758; 2,3.228851,3.504235;
2; 2,7.820249,5.898350; 2,7.839647,-5.917759;

```
ECP,Rh,28,4,3;
1; 2,1.000000,0.000000;
2; 2,12.194816,225.312054; 2,5.405137,32.441582;
4; 2,11.280755,52.872826; 2,10.927248,105.745526; 2,5.090117,8.619344; 2,4.851832,16.973459;
4; 2,9.136337,25.108501; 2,8.964808,37.695731; 2,3.643612,4.202584; 2,3.636007,6.292790;
2; 2,8.616228,-9.673568; 2,8.629435,-12.899847;
4; 2,11.280755,-105.745651; 2,10.927248,105.745526; 2,5.090117,-17.238687; 2,4.851832,16.973459;
4; 2,9.136337,-25.108501; 2,8.964808,25.130487; 2,3.643612,-4.202584; 2,3.636007,4.195193;
2; 2,8.616228,6.449046; 2,8.629435,-6.449924;
```

```
ECP,Rh,28,4,3;
1; 2,1.000000,0.000000;
2; 2,12.194816,225.312054; 2,5.405137,32.441582;
4; 2,11.280755,52.872826; 2,10.927248,105.745526; 2,5.090117,8.619344; 2,4.851832,16.973459;
4; 2,9.136337,25.108501; 2,8.964808,37.695731; 2,3.643612,4.202584; 2,3.636007,6.292790;
2; 2,8.616228,-9.673568; 2,8.629435,-12.899847;
4; 2,11.280755,-105.745651; 2,10.927248,105.745526; 2,5.090117,-17.238687; 2,4.851832,16.973459;
4; 2,9.136337,-25.108501; 2,8.964808,25.130487; 2,3.643612,-4.202584; 2,3.636007,4.195193;
2; 2,8.616228,6.449046; 2,8.629435,-6.449924;
```

```
ECP,Pd,28,4,3;
1; 2,1.000000,0.000000;
2; 2,12.798825,240.262789; 2,5.800528,34.729961;
4; 2,11.874697,56.746929; 2,11.474335,113.444417; 2,5.515999,9.345639; 2,5.248043,18.345447;
4; 2,8.502212,28.595554; 2,7.983324,43.453921; 2,3.107628,1.852286; 2,2.476734,1.406765;
2; 2,9.679571,-10.987255; 2,9.691349,-14.626190;
4; 2,11.874697,-113.493859; 2,11.474335,113.444417; 2,5.515999,-18.691279; 2,5.248043,18.345447;
4; 2,8.502212,-28.595554; 2,7.983324,28.969281; 2,3.107628,-1.852286; 2,2.476734,0.937844;
2; 2,9.679571,7.324837; 2,9.691349,-7.313095;
```

```
ECP,Ag,28,4,3;
1; 2,1.000000,0.000000;
2; 2,12.567714,255.054771; 2,6.997662,36.983393;
4; 2,11.316496,60.715705; 2,10.958063,121.443889; 2,7.111400,10.171866; 2,6.773319,20.486564;
4; 2,8.928437,29.504938; 2,11.102567,44.018736; 2,5.543212,5.368333; 2,3.928835,7.408375;
2; 2,11.012913,-12.623403; 2,11.019898,-16.764327;
4; 2,11.316496,-121.431411; 2,10.958063,121.443889; 2,7.111400,-20.343733; 2,6.773319,20.486564;
4; 2,8.928437,-29.504938; 2,11.102567,29.345824; 2,5.543212,-5.368333; 2,3.928835,4.938916;
2; 2,11.012913,8.415602; 2,11.019898,-8.382163;
```

```
ECP,Cd,28,4,3;
1; 2,1.000000,0.000000;
2; 2,13.355176,270.039448; 2,7.308378,38.877766;
4; 2,12.659728,64.607470; 2,12.289639,129.219445; 2,6.786176,10.622558; 2,6.400743,21.265046;
4; 2,11.161722,31.663965; 2,11.219615,47.489216; 2,4.537733,5.186200; 2,4.335727,7.566063;
2; 2,11.478986,-12.632785; 2,11.487027,-16.760171;
4; 2,12.659728,-129.214939; 2,12.289639,129.219445; 2,6.786176,-21.245116; 2,6.400743,21.265046;
4; 2,11.161722,-31.663965; 2,11.219615,31.659477; 2,4.537733,-5.186200; 2,4.335727,5.044042;
2; 2,11.478986,8.421857; 2,11.487027,-8.380086;
```

ECP60MDF: Hf-Hg Atoms

```
ECP, HF, 60, 5 ;
1; !  ul potential
2,1.00000000,0.00000000;
3; !  s-ul potential
2,10.24885600,478.23740400;
4,10.72912700,0.38802400;
2,1.96485600,-1.07123200;
6; !  p-ul potential
2,8.68346200,101.04681800;
2,7.91692000,212.19776100;
4,9.37924300,0.13264600;
4,8.24420900,0.45310800;
2,1.59668600,-0.40791400;
2,1.71028100,-0.56181700;
6; !  d-ul potential
2,5.18955700,43.11546700;
2,5.25322900,65.93473200;
4,5.93787100,0.02591200;
4,5.71290600,0.03374100;
2,1.01485600,-0.28057900;
2,0.47371200,-0.08484900;
2; !  f-ul potential
2,1.91260800,4.81180100;
2,1.88074000,6.41857400;
2; !  g-ul potential
2,2.72418000,-4.63658500;
2,2.70895300,-5.76728800;

ECP, Ta, 60, 5 ;
1; !  ul potential
2,1.00000000,0.00000000;
3; !  s-ul potential
2,10.31806900,454.60064900;
4,10.54026700,2.83797500;
2,2.57472600,-0.81473600;
6; !  p-ul potential
2,8.74334200,96.91078300;
2,7.91622300,195.85043200;
4,9.27573600,4.81252400;
4,8.10167500,6.33851200;
2,2.07712700,-0.45917300;
2,2.75037200,-0.64458600;
6; !  d-ul potential
2,5.44731400,45.96997600;
2,5.21254500,69.63897200;
4,5.88435800,0.80293300;
4,5.64957900,0.42959500;
2,1.38818000,-0.30722700;
2,1.29439800,-0.46156000;
2; !  f-ul potential
2,2.16127500,5.75777300;
2,2.12593900,7.67816700;
2; !  g-ul potential
2,3.14592000,-5.68406600;
2,3.12794200,-7.06231300;
```

```
ECP, W, 60, 5 ;
1; !  ul potential
2,1.00000000,0.00000000;
2; !  s-ul potential
2,11.06379500,419.22759900;
2,8.21764100,41.19130700;
6; !  p-ul potential
2,9.33818800,107.34811000;
2,8.43044800,214.69956800;
4,9.49002000,0.02544200;
4,9.48994700,0.05189500;
2,1.88299700,-0.11718400;
2,1.90697200,0.29668900;
6; !  d-ul potential
2,6.20543300,58.88127900;
2,6.12215700,98.68355600;
4,6.27455600,0.01953700;
4,6.22637500,0.02195600;
2,1.96387500,-0.08857700;
2,1.88828700,-0.20972600;
2; !  f-ul potential
2,2.30795300,6.23247200;
2,2.27060900,8.31134500;
2; !  g-ul potential
2,3.58349100,-6.80294400;
2,3.56251500,-8.44323200;

ECP,Re,60,5,4;
1; 2,1.000000,0.000000;
2; 2,12.163814,421.970300; 2,7.107595,50.134439;
4; 2,9.684597,88.481910; 2,9.476214,176.787220; 2,7.668066,10.434338; 2,5.055156,20.458743;
4; 2,6.509888,43.162431; 2,6.091216,64.767759; 2,4.164006,5.340301; 2,4.407379,8.243332;
2; 2,2.562658,7.244543; 2,2.521549,9.659266;
2; 2,4.034599,-7.974940; 2,4.009628,-9.882736;
4; 2,9.684597,-176.963820; 2,9.476214,176.787220; 2,7.668066,-20.868677; 2,5.055156,20.458743;
4; 2,6.509888,-43.162431; 2,6.091216,43.178506; 2,4.164006,-5.340301; 2,4.407379,5.495555;
2; 2,2.562658,-4.829695; 2,2.521549,4.829633;
2; 2,4.034599,3.987470; 2,4.009628,-3.953095;

ECP, Os, 60, 5 ;
1; !  ul potential
2,1.00000000,0.00000000;
2; !  s-ul potential
2,13.00200100,424.39037600;
2,6.96276300,57.12250300;
4; !  p-ul potential
2,10.49894100,88.27900300;
2,9.99252300,176.48606400;
2,6.58824800,11.71501200;
2,5.03716700,22.91500600;
4; !  d-ul potential
2,7.08344100,44.77770800;
2,6.72117900,67.15364800;
2,3.79462600,5.55038700;
2,3.74812500,8.22010400;
2; !  f-ul potential
2,2.82627600,8.32833200;
2,2.78119800,11.10310200;
2; !  g-ul potential
2,4.49698100,-9.18134300;
2,4.46773800,-11.36160400;
```

```
ECP, Ir, 60, 5 ;
1; !  ul potential
2,1.00000000,0.00000000;
2; !  s-ul potential
2,13.83147400,426.95814900;
2,7.04791400,65.33881300;
4; !  p-ul potential
2,11.10643300,88.07544200;
2,10.45655200,176.12477000;
2,6.37961400,12.68226000;
2,5.06472600,24.63594900;
4; !  d-ul potential
2,7.54875400,46.43325800;
2,7.26530800,69.63807400;
2,3.80096900,6.15024800;
2,3.71828000,9.46356000;
2; !  f-ul potential
2,3.09870600,9.48361000;
2,3.04946100,12.64274200;
2; !  g-ul potential
2,4.96979000,-10.41069700;
2,4.93561400,-12.86319600;

ECP, Pt, 60, 5 ;
1; !  ul potential
2,1.00000000,0.00000000;
2; !  s-ul potential
2,14.60450000,429.64608700;
2,7.21828700,73.15688400;
4; !  p-ul potential
2,11.57716200,88.02291700;
2,10.88384300,175.99819600;
2,6.42440300,13.68227400;
2,5.22419800,27.41465100;
4; !  d-ul potential
2,7.69961000,43.55785200;
2,7.55080800,65.36910800;
2,3.96116400,7.01859600;
2,3.87277700,11.39173300;
2; !  f-ul potential
2,3.37986900,10.71022000;
2,3.32625500,14.27812500;
2; !  g-ul potential
2,5.45202000,-11.65174900;
2,5.41258500,-14.37552500;

ECP, Au, 60, 5 ;
1; !  ul potential
2,1.0000000,0.0000000;
2; !  s-ul potential
2,13.5232180,426.6418670;
2,6.2643840,36.8006680;
4; !  p-ul potential
2,11.4138670,87.0020910;
2,10.3292150,174.0043700;
2,5.7074240,8.8706100;
2,4.8281650,17.9024380;
4; !  d-ul potential
2,7.4309630,49.8836550;
2,8.3219900,74.6845490;
2,4.6096420,6.4862270;
2,3.5115070,9.5468210;
2; !  f-ul potential
2,3.0846390,8.7916400;
2,3.0247430,11.6584560;
2; !  g-ul potential
2,3.9784420,-5.2343370;
2,4.0114910,-6.7381420;
```

```
ECP, Hg, 60, 5 ;
1; !  ul potential
2,1.0000000,0.0000000;
2; !  s-ul potential
2,12.4130710,275.7747970;
2,6.8979130,49.2678980;
4; !  p-ul potential
2,11.3103200,80.5069840;
2,10.2107730,161.0348240;
2,5.9398040,9.0834160;
2,5.0197550,18.3677730;
4; !  d-ul potential
2,8.4078950,51.1372560;
2,8.2140860,76.7074590;
2,4.0126120,6.5618210;
2,3.7953980,9.8180700;
2; !  f-ul potential
2,3.2731060,9.4290010;
2,3.2083210,12.4948560;
2; !  g-ul potential
2,4.4852960,-6.3384140;
2,4.5132000,-8.0998630;
```
